# Repurposing proteasome inhibitors for improved treatment of triple-negative breast cancer

**DOI:** 10.1038/s41420-024-01819-5

**Published:** 2024-01-29

**Authors:** Peter Larsson, Daniella Pettersson, Maxim Olsson, Sithumini Sarathchandra, Alexandra Abramsson, Henrik Zetterberg, Ella Ittner, Eva Forssell-Aronsson, Anikó Kovács, Per Karlsson, Khalil Helou, Toshima Z. Parris

**Affiliations:** 1 Department of Oncology, Institute of Clinical Sciences, Sahlgrenska Academy, University of Gothenburg, Gothenburg, Sweden; 2 Sahlgrenska Center for Cancer Research, Sahlgrenska Academy, University of Gothenburg, Gothenburg, Sweden; 3 Department of Medical Radiation Sciences, Institute of Clinical Sciences, Sahlgrenska Academy, University of Gothenburg, Gothenburg, Sweden; 4 Department of Chemistry and Molecular Biology, University of Gothenburg, Gothenburg, Sweden; 5 Department of Psychiatry and Neurochemistry, Institute of Neuroscience and Physiology, Sahlgrenska Academy, University of Gothenburg, Gothenburg, Sweden; 6 Department of Neurodegenerative Disease, UCL Institute of Neurology, Queen Square, London, UK; 7 Clinical Neurochemistry Laboratory, Sahlgrenska University Hospital, Mölndal, Sweden; 8 Dementia Research Institute, London, UK; 9 Hong Kong Center for Neurodegenerative Diseases, Clear Water Bay, Hong Kong China; 10 Wisconsin Alzheimer’s Disease Research Center, University of Wisconsin School of Medicine and Public Health, University of Wisconsin-Madison, Madison, WI USA; 11 Department of Medical Physics and Biomedical Engineering, Sahlgrenska University Hospital, Gothenburg, Sweden; 12 Department of Clinical Pathology, Sahlgrenska University Hospital, Gothenburg, Sweden; 13 Department of Oncology, Sahlgrenska University Hospital, Gothenburg, Sweden

**Keywords:** Breast cancer, Breast cancer, Chemotherapy

## Abstract

Triple-negative breast cancer (TNBC) is associated with poor prognosis and limited treatment options due to the lack of important receptors (estrogen receptor [ER], progesterone receptor [PR], and human epidermal growth factor receptor 2 [HER2]) used for targeted therapy. However, high-throughput in vitro drug screening of cell lines is a powerful tool for identifying effective drugs for a disease. Here, we determine the intrinsic chemosensitivity of TNBC cell lines to proteasome inhibitors (PIs), thereby identifying potentially potent 2-drug combinations for TNBC. Eight TNBC cell lines (BT-549, CAL-148, HCC1806, HCC38, HCC70, MDA-MB-436, MDA-MB-453, and MDA-MB-468) and two controls (MCF-10A and MCF-7) were first exposed to 18 drugs (11 PIs and 7 clinically relevant chemotherapeutic agents) as monotherapy, followed by prediction of potent 2-drug combinations using the IDACombo pipeline. The synergistic effects of the 2-drug combinations were evaluated with SynergyFinder in four TNBC cell lines (CAL-148, HCC1806, HCC38, and MDA-MB-468) and three controls (BT-474, MCF-7, and T47D) in vitro, followed by further evaluation of tumor regression in zebrafish tumor models established using HCC1806 and MCF-7 cells. Monotherapy identified nine effective drugs (bortezomib, carfilzomib, cisplatin, delanzomib, docetaxel, epoxomicin, MLN-2238, MLN-9708, and nedaplatin) across all cell lines. PIs (e.g., bortezomib, delanzomib, and epoxomicin) were highly potent drugs in TNBC cells, of which bortezomib and delanzomib inhibited the chymotrypsin-like activity of the 20 S proteasome by 100% at 10 µM. Moreover, several potent 2-drug combinations (e.g., bortezomib+nedaplatin and epoxomicin+epirubicin) that killed virtually 100% of cells were also identified. Although HCC1806- and MCF-7-derived xenografts treated with bortezomib+nedaplatin and carboplatin+paclitaxel were smaller, HCC1806 cells frequently metastasized to the trunk region. Taken together, we show that PIs used in combination with platinum agents or topoisomerase inhibitors exhibit increased efficiency with almost 100% inhibition in TNBC cell lines, indicating that PIs are therefore promising compounds to use as combination therapy for TNBC.

## Introduction

Triple-negative breast cancer (TNBC) is a heterogeneous disease with diverse clinicopathological and biological behavior, accounting for ~15–20% of the 2.3 million new breast cancer cases worldwide [1–3]. Due to the lack of hormone (estrogen- and progesterone receptors; ER and PR) and HER2 receptor expression (hence “triple-negative”), no standard of care with targeted agents is currently available [1, 3, 4]. TNBC is therefore frequently associated with poor 5-year survival rates, rapid recurrence within 1–3 years of diagnosis, and variable response to chemo- and radiation therapy [4–8]. Moreover, therapy response has been found to depend on TNBC molecular subtype (basal-like 1 [BL1], basal-like 2 [BL2], mesenchymal-like [M], luminal androgen receptor [LAR]). BL2 and LAR have shown the lowest pathological complete response rates to chemotherapy (0% and 10%, respectively), while LAR is associated with a higher risk of relapse following radiotherapy [6, 9]. Although treatment of patients with TNBC is challenging due to tumor heterogeneity [10], TNBCs with confirmed *BRCA1* mutations can be treated with platinum agents and PARP inhibitors [3, 10–12]. Otherwise, common mainstay treatment for TNBC patients is chemotherapy including anthracyclines (e.g., doxorubicin) and taxane-based agents (e.g., paclitaxel and docetaxel) both alone or in combination with carboplatin [1, 12], which are good at early stages but less so for late-stage disease [1]. Therefore, a more in-depth analysis of TNBCs is warranted to better understand the underlying causes of drug resistance and identify potent drugs that improve treatment of this disease.

The ubiquitin-proteasome pathway is responsible for the degradation of 80-90% of proteins in the cell. Treatment with proteasome inhibitors (PIs) causes toxic accumulation of misfolded and damaged proteins, protein homeostasis stress, which in turn leads to cell cycle arrest and ultimately apoptosis. Although PIs are primarily toxic to cancer cells, normal cells can also be influenced negatively which could lead to peripheral neuropathy or cardiovascular toxicity [13, 14]. However, PIs (e.g., bortezomib) have improved survival for patients diagnosed with multiple myeloma [15]. Subsequent studies have shown that bortezomib also has an antitumoral effect in other types of cancer [15, 16]. In 2013, Petrocca et al. showed that basal-like breast cancer (including TNBC) was highly sensitive to proteasome inhibition with bortezomib compared to luminal breast cancer subtypes [17]. Bortezomib was also shown to inhibit invasiveness and metastasis for basal-like tumors in vivo. In our recent work, we identified 33 genes involved in bortezomib resistance that could be used as biomarkers to discriminate patients that may benefit from treatment with bortezomib (unpublished work). To overcome single-drug resistance, preclinical studies have shown that treatment of TNBC cells can be improved by combining oprozomib (next-generation PI) with doxorubicin, while combination therapy with ixazomib (MLN-2238) and carboplatin (platinum agent) is considered to be an effective treatment for TNBC [1, 18]. Apart from these studies, few studies have been conducted combining proteasome inhibitors with other clinically relevant drugs to improve treatment of TNBC.

In oncology, the increasing number of newly initiated clinical trials using combination therapy led to a progressive trend away from single-agent therapy (from 70% in 2007 to ~25% in 2021) [19]. Combination treatments frequently consist of several pharmaceutical drugs together or targeted therapy (e.g., PD-1/PD-L1, Epithelial growth factor receptor (EGFR), Human epidermal growth factor receptor 2 (HER2), Poly (ADP-ribose) polymerase (PARP), Phosphatidylinositol-3-kinase (PI3K), CDK4/6) together with pharmaceutical drugs. Compared to monotherapy, combination therapy will ideally result in an increase in therapeutic efficacy, synergistic effects (above individual drug potencies), and reduced drug toxicity, which in turn could improve treatment success rates or lower the drug doses needed to achieve the same biological effect. However, drug combinations may have adverse effects due to drug interactions, e.g., unwanted side effects, increased toxicity, and antagonism (below individual drug potencies). Combination treatment is also less flexible because the drugs are given in fixed concentration ratios [20–23]. To predict the efficacy of drug combinations using monotherapy data from high-throughput cancer cell line screens, computational algorithms like IDACombo, an independent drug action (IDA)-based method, have been developed [24]. The prerequisite is that the 2-drug combinations should be non-interacting drugs and the strength of the drug combination is dependent on the most potent drug. Subsequently, the SynergyFinder package in R can then be used to determine possible synergism and phenotypic responses [25].

In the current study, we evaluate the efficacy and potency of 7 clinically relevant drugs and 11 PIs as mono- and combination therapy for TNBC cells in vitro and in vivo. Using eight human TNBC lines (BT-549, CAL-148, HCC1806, HCC38, HCC70, MDA-MB-436, MDA-MB-453, MDA-MB-468) representing the four TNBC subtypes and four control cell lines (BT-474, MCF-7, MCF-10A, and T47D), we could hereby identify powerful single drugs and synergistic 2-drug combinations capable of killing TNBC cells. The zebrafish xenograft models also provided further insight into tumor regression and metastasis following treatment.

## Results

### Drug screening identifies nine potent drugs in TNBC cells

**Fig. 1:**
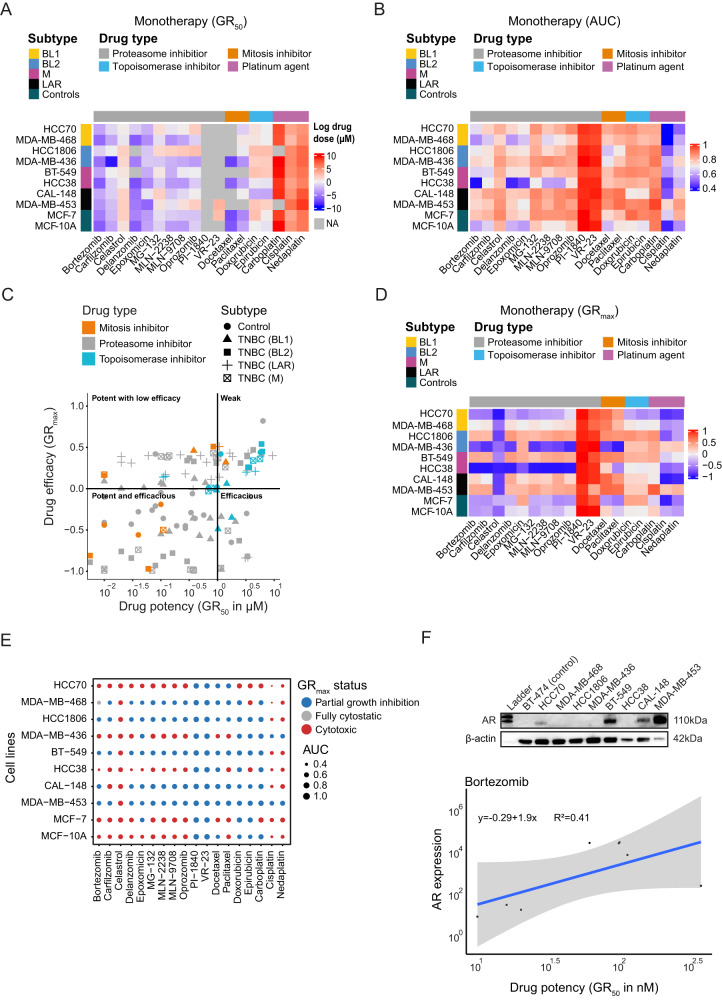
Cell line characterization and monotherapy for eight TNBC and two control cell lines. **A** and **B** show the efficiency of the 18 drugs on eight TNBC cell lines (BT-549, CAL-148, HCC1806, HCC38, HCC70, MDA-MB-436, MDA-MB-453, and MDA-MB-468) and two controls (MCF-10A and MCF-7) using growth rate inhibition (GR_50_) and area under the curve (AUC). Bortezomib, carfilzomib, cisplatin, delanzomib, docetaxel, epoxomicin, MLN-2238, MLN-9708 and nedaplatin were shown to be potent drugs (GR_50_ < 1000 nM and AUC < 0.80). **C** Scatterplot illustrating cytotoxicity with GR_max_ and GR_50_ values. Topoisomerases were shown to be weak inhibitors, while proteasome inhibitors, mitosis inhibitors, and platinum agents had an adverse effect on cell viability. **D** and **E** show the cytotoxicity of all 18 drugs at the highest tested dose. The most cytotoxic drugs were celastrol, cisplatin, and nedaplatin, and the most sensitive cell lines to the highest dose were HCC38, HCC70, and MDA-MB-436. **F** show expression of AR in the 10 cell lines. Cell lines BT-549 (M), CAL-148 (LAR), HCC70 (BL1) and MDA-MB-453 (LAR) were AR-positive. Elevated AR expression was associated with high GR_50_ values.

To evaluate the intrinsic chemosensitivity of TNBC cells to various PIs, a monotherapy drug sensitivity screen was conducted using eight TNBC breast cancer cell lines (BT-549, CAL-148, HCC1806, HCC38, HCC70, MDA-MB-436, MDA-MB-453, and MDA-MB-468) representing the four TNBC subtypes (BL1, BL2, M, and LAR) and two control cell lines (MCF-10A and MCF-7). The cells were exposed to 11 PIs and 7 other clinically relevant compounds (2 mitosis inhibitors, 3 platinum agents, and 2 topoisomerase inhibitors). Consequently, the lowest GR_50_ (growth rate inhibition concentration at GR(c) = 0.5 [26]) values (for drugs using the same doses: PIs, mitotic inhibitors, and topoisomerase inhibitors) following 24 h drug treatment were found for bortezomib (mean ± SD, 0.10 ± 0.11 µM), carfilzomib (0.44 ± 0.34 µM), delanzomib (0.079 ± 0.13 µM), docetaxel (0.0088 ± 0.0039 µM), epoxomicin (0.22 ± 0.23 µM), and MLN-2238 (0.68 ± 1.0 µM) across the TNBC cell lines and controls (bortezomib, 0.021 ± 0.0097 µM; delanzomib, 0.024 ± 0.013 µM; docetaxel, 0.0078 ± 0.0013 µM; epoxomicin, 0.085 ± 0.010 µM; Fig. 1A and Table 1). Furthermore, bortezomib (0.66 ± 0.10), carfilzomib (0.73 ± 0.10), cisplatin (0.51 ± 0.10), delanzomib (0.69 ± 0.10), docetaxel (0.79 ± 0.10), epoxomicin (0.69 ± 0.10), MLN-2238 (0.76 ± 0.10), MLN-9708 (0.79 ± 0.10), and nedaplatin (0.68 ± 0.10) had the lowest area under the dose response curve [27] (AUC) values in the TNBC cell lines and controls (bortezomib, 0.69 ± 0.10; cisplatin, 0.54 ± 0.12; epoxomicin, 0.66 ± 0.080; nedaplatin, 0.70 ± 0.13; Fig. 1B and Table 1). Using the GR_50_ and AUC values as metrics of drug potency (GR_50_ < 1.0 µM and AUC < 0.80), bortezomib, carfilzomib, cisplatin, delanzomib, docetaxel, epoxomicin, MLN-2238, MLN-9708, and nedaplatin were classified as potent drugs. Accordingly, bortezomib, carfilzomib, delanzomib, docetaxel, epoxomicin, and MLN-2238 were below the threshold and therefore considered as highly potent drugs. Although no significant difference in drug response (based on GR_50_) was found between the TNBC subtypes, both cell lines classified as BL1 (HCC70 and MDA-MB-468) and M subtype (BT-549 and HCC38) displayed the lowest AUC values for bortezomib.

**Table 1:** Drug efficacy and potency in 10 breast cell lines interpreted by IC_50_, AUC, GR_50_, and GR_max_ values.

	Drug																	
	Bortezomib	Carboplatin	Carfilzomib	Celastrol	Cisplatin	Delanzomib	Docetaxel	Doxorubicin	Epirubicin	Epoxomicin	MG 132	MLN 2238	MLN 9708	Nedaplatin	Oprozomib	Paclitaxel	PI-1840	VR-23
**IC**_**50**_ **(µM)**																		
BT-549	NA	NA	4.4	1.3	0.053	NA	NA	7.6	4.6	NA	4.3	NA	NA	0.15	NA	NA	NA	NA
CAL-148	NA	0.93	NA	3.7	0.048	NA	NA	0.22	0.20	NA	NA	NA	NA	0.32	NA	NA	NA	NA
HCC1806	0.79	NA	1.7	2.1	0.048	NA	NA	NA	NA	0.47	3.1	3	NA	0.15	6	NA	NA	NA
HCC38	0.024	0.89	0.57	1.4	0.030	0.012	NA	2.9	2.3	0.054	0.57	0.11	0.13	0.093	0.68	7.1	NA	NA
HCC70	0.81	0.45	2.1	1.8	0.022	3.3	NA	3.3	1.6	0.98	3.7	2.7	4.8	0.046	7.6	NA	NA	NA
MDA-MB-436	NA	NA	NA	3.2	0.064	NA	NA	9.7	NA	NA	NA	NA	NA	0.23	NA	NA	NA	NA
MDA-MB-453	NA	NA	NA	3.7	0.24	NA	NA	8.1	5.3	NA	NA	4.3	NA	NA	NA	NA	NA	NA
MDA-MB-468	0.054	0.47	0.46	1.0	0.015	0.077	NA	3.0	2.2	0.11	1.4	0.24	0.80	0.067	3.2	NA	NA	NA
MCF-10A	0.046	0.73	0.54	1.6	0.027	0.018	0.050	0.99	0.69	0.088	0.94	0.41	0.38	0.075	2.0	0.86	NA	NA
MCF-7	NA	NA	NA	2.8	0.16	NA	NA	1.8	1.5	1.2	NA	NA	NA	NA	NA	NA	NA	6.0
**AUC**																		
BT-549	0.66	0.85	0.67	0.82	0.57	0.73	0.79	0.89	0.88	0.73	0.83	0.75	0.80	0.66	0.81	0.87	0.99	0.98
CAL-148	0.77	0.92	0.84	0.85	0.48	0.77	0.89	0.72	0.73	0.78	0.89	0.83	0.88	0.78	0.90	0.92	1.0	1.0
HCC1806	0.70	0.90	0.73	0.81	0.48	0.74	0.64	0.87	0.86	0.73	0.82	0.75	0.79	0.66	0.85	0.72	1.0	1.0
HCC38	0.40	0.88	0.71	0.79	0.45	0.42	0.78	0.83	0.81	0.47	0.71	0.59	0.59	0.62	0.73	0.81	1.0	1.0
HCC70	0.68	0.83	0.76	0.82	0.37	0.75	0.83	0.88	0.83	0.72	0.87	0.78	0.82	0.50	0.89	0.86	1.0	0.98
MDA-MB-436	0.75	0.91	0.73	0.85	0.62	0.79	0.69	0.85	0.84	0.77	0.86	0.88	0.86	0.76	0.84	0.78	1.0	1.0
MDA-MB-453	0.75	0.98	0.77	0.87	0.75	0.76	0.87	0.88	0.90	0.70	0.87	0.81	0.88	0.86	0.84	0.93	1.0	0.99
MDA-MB-468	0.57	0.81	0.65	0.75	0.37	0.58	0.77	0.84	0.80	0.65	0.81	0.67	0.72	0.61	0.76	0.81	0.98	0.97
MCF-10A	0.59	0.90	0.66	0.81	0.42	0.62	0.63	0.72	0.69	0.58	0.78	0.72	0.72	0.57	0.79	0.73	1.0	1.0
MCF-7	0.78	0.91	0.81	0.89	0.66	0.77	0.75	0.93	0.89	0.74	0.87	0.87	0.87	0.82	0.88	0.79	1.0	0.98
**GR**_**50**_ **(µM)**																		
BT-549	0.10	0.67	1.0	1.5	0.035	NA	NA	5.6	4.3	0.13	1.4	0.14	0.84	0.081	0.94	NA	NA	NA
CAL-148	0.12	0.49	0.37	3.1	0.032	0.023	NA	0.12	0.11	0.77	3.4	0.27	0.50	0.21	1.1	1.1	NA	NA
HCC1806	0.38	NA	0.95	3.4	0.042	NA	NA	6.0	6.2	0.29	2.5	3.3	4.3	0.12	6.0	0.84	NA	NA
HCC38	0.010	0.43	0.38	1.2	0.019	0.0082	0.013	0.77	0.69	0.044	0.37	0.071	0.069	0.054	0.33	0.11	NA	NA
HCC70	0.10	0.27	0.48	1.2	0.015	0.37	NA	1.8	1.0	0.14	2.1	0.74	1.2	0.30	3.7	0.38	NA	NA
MDA-MB-436	0.021	0.87	0.0018	2.4	0.043	0.029	0.0050	3.4	3.6	0.32	0.29	0.16	0.11	0.13	0.075	0.055	NA	NA
MDA-MB-453	0.063	NA	0.22	3.4	0.095	0.027	NA	4.6	3.2	0.015	1.7	0.64	1.8	0.41	3.5	NA	NA	9319.49
MDA-MB-468	0.017	0.24	0.11	0.75	0.0075	0.011	NA	1.5	0.96	0.051	0.61	0.055	0.10	40.46	0.39	1.4	NA	NA
MCF-10A	0.012	0.76	0.11	1.8	0.028	0.012	0.0090	0.98	0.69	0.095	0.40	0.076	0.070	0.074	0.36	0.098	NA	NA
MCF-7	0.031	0.36	0.19	3.3	0.015	0.037	0.0065	5.7	1.1	0.075	0.57	0.34	0.27	0.075	0.27	0.041	NA	6334.47
**GR**_**max**_																		
BT-549	0.39	0.39	0.37	−0.51	−0.16	0.48	0.61	0.36	0.34	0.38	0.26	0.40	0.43	−0.02	0.38	0.58	1.0	0.85
CAL-148	0.17	0.03	−0.01	−0.84	−0.97	0.39	0.74	0.15	0.14	0.11	0.22	0.18	0.40	−0.93	0.14	0.51	1.0	0.95
HCC1806	0.33	0.53	0.31	−0.82	−0.72	0.50	0.56	0.54	0.45	0.34	0.31	0.39	0.47	−0.38	0.42	0.51	0.98	0.93
HCC38	−0.99	0.03	−0.99	−0.96	−0.67	−0.97	0.17	0.01	−0.03	−0.74	−0.98	−0.99	−0.99	−0.58	−0.98	−0.50	1.0	0.95
HCC70	−0.55	−0.42	−0.57	−0.98	−0.89	−0.36	0.72	−0.35	−0.49	−0.52	−0.40	−0.36	−0.34	−0.83	−0.10	0.46	1.0	0.82
MDA-MB-436	−0.76	0.41	−0.89	−0.63	0.17	−0.84	−0.81	0.26	0.26	0.37	−0.88	−0.78	−0.82	0.22	−0.93	−0.97	1.0	1.0
MDA-MB-453	0.40	0.79	0.41	−0.83	0.25	0.31	0.74	0.21	0.20	0.32	0.43	0.32	0.39	0.34	0.43	0.71	0.93	0.40
MDA-MB-468	0	0.02	0.05	−0.61	−0.46	0.09	0.64	0.06	−0.02	0.01	0.07	0.01	0.07	−0.38	0.12	0.32	0.96	0.74
MCF-10A	−0.41	0.19	−0.36	−0.65	−0.32	−0.10	0.16	0.02	0.01	−0.05	−0.36	−0.21	−0.26	−0.40	−0.06	−0.19	1.0	1.0
MCF-7	−0.43	−0.15	−0.45	−0.30	−0.96	−0.28	−0.44	0.44	0.42	0.42	−0.32	−0.29	−0.48	−0.57	−0.45	−0.56	0.97	0.82

Note: NA denotes IC_50_ and GR_50_ values (calculated using the GRmetrics package) that were removed for cases where values were (a) not reached (INF) or (b) higher than the tested drug dose range.

GR_max_ values were then evaluated as a metric of drug efficacy, thereby demonstrating that PIs, mitosis inhibitors, and platinum agents had an adverse effect on the viability of all investigated cell lines (Fig. 1C, D and Table 1). In contrast, topoisomerase inhibitors had low to weak efficiency (Fig. 1C). Although the LAR subtype was insensitive to most of the tested drugs, celastrol (GR_max_ = −0.84), cisplatin (GR_max_ = −0.97), and nedaplatin (GR_max_ = −0.93) were cytotoxic to CAL-148 cells, whereas celastrol was cytotoxic to MDA-MB-453 cells (GR_max_ = −0.83) at the highest tested dose (Fig. 1D and Table 1). Moreover, celastrol, cisplatin, nedaplatin were cytotoxic to TNBC cell lines at the highest dose, while carboplatin, doxorubicin, epirubicin, epoxomicin, PI-1840, and VR-23 showed the lowest cytotoxic effects (Fig. 1D and Table 1). Correlation analysis between GR_max_ status (i.e., partial growth inhibition, fully cytostatic, and cytotoxic) and AUC values subsequently identified ≥10 cytotoxic drugs for three TNBC cell lines (HCC38 [13 drugs], HCC70 [14 drugs], MDA-MB-436 [10 drugs]; Fig. 1E). Intriguingly, both LAR cell lines (CAL-148 [4 drugs] and MDA-MB-453 [1 drug]) and an M cell line BT-549 ([3 drugs]) were the least sensitive to the tested drugs. Cisplatin was found to be a highly cytotoxic drug with consistently low AUC values. The majority of drugs were also cytotoxic to the control cell lines (MCF-10A [12 drugs] and MCF-7 [13 drugs]; Fig. 1D, E). Androgen receptor (AR) status was confirmed in four cell lines (BT-549 [M], CAL-148 [LAR], HCC70 [BL1], and MDA-MB-453 [LAR]), which were found to be AR-positive (Fig. 1F and Supplementary Fig. 1). Furthermore, correlation analysis revealed that elevated AR expression was associated with high GR_50_ values and thus insensitivity to treatment (e.g., bortezomib; Fig. 1F).

### Bortezomib, delanzomib, MG-132, MLN-2238, and MLN-9708 inhibit 20 S proteasome β5 activity

**Fig. 2:**
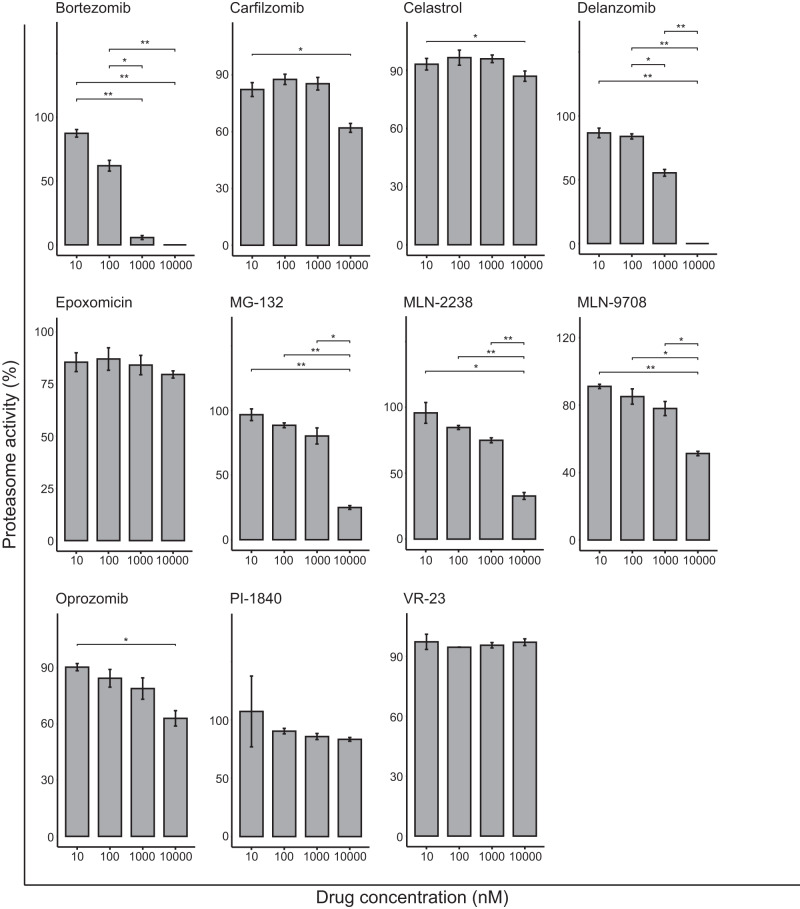
All eleven proteasome inhibitors were tested for their ability to inhibit the activity at the β5 proteasome site. Bortezomib and delanzomib show 95% and 55% inhibition at 1000 nM, and at 10000 nM they inhibit activity to 100% while MLN-9708 (51%), MLN-2238 (33%), and MG-132 (25%) inhibit the β5 site to a small extent. Statistically significant differences in the suppression of chymotrypsin-like activity in the proteasome between various doses of proteasome inhibitor were determined using the paired *t*-test. *P < 0.05; **P ≤ 0.01; ***P ≤ 0.001; ****P ≤ 0.0001.

To evaluate whether the 11 PIs inhibited the β5 catalytic site of the 20 S proteasome, MCF-7 cells were treated for 6 h at 10, 100, 1000, and 10000 nM with each proteasome inhibitor. This analysis showed that bortezomib inhibited the β5 activity to 95% and delanzomib to 55% at 1000 nM, thereby demonstrating drug potency in breast cancer and the ability to inhibit β5 activity. At 10000 nM, both drugs showed 100% inhibition of β5 activity, followed by MLN-9708 (51%), MLN-2238 (33%), and MG-132 (25%; Fig. 2). Despite its drug potency and ability to lead to the accumulation of Ub-tagged proteins (unpublished work) in breast cancer cells, epoxomicin demonstrated surprisingly poor suppression of β5 activity.

### Bortezomib + doxorubicin and bortezomib + nedaplatin are potent drug combinations in TNBC cell lines

**Fig. 3:**
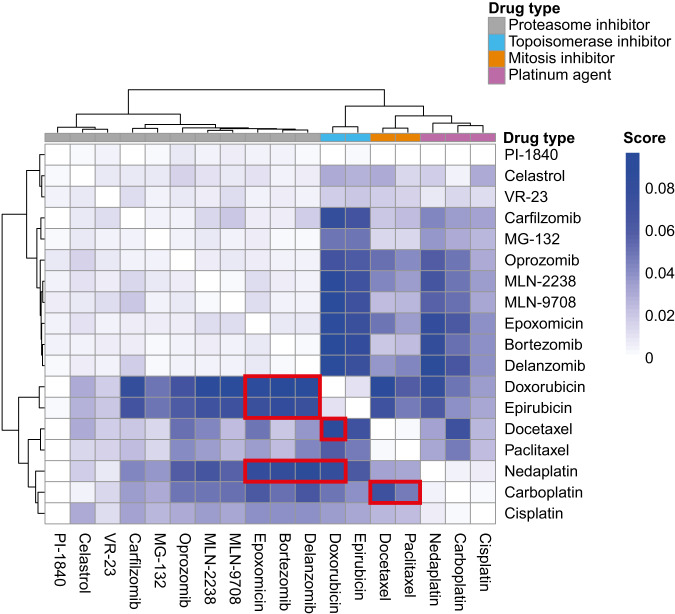
Prediction of potent 2-drug combinations by IDACombo. Data from the monotherapy experiments were evaluated by IDACombo, whereby we chose 11 combinations (bortezomib + doxorubicin, bortezomib + epirubicin, bortezomib + nedaplatin, delanzomib + doxorubicin, delanzomib + epirubicin, delanzomib + nedaplatin, epoxomicin + doxorubicin, epoxomicin + epirubicin, epoxomicin + nedaplatin, doxorubicin + docetaxel, and doxorubicin + nedaplatin) with combo scores >0.06 and two combinations (carboplatin + docetaxel and carboplatin + paclitaxel) currently used in clinical practice.

Based on the monotherapy data of the 18 drugs, we utilized the IDACombo pipeline to predict promising combination therapies. Surprisingly, 2-drug combinations between compounds from the same drug class (e.g., proteasome inhibitor+proteasome inhibitor) demonstrated relatively low IDAComboscores and thus poor predicted drug combination efficacy (Fig. 3). In total, 11 drug combinations containing compounds from different drug classes (bortezomib+doxorubicin, bortezomib+epirubicin, bortezomib+nedaplatin, delanzomib+doxorubicin, delanzomib+epirubicin, delanzomib+nedaplatin, epoxomicin+doxorubicin, epoxomicin+epirubicin, epoxomicin+nedaplatin, doxorubicin+docetaxel, and doxorubicin+nedaplatin) were selected for inclusion in the in vitro combination therapies based on IDAComboscores >0.06. Carboplatin+docetaxel and carboplatin+paclitaxel were also chosen due to their routine use in the clinical management of breast cancer.

**Fig. 4:**
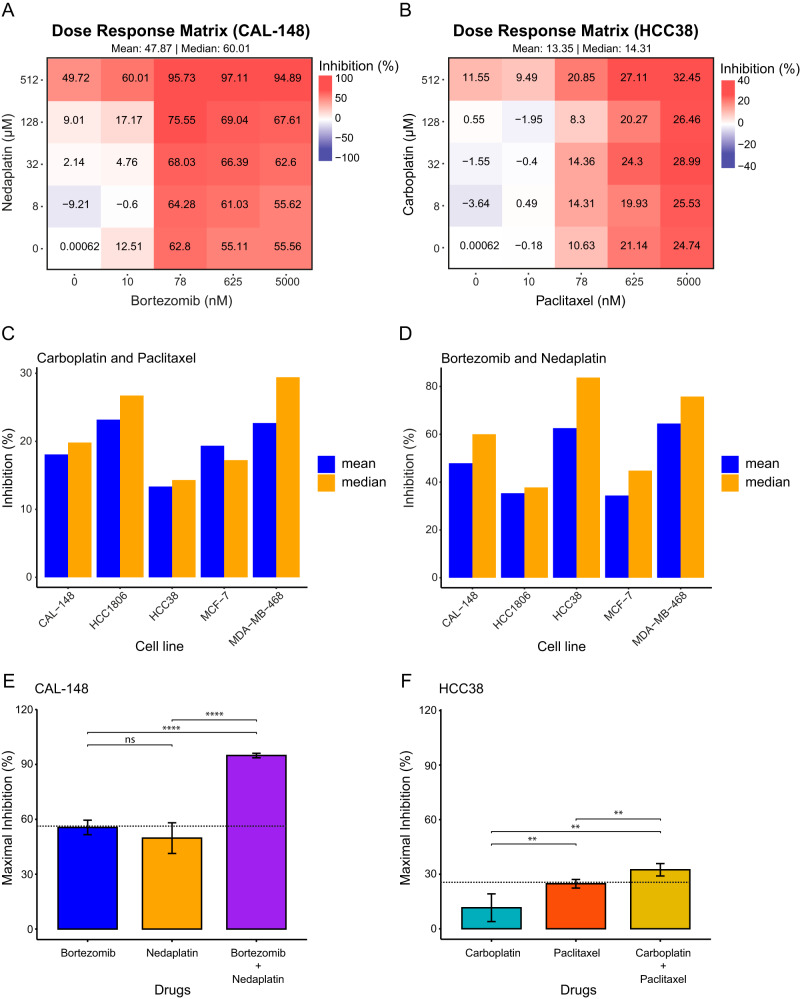
Potency of the 2-drug combinations evaluated by SynergyFinder using inhibition (%) scores. **A** Bortezomib + nedaplatin was a very potent drug combination and inhibited CAL-148 with a mean (47.9) and median (60.0) inhibition score. **B** Carboplatin + paclitaxel had the lowest inhibition scores, mean (13.4) and median (14.3). **C** Carboplatin + paclitaxel had the highest mean and median inhibition on the MDA-MB-468 cell line. **D** Bortezomib + nedaplatin had the highest inhibition on the HCC38 cell line and lowest inhibition on HCC1806. **E** Bortezomib + nedaplatin achieved 34.3% above maximal inhibition by bortezomib alone and **F** carboplatin + paclitaxel achieved 7.7% above the maximal inhibition by paclitaxel alone. Paired *t*-test was used to calculate statistically significant differences between inhibition following monotherapy or with 2-drug combinations. *P < 0.05; **P ≤ 0.01; ***P ≤ 0.001; ****P ≤ 0.0001.

To evaluate drug potency and synergistic effects for the 13 drug combinations, five cell lines (representing each TNBC subtype: CAL-148 [LAR], HCC38 [M], HCC1806 [BL2], MDA-MB-468 [BL1]; and MCF-7 control cells) were treated with each drug combination for 24 h and the results evaluated with SynergyFinder. BT-474 and T47D control cells were also treated with bortezomib+nedaplatin and carboplatin+paclitaxel. Consequently, three drug combinations (i.e., bortezomib+nedaplatin, bortezomib+doxorubicin, and epoxomicin+epirubicin) had a profound negative impact on cell survival (inhibition) across drug concentrations (mean and median inhibition values) and multiple cell lines (Fig. 4A and Supplementary Fig. 2). In particular, the dose response matrices showed high levels of inhibition for bortezomib+nedaplatin in CAL-148 (mean = 47.9%, median = 60.0%) and MDA-MB-468 cells (mean = 64.5%, median = 75.8%), bortezomib+doxorubicin in HCC38 cells (mean = 60.5%, median = 85.2%), as well as epoxomicin+epirubicin in HCC1806 (mean = 39.3%, median = 61.0%) and MCF-7 cells (mean = 35.6%, median = 45.1%; Fig. 4A and Supplementary Fig. 2). In contrast, the lowest impact on cell survival was shown for doxorubicin+nedaplatin in MCF-7 cells (mean = 16.1%, median = 1.6%), and carboplatin+paclitaxel in HCC38 cells (mean = 13.4%, median = 14.3%; Fig. 4B, C and Supplementary Fig. 2). Interestingly, bortezomib + nedaplatin showed the highest levels of inhibition for 3/5 investigated cell lines, i.e., 97.1% inhibition for CAL-148 (625 nM bortezomib + 512 µM nedaplatin), 85.1% inhibition for HCC1806 (5000 nM bortezomib + 512 µM nedaplatin), and 91.9% inhibition for MDA-MB-468 (78 nM bortezomib + 512 µM nedaplatin), though 92.9% inhibition was also shown for HCC38 (5000 nM bortezomib + 512 µM nedaplatin; Fig. 4D and Supplementary Fig. 2). Furthermore, 93.5% inhibition was found for HCC38 treated with 5000 nM bortezomib + 625 nM doxorubicin, and 73.6% inhibition for epoxomicin (625 nM) + nedaplatin (512 µM) treated MCF-7 cells. Compared to monotherapy, combination therapy frequently resulted in an increase in maximum inhibition. For example, a 34.3% increase in maximal inhibition was found for the bortezomib+nedaplatin combo in CAL-148 cells compared with monotherapy, while only a 7.7% increase in inhibition was found for the carboplatin+paclitaxel combo in HCC38 cells compared to paclitaxel, the most potent single drug (Fig. 4E, F and Supplementary Fig. 2).

**Fig. 5:**
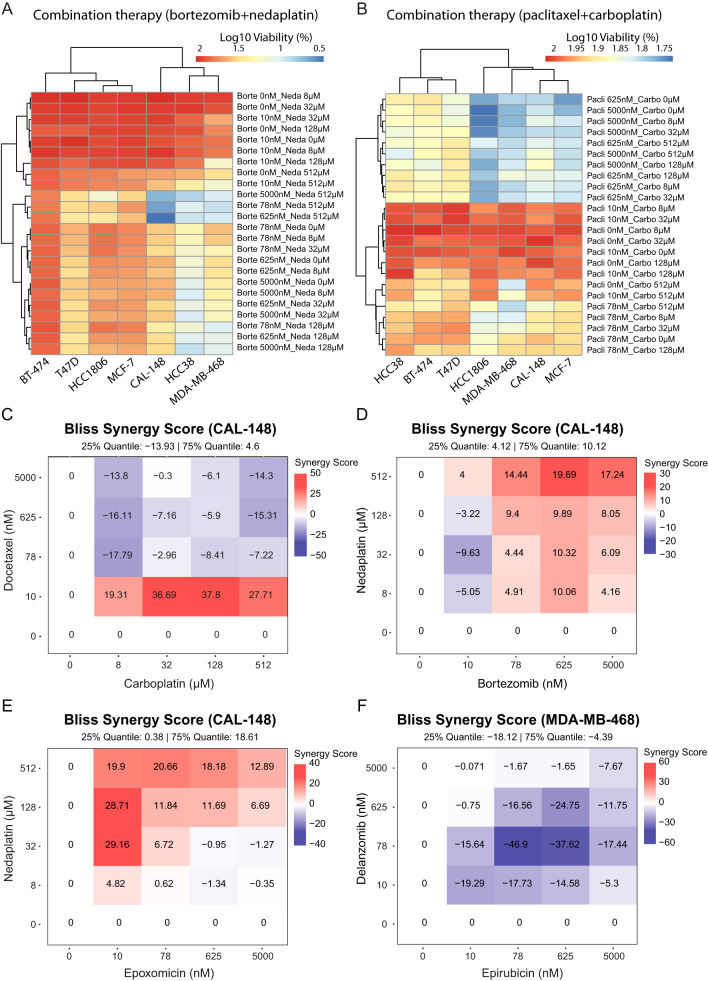
Overview of cell viability and the synergistic effect between 2-drug combinations in four TNBC cell lines (CAL-148, HCC38, HCC1806, and MDA-MB-468) and three controls (BT-474, MCF-7, and T47D). **A** The controls and HCC1806 cells were insensitive to the potent drug combination with bortezomib+nedaplatin. **B** All tested cell lines were insensitive to clinically relevant carboplatin+paclitaxel. **C** The combination with the highest synergy score was carboplatin + docetaxel on the CAL-148 cell line, achieving a synergy score of 36.7 at 32 µM/10 nM, respectively. **D** Bortezomib + nedaplatin on CAL-148 cell line had a synergy score of 19.7 at 625 nM/512 µM, respectively, and **E** epoxomicin + nedaplatin had a synergy score of 29.2 at 10 nM/32 µM, respectively. **F** Epirubicin + delanzomib showed an antagonistic score at all tested doses for MDA-MB-468. A synergy score ≥10 is considered to be strong synergy and ≤-10 as strong antagonism.

Consequently, bortezomib+nedaplatin had the lowest impact on the controls (BT-474, MCF-7, and T47D) and HCC1806 cells (Fig. 5A), while carboplatin+paclitaxel had the weakest effect across the tested cell lines (Fig. 5B). We then identified drug combinations displaying synergistic effects (i.e., multiplicative effect of single drugs as if they acted independently) based on Bliss Synergy scores ≥10. Carboplatin+docetaxel reached the threshold for all five tested cell lines (CAL-148 [synergy score 37.8], HCC38 [30.8], HCC1806 [33.0], MCF-7 [27.4], and MDA-MB-468 [18.1]). Bortezomib+nedaplatin (CAL-148 [19.7], HCC38 [11.5], HCC1806 [15.0], and MDA-MB-468 [25.4]) and epoxomicin+nedaplatin (CAL-148 [29.2], HCC38 [13.6], HCC1806 [13.0], and MDA-MB-468 [26.5]) showed synergy for the four TNBC cell lines, but not the MCF-7 cell line (Fig. 5C–E and Supplementary Fig. 3). Although the carboplatin+docetaxel combo had the highest synergy score (37.8) for CAL-148 cells, epoxomicin+nedaplatin showed the highest synergy scores across the tested concentrations (25% quantile: 0.38; 75% quantile: 18.6; Fig. 5E and Supplementary Fig. 3). Interestingly, the delanzomib+epirubicin combo was consistently antagonistic for 3/5 cell lines (HCC38, HCC1806, and MDA-MB-468; Fig. 5F).

### Combination treatment leads to tumor regression in zebrafish xenografts

**Fig. 6:**
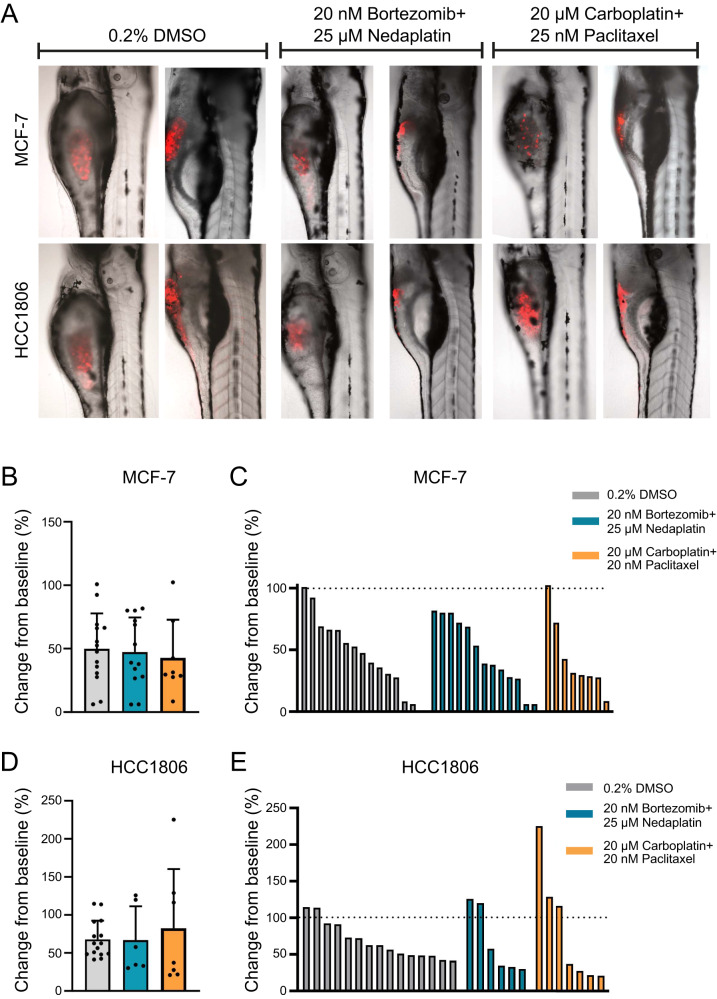
Drug treatment of xenografted zebrafish larvae. **A** Zebrafish larvae xenotransplanted with DiI-labeled (red) MCF-7 or HCC1806 human breast cancer cells were treated from 1–3 days post injection (dpi) with 0.20% DMSO, or a combination of 20 nM bortezomib and 25 µM nedaplatin or 20 µM carboplatin and 20 nM paclitaxel. Dot plots show relative change in tumor volume from baseline in MCF-7 treated with 0.20% DMSO (*n* = 14), 20 nM bortezomib and 25 µM nedaplatin (*n* = 13) or 20 µM carboplatin and 20 nM paclitaxel (*n* = 8) plotted as **B** mean with standard deviation **C** or individually. Dot plots show the relative change in tumor volume from baseline in HCC1806 treated with 0.20% DMSO (*n* = 15), 20 nM bortezomib and 25 µM nedaplatin (*n* = 6) or 20 µM carboplatin and 20 nM paclitaxel (*n* = 7) plotted as **D** mean with standard deviation **E** or as individual larvae. The dotted line indicates relative volume at 1 dpi.

To further validate our results, we evaluated the effect of combination treatment with bortezomib+nedaplatin (promising combination treatment) and carboplatin+paclitaxel (clinical relevant combination treatment) using a zebrafish xenograft in vivo model (Fig. 6). To this end, zebrafish larvae with DiI-labeled MCF-7 or HCC1806 cells transplanted into the yolk were treated for 48 h with a combination of either 20 nM bortezomib and 25 µM nedaplatin or 20 µM carboplatin and 20 nM paclitaxel in 0.20% DMSO. Control larvae were exposed to 0.20% DMSO. Tumor growth was evaluated as the change in tumor cell volume from the start of treatment (1 day post-injection [dpi]) to the end of drug treatment (3 dpi; Fig. 6A). The drugs were well tolerated by the zebrafish larvae and did not cause an increased death rate at the chosen concentrations. However, the invasive growth of the HCC1806 tumor cells resulted in metastasis, mainly located to the trunk of the zebrafish larvae. Furthermore, the invasive growth of the HCC1806 cells occasionally disrupted the yolk sac. Larvae in which this happened were not included in the tumor growth analysis as the exact tumor size became hard to measure. There was no significant change in the mean tumor volume between the treatments or cell lines (Fig. 6B–E). However, more MCF-7 xenografts treated with carboplatin+paclitaxel (75%) had a tumor volume <50% at 3 dpi than either DMSO (50%) or bortezomib+nedaplatin (50%). The same trend was observed with HCC1806-derived xenografts where 33% of larvae had an end tumor volume <50% after treatment with DMSO, 50% for bortezomib+nedaplatin, and 57% for carboplatin+paclitaxel.

## Discussion

In this study, we evaluate the intrinsic chemosensitivity of TNBC cells to clinically relevant drugs and PIs in both mono- and combination therapy [28, 29]. Hereby, the degree of chemosensitivity in monotherapy could not be directly linked to specific TNBC subtypes due to the variation in response within each subtype. LAR was the only subtype where both investigated cell lines showed similar sensitivity to the tested drugs, indicating underlying causes that need to be investigated further. However, we identified highly potent single-drug treatments (e.g., bortezomib, epoxomicin, nedaplatin) and 2-drug combinations (e.g., bortezomib+nedaplatin and epoxomicin+epirubicin) for all TNBC subtypes with almost 100% inhibition that have potential to ultimately improve TNBC treatment.

For monotherapy, we used several metrics (GR_50_, GR_max_, IC_50_ [half maximal inhibitory concentration [27]] and AUC) to evaluate drug potency/efficacy and cell line chemosensitivity. Metrics like GR_50_ and IC_50_ show potency at an endpoint, while GR_max_ and AUC summarize drug efficacy from low to high doses [26, 30, 31]. Using only one drug response metric (e.g., GR_50_ or AUC) should be avoided because it will not give full information about drug potency at the concentrations tested. In this analysis, we were able to identify six potent drugs (bortezomib, cisplatin, delanzomib, docetaxel, epoxomicin, and nedaplatin) for all cell lines, representing three different drug classes (proteasome inhibitors, platinum agents, and mitotic inhibitors). Topoisomerase inhibitors had a weak impact on cell viability in the tested cell lines, which could possibly be due to drug resistance [32, 33]. However, cell lines that were least sensitive to all drugs were CAL-148, HCC1806, and MDA-MB-453, whereas HCC38, MDA-MB-436, and MDA-MB-468 were most sensitive, indicating variability in chemosensitivity within each TNBC subtype. Interestingly, drug sensitivity could not be assigned to a subtype because sensitivity was heterogeneous within each subtype except for the LAR subtype (CAL-148 and MDA-MB-453 cell lines). Both LAR cell lines were similarly least sensitive to the tested drugs according to GR_max_. These findings are in line with previous studies showing that the LAR subtype has the lowest response rates to chemotherapy and high risk of relapse following radiation therapy [6, 9].

Furthermore, AR is an important marker that has previously been correlated with e.g., tumor growth, proliferation, and invasion [34, 35]. In the present study, BT-549, CAL-148, HCC70, and MDA-MB-453 were positive for AR which is in line with previous studies [36, 37]. Although we could establish an association between AR expression and sensitivity to drug treatment, its role in the intrinsic chemosensitivity of TNBC is not fully understood and warrants further investigation [34, 35]. Speers et al. also recently showed that AR-positive TNBC cells increase their AR expression during radiation therapy, which in turn counteracts the effect of treatment possibly due to defects in DNA repair [38]. De Amicis et al. also showed that breast cancer cells with high AR expression became less sensitive to tamoxifen treatment [39]. Our findings show that AR-expressing cells were the least sensitive to monotherapy, but were more sensitive when treated with 2-drug combinations.

Increased proteasome activity has been associated with cancer, where it controls multiple biological processes within the cell [40, 41]. PIs have been proven to inhibit these processes. Here, we investigate whether the tested PIs inhibit the chymotrypsin-like (β5 catalytic site) activity of the 20 S proteasome in MCF-7 cells. At the highest dose (10000 nM), this analysis revealed that bortezomib and delanzomib showed 100% inhibition of β5 activity, while treatment with MLN-2238 and MLN-9708 lead to only 33% and 51% inhibition, respectively. The similar inhibition patterns shown by bortezomib and delanzomib are not surprising since both drugs have previously been correlated with similar proteasome inhibitory activity [42]. Nevertheless, it was surprising that some of the other PIs showed weak inhibition of β5 activity, especially potent drugs such as epoxomicin (20% inhibition). This could, however, be due to weak inhibition of proteases with chymotrypsin-like activity, which may also be the case for the other weak PIs [43].

In the combination therapy analysis, we evaluate 11 potentially potent 2-drug combinations as well as 2 drug combinations currently used to treat patients with TNBC. Ideally, drug combinations are used to prevent drug resistance, achieve synergy, and/or increase response above the strongest single drug [44]. Firstly, we evaluated response using mean and median inhibition values across all tested doses. Consequently, we were able to identify three promising combinations (bortezomib+nedaplatin, bortezomib+doxorubicin, and epoxomicin+epirubicin) in the four tested TNBC cell lines. Surprisingly, topoisomerase inhibitors were weak compounds in monotherapy, but we observed an enhanced effect in combination with e.g., bortezomib above the strongest single drug. Secondly, we identify the highest percentage inhibition that each drug combination could achieve. The three drug combinations achieved almost 100% inhibition by bortezomib+nedaplatin in the four TNBC cell lines (<70% in control MCF-7 cells), between 68% and 94% for epoxomicin+epirubicin (~70% in MCF-7 cells), and between 64% and 94% for bortezomib+doxorubicin (~64% in MCF-7 cells) after 24 h treatment. Notably, bortezomib had above 94% inhibition alone in HCC38 cells (96% inhibition in combination), so doxorubicin did not enhance the effect of bortezomib in the HCC38 cell line. Consequently, it is difficult to increase drug response above the highest single drug when that drug is very potent. Thirdly, Bliss Synergy scores between drugs were evaluated using SynergyFinder, thereby showing that carboplatin+docetaxel had high synergy scores (range 18.1-37.8), but with unexpectedly low inhibition scores (mean range 15.6-33.0%, median range 20.8-41.2%). Of the three combinations with the highest response scores, bortezomib+nedaplatin and epoxomicin+epirubicin had synergy scores above 10 which is considered to be high, though the controls (BT-474, MCF-7, and T47D) and HCC1806 were least sensitive to bortezomib+nedaplatin.

Zebrafish xenograft models have become attractive in vivo animal models for cancer research given the relatively low cost of high-throughput studies, high homology with human genes (~80%), high fertility levels and fast development, quick tumor development, and transparency of the fish [45–47]. In the present study, we show that tumor-bearing zebrafish larvae treated with bortezomib+nedaplatin and carboplatin+paclitaxel experience tumor regression within 48 h, corroborating the cell line-derived results. TNBC HCC1806 xenografts also frequently resulted in metastasis despite treatment with chemotherapeutic agents, while ER+ MCF-7 xenografts did not. These findings highlight the metastatic potential of TNBC cells in vivo. Recent studies have demonstrated the invasive properties of tumor xenografts, as well as the presentation of micrometastases in zebrafish larvae [48, 49]. Nevertheless, no differences in the mean tumor volume were found between either drug combination or xenograft model. This could possibly be explained by: (1) HCC1806 and MCF-7 were less sensitive to bortezomib+nedaplatin, (2) the use of suboptimal drug doses to avoid toxic effects in the larvae, and (3) the use of fixed drug doses rather than a range of doses. However, our results are promising and warrant further evaluation in this animal model.

Drug combinations containing DNA damage inducers (epirubicin and nedaplatin) and proteasome inhibitors (bortezomib and epoxomicin) will inhibit the DNA repair system, and were shown to work well together to kill TNBC cells [50–52]. This approach has also been proposed as a strategy for overcoming treatment resistance to PIs. In other words, PIs will ultimately lead to an accumulation of proteins, while drug #2 can be used to prevent the cell from overcoming the effect of the PI, e.g., by blocking lysosomal degradation [53]. PIs have recently been found to induce immunogenic cell death by activating the cGAS/STING-pathway, which could be used as a target for immunotherapy in combination with PIs to increase treatment efficacy and as a means of overcoming resistance to PIs [54]. However, future studies are warranted to evaluate this molecular mechanism in connection with PIs in more detail [54].

Our drug screening experiments were performed with high precision and according to our recent paper using this approach, in which we optimize the performance of the in vitro experiments to obtain replicable and reproducible results [31]. By only testing two cell lines per TNBC subtype, we were not able to pinpoint specific treatments that are most effective for each TNBC subtype. Thus, further studies are warranted to assess the suitability of the identified combination therapies using a larger number of TNBC cell lines and examine the transcriptomic and genomic profiles of cell lines and patients with TNBC to identify therapeutic biomarkers associated with therapy response [17]. In combination therapy, we believe that it is not the drug doses themselves that contribute to synergy, but rather the ratio between the drugs. Therefore, drug doses can be adjusted up and down if the ratio of drug #1 and drug #2 is maintained, but this needs to be studied in more detail.

Taken together, our in vitro drug screens identified highly potent single drugs (e.g., bortezomib, delanzomib, epoxomicin, and nedaplatin) and 2-drug combinations (bortezomib+nedaplatin for CAL-148 [97.1% inhibition], HCC1806 [85.1% inhibition], and MDA-MB-468 [91.1% inhibition]; epoxomicin+epirubicin for HCC38 [94.4% inhibition]) showing almost 100% inhibition in individual TNBC cell lines. Surprisingly, LAR was the least sensitive TNBC subtype during monotherapy but the most sensitive when treated with bortezomib+nedaplatin. Otherwise, there was no connection between TNBC subtype and chemosensitivity to combination treatment. Although the proteasome activity was not always fully inhibited, we were able to identify several potent proteasome inhibitors (e.g., bortezomib and epoxomicin) that were very potent together with other drug classes (e.g., the platinum agent nedaplatin). Our findings highlight the importance of a more in-depth understanding of the underlying mechanisms for variability in therapy response for certain TNBC subtypes. Ultimately, this could lead to enhanced/improved treatment approaches for patients with TNBC. However, these promising drug combinations need to be evaluated further in TNBC patient samples (e.g., patient-derived organoids and xenografts).

## Materials and methods

The Supplementary Materials and Methods describes the experimental procedures in more detail.

### Cell culture

Eight human TNBC cell lines (BT-549, CAL-148, HCC38, HCC70, HCC1806, MDA-MB-436, MDA-MB-453, and MDA-MB-468) and four control cell lines (BT-474, MCF-7, MCF-10A, and T47D) were purchased from American Type Culture Collection (ATCC) or German Collection of Microorganisms and Cell Cultures GmbH (DSMZ), and authenticated using the Eurofins Genomics Human Cell Line Authentication Service. Breast cancer subtyping and culture medium composition is outlined in Supplementary Table 1. The cells were cultured at 37 °C in a humidified 5% CO_2_ environment.

### Western blot

Denatured protein lysates (50 µg) were separated using SDS-PAGE electrophoresis and transferred to nitrocellulose membranes. The membranes were probed with primary antibodies for androgen receptor or beta-actin (loading control), followed by visualization of protein bands using a chemiluminescence kit and quantification using ImageJ/FIJI software (version 1.53t) [55].

### Pharmaceutical compounds

**Fig. 7:**
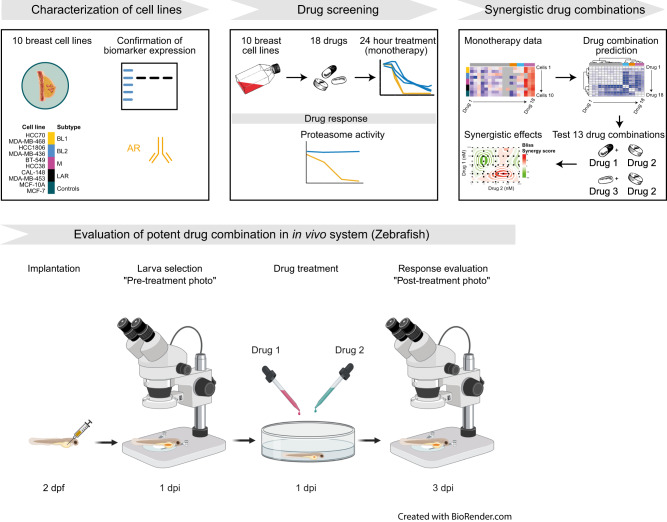
Study design and workflow. Characterization of cell lines was performed using key marker for breast cancer (androgen receptor [AR]). Drug screening was performed using 18 drugs on 10 cell lines, and proteasome activity was measured for proteasome inhibitors to measure their ability to suppress the β5 site on the 20 S proteasome. The synergistic effect and inhibitory potency of 13 drug combinations were tested on five cell lines (CAL-148, HCC38, HCC1806, MCF-7 and MDA-MB-468). The data obtained in monotherapy were used to predict potent drug combinations by IDACombo. The predicted combinations plus combinations used in clinical practice were evaluated. This figure was designed using assets from Freepik.com (https://www.freepik.com/free-vector/set-female-breast-augmentation_2413429.htm#query=breast%20anatomy&position=5&from_view=search&track=sph).

Eighteen pharmaceutical compounds were purchased from Selleckchem (Supplementary Table 2). Stock solutions were prepared using dimethyl sulfoxide (DMSO; Merck), physiological saline (sodium chloride 0.9%) or Milli-Q water, and stored at −80 °C. Stock solutions were diluted with 1xPBS to nine working concentrations (3–768 µM cisplatin; 2-1024 µM carboplatin and nedaplatin; 1-10000 nM mitosis-, proteasome-, and topoisomerase inhibitors) for monotherapy or four working concentrations (8–512 µM carboplatin and nedaplatin; 10–5000 nM mitosis-, proteasome-, and topoisomerase inhibitors) for combination therapy. Matched concentration solvent vehicle controls were used for each drug concentration. The drug screens were performed in triplicate and repeated three (monotherapy) or two (combination therapy) times (Fig. 7).

### Resazurin-based cell viability assay

The optimal seeding density per 96-well was determined for each cell line as previously described [31]. The cells were seeded at a density of 4.0 × 10^3^ to 7.5 × 10^3^ (cell type dependent) in 96-well clear, flat-bottom microplates (Corning Life Sciences, Sigma-Aldrich) in 100 µl complete culture medium (Supplementary Table 1) and cultured for 24 h. Cell viability was determined using the resazurin assay in untreated cells at the time of plating (*t* = 0) and treated cells after 24 h drug treatment (Fig. 7). Percentage cell viability was calculated as 100% × (absorbance of treated cells – absorbance of background controls) / (absorbance of matched solvent vehicle controls – absorbance of background controls). The area under the curve (AUC), half-maximal inhibitory concentration (IC_50_), drug potency (GR_50_), and drug efficacy (GR_max_) were determined for each compound using the GRmetrics (version 1.20.0) package [56] in R. The monotherapy data were evaluated with the IDACombo (version 1.0.2) package in R to predict potential drug combinations (Table 1). SynergyFinder (version 3.2.10) [57] package in R was used to identify synergistic drug combinations. A synergy score ≥10 was considered a strong synergistic effect between compounds.

### Proteasome activity

Proteasome activity of the β5 catalytic site was determined for the PIs using a Suc-Leu-Leu-Val-Tyr-AMC fluorescent proteasome substrate (Enzo Life Sciences, Cat. BML-P802) at a final concentration of 20 µM. MCF-7 cells were seeded at 7.5 × 10^3^ cells/well in black 96-well flat-bottom microplates (Nuclon™ Delta Surface, ThermoFisher Scientific) with 100 µl culture medium supplemented with 10% FBS. After 24 h, the cells were treated for 6 h with the 11 PIs at 1, 10, 100, and 1000 nM and matched untreated controls. The fluorescence intensity was measured after 2 h at 380 nm (excitation filter) and 460 nm (emission filter) in a Wallac 1420 VICTOR2™ microplate reader (Perkin Elmer; Fig. 7).

### Xenotransplantation

Zebrafish larvae were positioned in an 1% agarose injection mold and between 500 and 1000 CM-DiI-labeled HCC1806 and MCF-7 cells were injected into the yolk sac of each zebrafish larva using a FemtoJet express microinjector (Eppendorf, Hamburg, Germany) and glass microinjection needles without filaments (World Precision Instruments). Larvae were incubated at 28.5 °C for 1 h post implantation and then incubated at 34 °C until the end of the experiment. Tumor xenografts were evaluated at 1 dpi. Larvae with DiI fluorescence at the injection site were selected for drug treatment. Selected embryos were transferred to a 96-well plate (Corning) and incubated in freshly prepared embryo medium (EM; 1.0 mM MgSO_4_, 0.15 mM KH_2_PO_4_, 0.042 mM Na_2_HPO_4_, 1 mM CaCl_2_, 0.5 mM KCl, 15 mM NaCl, 0.7 mM NaHCO_3_) containing drugs or 0.2% DMSO at 34 °C until 3 dpi. Drug combinations used were 20 nM bortezomib with 25 µM nedaplatin or 20 µM carboplatin with 20 nM paclitaxel in EM with a total DMSO concentration of 0.2%. Tumor growth was evaluated by confocal microscopy with an inverted Nikon A1 confocal system (Nikon Instruments, Melville, NY, USA) using a 10x objective before drug exposure (1 dpi) and at the end of drug treatment (3 dpi). The acquired stacks were analyzed and produced using ImageJ software (National Institute of Health, USA).

### Statistical analysis

Statistical analyses were performed using a *p*-value cutoff of 0.05 (two-tailed) in R/Bioconductor (version 3.14.0) or GraphPad Prism 9 software (Prism®, San Diego, CA, USA). IC_50_, AUC, GR_50_, and GR_max_ values were expressed as mean and standard deviation. Weighted scatterplots and dotplots were constructed using the ggplot2 package (version 3.3.6) [58] in R. A heatmap for IDACombo-derived data was constructed using pheatmap package (version 1.0.12) and Manhattan distance metric and clustered by Ward.D2 [59]. Linear regression analysis was performed using the ggplot2 package to determine the correlation between GR_50_ values and quantified protein bands for AR. Bar charts were constructed using the ggpubr (version 0.5.0 [60]) and rstatix (version 0.7.1) R packages with pairwise t-test and Bonferroni adjusted *p*-values (*P < 0.05; **P ≤ 0.01; ***P ≤ 0.001; ****P ≤ 0.0001). Non-significant differences were only shown in the highest drug concentration evaluation.

### Supplementary information

Original Data File

Supplementary Materials

Supplementary Figure 1

Supplementary Figure 2

Supplementary Figure 3

## Data Availability

All data used in this study are included or referred to within this work.
