# Supplementary material for: Repurposing proteasome inhibitors for improved treatment of triple-negative breast cancer
Source: Cell Death Discov. 2024 Jan 29;10:57. doi: 10.1038/s41420-024-01819-5 (PMC10825133; doi:10.1038/s41420-024-01819-5)
Supplement: Supplementary file 1 — Original Data File [file 41420_2024_1819_MOESM1_ESM.docx]

**Supplementary Materials for**

**Repurposing proteasome inhibitors for improved treatment of triple-negative breast cancer**

**This file includes:**
Western blots

**Western blot images**


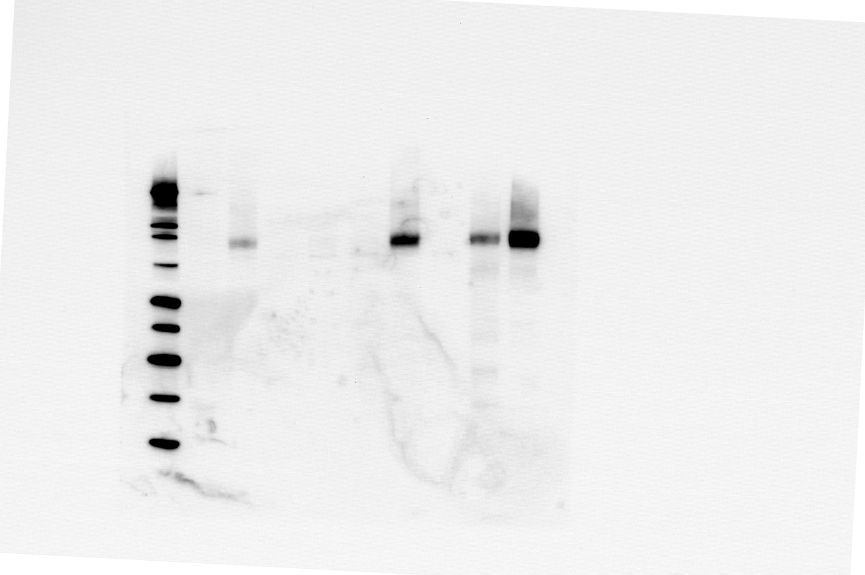


Rabbit anti-androgen receptor (AR, 1:250; Abcam, Cat. ab133273)


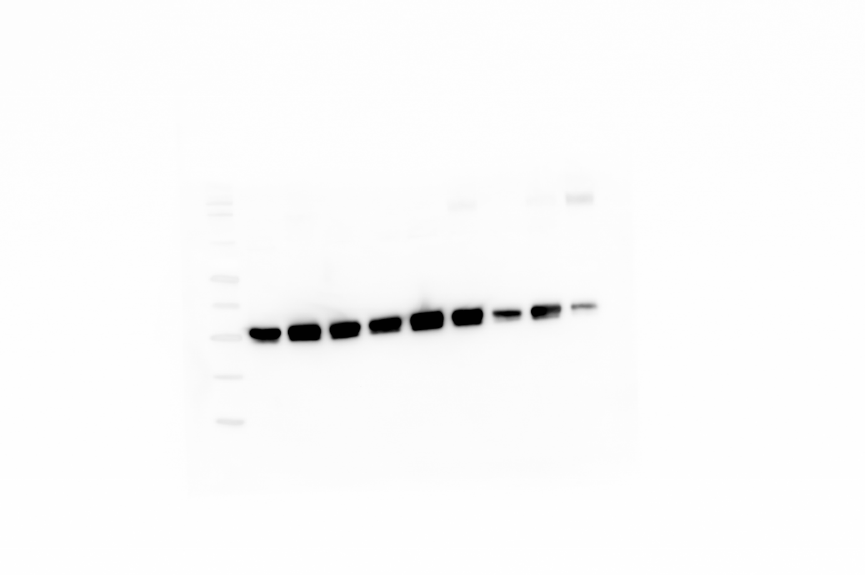


Mouse anti-Beta-actin (1:2000; Abcam, Cat. ab6276)
