# Supplementary material for: Repurposing proteasome inhibitors for improved treatment of triple-negative breast cancer
Source: Cell Death Discov. 2024 Jan 29;10:57. doi: 10.1038/s41420-024-01819-5 (PMC10825133; doi:10.1038/s41420-024-01819-5)
Supplement: Supplementary file 2 — Supplementary Materials [file 41420_2024_1819_MOESM2_ESM.docx]

**Supplementary Materials for**

**Repurposing proteasome inhibitors for improved treatment of triple-negative breast cancer**

**This file includes:**
Supplementary Materials and Methods

Supplementary Figure Legends

Supplementary Tables 1-2

Western blots

**Supplementary Materials and Methods**

**Western blot**

Cells were lysed in Qproteome Mammalian Lysis Buffer (Qiagen) supplemented with Benzonase® Nuclease, as well as protease and phosphatase inhibitors. Protein concentration was determined using Bradford Protein Assay Dye Reagent Concentrate (BioRad). The protein lysates (50 µg) were separated on NuPAGE® Bis-Tris Gel 1.0 mm 4-12% polyacrylamide gels (ThermoFisher Scientific) and transferred to nitrocellulose membranes. The membranes were incubated overnight in 5% non-fat dry milk (NFDM; Semper) solution at 4°C, followed by incubation with primary antibodies for rabbit anti-androgen receptor (AR, 1:250; Abcam, Cat. ab133273) and mouse anti-beta-actin (1:2000; Abcam, Cat. ab6276) at room temperature (RT) for 2h. The membranes were then incubated with secondary horseradish peroxidase-linked anti-mouse (1:2000; Amersham, Cat. NA931V) or anti-rabbit (1:2000; Amersham, Cat. NA934V) IgG antibodies at RT for 1h. Proteins were detected using the SuperSignal™ West Femto Maximum Sensitivity Substrate (ThermoFisher Scientific). Quantification of the region of interest (ROI) for AR was performed using ImageJ/FIJI software (version 1.53t) [[17](#_ENREF_17)].

**Pharmaceutical compounds**

Eighteen pharmaceutical compounds were purchased from Selleckchem. Stock solutions of platinum-based agents were prepared using physiological saline (sodium chloride 0.9%) for cisplatin (7.68 mM) and MilliQ water for carboplatin (50 mM) and nedaplatin (25 mM). The solubility of the compound was enhanced by pre-heating the solvent (lukewarm), if necessary. Dimethyl sulfoxide (DMSO; Merck) was used to prepare stock solutions for proteasome inhibitors (1 mM; bortezomib, carfilzomib, celastrol, delanzomib, epoxomicin, MG-132, MLN2238, MLN9708, oprozomib, PI-1840, and VR23), mitosis inhibitors (1 mM; docetaxel and paclitaxel), and topoisomerase inhibitors (1 mM; doxorubicin and epirubicin). The stock solutions were stored at -80°C. For monotherapy, a 2-, 5- and/or 10-fold dilution series containing nine working concentrations (3-768 µM cisplatin; 2-1024 µM carboplatin and nedaplatin; 1-10000 nM mitosis-, proteasome-, and topoisomerase inhibitors) were prepared by diluting the stock solutions with 1xPBS. Combination treatments were performed using 8- and 4-fold dilution series containing four working concentrations (8-512 µM carboplatin and nedaplatin; 10-5000 nM mitosis-, proteasome-, and topoisomerase inhibitors) prepared by diluting the stock solutions in 1xPBS. Matched concentration solvent vehicle controls were used for each drug concentration. The drug screens were performed in triplicate and repeated three times for the monotherapies and twice for the combination treatments. The drug(s) and corresponding controls were plated in 96-well PCR plates (VWR) and stored at -20°C (**Fig. 6**). The drug plates were thawed, centrifuged (3000 rpm for 30s), and acclimatized to RT before the drugs were added to the cells. New drug plates were prepared for each drug screen.

­­­­

**Resazurin-based cell viability assay**

To determine the optimal seeding density and ensure that the cells were in the exponential phase during the duration of the experiment (approx. 52h), each cell line (BT-549, CAL-148, HCC1806, HCC38, HCC70, MCF-10A, MCF-7, MDA-MB-436, MDA-MB-453, and MDA-MB-468) was seeded at different concentrations (1000 to 16000 cells) per 96-well, as previously described [[18](#_ENREF_18)]. Cell viability was measured daily (day 0 [plating day] to day 4) using 0.2 mg/ml resazurin solution prepared from resazurin sodium salt (Fisher Scientific) dissolved in sterile 1xPBS (ThermoFisher Scientific). Before use, the resazurin solution was brought to RT before 10 µl (10% of cell medium volume) was added to each 96-well and incubated for 4h at 37°C. The absorbance was measured with a 560 nm excitation filter and a 615 nm emission filter in a Wallac 1420 VICTOR2™ microplate reader (Perkin Elmer). The slope of the curve was used to determine the optimal seeding density, i.e., the highest seeding density with linear growth between days 1 and 3.

The cells were seeded at a density of 4.0x10^3^ to 7.5x10^3^ (cell type-dependent) in 96-well clear, flat-bottom microplates (Corning Life Sciences, Sigma-Aldrich) in 100 µl culture medium supplemented with 10% FBS and cultured for 24h. Cell viability was determined using the resazurin assay after 24h drug treatment (**Fig. 6**). Percentage cell viability was calculated as 100% × (absorbance of treated cells – absorbance of background controls) / (absorbance of matched solvent vehicle controls – absorbance of background controls). Normalized growth rate inhibition (GR) was assessed using the absorbance of untreated cells after the time of plating (t = 0). The area under the curve (AUC), half-maximal inhibitory concentration (IC50), drug potency (GR50), and drug efficacy (GRmax <0 = cytotoxic compound, 0 = fully cytostatic compound, and >0 = partial growth inhibition) were determined for each compound using the GRmetrics (version 1.20.0) package [[19](#_ENREF_19)] in R. IC50 and GR50 values were removed for cases where values were (a) not reached (INF) or (b) higher than the tested dose range before mean IC50 and GR50 values for multiple experiments were calculated. For combination treatment, the monotherapy data were evaluated with the IDACombo (version 1.0.2)[[20](#_ENREF_20)] package in R to predict potential drug combinations (**Table 1**). SynergyFinder (version 3.2.10)[[21](#_ENREF_21)] package in R was used to identify synergistic drug combinations. A synergy score ≥10 was considered a strong synergistic effect between compounds.

**Cell preparation and xenotransplantation**

For xenotransplantation, HCC1806 and MCF-7 cells were washed twice with PBS and incubated with 5 µl/ml CellTracker™ CM-DiI Dye (ThermoFisher Scientific) in PBS for 30 min at 37°C. The CM-DiI-labeled cells were then washed with PBS, trypsinized, and centrifuged at 220xg for 5 min. Single cell suspensions were re-suspended at 0.5x10^6^ cells/µl in 2% PVP/PBS and stored on ice until implantation. MCF-7 cells were passed through a 40 μm cell strainer prior to resuspension in 2% PVP/PBS.

Zebrafish larvae were raised at 28.5°C until 2 days post fertilization (dpf) in embryo medium (EM; 1.0 mM MgSO_4_, 0.15 mM KH_2_PO_4_, 0.042 mM Na_2_HPO_4_, 1 mM CaCl_2_, 0.5 mM KCl, 15mM NaCl, 0.7 mM NaHCO_3_). Before implantation, zebrafish larvae were anesthetized in 0.02% MS-222 (Sigma-Aldrich, E10521) in EM. Zebrafish larvae were positioned in an 1% agarose injection mold and between 500 and 1000 CM-DiI-labeled tumor cells were injected into the yolk sac of each zebrafish larva using a FemtoJet express microinjector (Eppendorf, Hamburg, Germany) and glass microinjection needles without filaments (World Precision Instruments). Larvae were incubated at 28.5°C for 1 hour post implantation and then incubated at 34°C incubator until the end of the experiment.

Tumor xenografts were evaluated at 1 dpi. Larvae with DiI fluorescence at the injection site were selected for drug treatment and randomly into the different treatment groups. Selected embryos were transferred to a 96-well plate (Corning) and incubated in freshly prepared EM containing drugs or 0.2% DMSO at 34°C until 3 dpi. Drug combinations used were 20 nM bortezomib with 25 µM nedaplatin or 20 µM carboplatin with 20 nM paclitaxel in EM with a total DMSO concentration of 0.2%. Tumor growth was evaluated by confocal microscopy before drug exposure (1 dpi). Immediately preceding imaging, larvae were anesthetized in 0.02% MS-222 and embedded laterally in 1% low temperature gelling agarose (Sigma-Aldrich, A4018) in glass bottom dishes (D29-10-1.5-N, Cellvis). At the end of drug treatment (3 dpi), all zebrafish larvae were euthanized in 0.04% MS-222 and incubated overnight in 4% paraformaldehyde solution (Sigma-Aldrich, St Louis, MO), washed in phosphate-buffered saline (PBS) and then embedded laterally in 1% low temperature gelling agarose (Sigma-Aldrich, A4018) in glass bottom dishes (D29-10-1.5-N, Cellvis). Zebrafish in which implanted cells extruded from the yolk sac were not included due to uncertainties regarding tumor size. Images were obtained with an inverted Nikon A1 confocal system (Nikon Instruments, Melville, NY, USA) using a 10x objective. The investigators were blinded to the treatment groups during analysis of the acquired stacks using ImageJ software (National Institute of Health, USA). Graphs and statistical analyses were generated using GraphPad Prism 9 software (Prism®, San Diego, CA, USA).

**Supplementary Figure legends**

**Supplementary Figure 1**

Nine cell lines were evaluated for androgen receptor (AR) expression. A) displays the loading control (Beta-Actin). (B) Four cell lines (BT-549, CAL-148, HCC70, and MDA-MB-453) were AR positive.

**Supplementary Figure 2**

Five cell lines were exposed to 13 drug combinations. Drug response matrix was used to visualize the sensitivity of four TNBC cell lines (CAL-148, HCC1806, HCC38, and MDA-MB-468) and one breast cancer cell line (MCF-7) for 13 drug combinations. The sensitivity was determined by mean, median, and direct percentages of response at various concentrations.

**Supplementary Figure 3**

Bliss synergy score was used to identify synergistic effect occurrence between 13 drug combinations on five cell lines (CAL-148, HCC1806, HCC38, MCF-7, and MDA-MB-468). A synergy score ≥10 was considered as strong synergistic effect and ≤-10 were considered as strong antagonistic effect between compounds.

| **Supplementary Table 1. Cell lines included in the study derived from breast cancer and normal breast tissue** | | | | |
| --- | --- | --- | --- | --- |
|  |  |  |  |  |
|  | **Cell line** | **Breast cancer subtype** | **TNBC subtype** | **Culture medium** |
| 1 | HCC70 | TNBC | BL1 | RPMI + 10% FBS |
| 2 | MDA-MB-468 | TNBC | BL1 | RPMI + 10% FBS + 1% SP |
| 3 | HCC1806 | TNBC | BL2 | RPMI + 10% FBS |
| 4 | MDA-MB-436 | TNBC | BL2 | DMEM + 10% FBS |
| 5 | CAL-148 | TNBC | LAR | DMEM + 10% FBS |
| 6 | MDA-MB-453 | TNBC | LAR | DMEM + 10% FBS |
| 7 | BT-549 | TNBC | M | RPMI + 10% FBS |
| 8 | HCC38 | TNBC | M | RPMI + 10% FBS |
| 9 | MCF-7 | Luminal A | - | DMEM + 10% FBS |
| 10 | MCF-10A | Non-cancer | - | RPMI + 10% FBS + 0.5 mg/ml HC + 20 ng/mL EGF + 100 ng/ml CT + 10 µg/mL insulin |
| 11 | BT-474 | Luminal B | - | DMEM + 10% FBS |
| 12 | T47D | Luminal A | - | DMEM + 10% FBS |
| All culture medium (Dulbecco Modified Eagle´s Medium [DMEM] and RPMI 1640 medium) was purchased from ThermoFisher Scientific and supplemented with 2 mM L-glutamine and 2 g/L D-glucose.  BL1, basal-like 1; BL2, basal-like 2; CT, cholera toxin; EGF, epidermal growth factor; FBS, fetal bovine serum; HC, hydrocortisone; LAR, luminal androgen receptor; M, mesenchymal; NEAA, non-essential amino acids; SP, sodium pyruvate; TNBC, triple-negative; UNS, unspecified | | | | |

| **Supplementary Table 2. Pharmaceutical compounds** | | | | |
| --- | --- | --- | --- | --- |
|  | | | | |
| **Drug** | **Solvent** | **Working concentration** | **Supplier** | **Catalog no.** |
| Proteasome inhibitors | | | |  |
| Bortezomib | DMSO | 1-10000 nM | Selleckchem | S1013 |
| Carfilzomb | DMSO | 1-10000 nM | Selleckchem | S2853 |
| Celastrol | DMSO | 1-10000 nM | Selleckchem | S1290 |
| Delanzomib | DMSO | 1-10000 nM | Selleckchem | S1157 |
| Epoxomicin | DMSO | 1-10000 nM | Selleckchem | S7038 |
| MG-132 | DMSO | 1-10000 nM | Selleckchem | S2619 |
| MLN-2238 | DMSO | 1-10000 nM | Selleckchem | S2180 |
| MLN-9708 | DMSO | 1-10000 nM | Selleckchem | S2181 |
| Oprozomib | DMSO | 1-10000 nM | Selleckchem | S7049 |
| PI-1840 | DMSO | 1-10000 nM | Selleckchem | S7462 |
| VR-23 | DMSO | 1-10000 nM | Selleckchem | S7933 |
| Mitosis inhibitors |  |  |  |  |
| Docetaxel | DMSO | 1-10000 nM | Selleckchem | S1148 |
| Paclitaxel | DMSO | 1-10000 nM | Selleckchem | S1150 |
| Topoisomerase inhibitors | | | |  |
| Doxorubicin | DMSO | 1-10000 nM | Selleckchem | S1208 |
| Epirubicin | DMSO | 1-10000 nM | Selleckchem | S1223 |
| Platinum agents | | | |  |
| Carboplatin | Milli-Q water | 2-1024 µM | Selleckchem | S1215 |
| Cisplatin | 0.9% NaCl | 3-768 µM | Selleckchem | S1166 |
| Nedaplatin | Milli-Q water | 2-1024 µM | Selleckchem | S1826 |

The working concentration was prepared using 1x phosphate buffered saline. DMSO, dimethyl sulfoxide; NaCl, sodium chloride

**Western blot images**


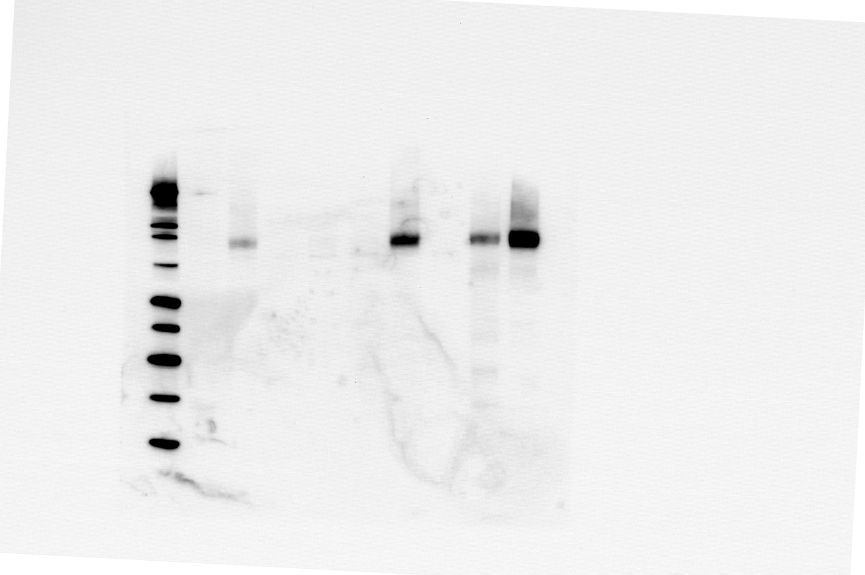


Rabbit anti-androgen receptor (AR, 1:250; Abcam, Cat. ab133273)


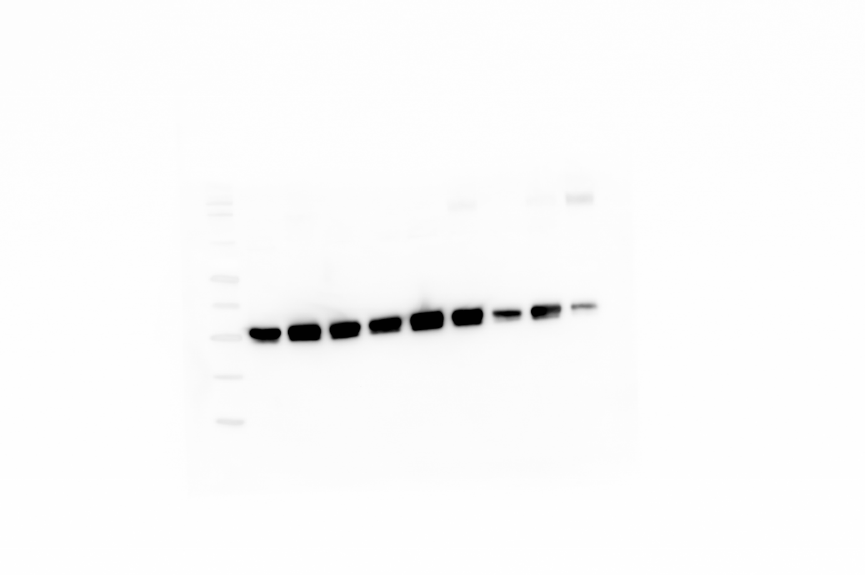


Mouse anti-Beta-actin (1:2000; Abcam, Cat. ab6276)
