# Supplementary figures and images for: Repurposing proteasome inhibitors for improved treatment of triple-negative breast cancer
Source: Cell Death Discov. 2024 Jan 29;10:57. doi: 10.1038/s41420-024-01819-5 (PMC10825133; doi:10.1038/s41420-024-01819-5)

A

## Beta-actin

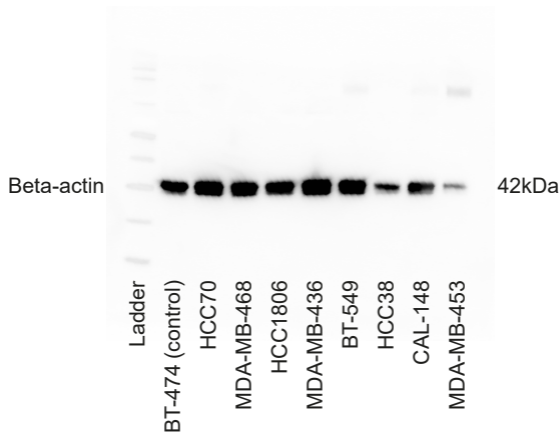

B

## Androgen receptor (AR)

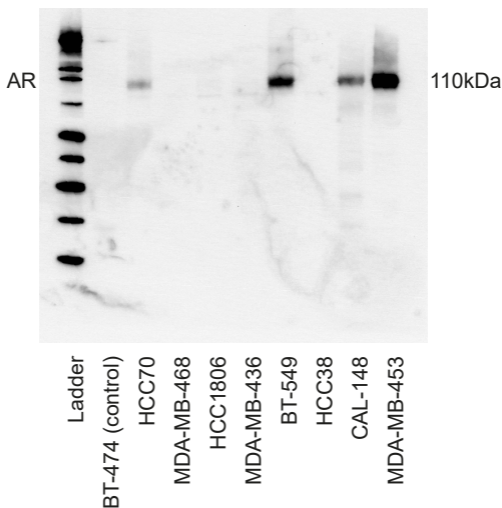

Supplement: Supplementary file 3 — Supplementary Figure 1 [file 41420_2024_1819_MOESM3_ESM.pdf]
