# Supplementary material for: Repurposing proteasome inhibitors for improved treatment of triple-negative breast cancer
Source: Cell Death Discov. 2024 Jan 29;10:57. doi: 10.1038/s41420-024-01819-5 (PMC10825133; doi:10.1038/s41420-024-01819-5)
Supplement: Supplementary file 4 — Supplementary Figure 2 [file 41420_2024_1819_MOESM4_ESM.pdf]

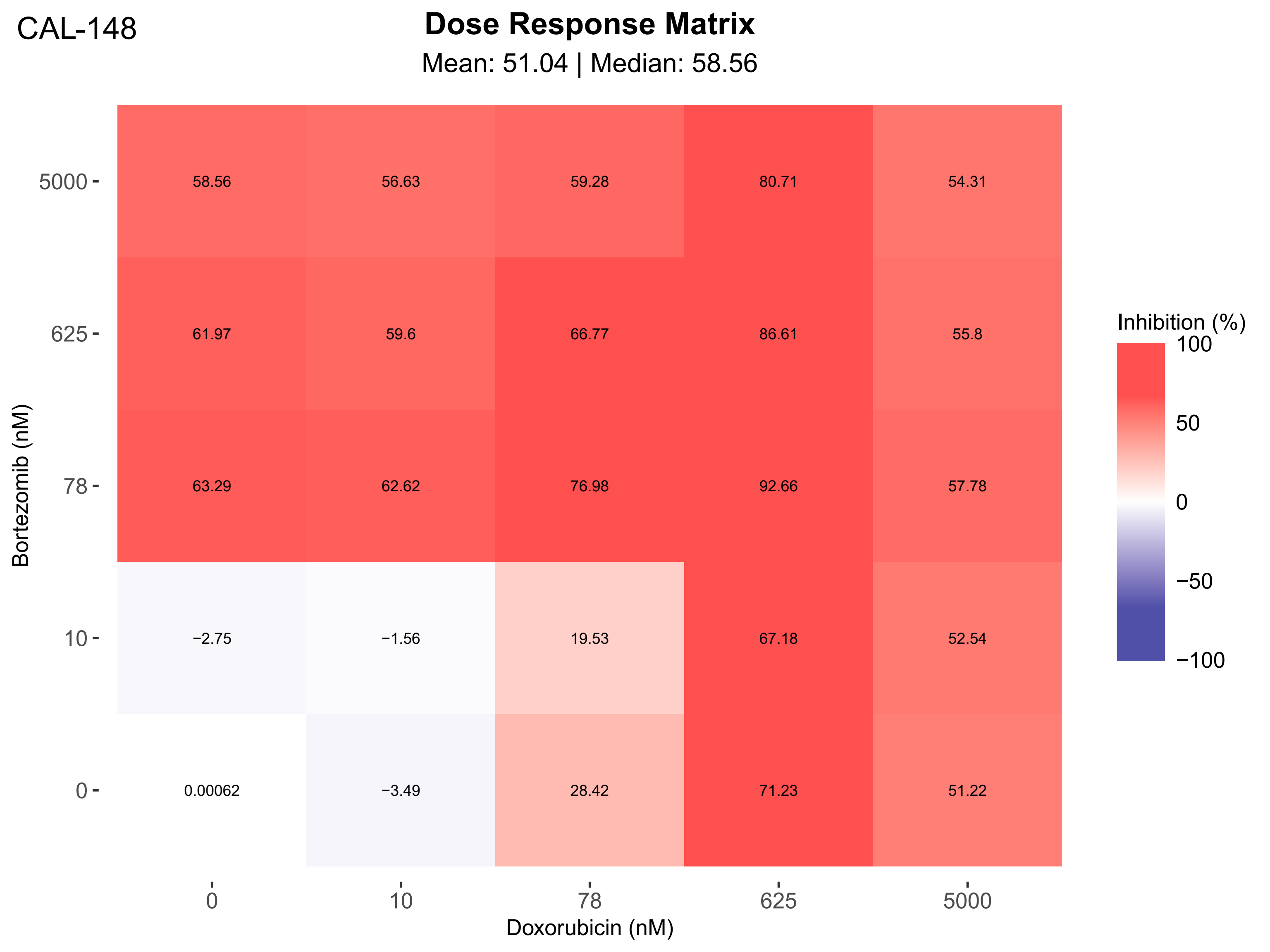
CAL-148
Dose Response Matrix
Mean: 51.04 | Median: 58.56
Inhibition (%)
100
50
0
−50
−100
Bortezomib (nM)
5000
625
78
10
0
Doxorubicin (nM)
0
10
78
625
5000
58.56
56.63
59.28
80.71
54.31
61.97
59.6
66.77
86.61
55.8
63.29
62.62
76.98
92.66
57.78
−2.75
−1.56
19.53
67.18
52.54
0.00062
−3.49
28.42
71.23
51.22

CAL-148

Dose Response Matrix

Mean: 52.78 | Median: 59.33

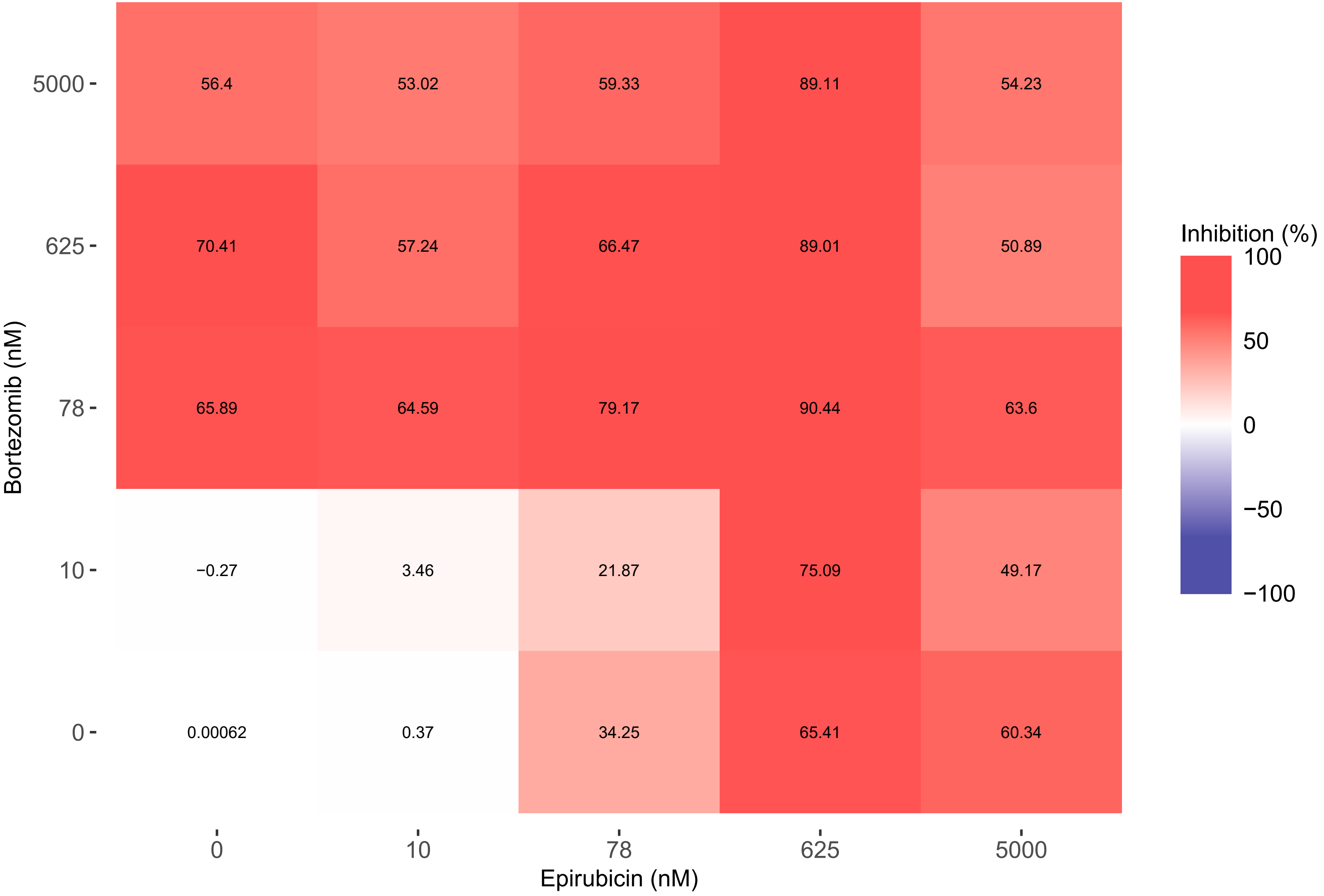

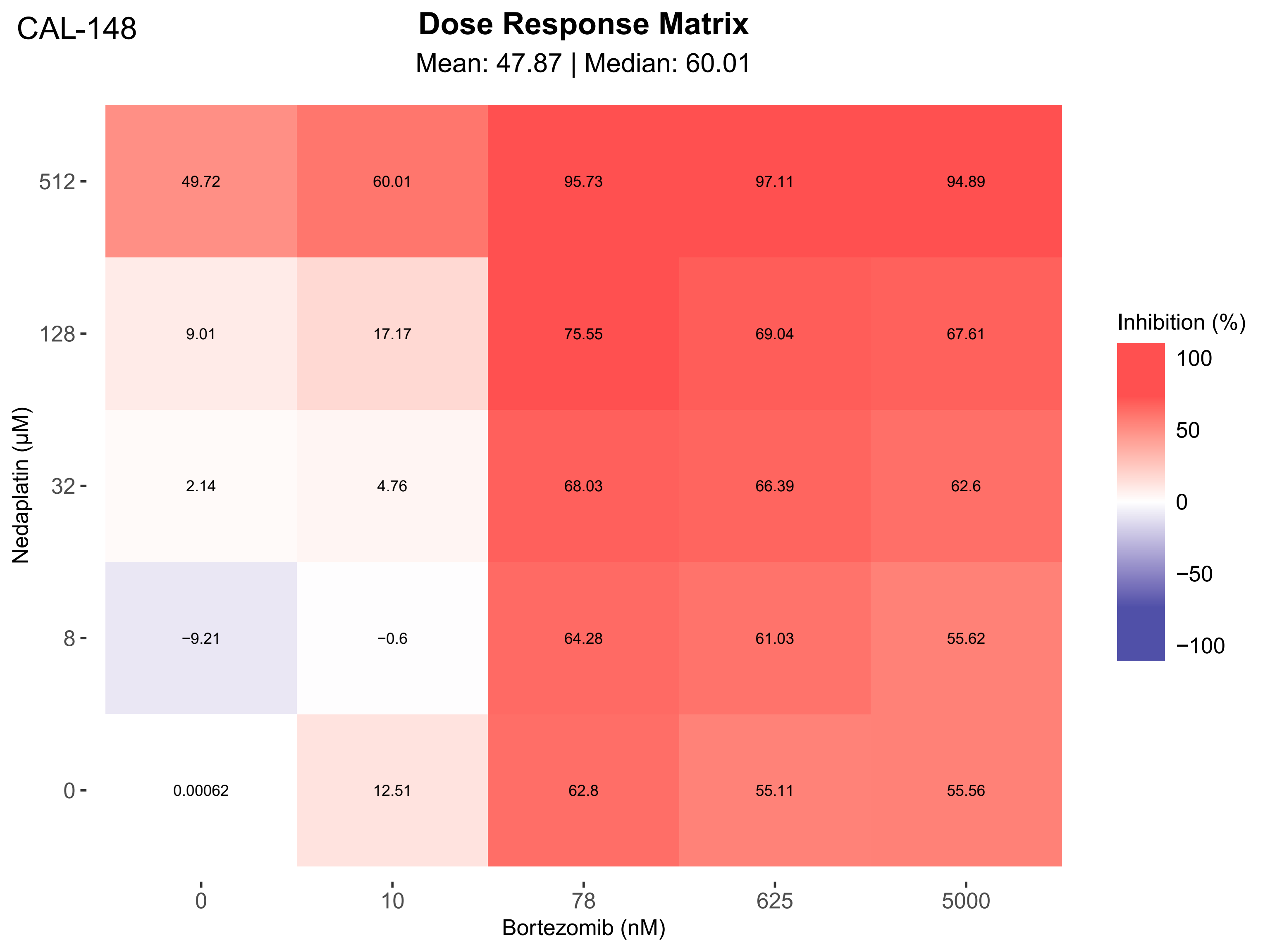
CAL-148
Dose Response Matrix
Mean: 47.87 | Median: 60.01
Nedaplatin (μM)
512
128
32
8
0
49.72
60.01
95.73
97.11
94.89
9.01
17.17
75.55
69.04
67.61
2.14
4.76
68.03
66.39
62.6
−9.21
−0.6
64.28
61.03
55.62
0.00062
12.51
62.8
55.11
55.56
0
10
78
625
5000
Bortezomib (nM)
Inhibition (%)
100
50
0
−50
−100

CAL-148

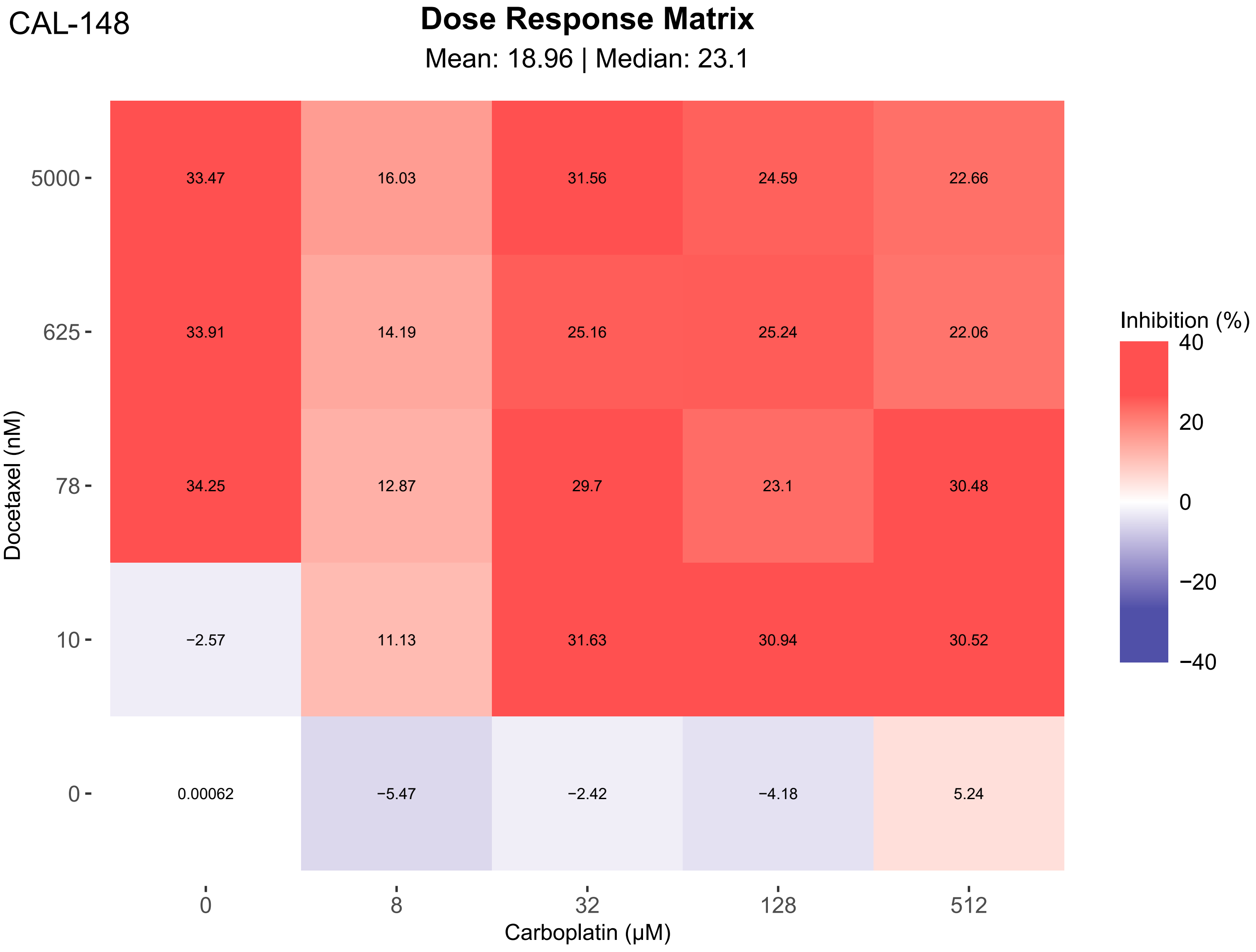
Dose Response Matrix
Mean: 18.96 | Median: 23.1
Docetaxel (nM)
5000
625
78
10
0
33.47
16.03
31.56
24.59
22.66
33.91
14.19
25.16
25.24
22.06
34.25
12.87
29.7
23.1
30.48
−2.57
11.13
31.63
30.94
30.52
0.00062
−5.47
−2.42
−4.18
5.24
0
8
32
128
512
Carboplatin (μM)
Inhibition (%)
40
20
0
−20
−40

CAL-148

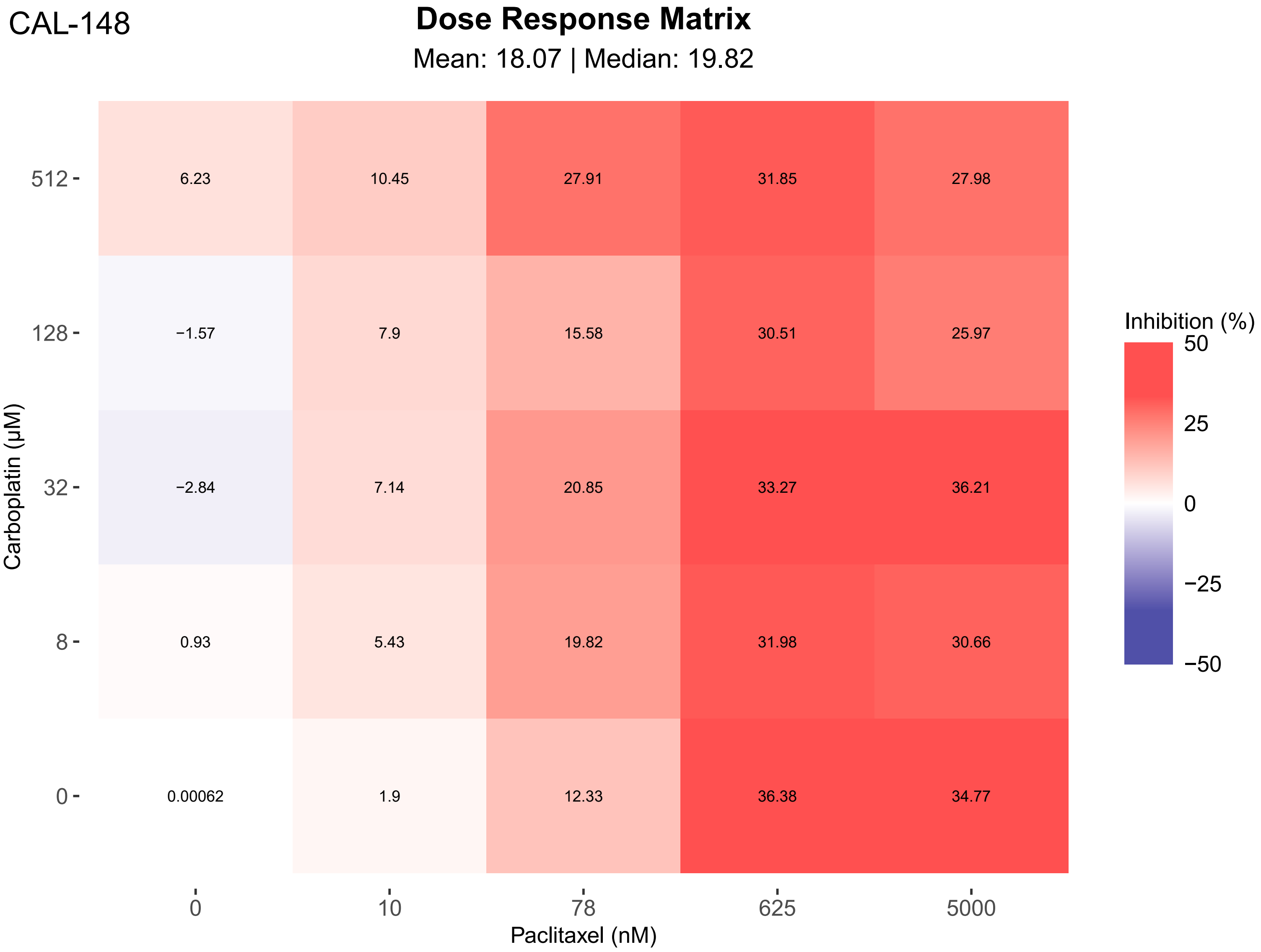

Dose Response Matrix
Mean: 18.07 | Median: 19.82
Carboplatin (μM)
512
128
32
8
0
Paclitaxel (nM)
0
10
78
625
5000
6.23
10.45
27.91
31.85
27.98
−1.57
7.9
15.58
30.51
25.97
−2.84
7.14
20.85
33.27
36.21
0.93
5.43
19.82
31.98
30.66
0.00062
1.9
12.33
36.38
34.77
Inhibition (%)
50
25
0
−25
−50

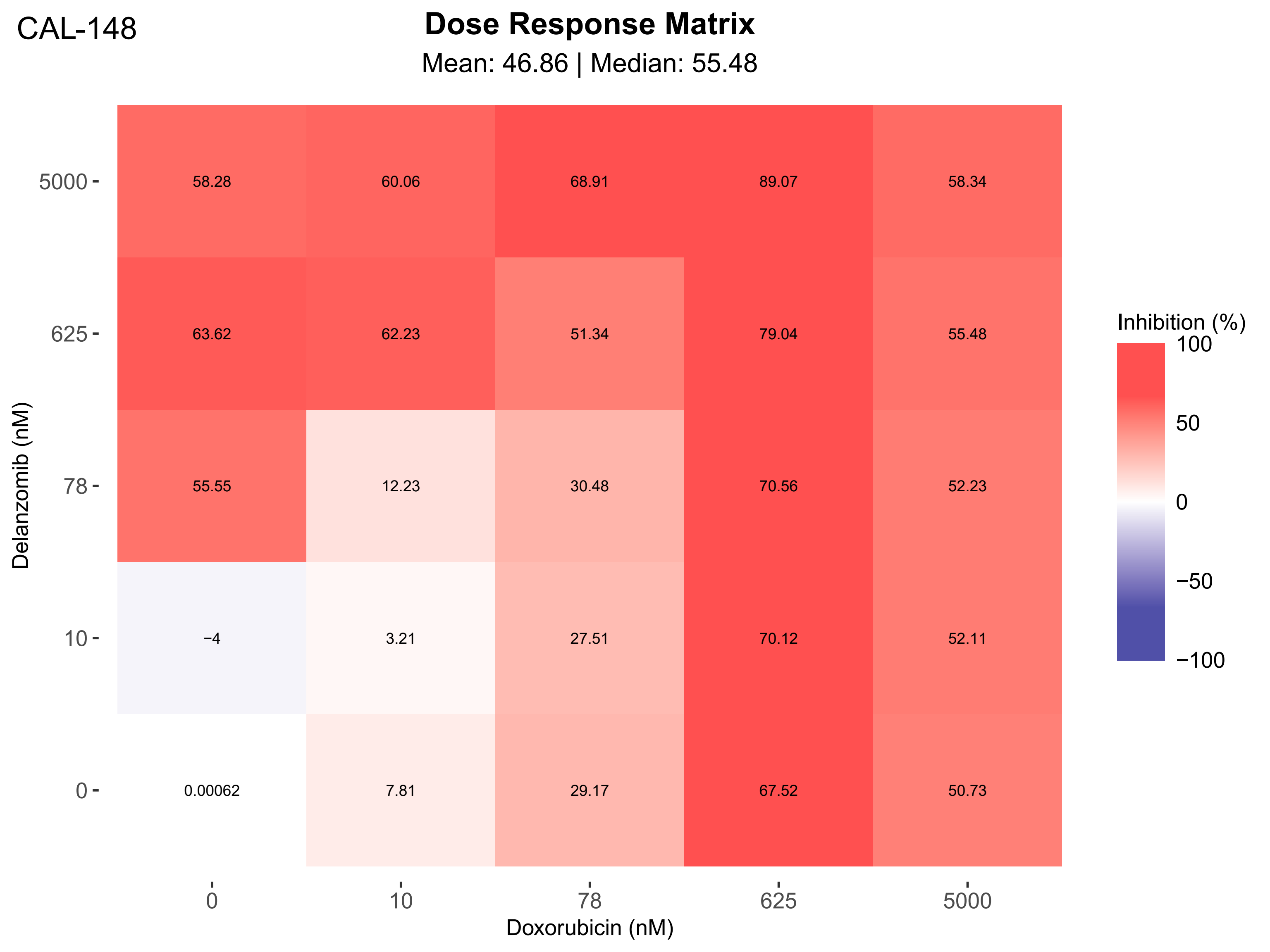
CAL-148
Dose Response Matrix
Mean: 46.86 | Median: 55.48
Delanzomib (nM)
5000
625
78
10
0
58.28
60.06
68.91
89.07
58.34
63.62
62.23
51.34
79.04
55.48
55.55
12.23
30.48
70.56
52.23
−4
3.21
27.51
70.12
52.11
0.00062
7.81
29.17
67.52
50.73
0
10
78
625
5000
Doxorubicin (nM)
Inhibition (%)
100
50
0
−50
−100

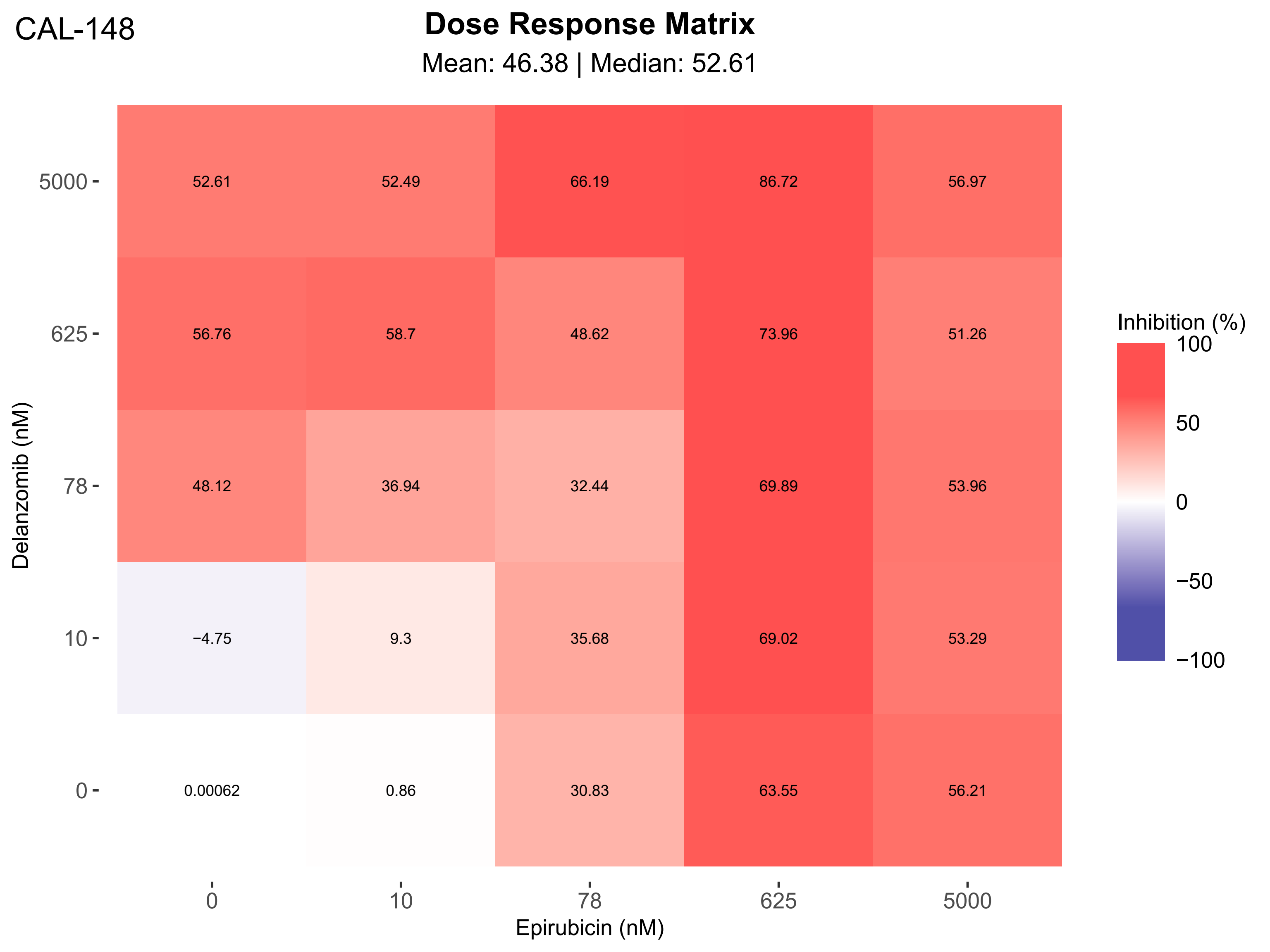
CAL-148
Dose Response Matrix
Mean: 46.38 | Median: 52.61
Delanzomib (nM)
5000
625
78
10
0
52.61
52.49
66.19
86.72
56.97
56.76
58.7
48.62
73.96
51.26
48.12
36.94
32.44
69.89
53.96
−4.75
9.3
35.68
69.02
53.29
0.00062
0.86
30.83
63.55
56.21
0
10
78
625
5000
Epirubicin (nM)
Inhibition (%)
100
50
0
−50
−100

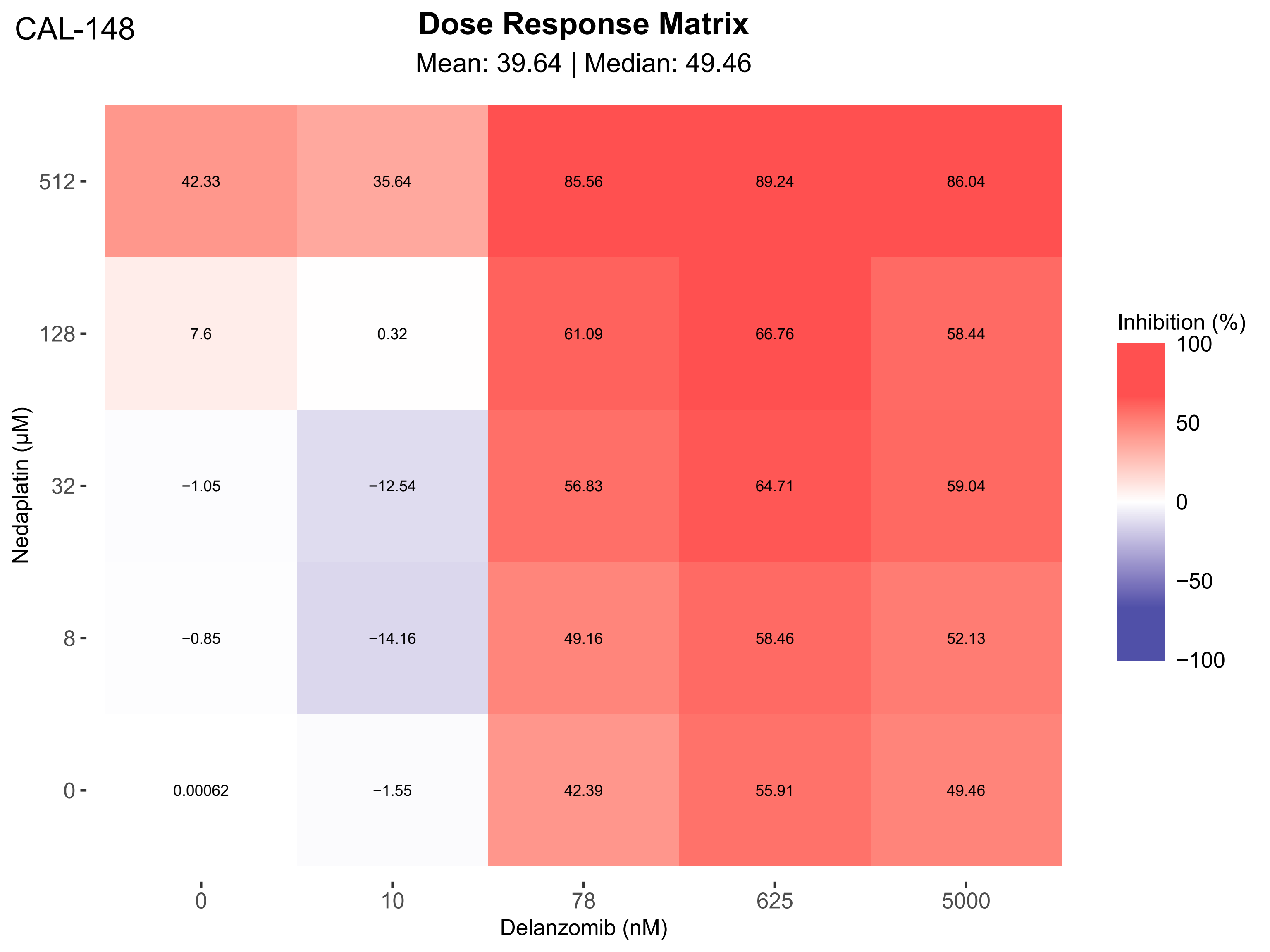
CAL-148
Dose Response Matrix
Mean: 39.64 | Median: 49.46
Nedaplatin (μM)
Delanzomib (nM)
Inhibition (%)
100
50
0
−50
−100
512
128
32
8
0
0
10
78
625
5000
42.33
35.64
85.56
89.24
86.04
7.6
0.32
61.09
66.76
58.44
−1.05
−12.54
56.83
64.71
59.04
−0.85
−14.16
49.16
58.46
52.13
0.00062
−1.55
42.39
55.91
49.46

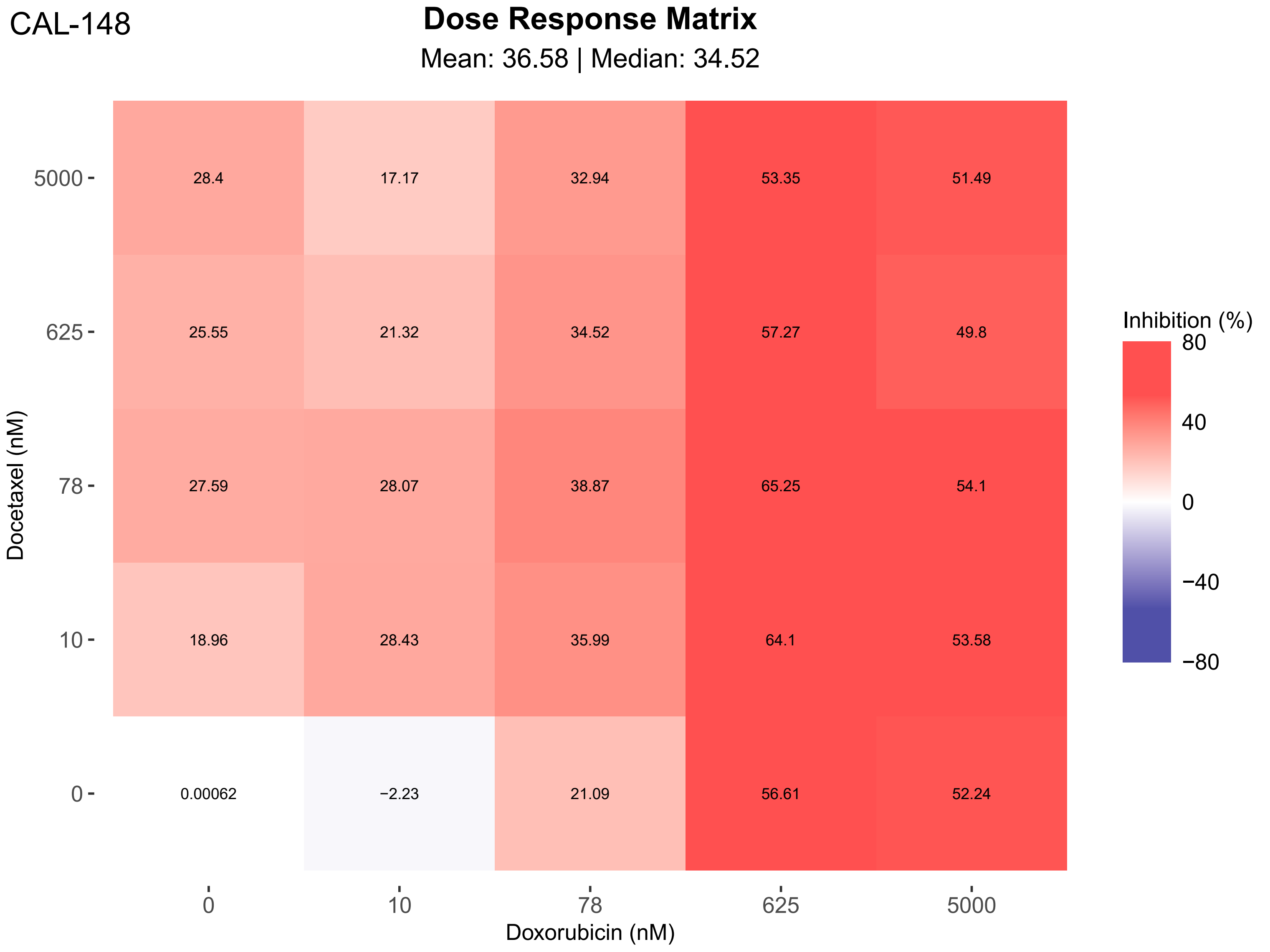
CAL-148
Dose Response Matrix
Mean: 36.58 | Median: 34.52
Docetaxel (nM)
5000
625
78
10
0
Doxorubicin (nM)
0
10
78
625
5000
28.4
17.17
32.94
53.35
51.49
25.55
21.32
34.52
57.27
49.8
27.59
28.07
38.87
65.25
54.1
18.96
28.43
35.99
64.1
53.58
0.00062
−2.23
21.09
56.61
52.24
Inhibition (%)
80
40
0
−40
−80

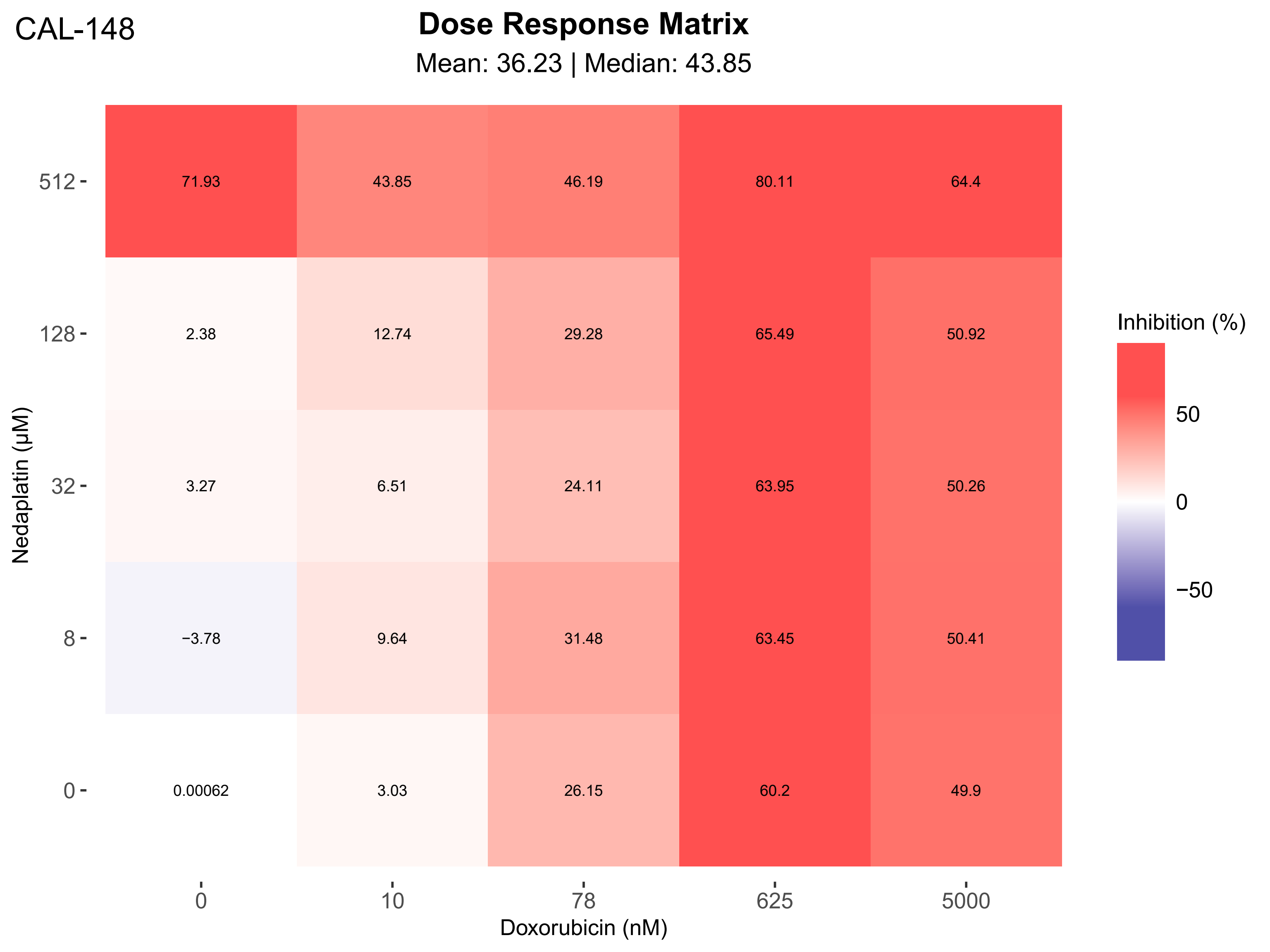
CAL-148
Dose Response Matrix
Mean: 36.23 | Median: 43.85
Nedaplatin (μM)
512
128
32
8
0
Doxorubicin (nM)
0
10
78
625
5000
71.93
43.85
46.19
80.11
64.4
2.38
12.74
29.28
65.49
50.92
3.27
6.51
24.11
63.95
50.26
−3.78
9.64
31.48
63.45
50.41
0.00062
3.03
26.15
60.2
49.9
Inhibition (%)
50
0
−50

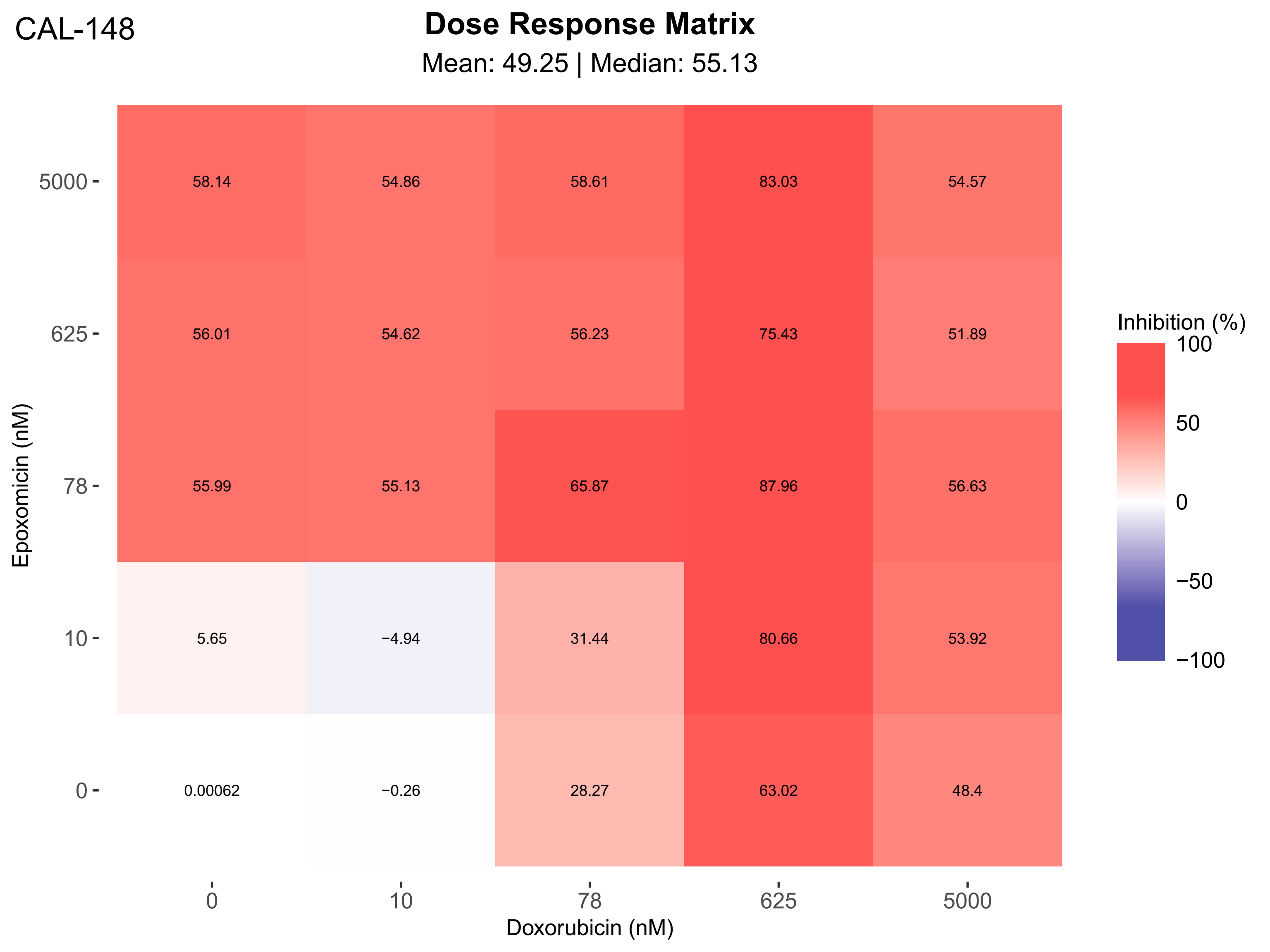
CAL-148
Dose Response Matrix
Mean: 49.25 | Median: 55.13
Epoxomicin (nM)
5000
625
78
10
0
58.14
54.86
58.61
83.03
54.57
56.01
54.62
56.23
75.43
51.89
55.99
55.13
65.87
87.96
56.63
5.65
−4.94
31.44
80.66
53.92
0.00062
−0.26
28.27
63.02
48.4
0
10
78
625
5000
Doxorubicin (nM)
Inhibition (%)
100
50
0
−50
−100

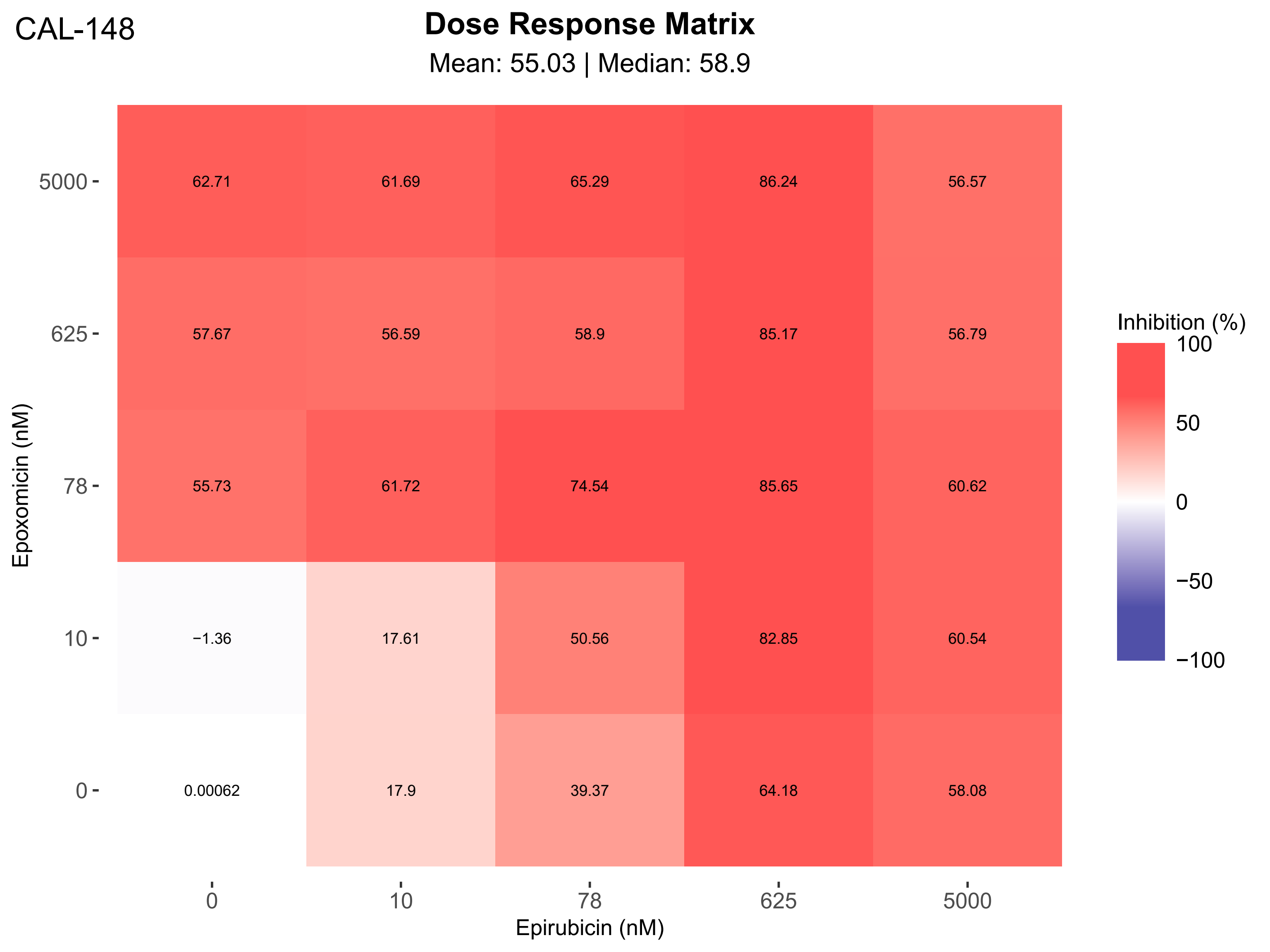
CAL-148
Dose Response Matrix
Mean: 55.03 | Median: 58.9
Epoxomicin (nM)
5000
625
78
10
0
62.71
61.69
65.29
86.24
56.57
57.67
56.59
58.9
85.17
56.79
55.73
61.72
74.54
85.65
60.62
−1.36
17.61
50.56
82.85
60.54
0.00062
17.9
39.37
64.18
58.08
0
10
78
625
5000
Epirubicin (nM)
Inhibition (%)
100
50
0
−50
−100

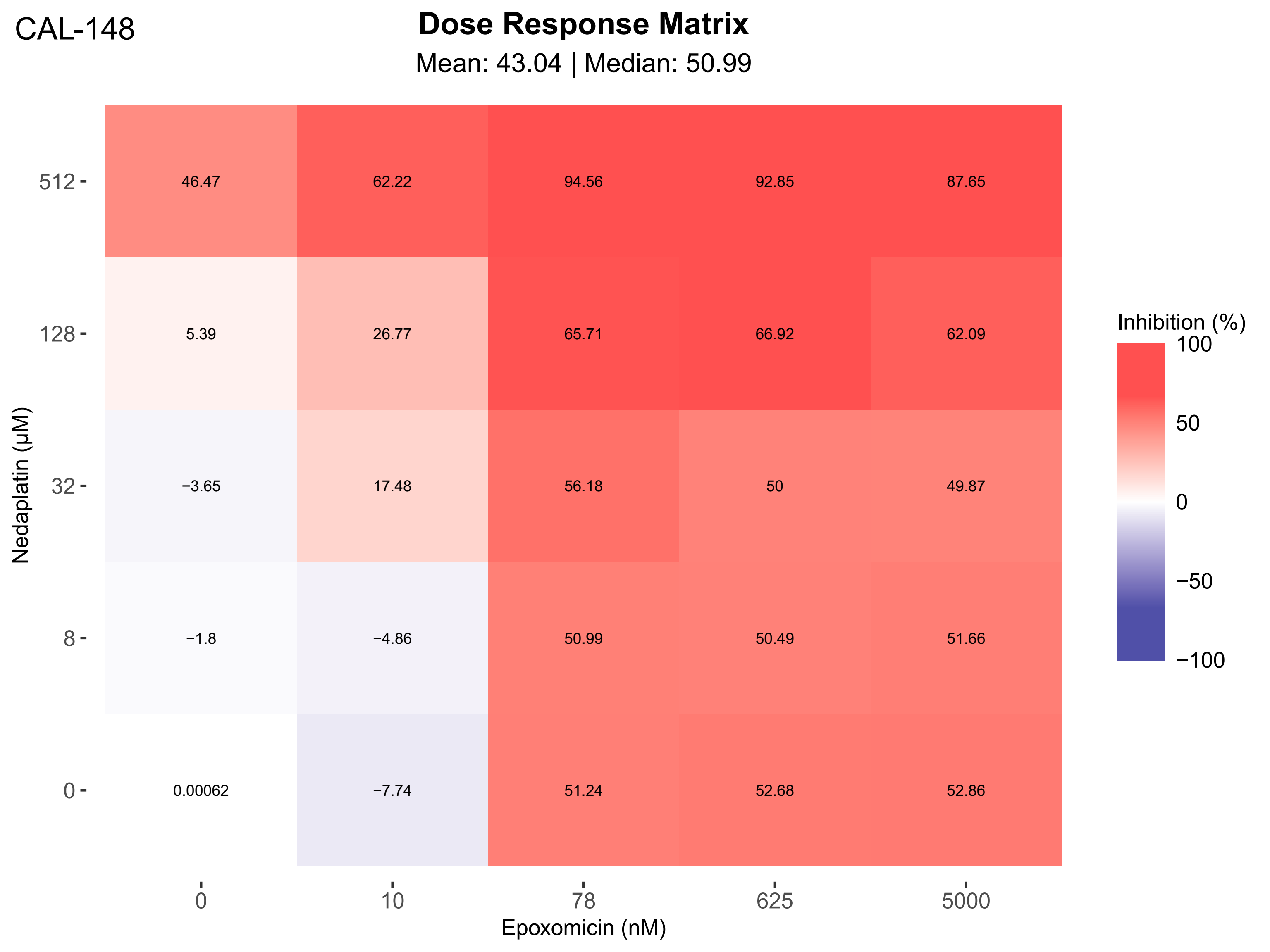

CAL-148
Dose Response Matrix
Mean: 43.04 | Median: 50.99
Inhibition (%)
100
50
0
−50
−100
Nedaplatin (µM)
512
128
32
8
0
Epoxomicin (nM)
0
10
78
625
5000
46.47
62.22
94.56
92.85
87.65
5.39
26.77
65.71
66.92
62.09
−3.65
17.48
56.18
50
49.87
−1.8
−4.86
50.99
50.49
51.66
0.00062
−7.74
51.24
52.68
52.86

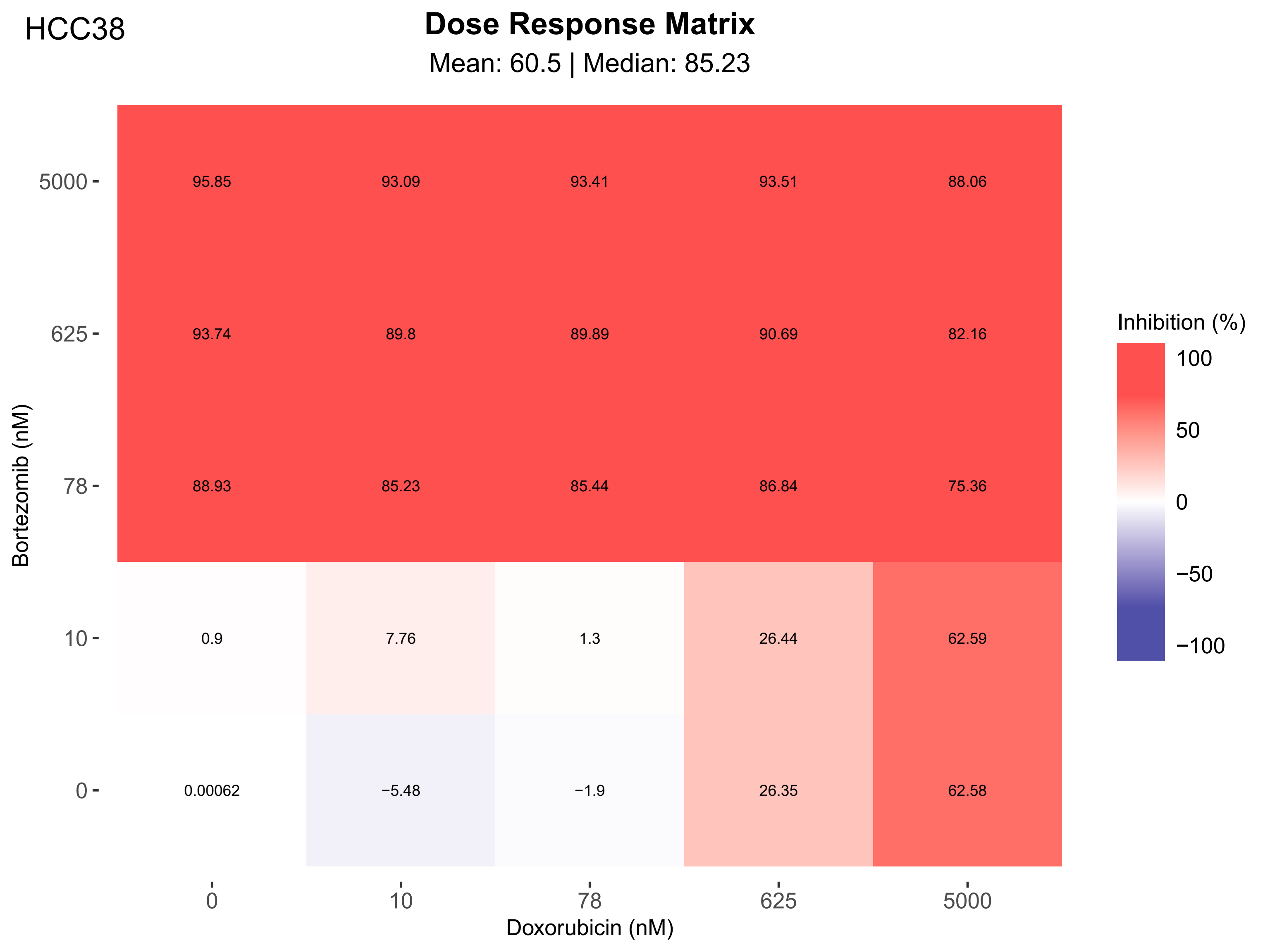
HCC38
Dose Response Matrix
Mean: 60.5 | Median: 85.23
Bortezomib (nM)
5000
625
78
10
0
Doxorubicin (nM)
0
10
78
625
5000
95.85
93.09
93.41
93.51
88.06
93.74
89.8
89.89
90.69
82.16
88.93
85.23
85.44
86.84
75.36
0.9
7.76
1.3
26.44
62.59
0.00062
−5.48
−1.9
26.35
62.58
Inhibition (%)
100
50
0
−50
−100

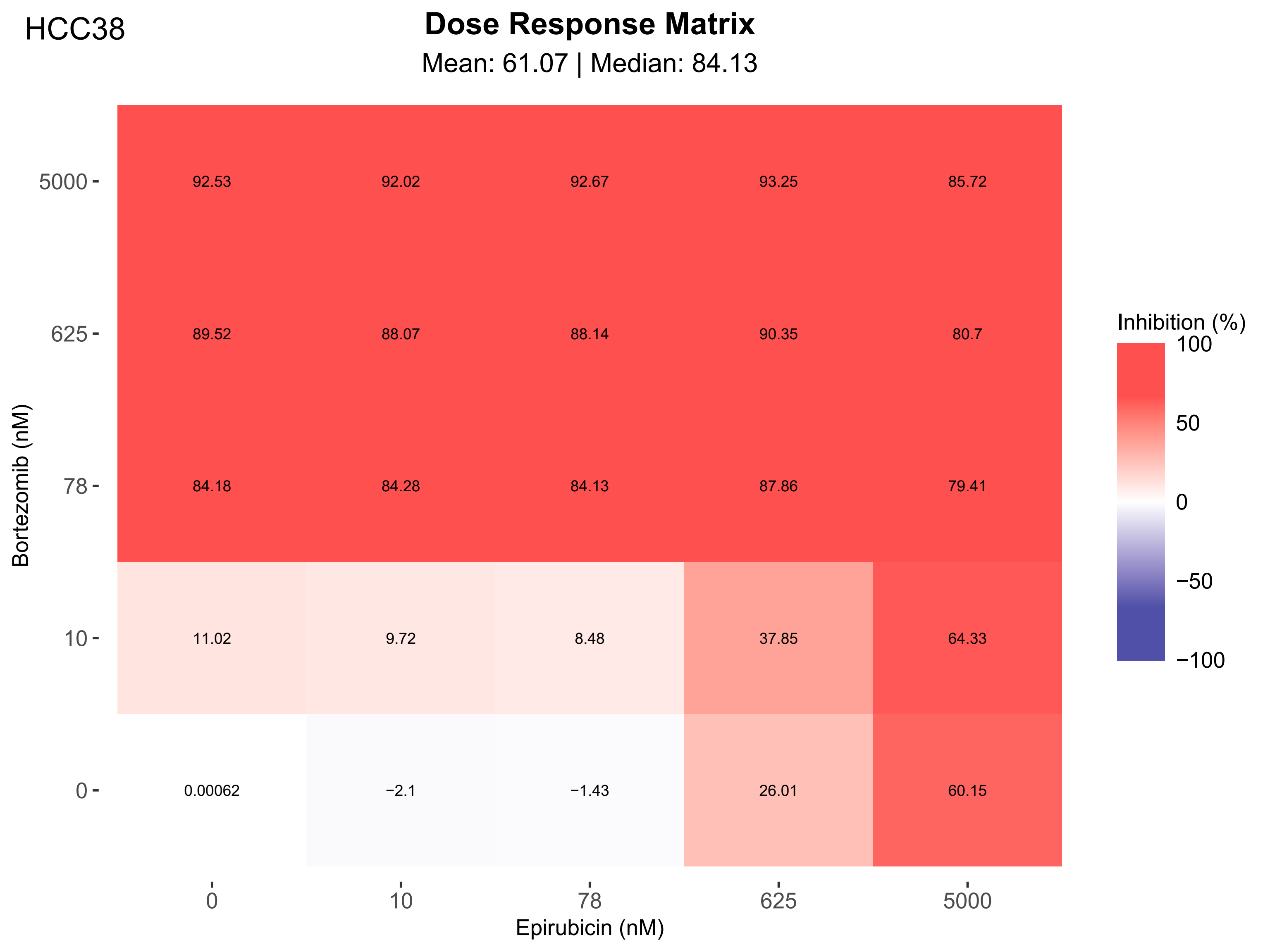
HCC38
Dose Response Matrix
Mean: 61.07 | Median: 84.13
92.53
92.02
92.67
93.25
85.72
89.52
88.07
88.14
90.35
80.7
84.18
84.28
84.13
87.86
79.41
11.02
9.72
8.48
37.85
64.33
0.00062
−2.1
−1.43
26.01
60.15
5000
625
78
10
0
Bortezomib (nM)
0
10
78
625
5000
Epirubicin (nM)
Inhibition (%)
100
50
0
−50
−100

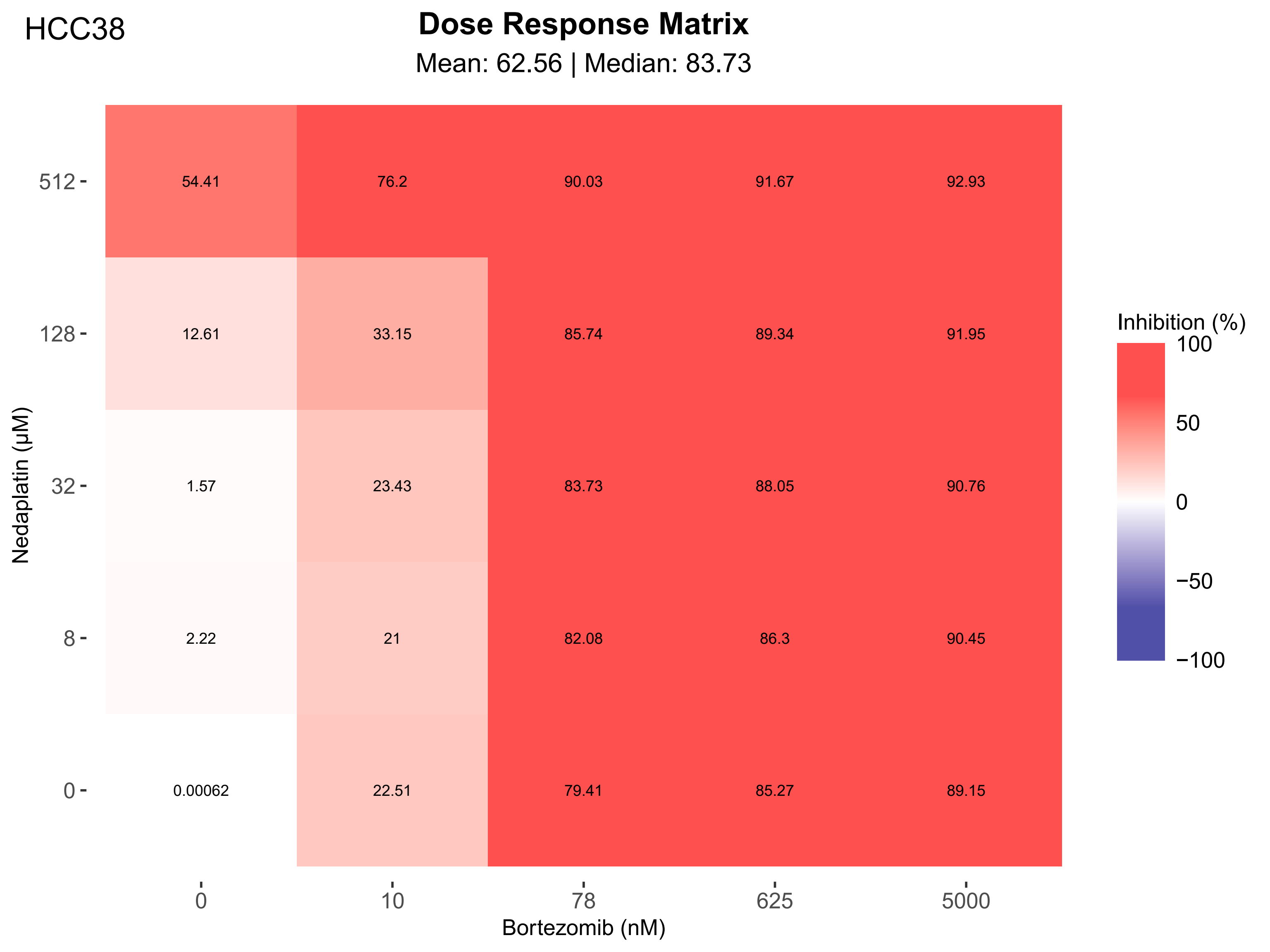
HCC38
Dose Response Matrix
Mean: 62.56 | Median: 83.73
Nedaplatin (µM)
512
128
32
8
0
Bortezomib (nM)
0
10
78
625
5000
54.41
76.2
90.03
91.67
92.93
12.61
33.15
85.74
89.34
91.95
1.57
23.43
83.73
88.05
90.76
2.22
21
82.08
86.3
90.45
0.00062
22.51
79.41
85.27
89.15
Inhibition (%)
100
50
0
−50
−100

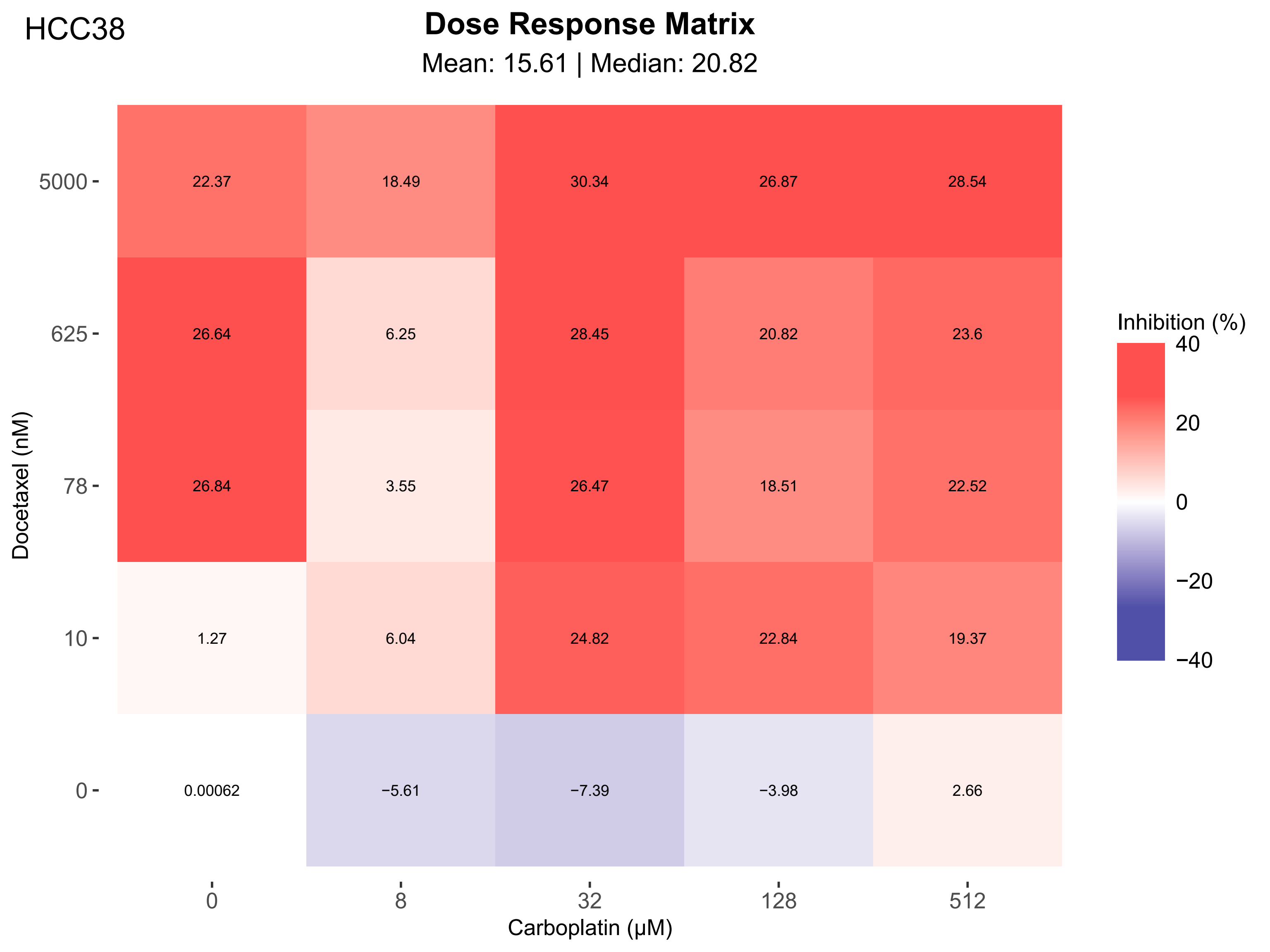
HCC38
Dose Response Matrix
Mean: 15.61 | Median: 20.82
Docetaxel (nM)
5000
625
78
10
0
22.37
18.49
30.34
26.87
28.54
26.64
6.25
28.45
20.82
23.6
26.84
3.55
26.47
18.51
22.52
1.27
6.04
24.82
22.84
19.37
0.00062
−5.61
−7.39
−3.98
2.66
0
8
32
128
512
Carboplatin (µM)
Inhibition (%)
40
20
0
−20
−40

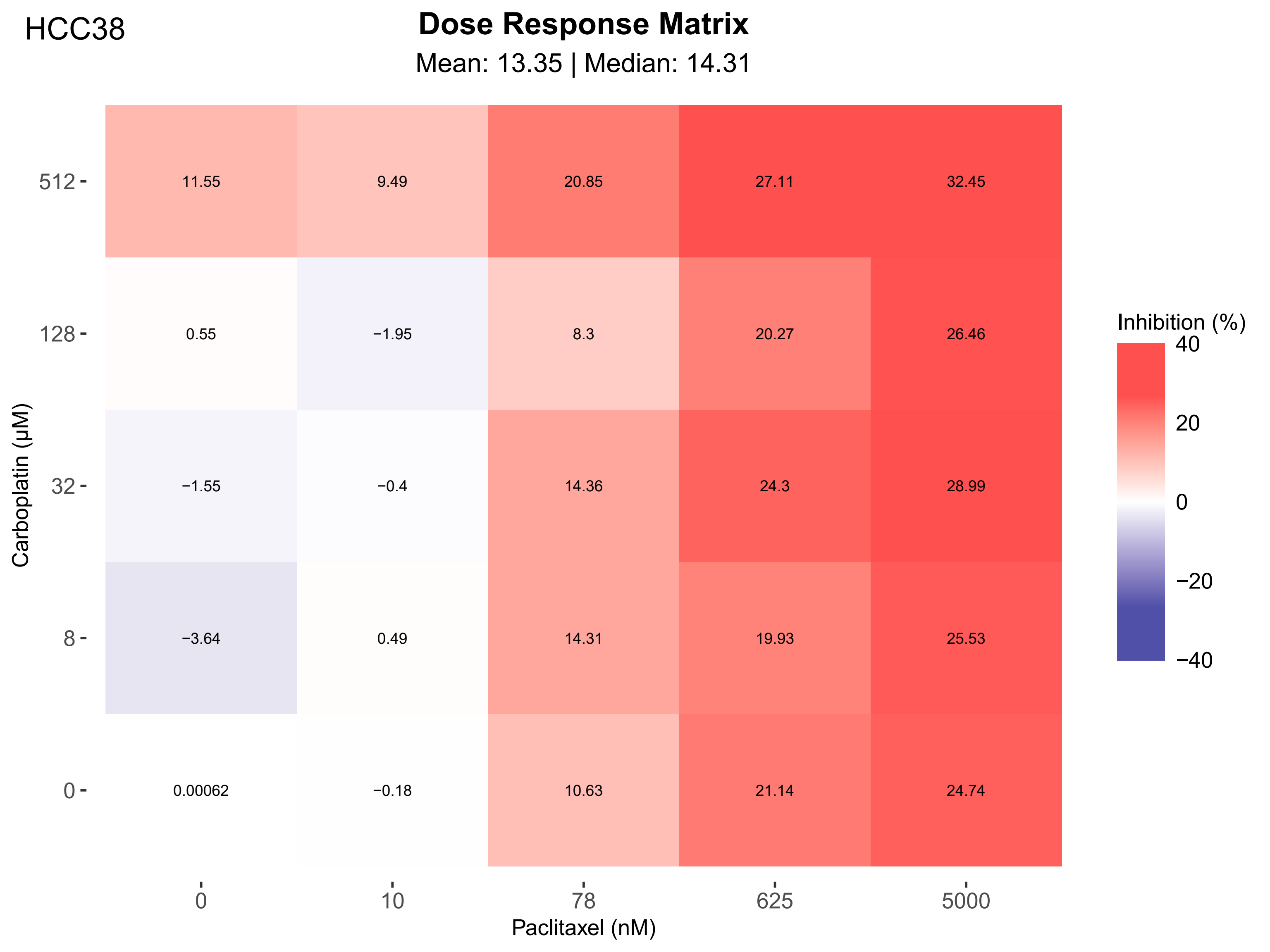
HCC38
Dose Response Matrix
Mean: 13.35 | Median: 14.31
512
128
32
8
0
Carboplatin (μM)
11.55
9.49
20.85
27.11
32.45
0.55
−1.95
8.3
20.27
26.46
−1.55
−0.4
14.36
24.3
28.99
−3.64
0.49
14.31
19.93
25.53
0.00062
−0.18
10.63
21.14
24.74
0
10
78
625
5000
Paclitaxel (nM)
Inhibition (%)
40
20
0
−20
−40

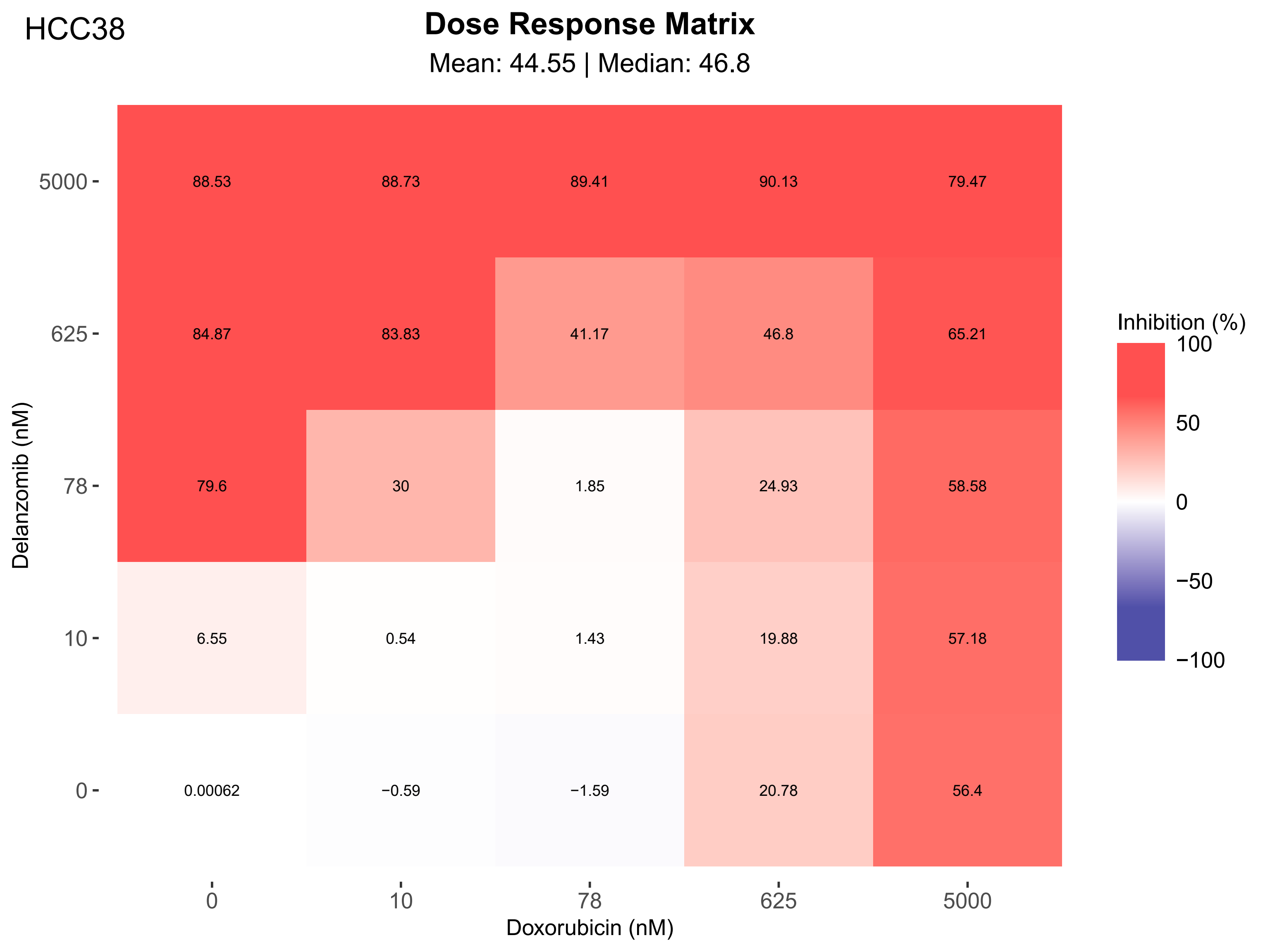
HCC38
Dose Response Matrix
Mean: 44.55 | Median: 46.8
Delanzomib (nM)
5000
625
78
10
0
88.53
88.73
89.41
90.13
79.47
84.87
83.83
41.17
46.8
65.21
79.6
30
1.85
24.93
58.58
6.55
0.54
1.43
19.88
57.18
0.00062
−0.59
−1.59
20.78
56.4
0
10
78
625
5000
Doxorubicin (nM)
Inhibition (%)
100
50
0
−50
−100

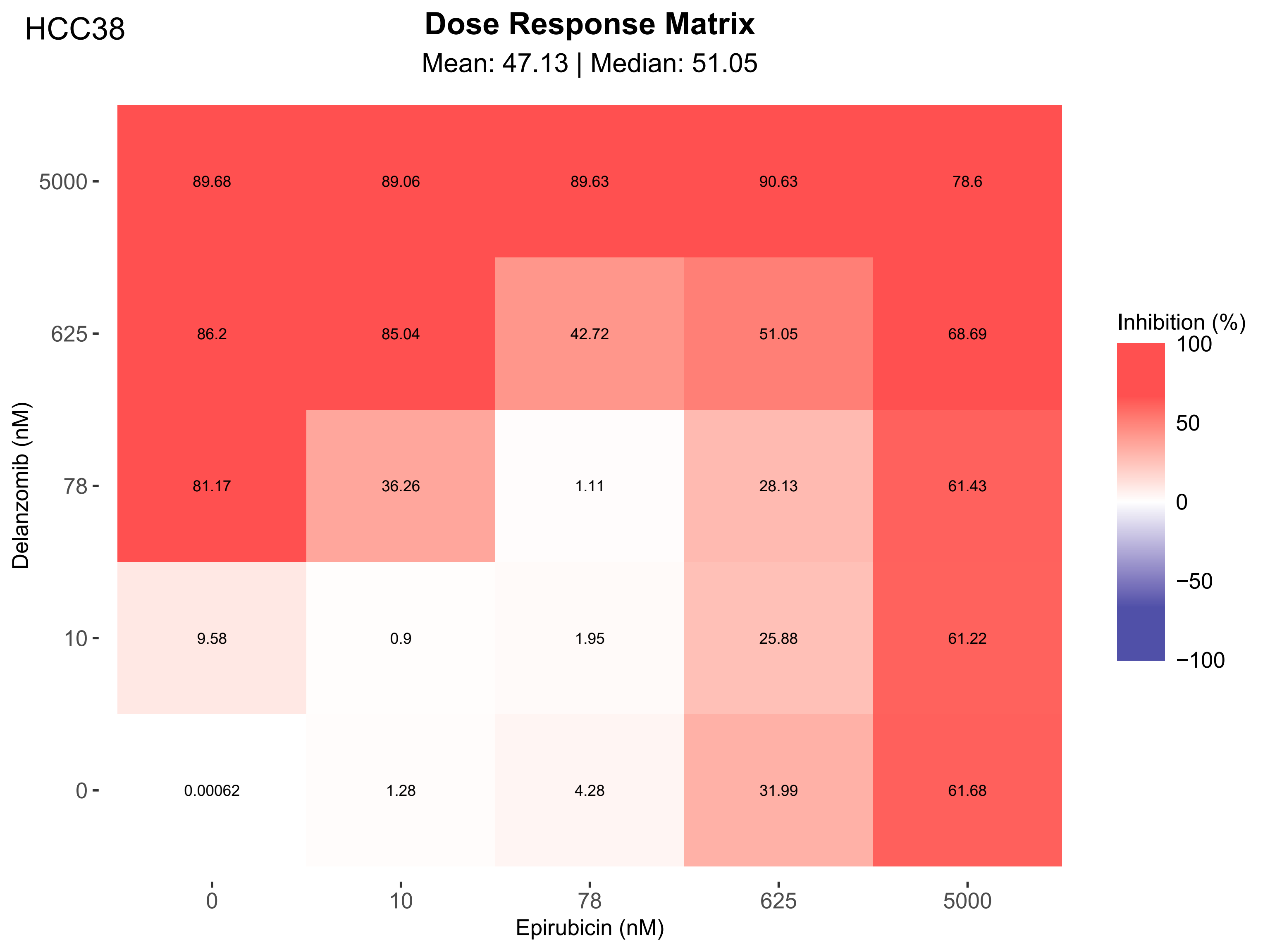
HCC38
Dose Response Matrix
Mean: 47.13 | Median: 51.05
Delanzomib (nM)
5000
625
78
10
0
89.68
89.06
89.63
90.63
78.6
86.2
85.04
42.72
51.05
68.69
81.17
36.26
1.11
28.13
61.43
9.58
0.9
1.95
25.88
61.22
0.00062
1.28
4.28
31.99
61.68
0
10
78
625
5000
Epirubicin (nM)
Inhibition (%)
100
50
0
−50
−100

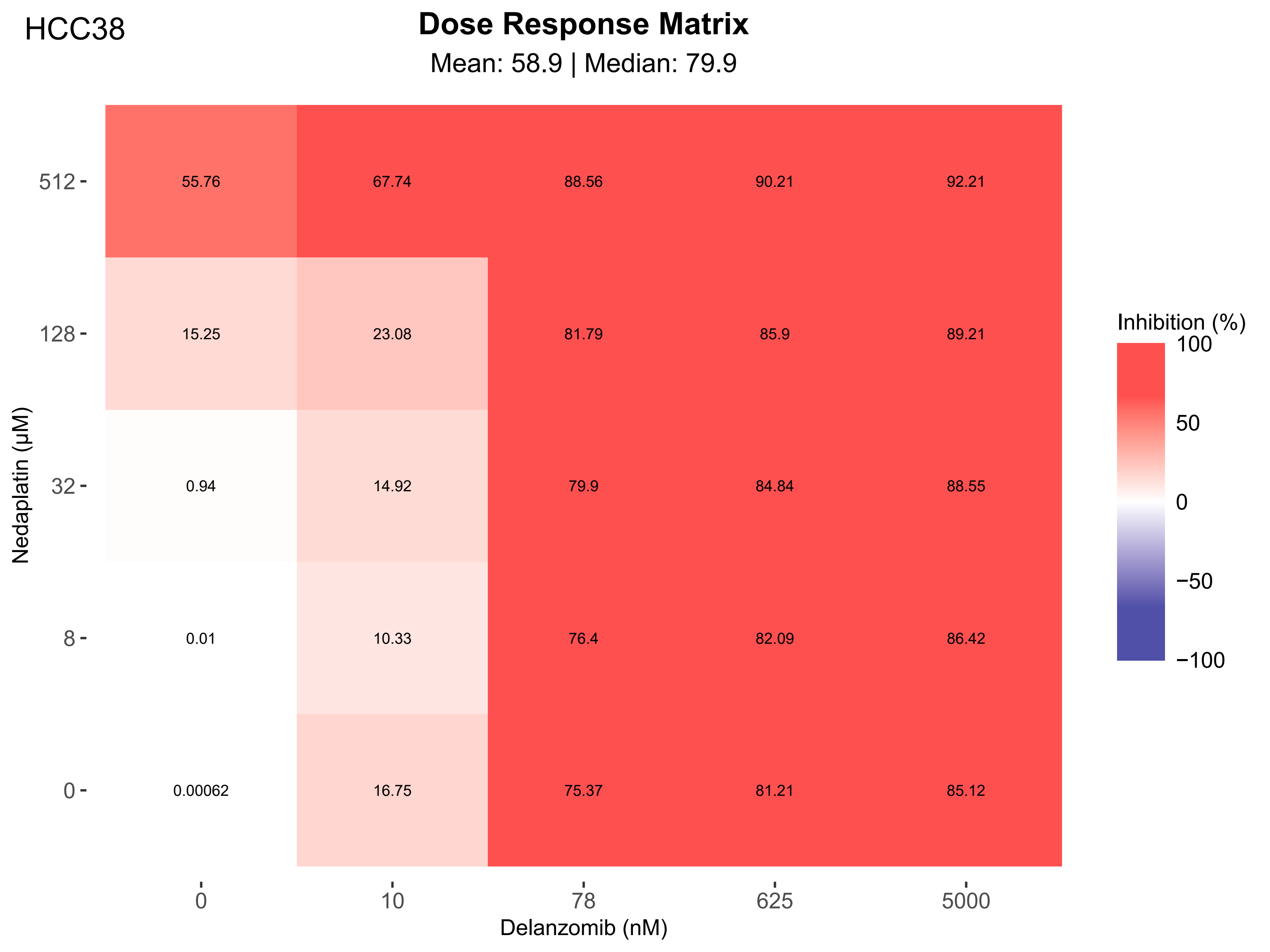
HCC38
Dose Response Matrix
Mean: 58.9 | Median: 79.9
Nedaplatin (μM)
512
128
32
8
0
55.76
67.74
88.56
90.21
92.21
15.25
23.08
81.79
85.9
89.21
0.94
14.92
79.9
84.84
88.55
0.01
10.33
76.4
82.09
86.42
0.00062
16.75
75.37
81.21
85.12
0
10
78
625
5000
Delanzomib (nM)
Inhibition (%)
100
50
0
−50
−100

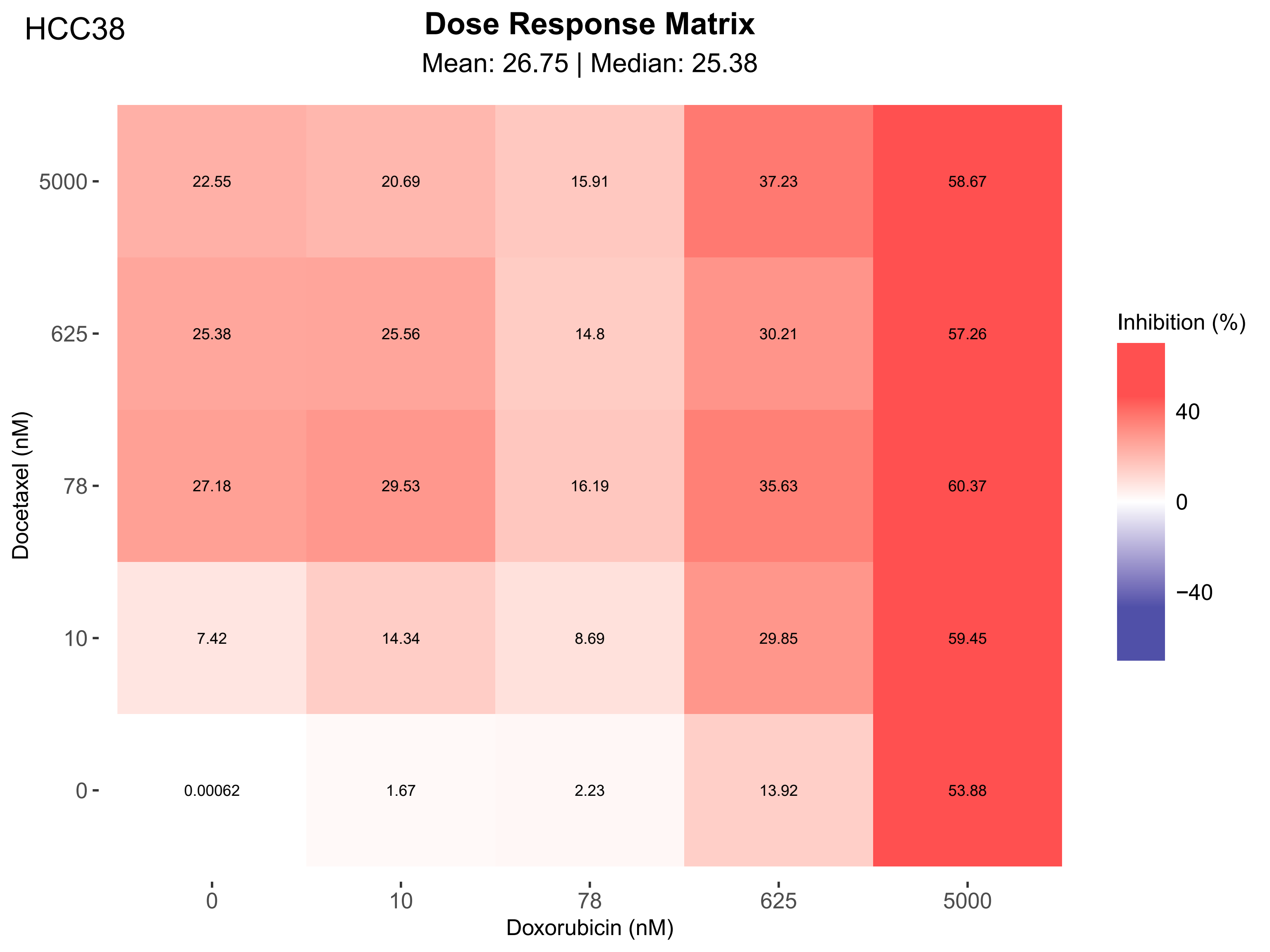

HCC38
Dose Response Matrix
Mean: 26.75 | Median: 25.38
Docetaxel (nM)
5000
625
78
10
0
22.55
20.69
15.91
37.23
58.67
25.38
25.56
14.8
30.21
57.26
27.18
29.53
16.19
35.63
60.37
7.42
14.34
8.69
29.85
59.45
0.00062
1.67
2.23
13.92
53.88
0
10
78
625
5000
Doxorubicin (nM)
Inhibition (%)
40
0
−40

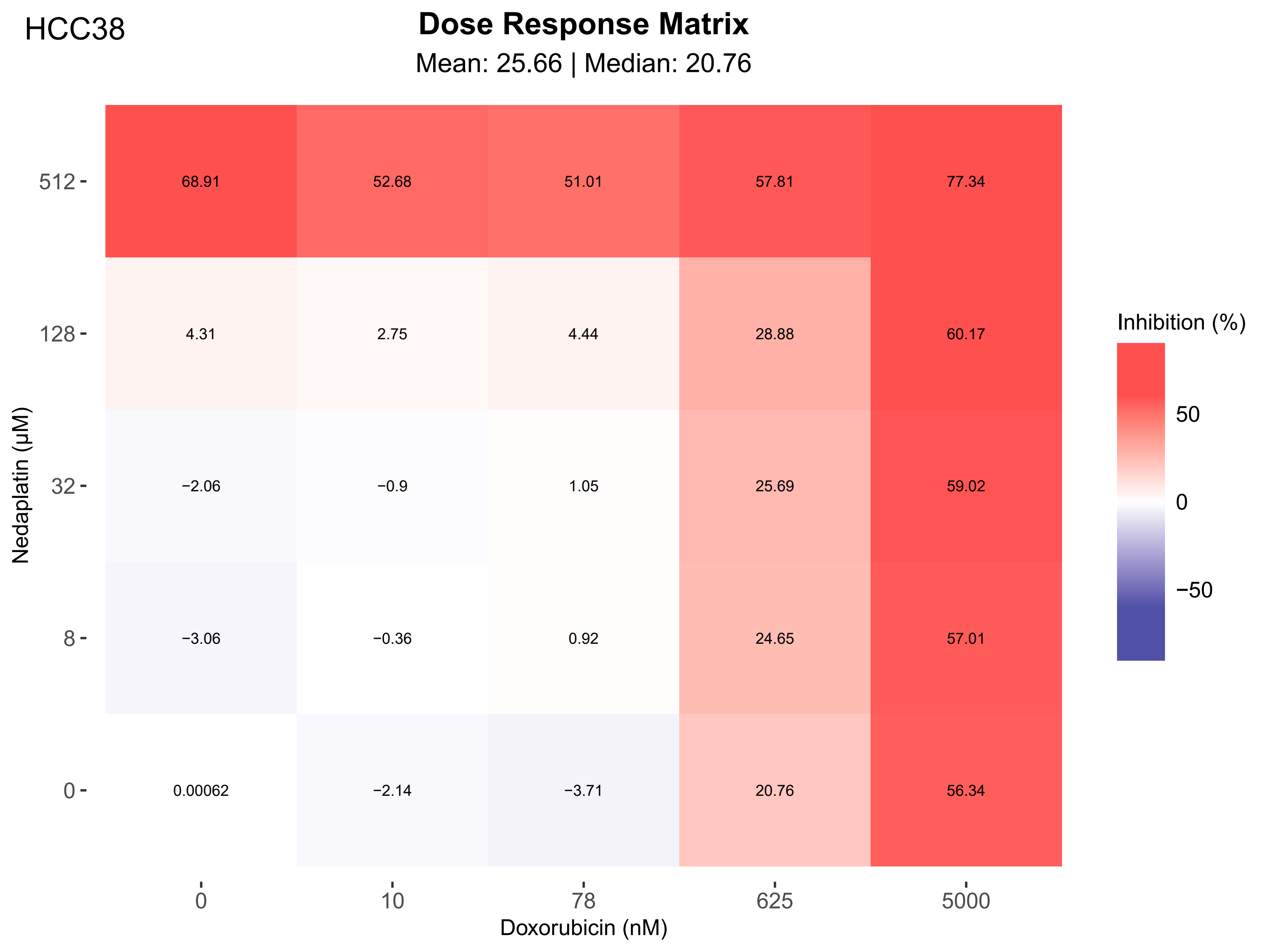
HCC38
Dose Response Matrix
Mean: 25.66 | Median: 20.76
Nedaplatin (μM)
512
128
32
8
0
68.91
52.68
51.01
57.81
77.34
4.31
2.75
4.44
28.88
60.17
−2.06
−0.9
1.05
25.69
59.02
−3.06
−0.36
0.92
24.65
57.01
0.00062
−2.14
−3.71
20.76
56.34
0
10
78
625
5000
Doxorubicin (nM)
Inhibition (%)
50
0
−50

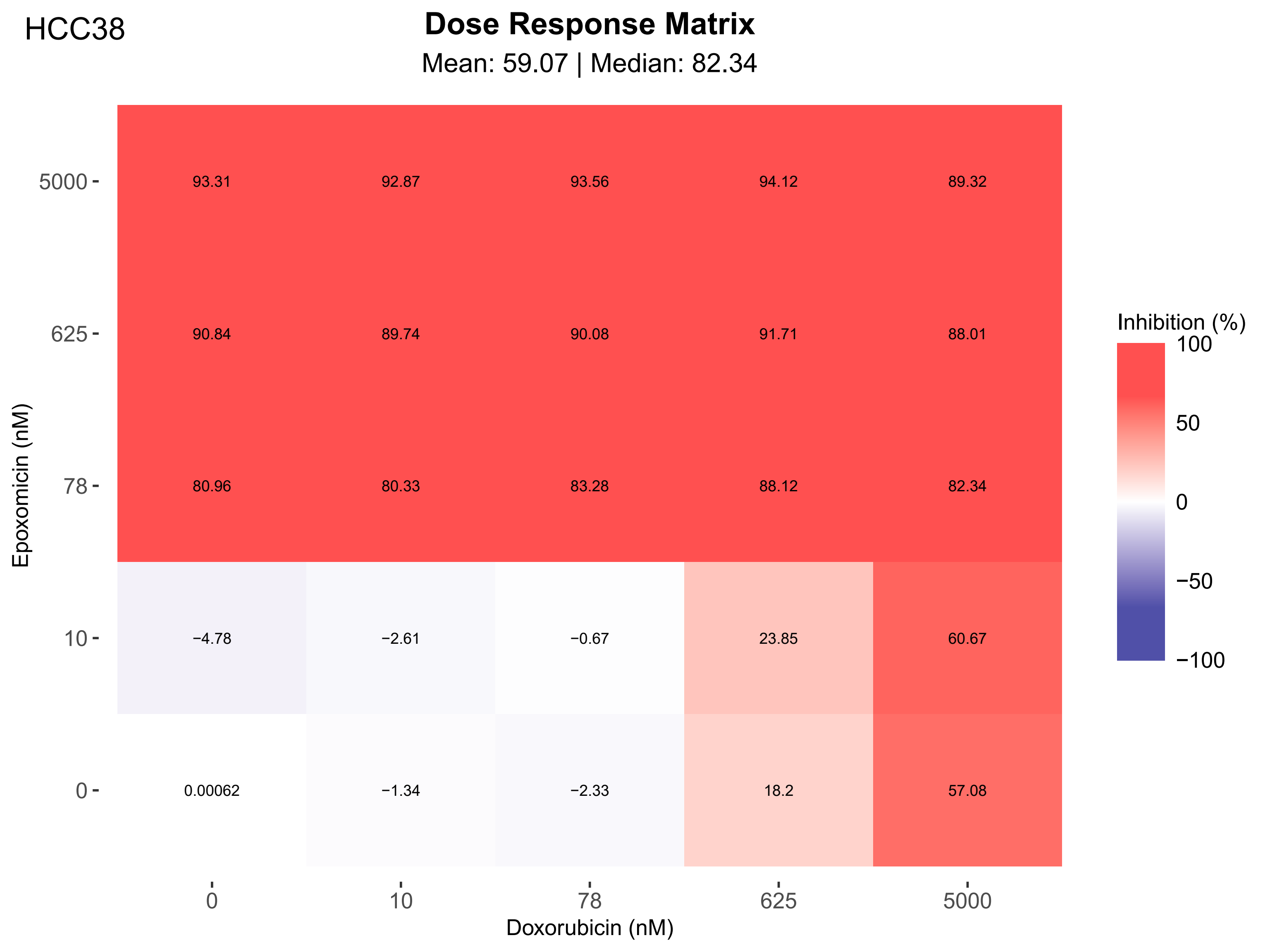
HCC38
Dose Response Matrix
Mean: 59.07 | Median: 82.34
Epoxomicin (nM)
5000
625
78
10
0
93.31
92.87
93.56
94.12
89.32
90.84
89.74
90.08
91.71
88.01
80.96
80.33
83.28
88.12
82.34
−4.78
−2.61
−0.67
23.85
60.67
0.00062
−1.34
−2.33
18.2
57.08
0
10
78
625
5000
Doxorubicin (nM)
Inhibition (%)
100
50
0
−50
−100

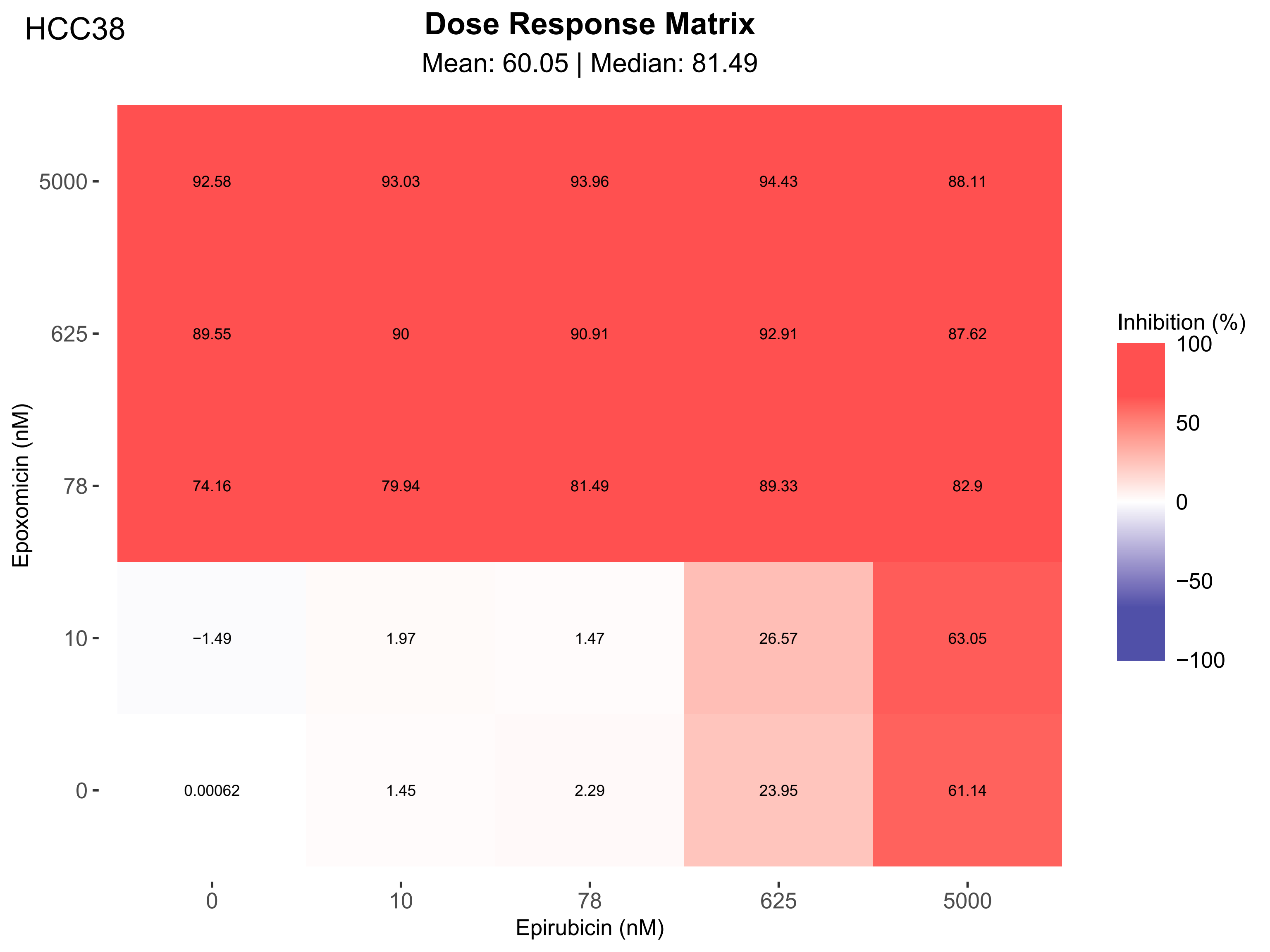
HCC38
Dose Response Matrix
Mean: 60.05 | Median: 81.49
Epoxomicin (nM)
5000
625
78
10
0
92.58
93.03
93.96
94.43
88.11
89.55
90
90.91
92.91
87.62
74.16
79.94
81.49
89.33
82.9
−1.49
1.97
1.47
26.57
63.05
0.00062
1.45
2.29
23.95
61.14
0
10
78
625
5000
Epirubicin (nM)
Inhibition (%)
100
50
0
−50
−100

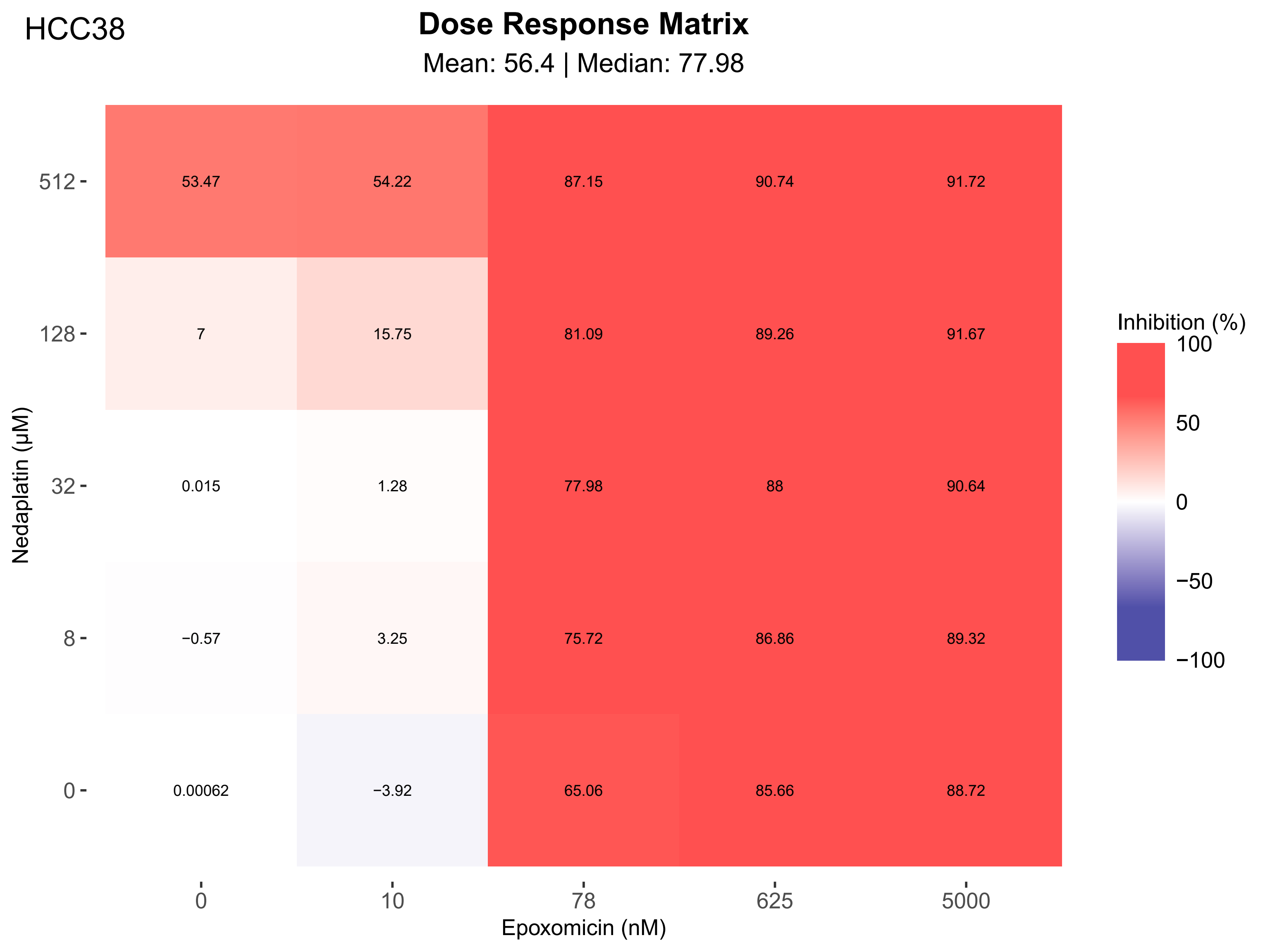

HCC38
Dose Response Matrix
Mean: 56.4 | Median: 77.98
Nedaplatin (μM)
512
128
32
8
0
Epoxomicin (nM)
0
10
78
625
5000
53.47
54.22
87.15
90.74
91.72
7
15.75
81.09
89.26
91.67
0.015
1.28
77.98
88
90.64
−0.57
3.25
75.72
86.86
89.32
0.00062
−3.92
65.06
85.66
88.72
Inhibition (%)
100
50
0
−50
−100

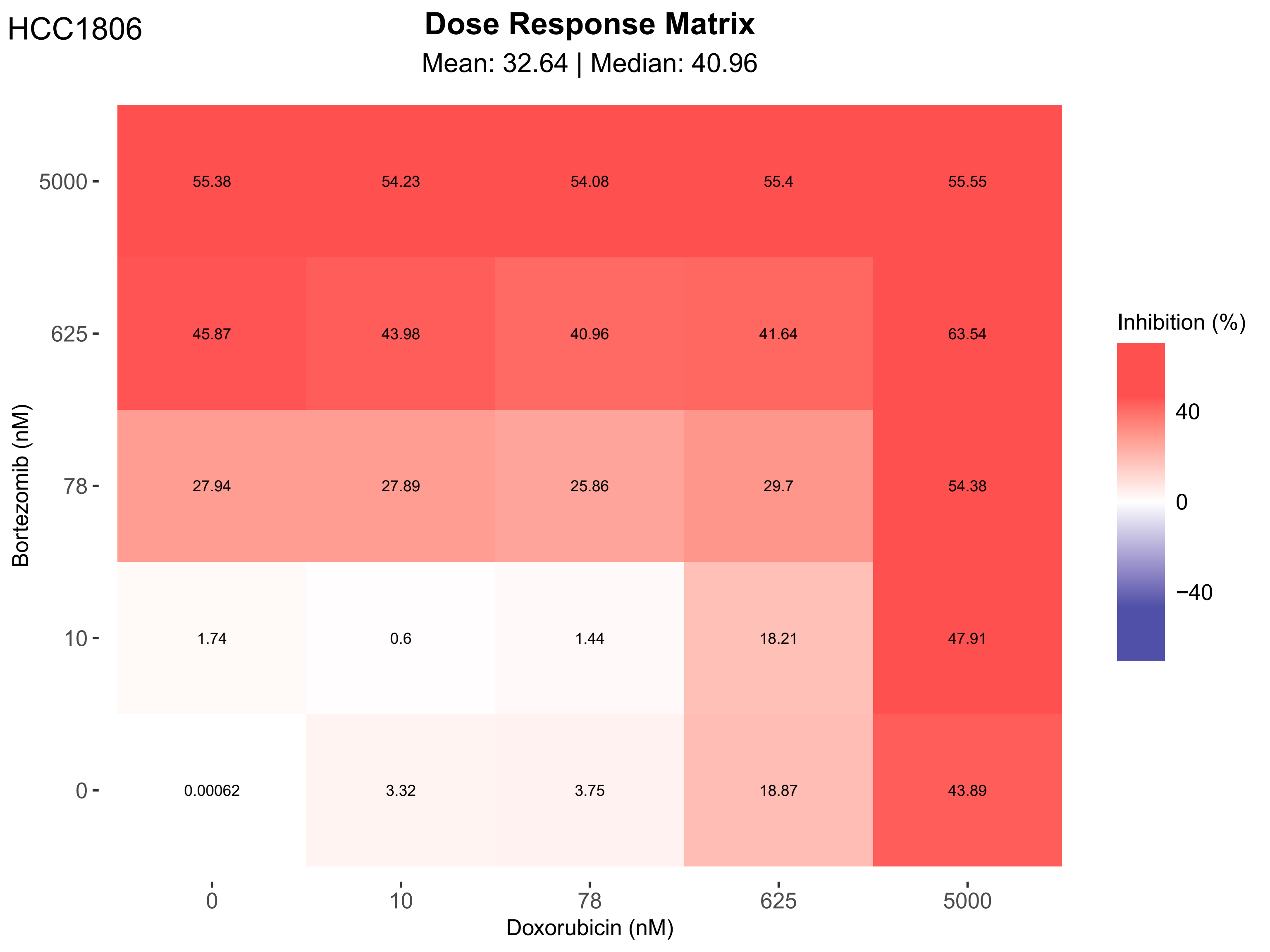
HCC1806
Dose Response Matrix
Mean: 32.64 | Median: 40.96
Bortezomib (nM)
5000
625
78
10
0
Doxorubicin (nM)
0
10
78
625
5000
55.38
54.23
54.08
55.4
55.55
45.87
43.98
40.96
41.64
63.54
27.94
27.89
25.86
29.7
54.38
1.74
0.6
1.44
18.21
47.91
0.00062
3.32
3.75
18.87
43.89
Inhibition (%)
40
0
−40

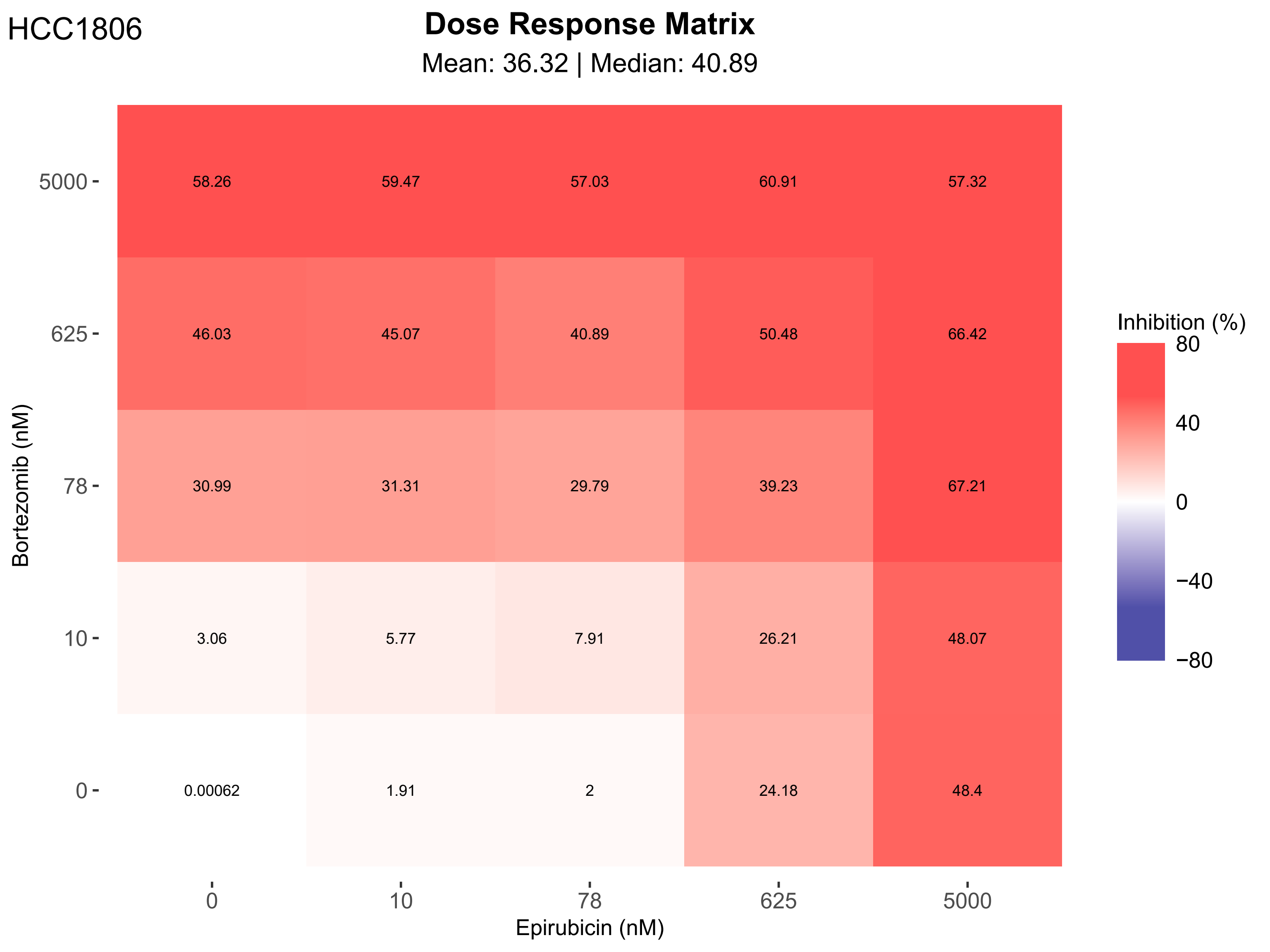
HCC1806
Dose Response Matrix
Mean: 36.32 | Median: 40.89
Bortezomib (nM)
5000
625
78
10
0
58.26
59.47
57.03
60.91
57.32
46.03
45.07
40.89
50.48
66.42
30.99
31.31
29.79
39.23
67.21
3.06
5.77
7.91
26.21
48.07
0.00062
1.91
2
24.18
48.4
0
10
78
625
5000
Epirubicin (nM)
Inhibition (%)
80
40
0
−40
−80

HCC1806

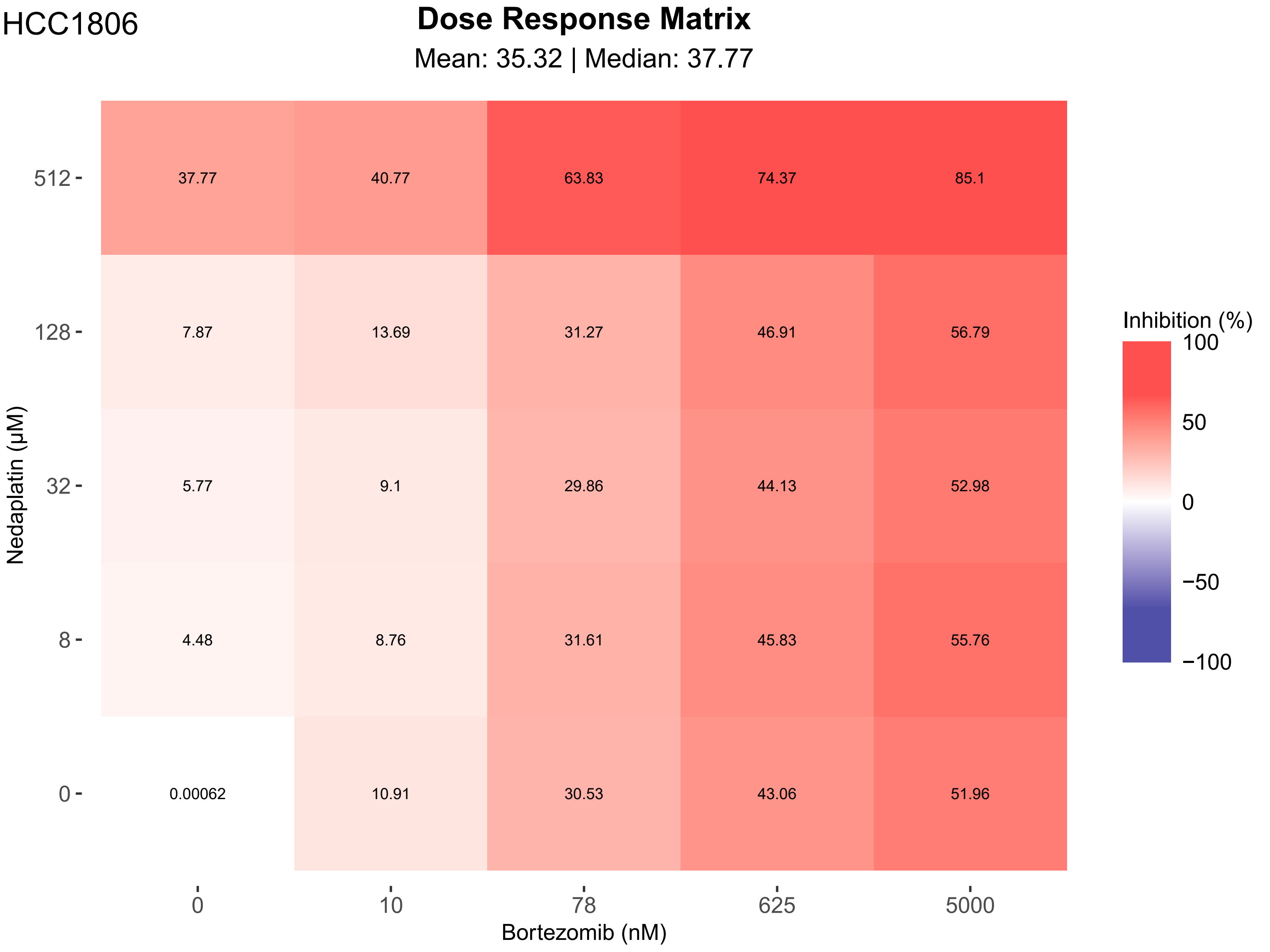
Dose Response Matrix
Mean: 35.32 | Median: 37.77
Nedaplatin (µM)
512
128
32
8
0
37.77
40.77
63.83
74.37
85.1
7.87
13.69
31.27
46.91
56.79
5.77
9.1
29.86
44.13
52.98
4.48
8.76
31.61
45.83
55.76
0.00062
10.91
30.53
43.06
51.96
0
10
78
625
5000
Bortezomib (nM)
Inhibition (%)
100
50
0
−50
−100

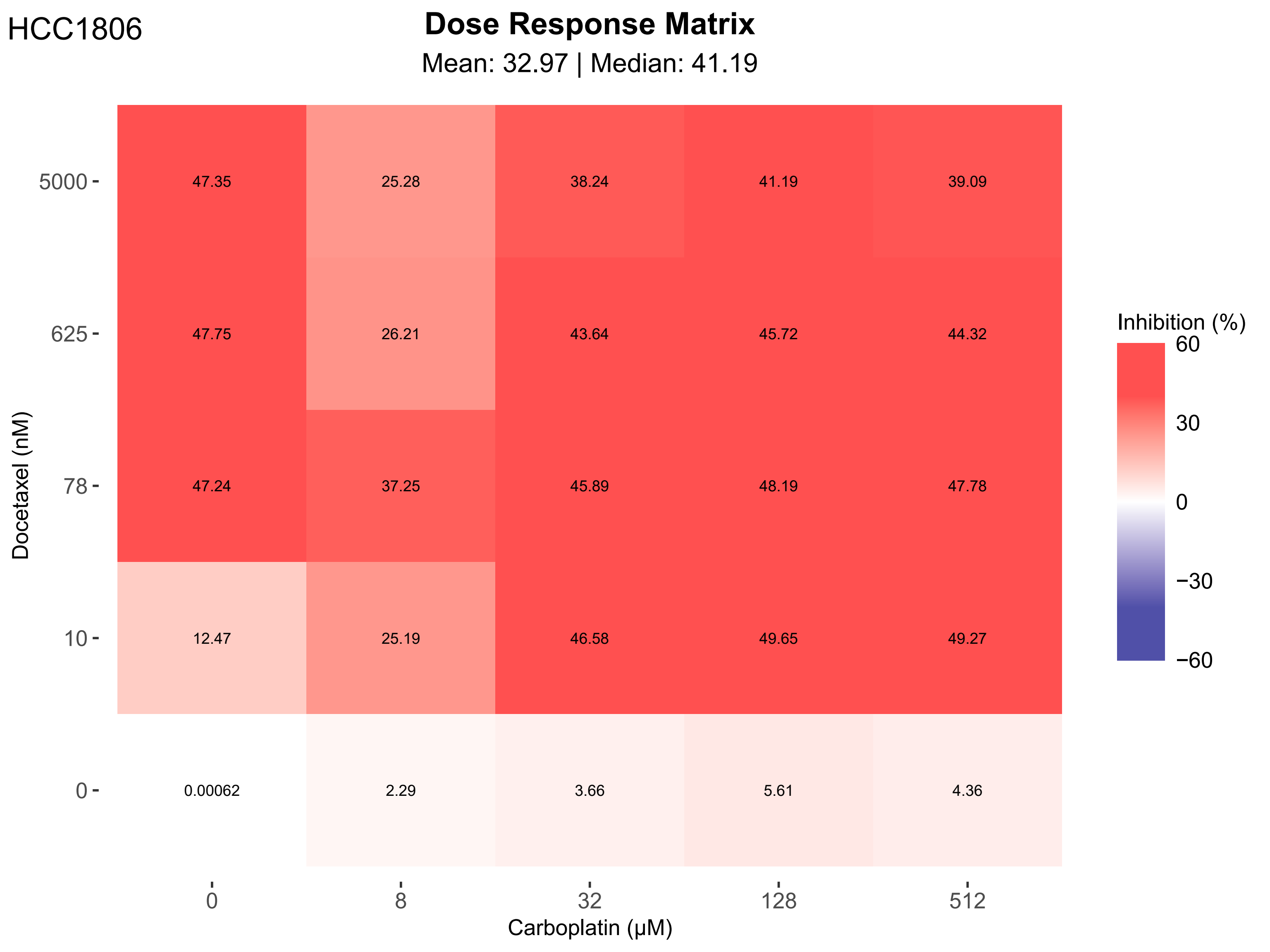

HCC1806
Dose Response Matrix
Mean: 32.97 | Median: 41.19
Docetaxel (nM)
5000
625
78
10
0
47.35
25.28
38.24
41.19
39.09
47.75
26.21
43.64
45.72
44.32
47.24
37.25
45.89
48.19
47.78
12.47
25.19
46.58
49.65
49.27
0.00062
2.29
3.66
5.61
4.36
0
8
32
128
512
Carboplatin (µM)
Inhibition (%)
60
30
0
−30
−60

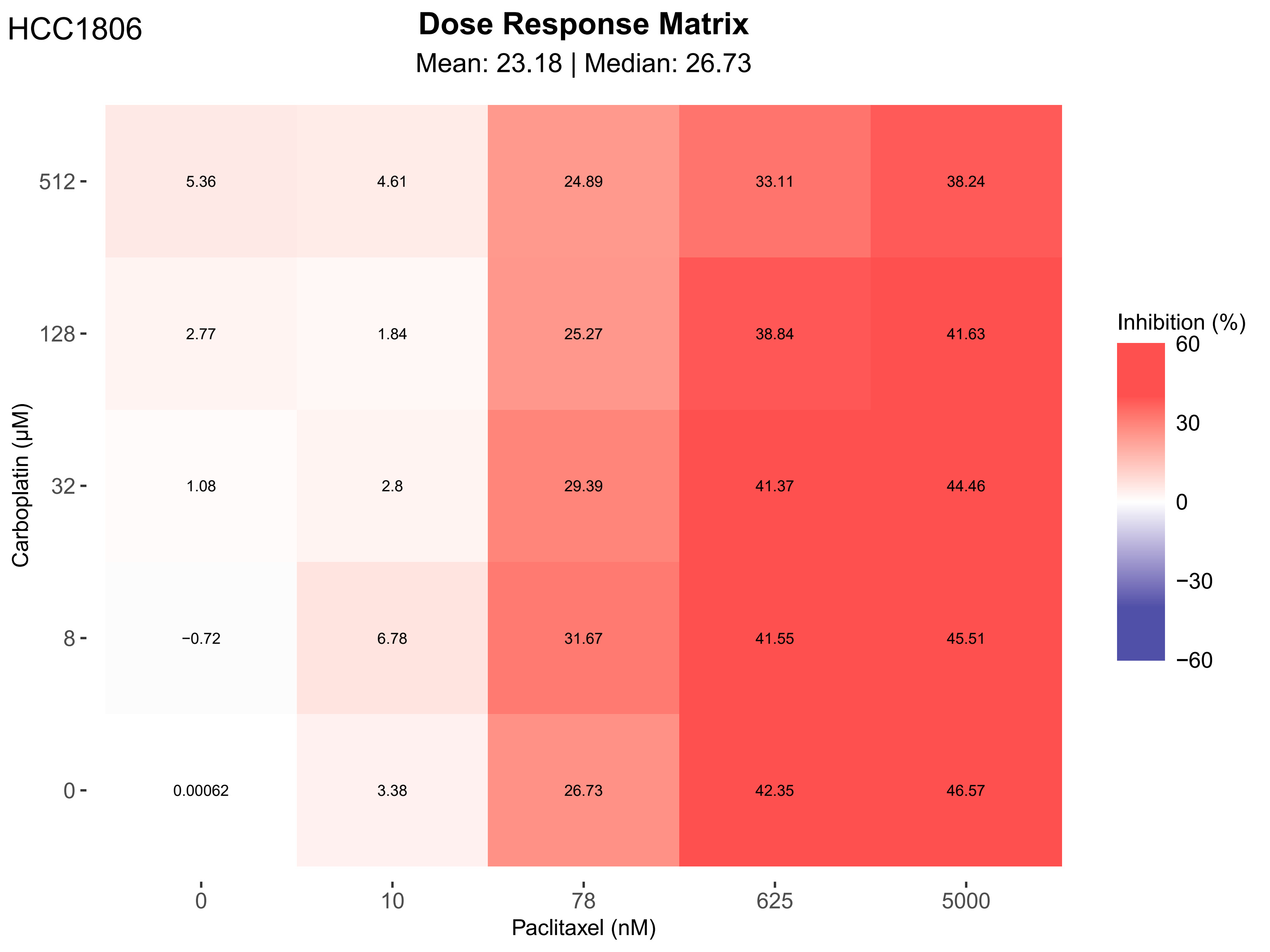
HCC1806
Dose Response Matrix
Mean: 23.18 | Median: 26.73
512
128
32
8
0
Carboplatin (µM)
0
10
78
625
5000
Paclitaxel (nM)
5.36
4.61
24.89
33.11
38.24
2.77
1.84
25.27
38.84
41.63
1.08
2.8
29.39
41.37
44.46
−0.72
6.78
31.67
41.55
45.51
0.00062
3.38
26.73
42.35
46.57
Inhibition (%)
60
30
0
−30
−60

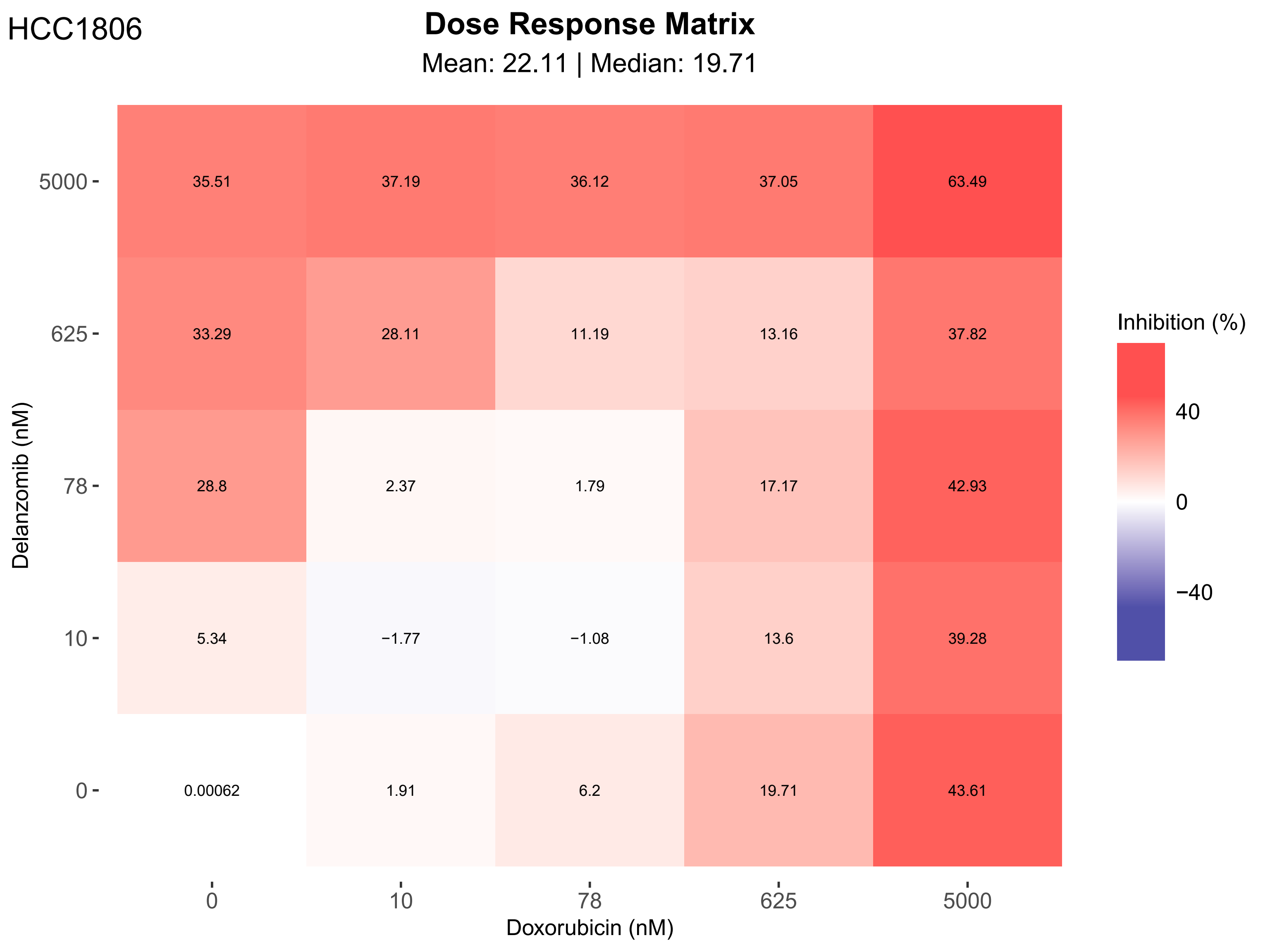
HCC1806
Dose Response Matrix
Mean: 22.11 | Median: 19.71
Delanzomib (nM)
5000
625
78
10
0
35.51
37.19
36.12
37.05
63.49
33.29
28.11
11.19
13.16
37.82
28.8
2.37
1.79
17.17
42.93
5.34
−1.77
−1.08
13.6
39.28
0.00062
1.91
6.2
19.71
43.61
0
10
78
625
5000
Doxorubicin (nM)
Inhibition (%)
40
0
−40

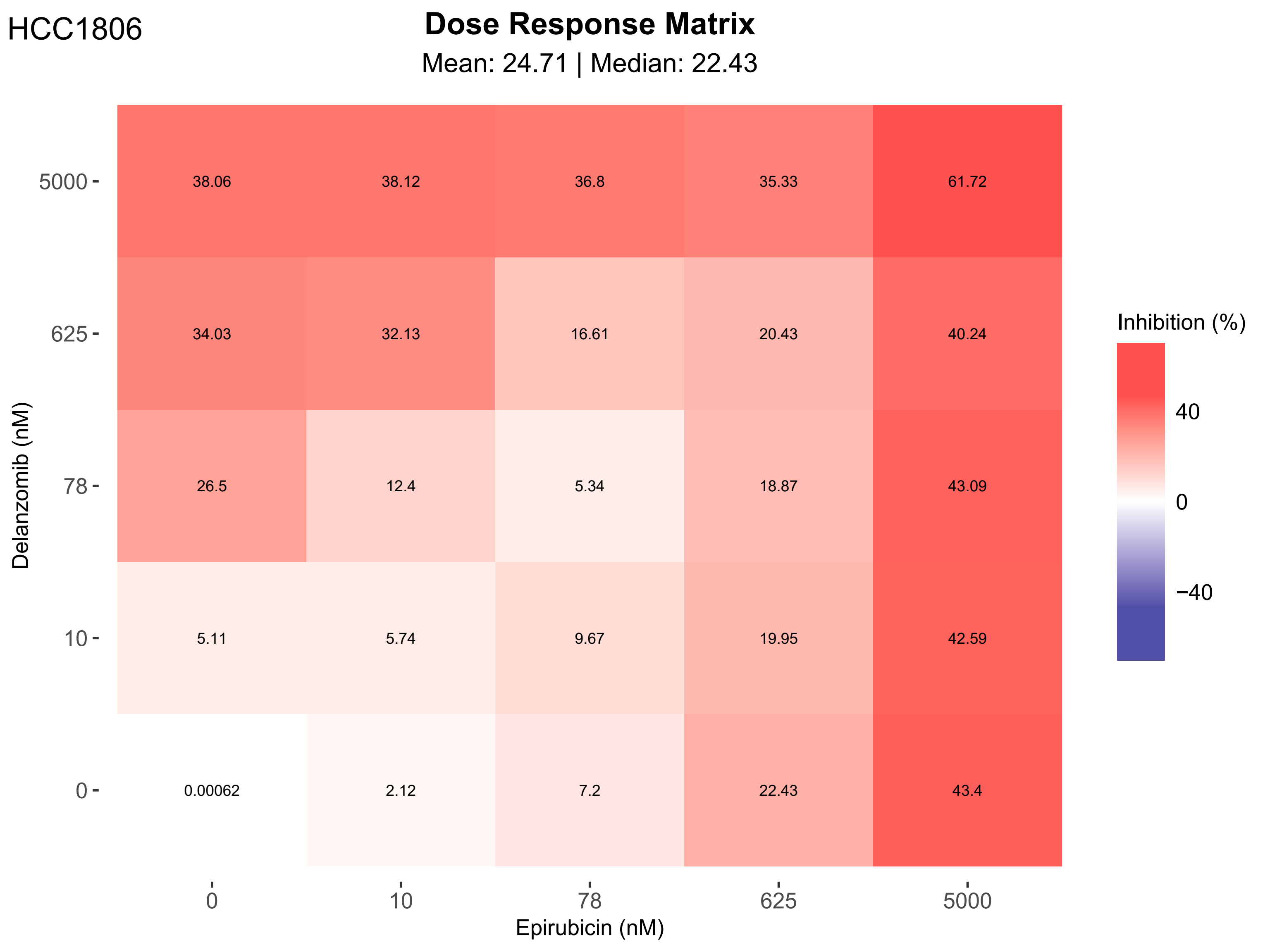
HCC1806
Dose Response Matrix
Mean: 24.71 | Median: 22.43
Delanzomib (nM)
5000
625
78
10
0
38.06
38.12
36.8
35.33
61.72
34.03
32.13
16.61
20.43
40.24
26.5
12.4
5.34
18.87
43.09
5.11
5.74
9.67
19.95
42.59
0.00062
2.12
7.2
22.43
43.4
0
10
78
625
5000
Epirubicin (nM)
Inhibition (%)
40
0
−40

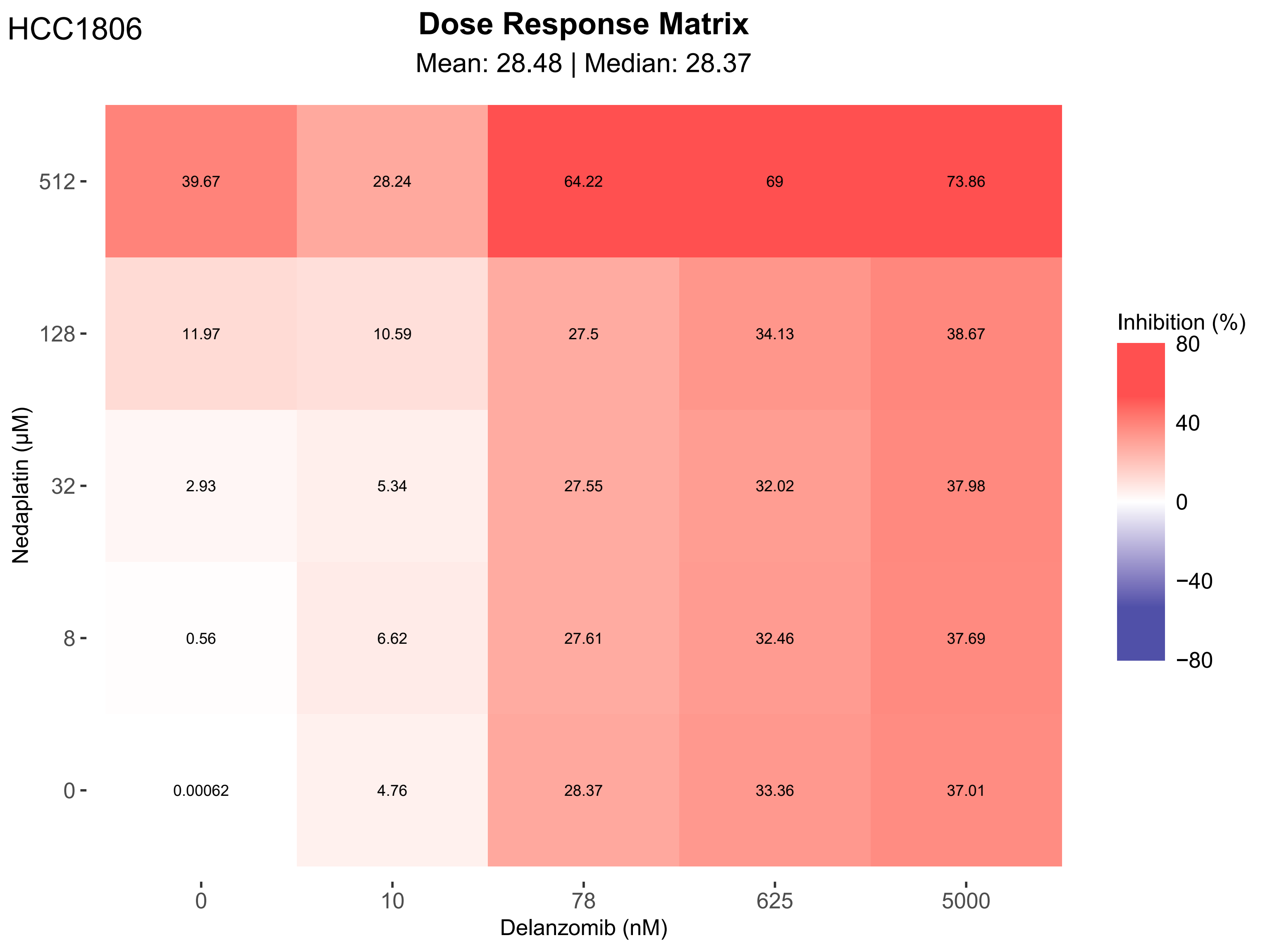

HCC1806
Dose Response Matrix
Mean: 28.48 | Median: 28.37
512
128
32
8
0
39.67
28.24
64.22
69
73.86
11.97
10.59
27.5
34.13
38.67
2.93
5.34
27.55
32.02
37.98
0.56
6.62
27.61
32.46
37.69
0.00062
4.76
28.37
33.36
37.01
Nedaplatin (μM)
0
10
78
625
5000
Delanzomib (nM)
Inhibition (%)
80
40
0
−40
−80

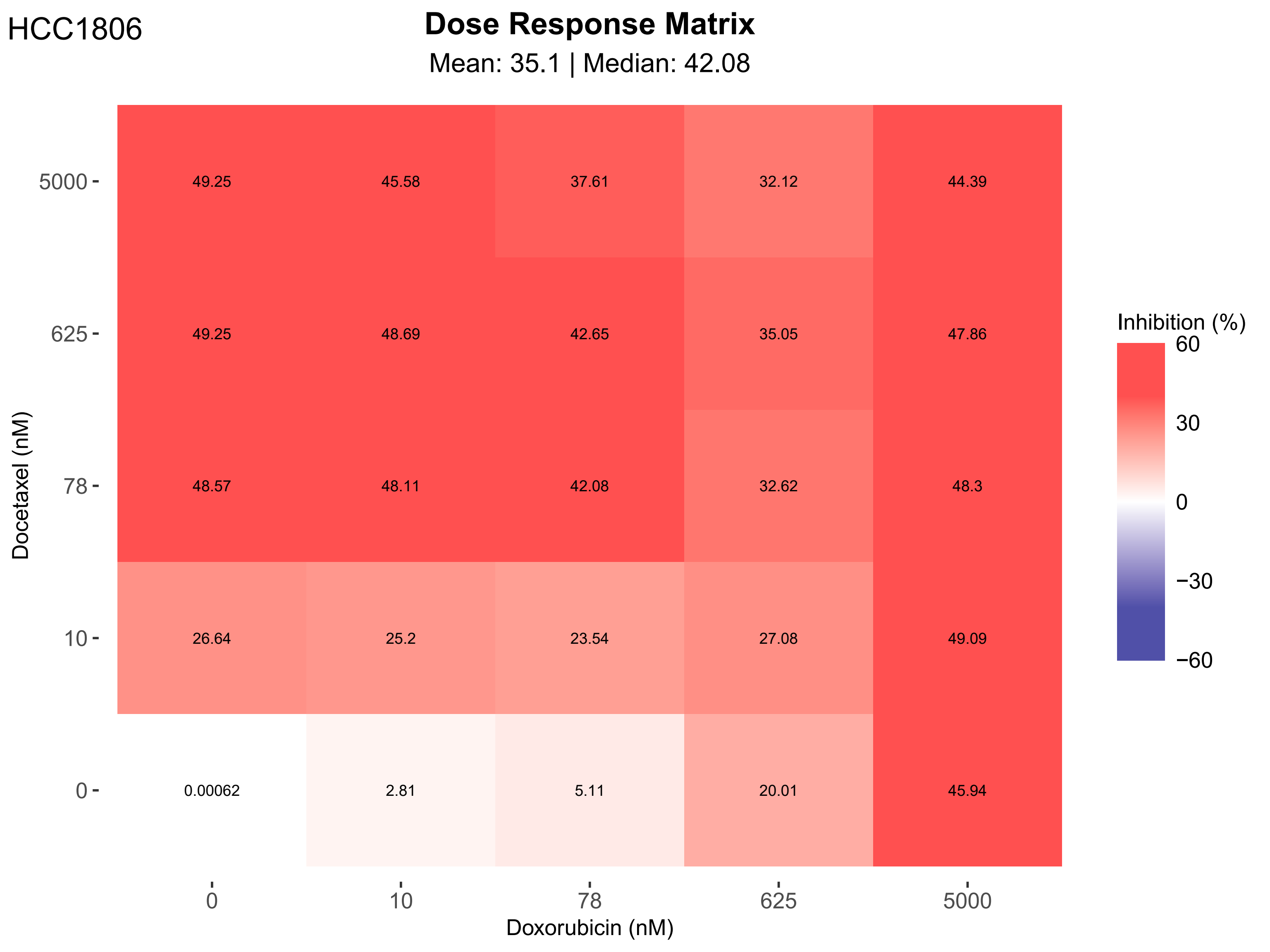
HCC1806
Dose Response Matrix
Mean: 35.1 | Median: 42.08
49.25
45.58
37.61
32.12
44.39
49.25
48.69
42.65
35.05
47.86
48.57
48.11
42.08
32.62
48.3
26.64
25.2
23.54
27.08
49.09
0.00062
2.81
5.11
20.01
45.94
5000
625
78
10
0
Docetaxel (nM)
0
10
78
625
5000
Doxorubicin (nM)
Inhibition (%)
60
30
0
−30
−60

HCC1806

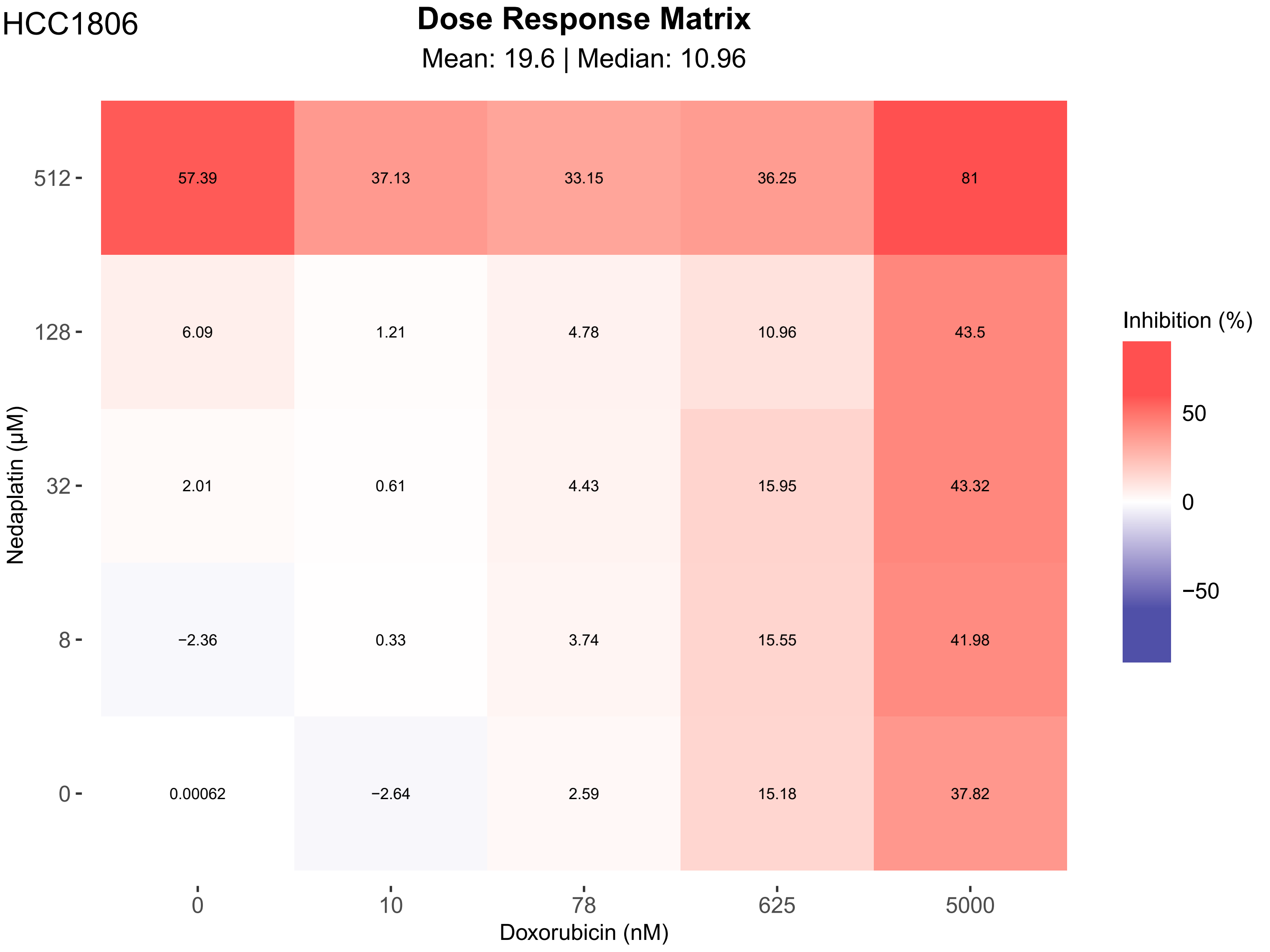

Dose Response Matrix
Mean: 19.6 | Median: 10.96
512
128
32
8
0
Nedaplatin (μM)
57.39
37.13
33.15
36.25
81
6.09
1.21
4.78
10.96
43.5
2.01
0.61
4.43
15.95
43.32
−2.36
0.33
3.74
15.55
41.98
0.00062
−2.64
2.59
15.18
37.82
0
10
78
625
5000
Doxorubicin (nM)
Inhibition (%)
50
0
−50

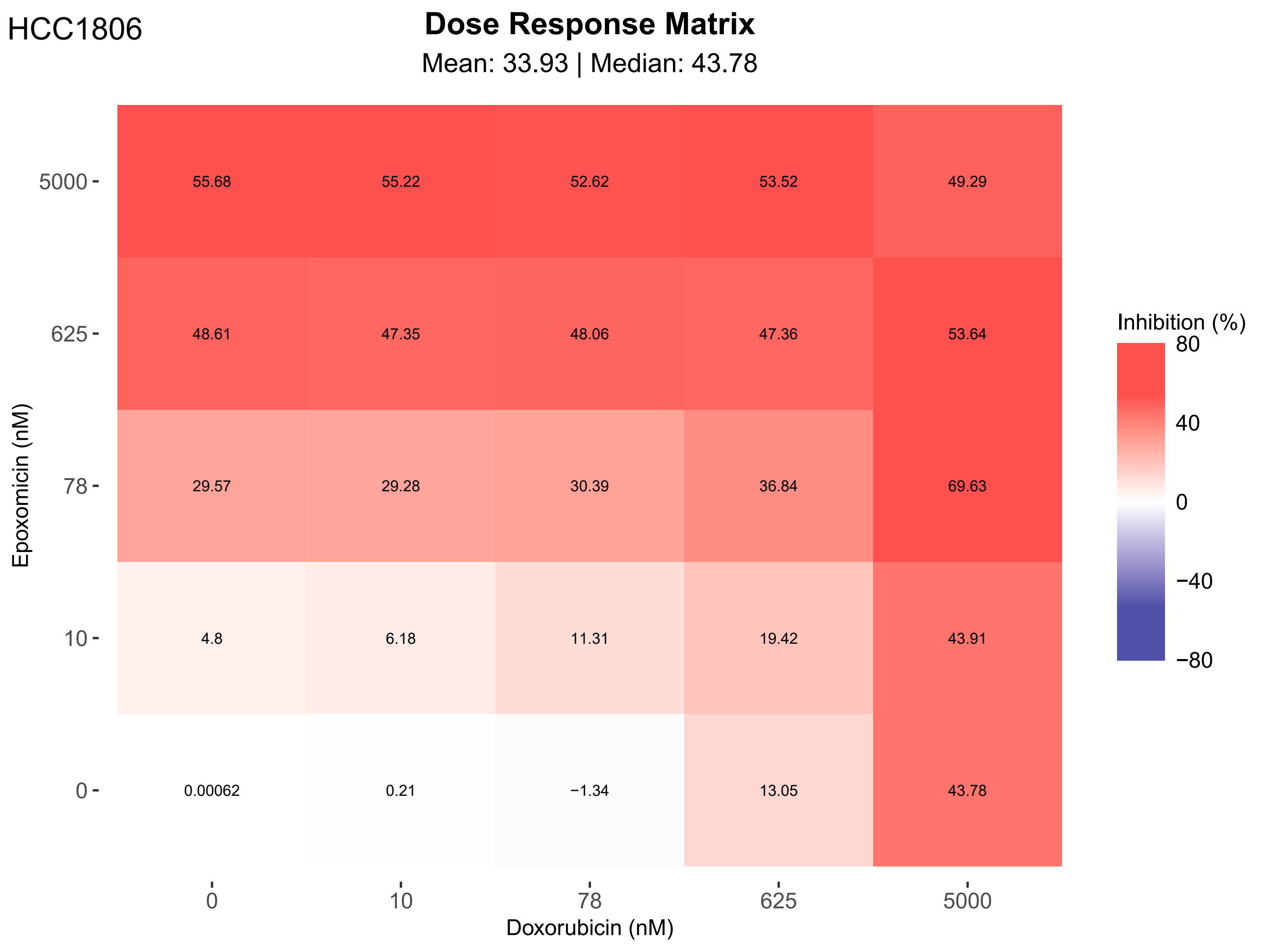
HCC1806
Dose Response Matrix
Mean: 33.93 | Median: 43.78
5000
625
78
10
0
Epoxomicin (nM)
55.68
55.22
52.62
53.52
49.29
48.61
47.35
48.06
47.36
53.64
29.57
29.28
30.39
36.84
69.63
4.8
6.18
11.31
19.42
43.91
0.00062
0.21
−1.34
13.05
43.78
0
10
78
625
5000
Doxorubicin (nM)
Inhibition (%)
80
40
0
−40
−80

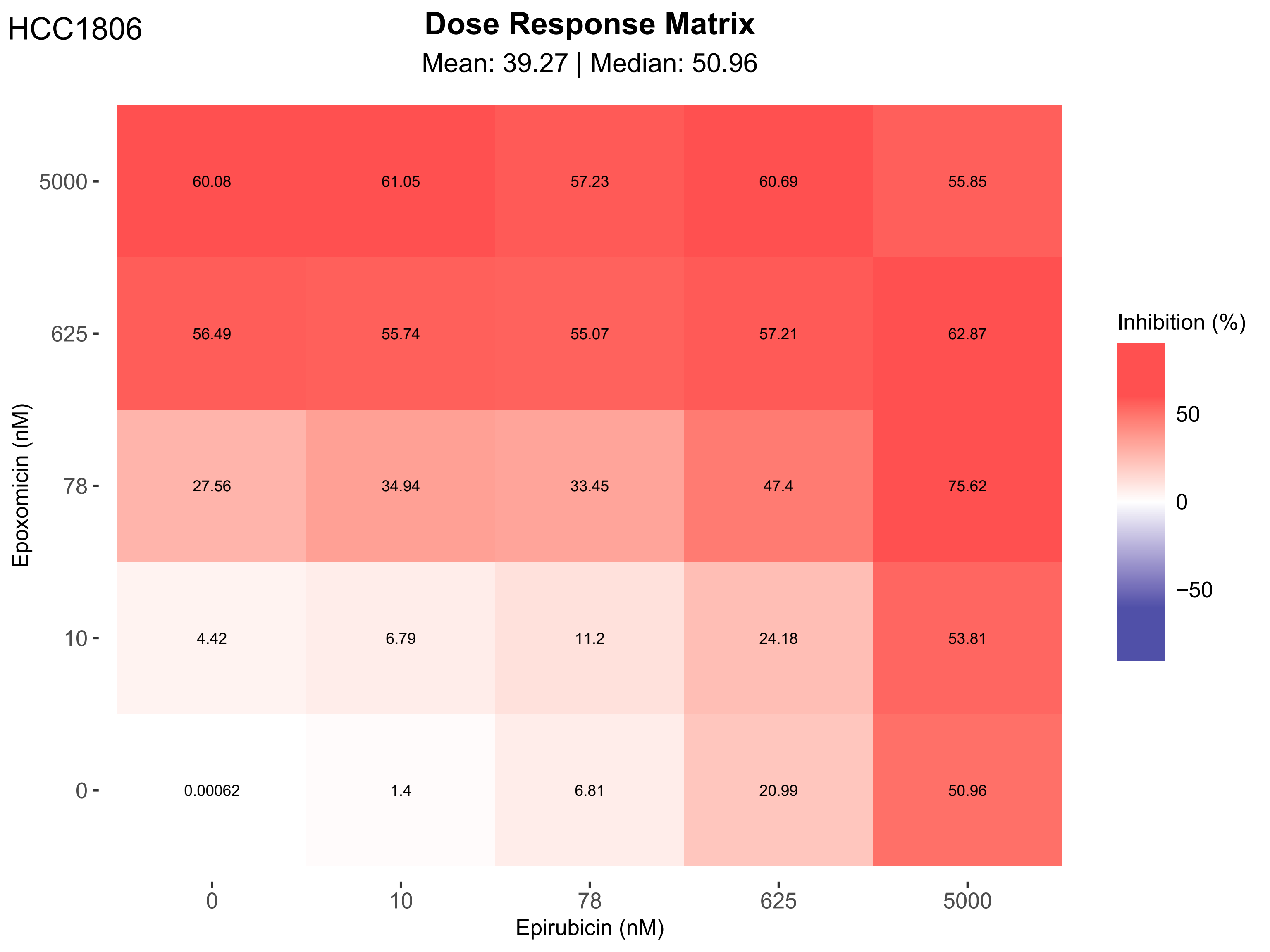
HCC1806
Dose Response Matrix
Mean: 39.27 | Median: 50.96
Epoxomicin (nM)
5000
625
78
10
0
Epirubicin (nM)
0
10
78
625
5000
60.08
61.05
57.23
60.69
55.85
56.49
55.74
55.07
57.21
62.87
27.56
34.94
33.45
47.4
75.62
4.42
6.79
11.2
24.18
53.81
0.00062
1.4
6.81
20.99
50.96
Inhibition (%)
50
0
−50

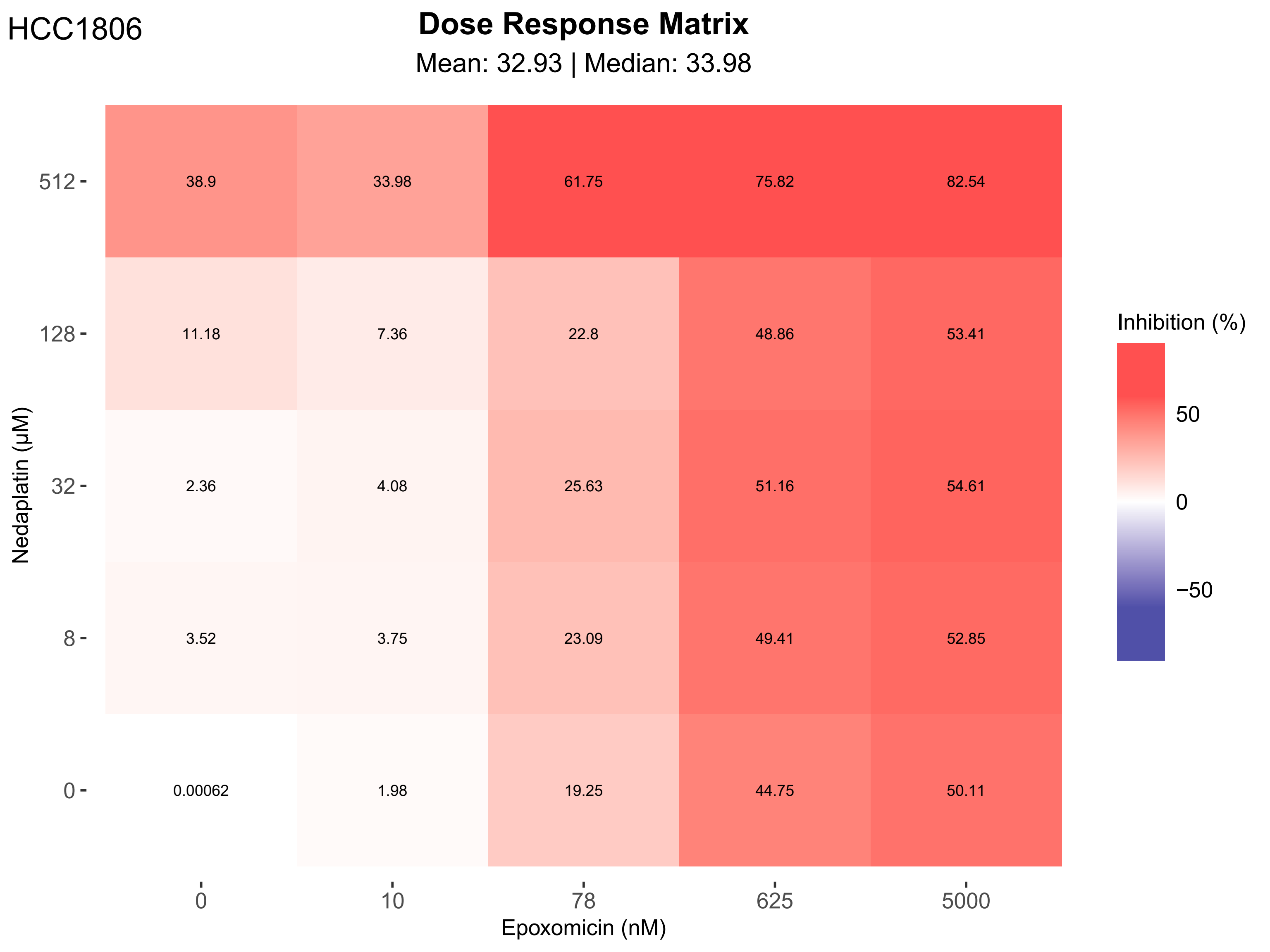
HCC1806
Dose Response Matrix
Mean: 32.93 | Median: 33.98
Nedaplatin (µM)
512
128
32
8
0
Epoxomicin (nM)
0
10
78
625
5000
38.9
33.98
61.75
75.82
82.54
11.18
7.36
22.8
48.86
53.41
2.36
4.08
25.63
51.16
54.61
3.52
3.75
23.09
49.41
52.85
0.00062
1.98
19.25
44.75
50.11
Inhibition (%)
50
0
−50

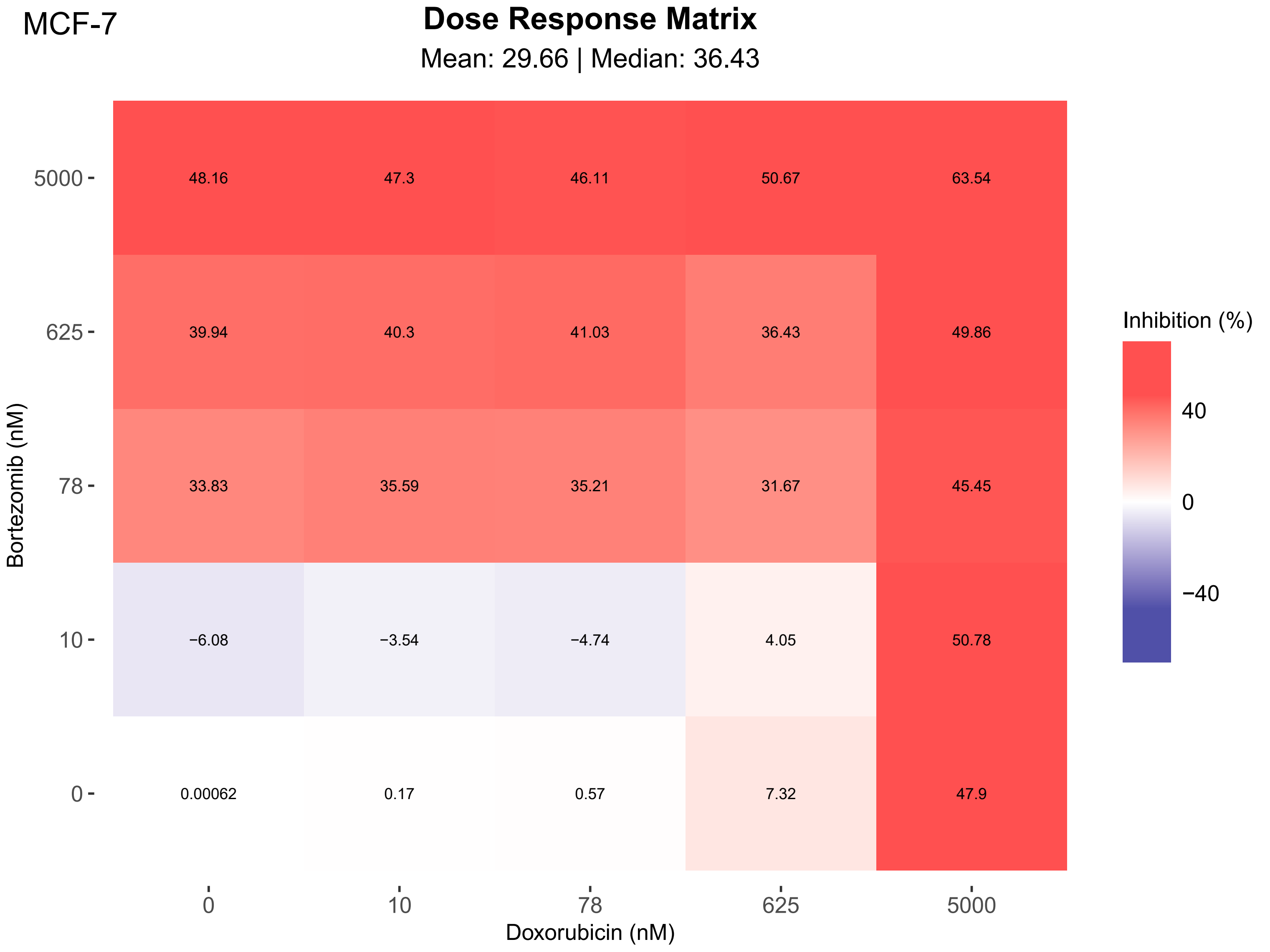
MCF-7
Dose Response Matrix
Mean: 29.66 | Median: 36.43
Bortezomib (nM)
5000
625
78
10
0
48.16
47.3
46.11
50.67
63.54
39.94
40.3
41.03
36.43
49.86
33.83
35.59
35.21
31.67
45.45
−6.08
−3.54
−4.74
4.05
50.78
0.00062
0.17
0.57
7.32
47.9
0
10
78
625
5000
Doxorubicin (nM)
Inhibition (%)
40
0
−40

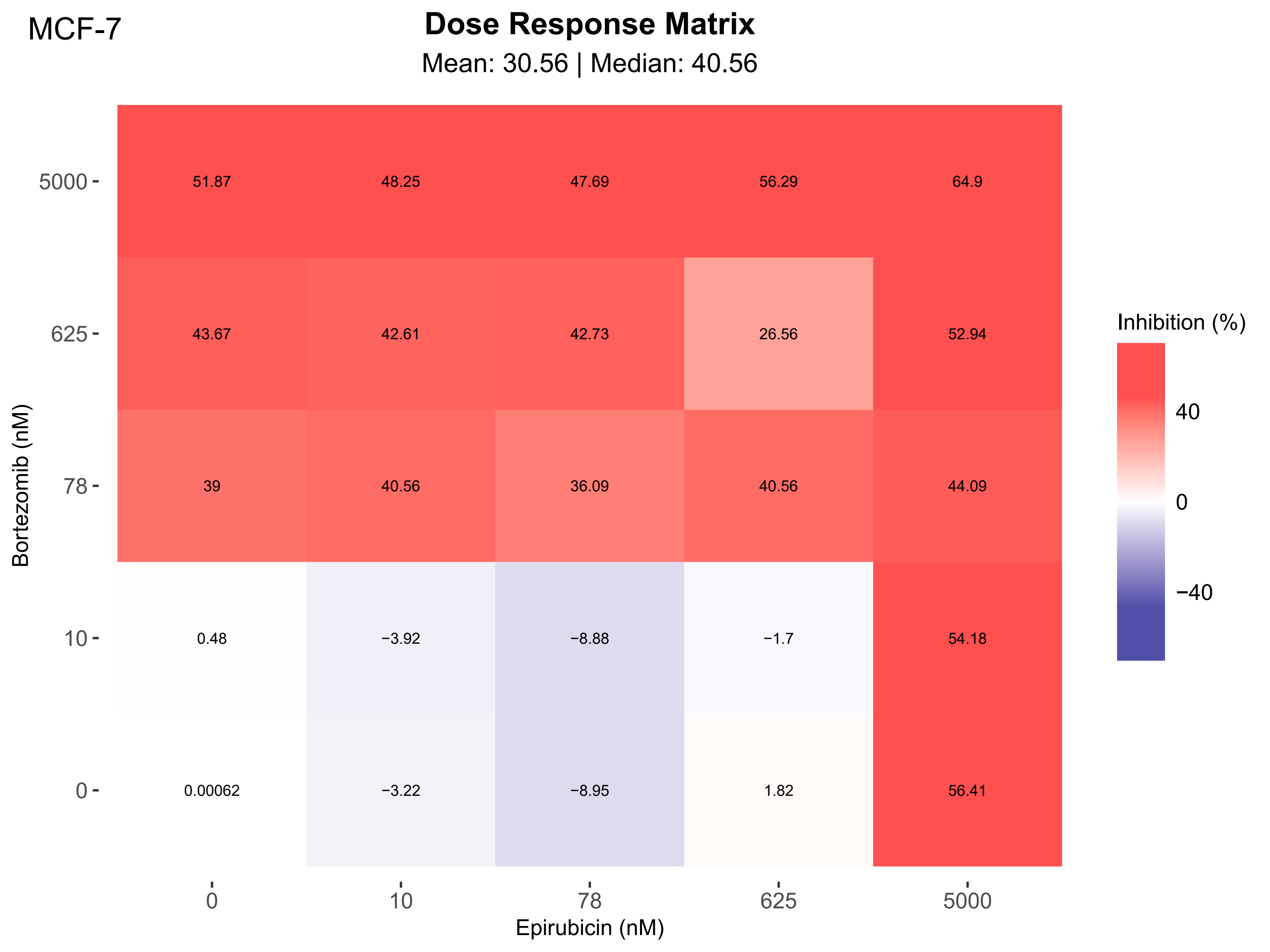
MCF-7
Dose Response Matrix
Mean: 30.56 | Median: 40.56
5000
625
78
10
0
Bortezomib (nM)
51.87
48.25
47.69
56.29
64.9
43.67
42.61
42.73
26.56
52.94
39
40.56
36.09
40.56
44.09
0.48
−3.92
−8.88
−1.7
54.18
0.00062
−3.22
−8.95
1.82
56.41
0
10
78
625
5000
Epirubicin (nM)
Inhibition (%)
40
0
−40

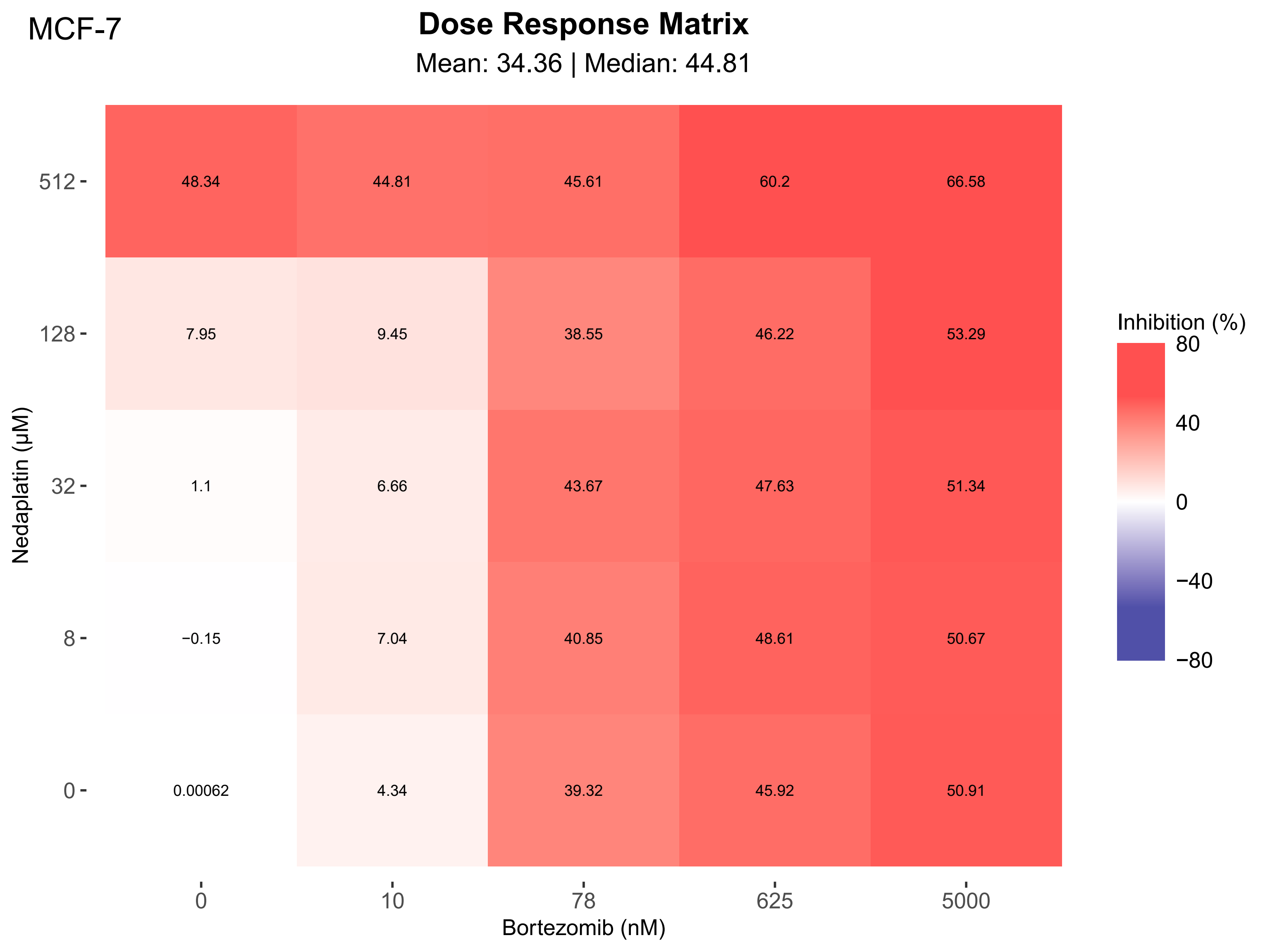
MCF-7
Dose Response Matrix
Mean: 34.36 | Median: 44.81
Nedaplatin (μM)
512
128
32
8
0
48.34
44.81
45.61
60.2
66.58
7.95
9.45
38.55
46.22
53.29
1.1
6.66
43.67
47.63
51.34
−0.15
7.04
40.85
48.61
50.67
0.00062
4.34
39.32
45.92
50.91
0
10
78
625
5000
Bortezomib (nM)
Inhibition (%)
80
40
0
−40
−80

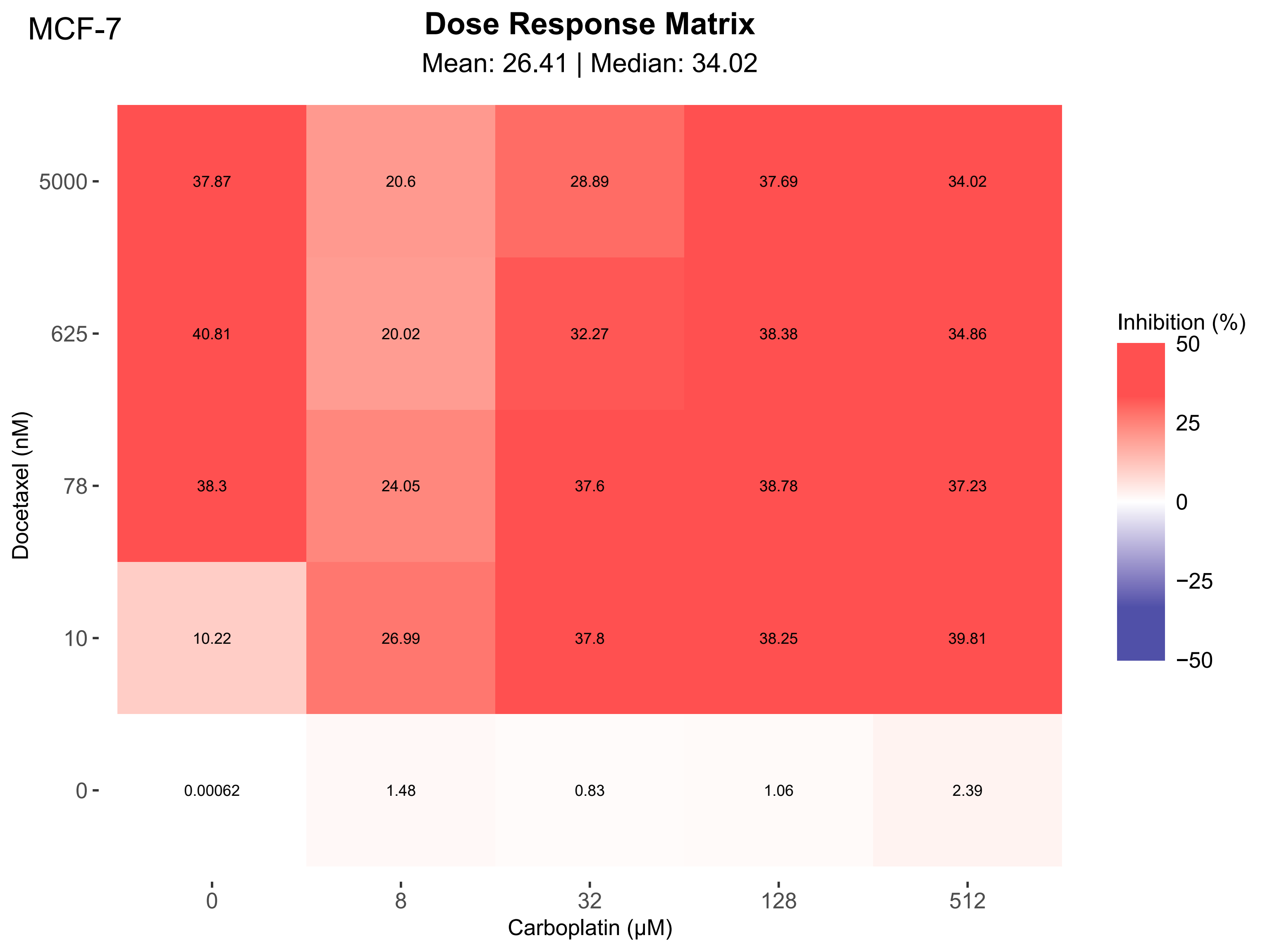
MCF-7
Dose Response Matrix
Mean: 26.41 | Median: 34.02
Docetaxel (nM)
5000
625
78
10
0
37.87
20.6
28.89
37.69
34.02
40.81
20.02
32.27
38.38
34.86
38.3
24.05
37.6
38.78
37.23
10.22
26.99
37.8
38.25
39.81
0.00062
1.48
0.83
1.06
2.39
0
8
32
128
512
Carboplatin (μM)
Inhibition (%)
50
25
0
−25
−50

MCF-7

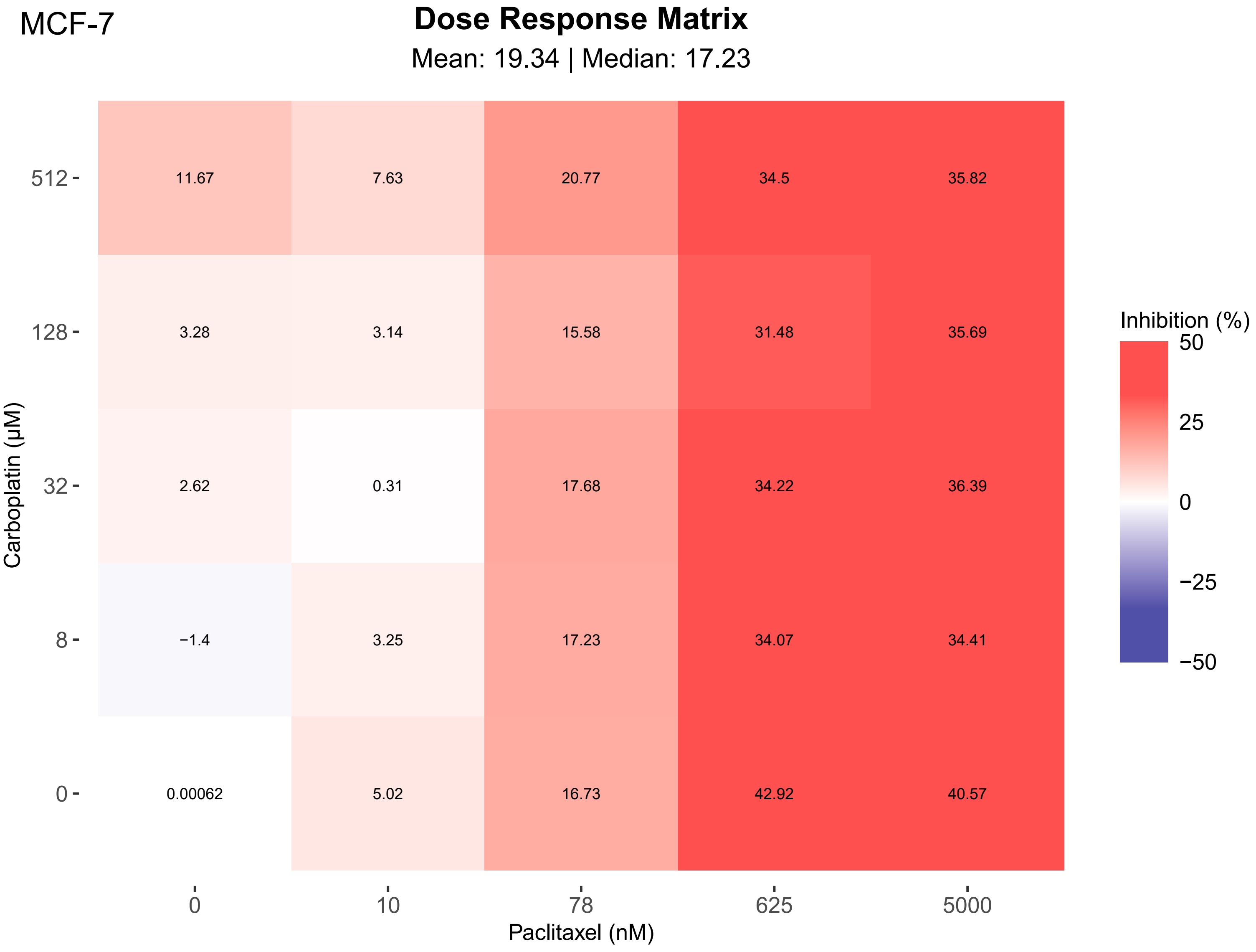

Dose Response Matrix
Mean: 19.34 | Median: 17.23
512
128
32
8
0
Carboplatin (µM)
0
10
78
625
5000
Paclitaxel (nM)
11.67
7.63
20.77
34.5
35.82
3.28
3.14
15.58
31.48
35.69
2.62
0.31
17.68
34.22
36.39
−1.4
3.25
17.23
34.07
34.41
0.00062
5.02
16.73
42.92
40.57
Inhibition (%)
50
25
0
−25
−50

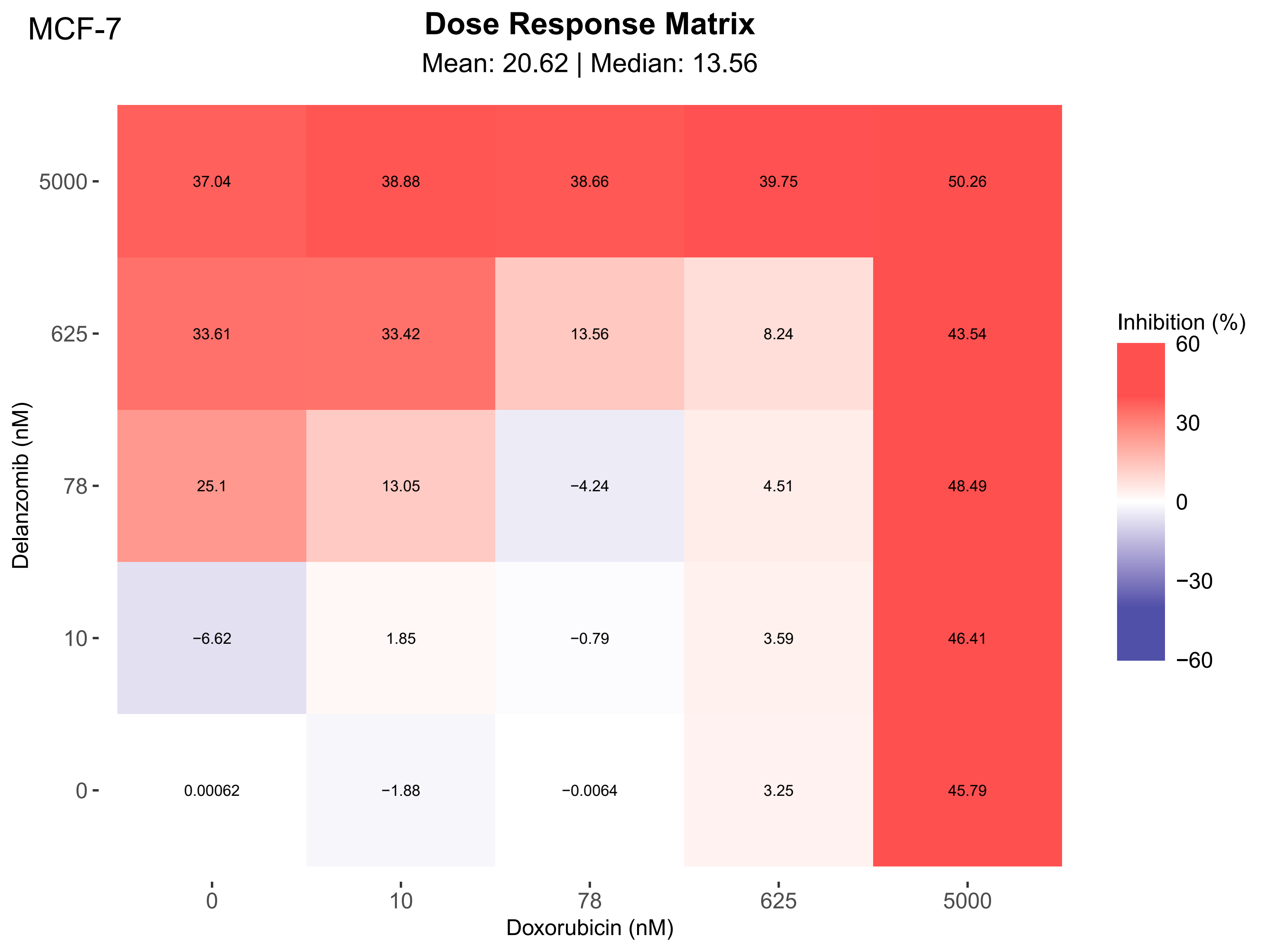
MCF-7
Dose Response Matrix
Mean: 20.62 | Median: 13.56
Delanzomib (nM)
5000
625
78
10
0
Doxorubicin (nM)
0
10
78
625
5000
37.04
38.88
38.66
39.75
50.26
33.61
33.42
13.56
8.24
43.54
25.1
13.05
−4.24
4.51
48.49
−6.62
1.85
−0.79
3.59
46.41
0.00062
−1.88
−0.0064
3.25
45.79
Inhibition (%)
60
30
0
−30
−60

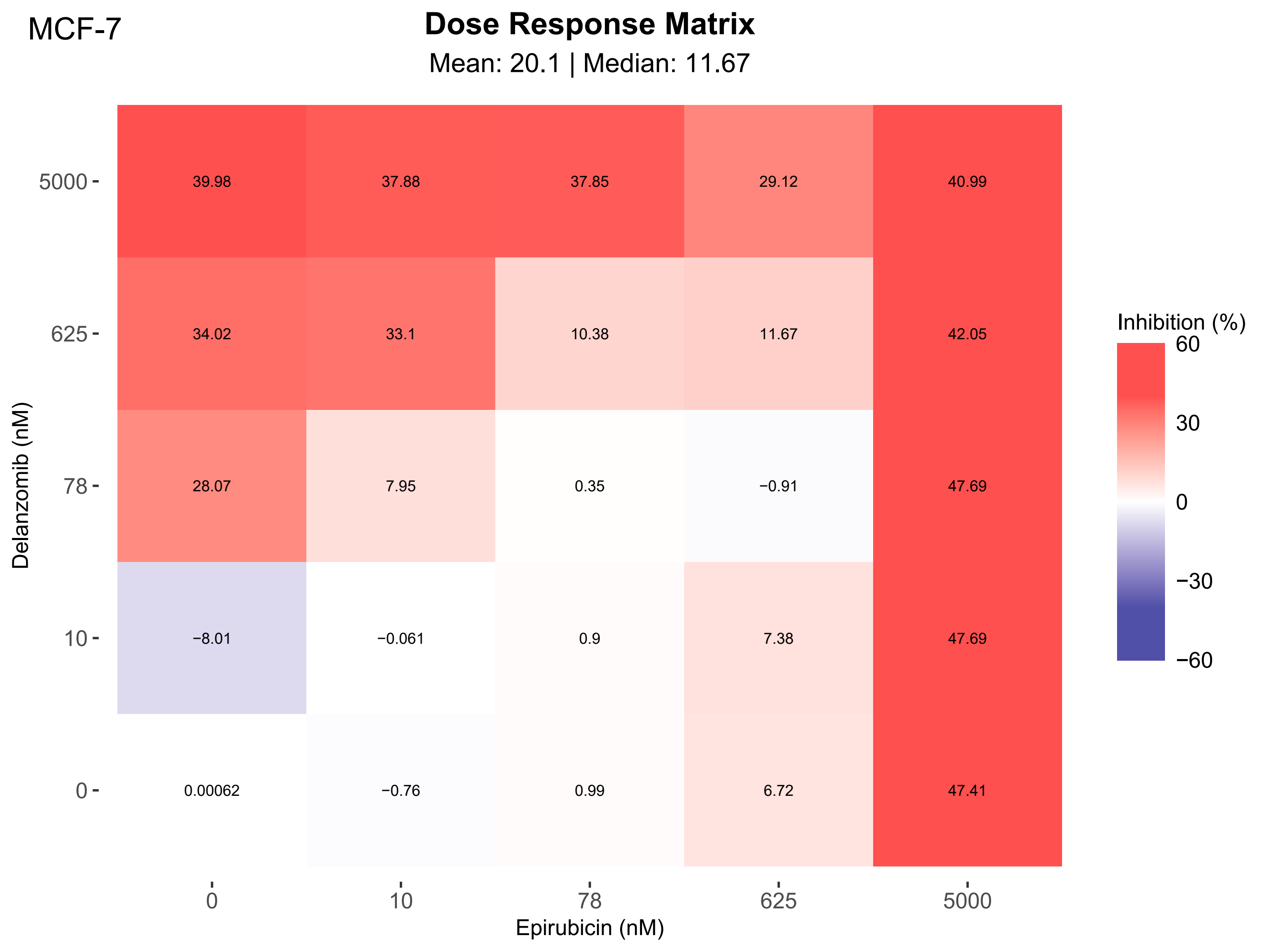
MCF-7
Dose Response Matrix
Mean: 20.1 | Median: 11.67
Delanzomib (nM)
5000
625
78
10
0
39.98
37.88
37.85
29.12
40.99
34.02
33.1
10.38
11.67
42.05
28.07
7.95
0.35
−0.91
47.69
−8.01
−0.061
0.9
7.38
47.69
0.00062
−0.76
0.99
6.72
47.41
0
10
78
625
5000
Epirubicin (nM)
Inhibition (%)
60
30
0
−30
−60

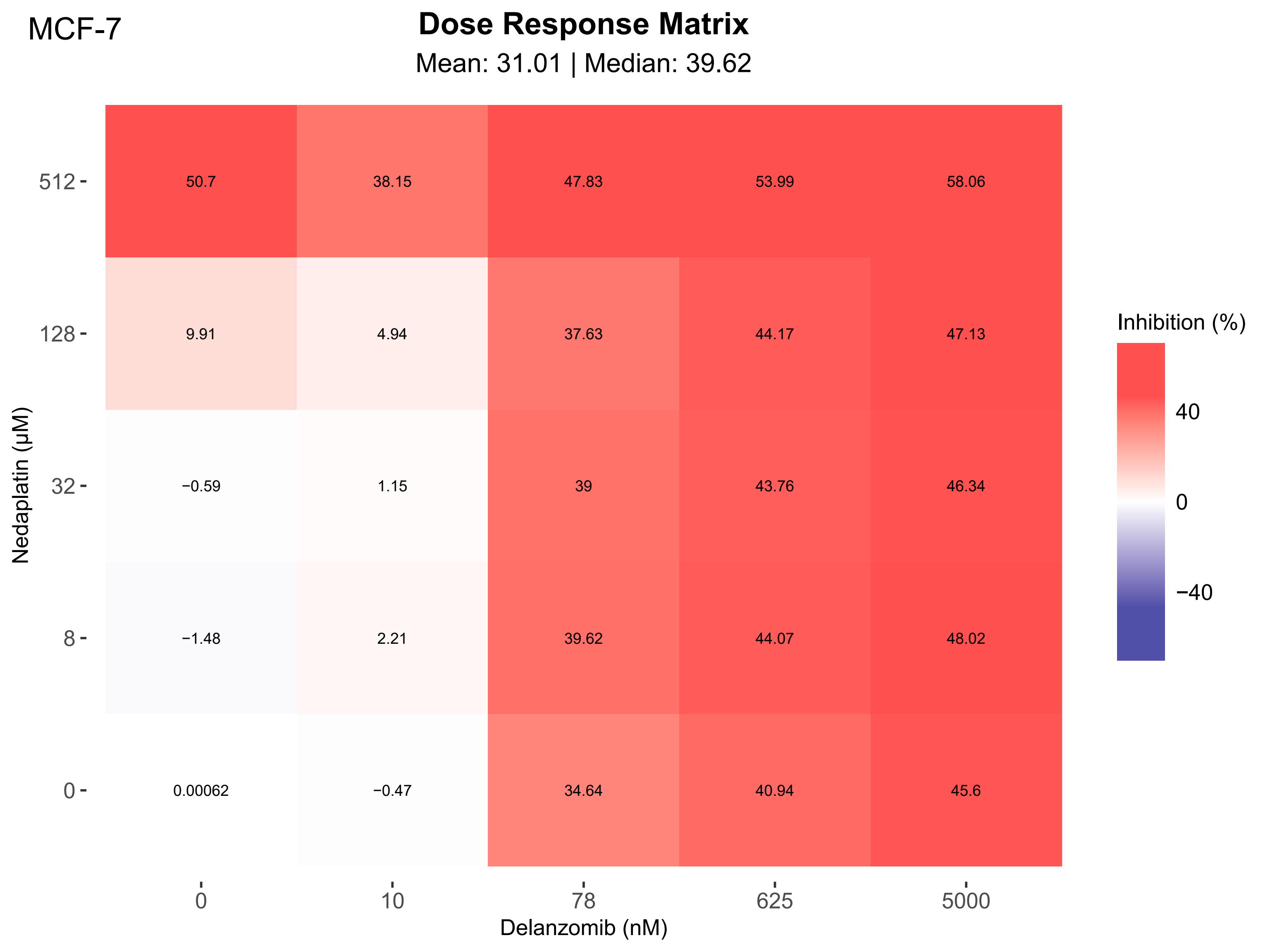
MCF-7
Dose Response Matrix
Mean: 31.01 | Median: 39.62
Nedaplatin (μM)
512
128
32
8
0
Delanzomib (nM)
0
10
78
625
5000
50.7
38.15
47.83
53.99
58.06
9.91
4.94
37.63
44.17
47.13
−0.59
1.15
39
43.76
46.34
−1.48
2.21
39.62
44.07
48.02
0.00062
−0.47
34.64
40.94
45.6
Inhibition (%)
40
0
−40

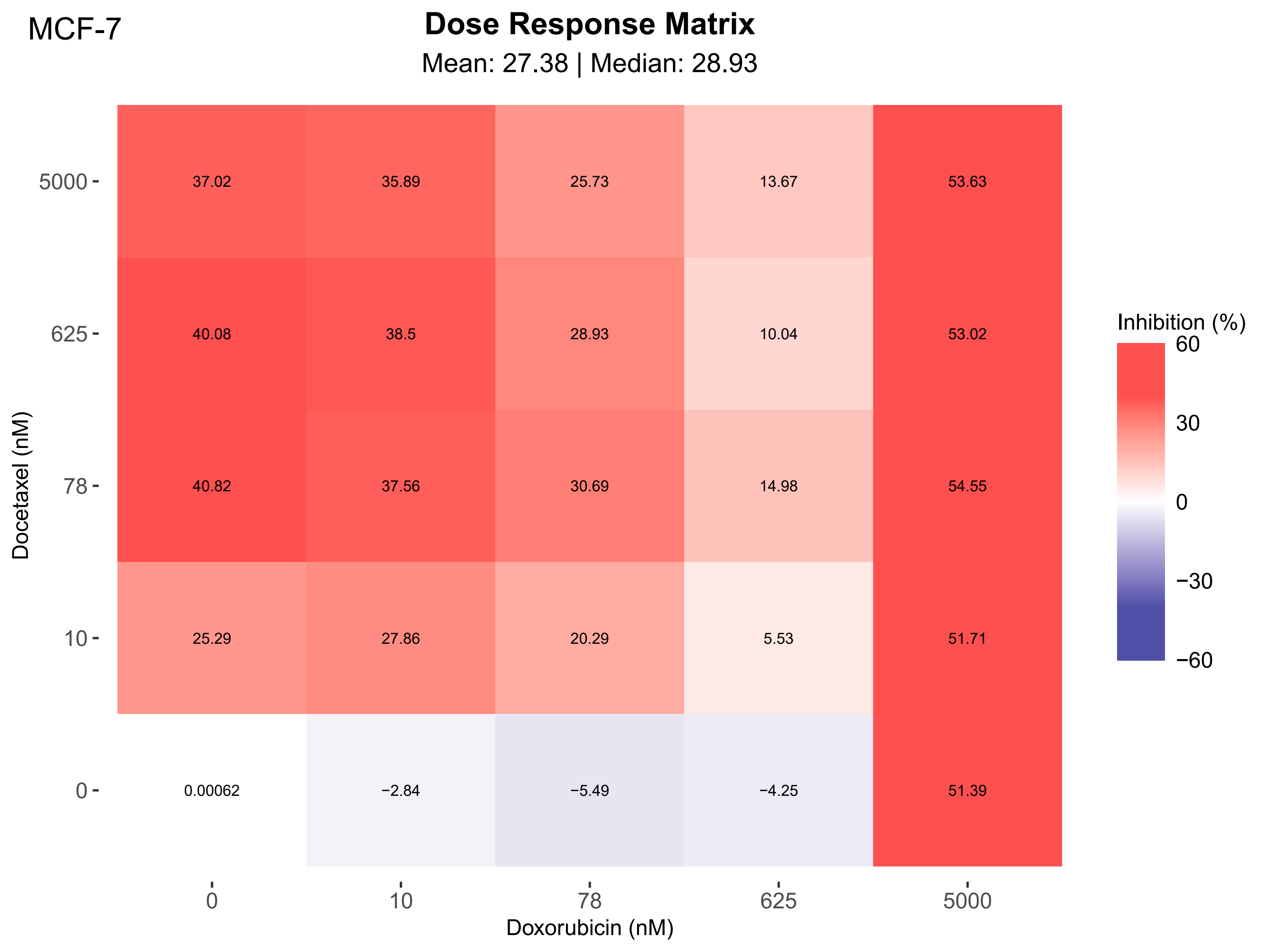
MCF-7
Dose Response Matrix
Mean: 27.38 | Median: 28.93
Docetaxel (nM)
5000
625
78
10
0
37.02
35.89
25.73
13.67
53.63
40.08
38.5
28.93
10.04
53.02
40.82
37.56
30.69
14.98
54.55
25.29
27.86
20.29
5.53
51.71
0.00062
−2.84
−5.49
−4.25
51.39
0
10
78
625
5000
Doxorubicin (nM)
Inhibition (%)
60
30
0
−30
−60

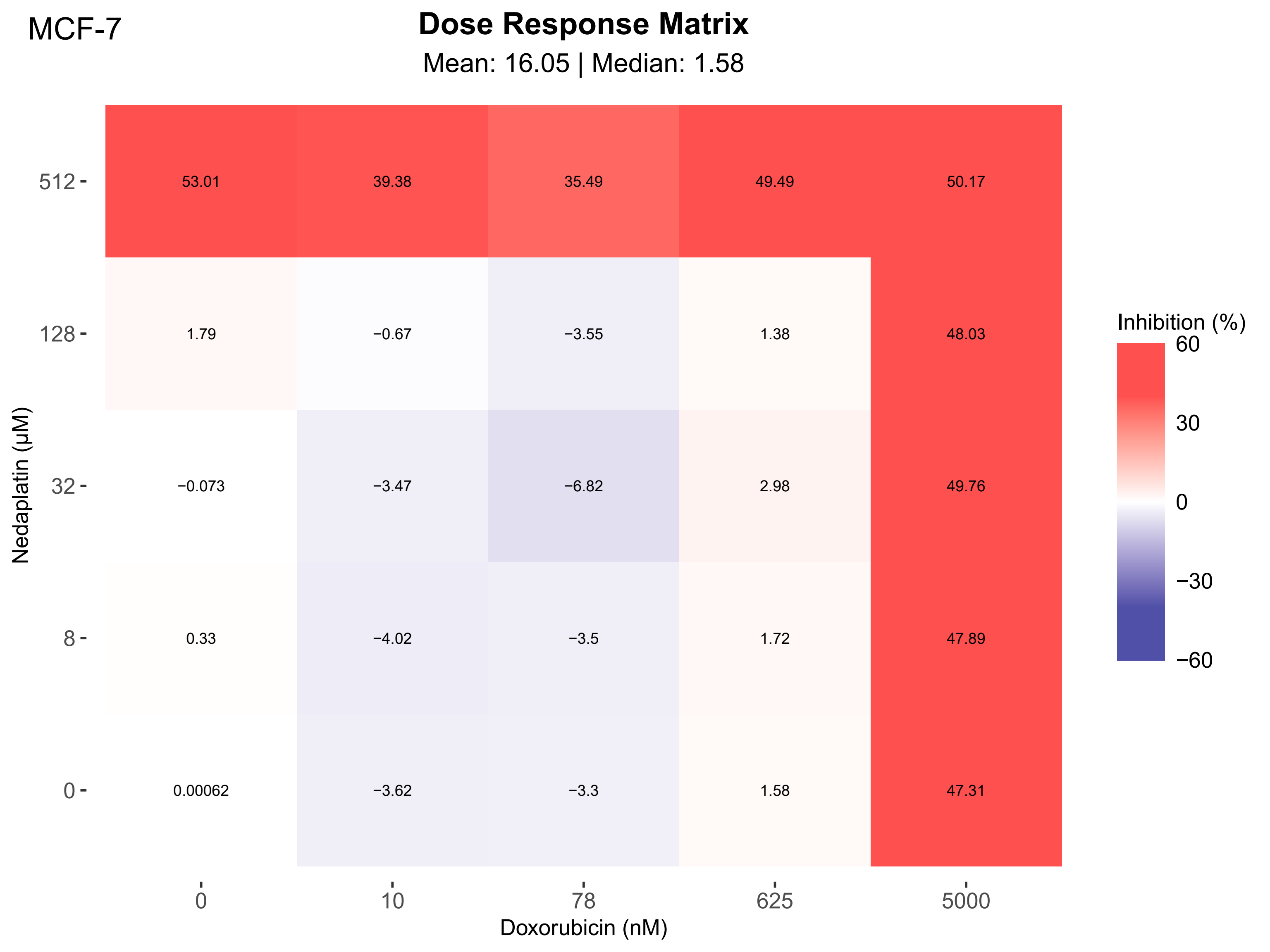

MCF-7
Dose Response Matrix
Mean: 16.05 | Median: 1.58
Nedaplatin (μM)
512
128
32
8
0
53.01
39.38
35.49
49.49
50.17
1.79
−0.67
−3.55
1.38
48.03
−0.073
−3.47
−6.82
2.98
49.76
0.33
−4.02
−3.5
1.72
47.89
0.00062
−3.62
−3.3
1.58
47.31
0
10
78
625
5000
Doxorubicin (nM)
Inhibition (%)
60
30
0
−30
−60

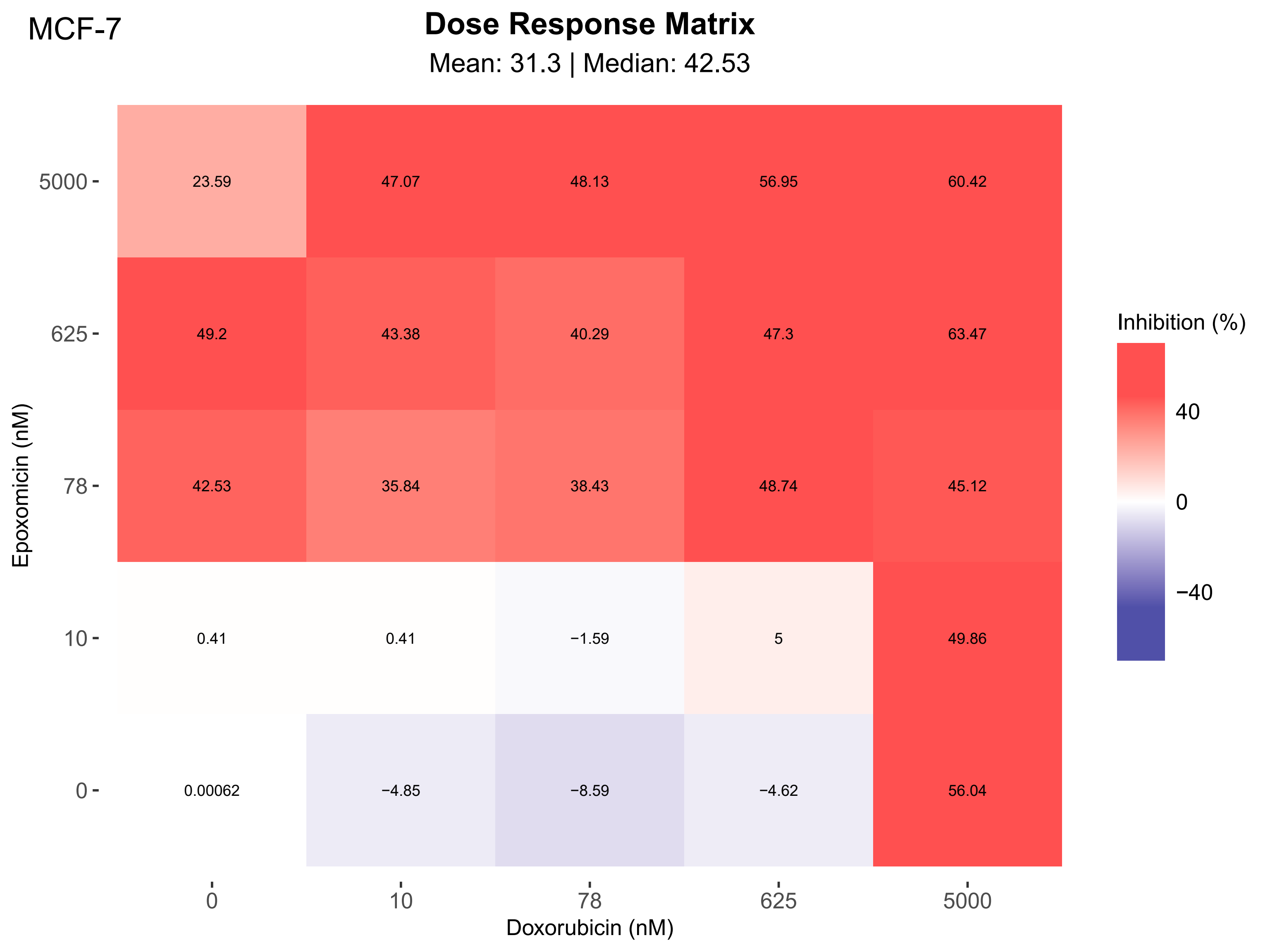

MCF-7
Dose Response Matrix
Mean: 31.3 | Median: 42.53
Epoxomicin (nM)
5000
625
78
10
0
23.59
47.07
48.13
56.95
60.42
49.2
43.38
40.29
47.3
63.47
42.53
35.84
38.43
48.74
45.12
0.41
0.41
−1.59
5
49.86
0.00062
−4.85
−8.59
−4.62
56.04
0
10
78
625
5000
Doxorubicin (nM)
Inhibition (%)
40
0
−40

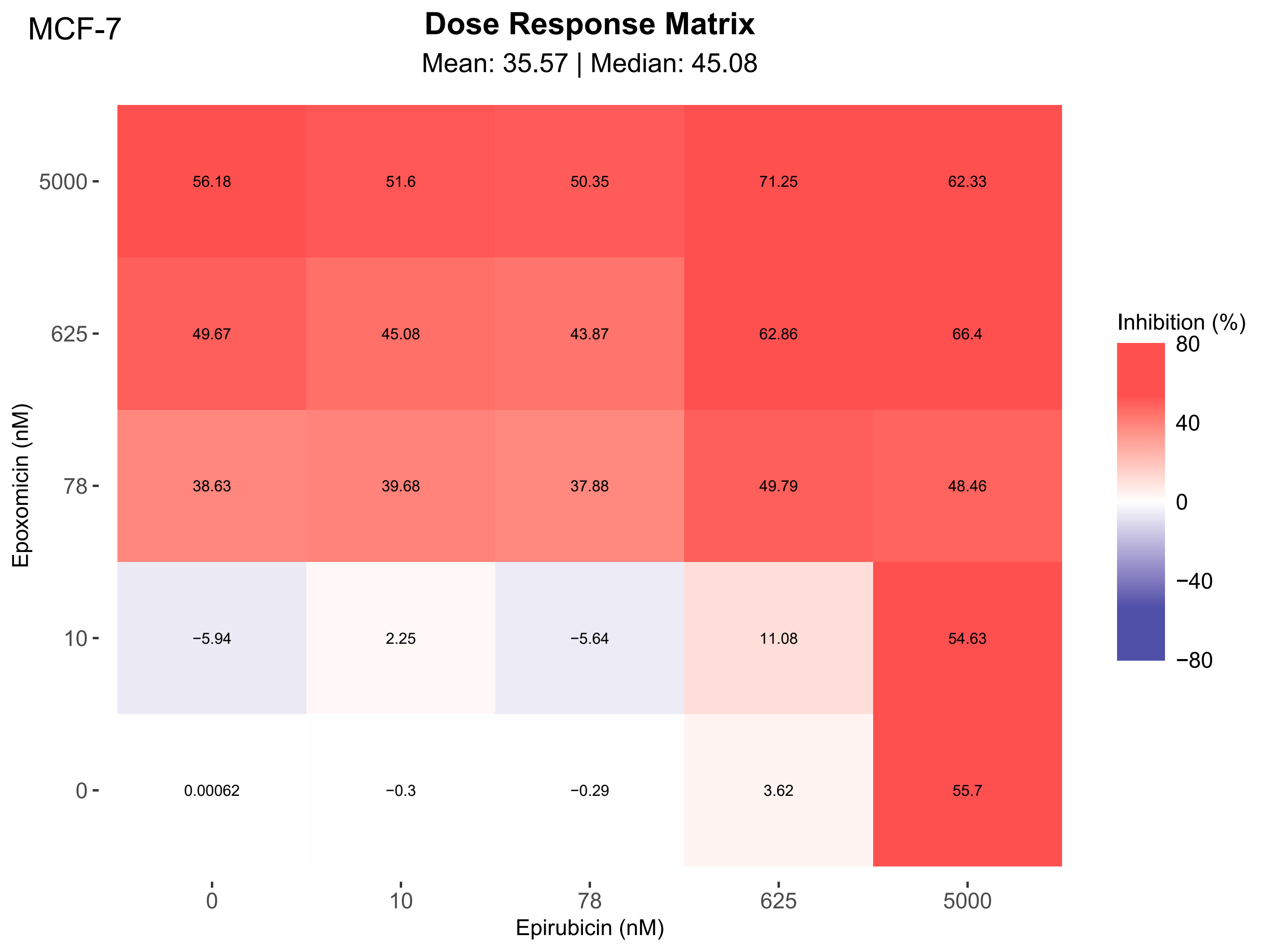
MCF-7
Dose Response Matrix
Mean: 35.57 | Median: 45.08
Epoxomicin (nM)
5000
625
78
10
0
56.18
51.6
50.35
71.25
62.33
49.67
45.08
43.87
62.86
66.4
38.63
39.68
37.88
49.79
48.46
−5.94
2.25
−5.64
11.08
54.63
0.00062
−0.3
−0.29
3.62
55.7
0
10
78
625
5000
Epirubicin (nM)
Inhibition (%)
80
40
0
−40
−80

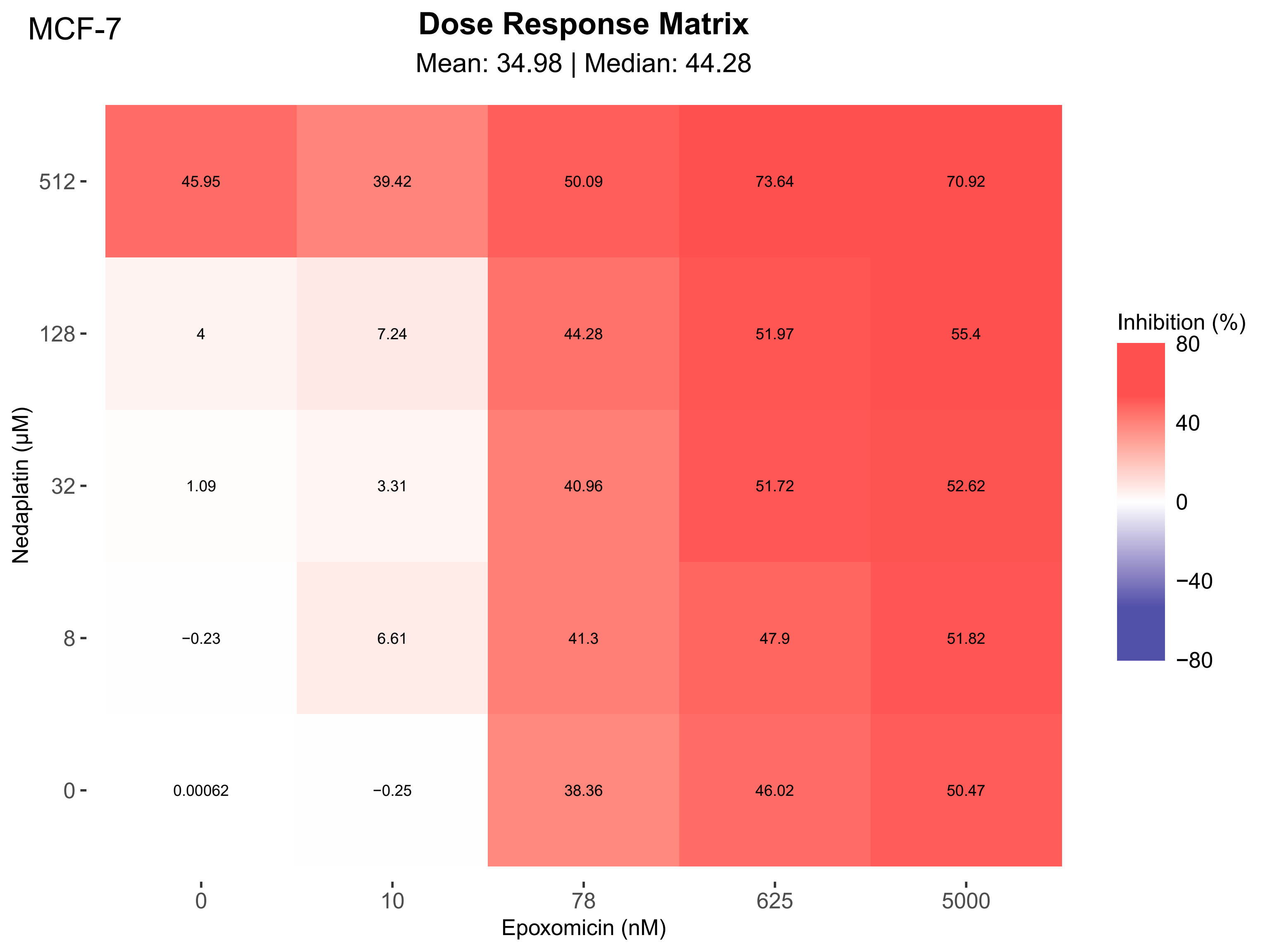

MCF-7
Dose Response Matrix
Mean: 34.98 | Median: 44.28
Nedaplatin (µM)
512
128
32
8
0
45.95
39.42
50.09
73.64
70.92
4
7.24
44.28
51.97
55.4
1.09
3.31
40.96
51.72
52.62
−0.23
6.61
41.3
47.9
51.82
0.00062
−0.25
38.36
46.02
50.47
0
10
78
625
5000
Epoxomicin (nM)
Inhibition (%)
80
40
0
−40
−80

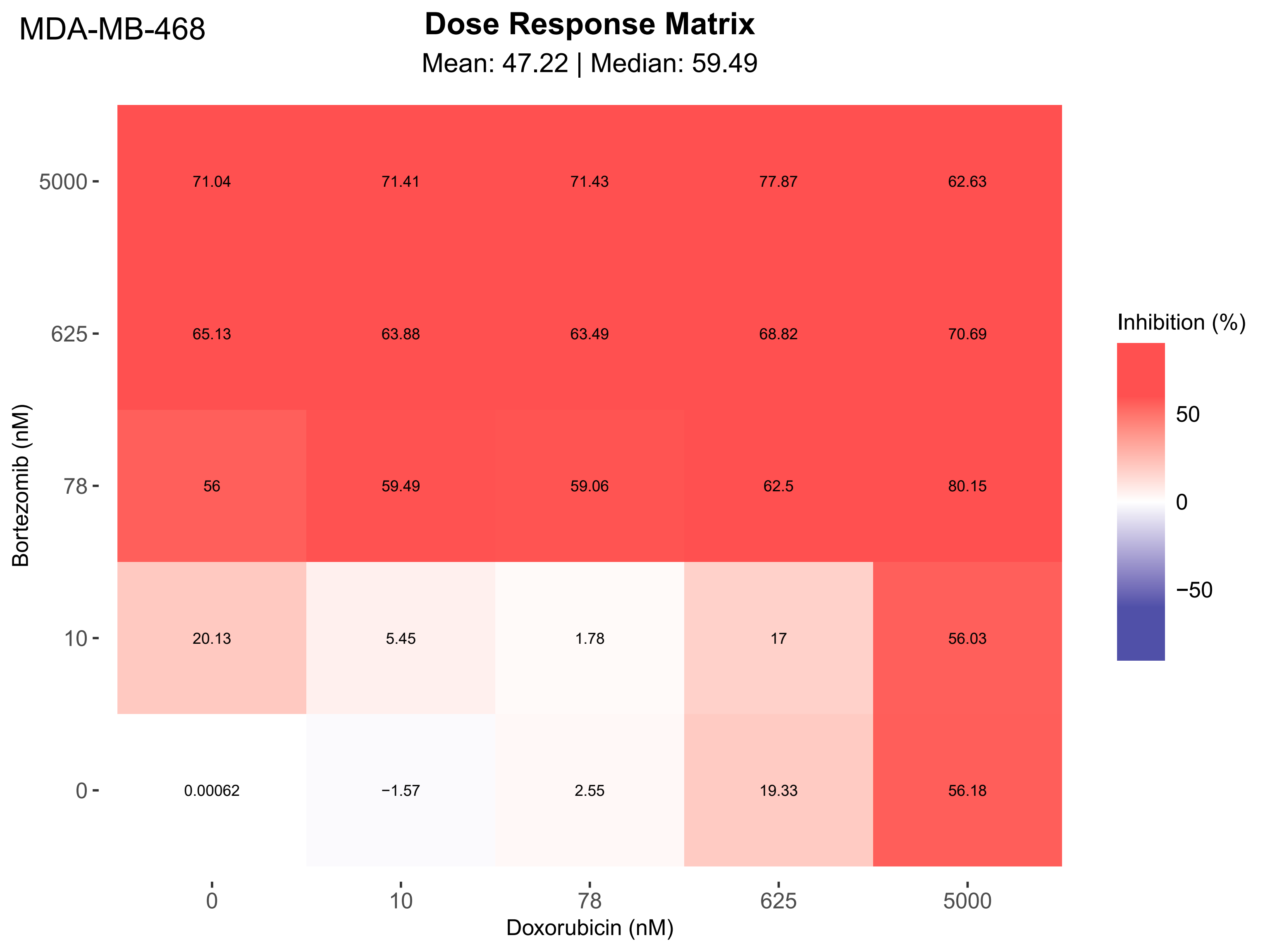
MDA-MB-468
Dose Response Matrix
Mean: 47.22 | Median: 59.49
Bortezomib (nM)
5000
625
78
10
0
71.04
71.41
71.43
77.87
62.63
65.13
63.88
63.49
68.82
70.69
56
59.49
59.06
62.5
80.15
20.13
5.45
1.78
17
56.03
0.00062
−1.57
2.55
19.33
56.18
0
10
78
625
5000
Doxorubicin (nM)
Inhibition (%)
50
0
−50

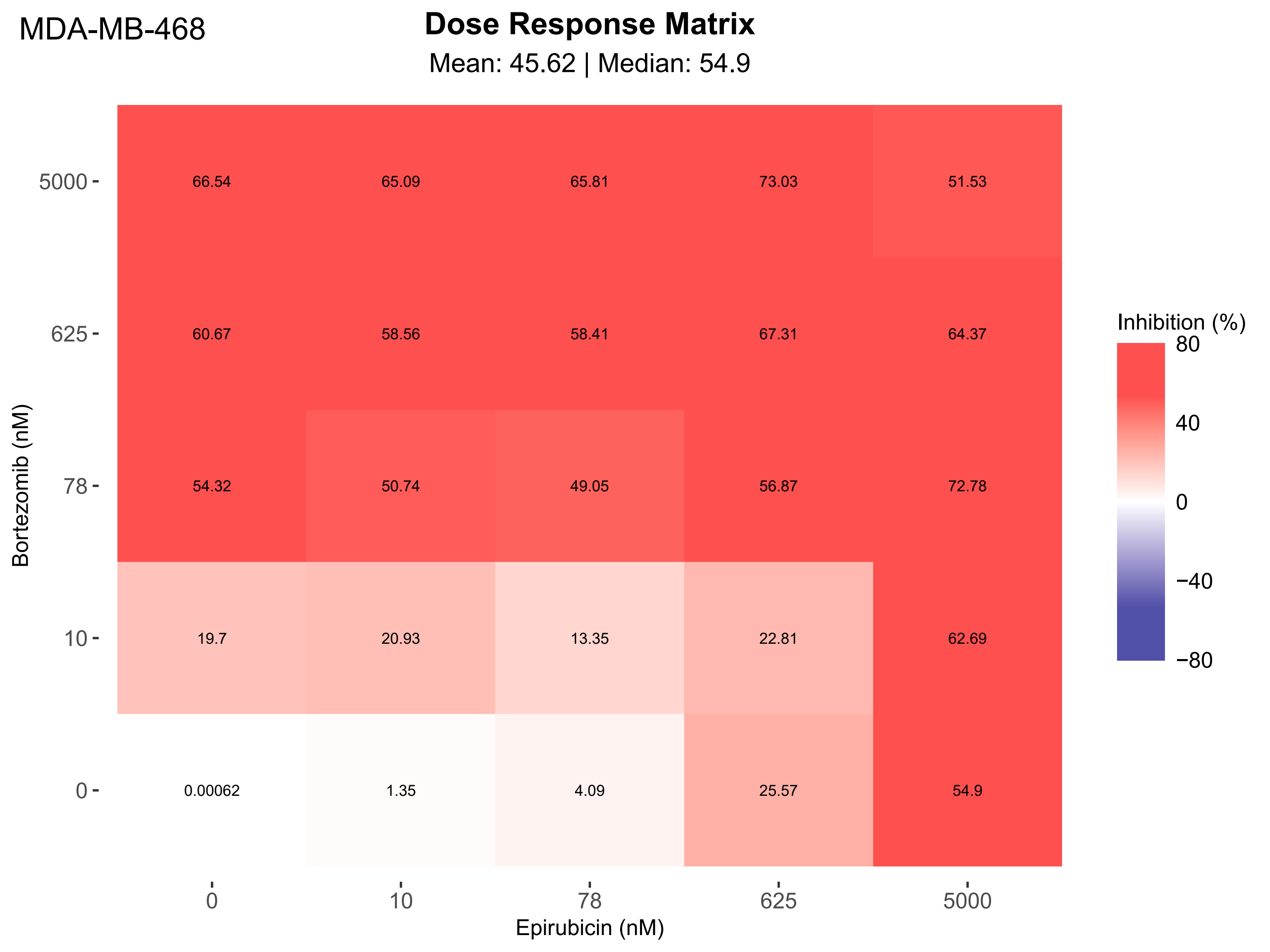
MDA-MB-468
Dose Response Matrix
Mean: 45.62 | Median: 54.9
Bortezomib (nM)
5000
625
78
10
0
66.54
65.09
65.81
73.03
51.53
60.67
58.56
58.41
67.31
64.37
54.32
50.74
49.05
56.87
72.78
19.7
20.93
13.35
22.81
62.69
0.00062
1.35
4.09
25.57
54.9
0
10
78
625
5000
Epirubicin (nM)
Inhibition (%)
80
40
0
−40
−80

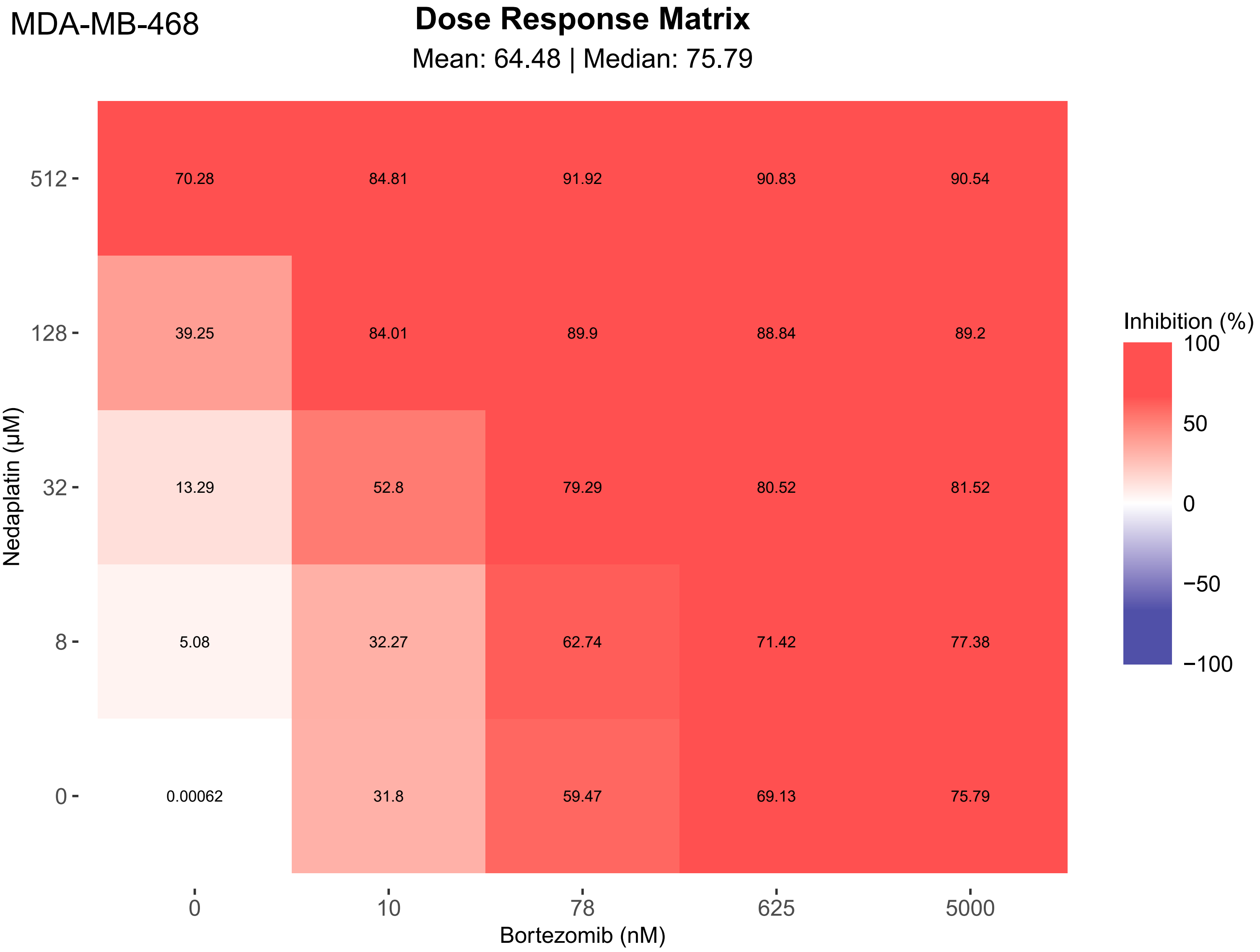
MDA-MB-468
Dose Response Matrix
Mean: 64.48 | Median: 75.79
512
128
32
8
0
Nedaplatin (μM)
70.28
84.81
91.92
90.83
90.54
39.25
84.01
89.9
88.84
89.2
13.29
52.8
79.29
80.52
81.52
5.08
32.27
62.74
71.42
77.38
0.00062
31.8
59.47
69.13
75.79
0
10
78
625
5000
Bortezomib (nM)
Inhibition (%)
100
50
0
−50
−100

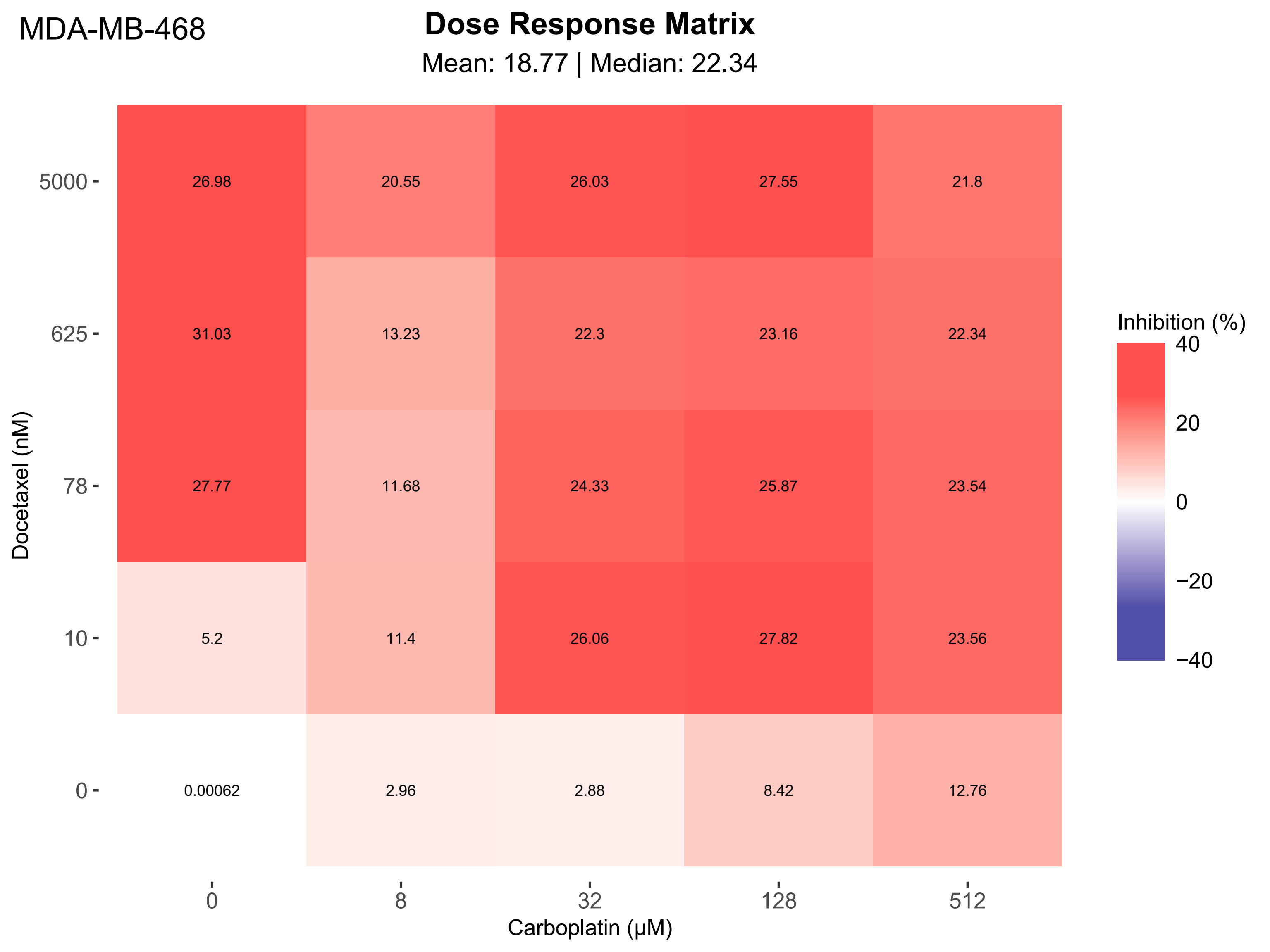
MDA-MB-468
Dose Response Matrix
Mean: 18.77 | Median: 22.34
Docetaxel (nM)
5000
625
78
10
0
26.98
20.55
26.03
27.55
21.8
31.03
13.23
22.3
23.16
22.34
27.77
11.68
24.33
25.87
23.54
5.2
11.4
26.06
27.82
23.56
0.00062
2.96
2.88
8.42
12.76
0
8
32
128
512
Carboplatin (μM)
Inhibition (%)
40
20
0
−20
−40

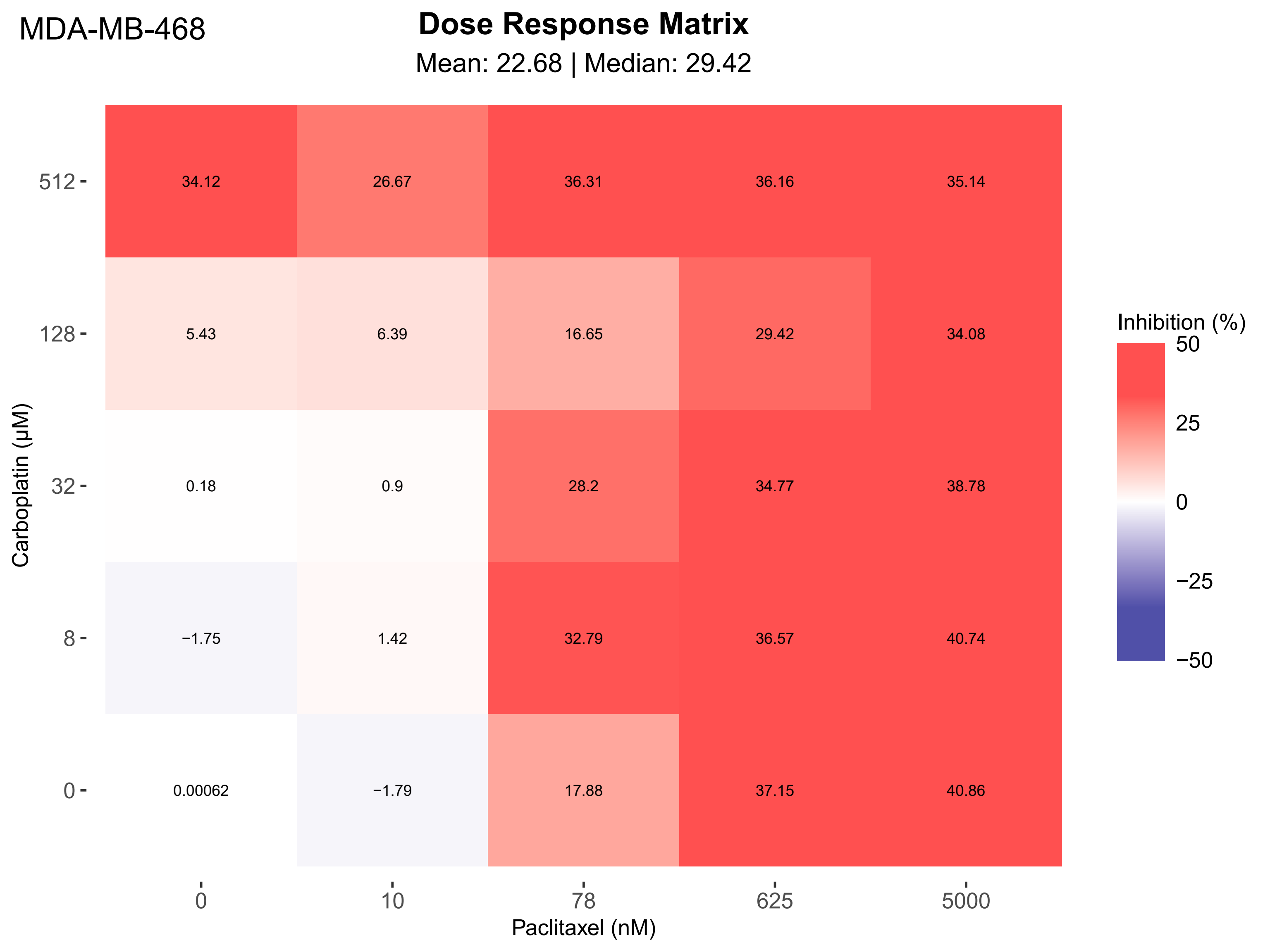
MDA-MB-468
Dose Response Matrix
Mean: 22.68 | Median: 29.42
Carboplatin (μM)
512
128
32
8
0
34.12
26.67
36.31
36.16
35.14
5.43
6.39
16.65
29.42
34.08
0.18
0.9
28.2
34.77
38.78
−1.75
1.42
32.79
36.57
40.74
0.00062
−1.79
17.88
37.15
40.86
0
10
78
625
5000
Paclitaxel (nM)
Inhibition (%)
50
25
0
−25
−50

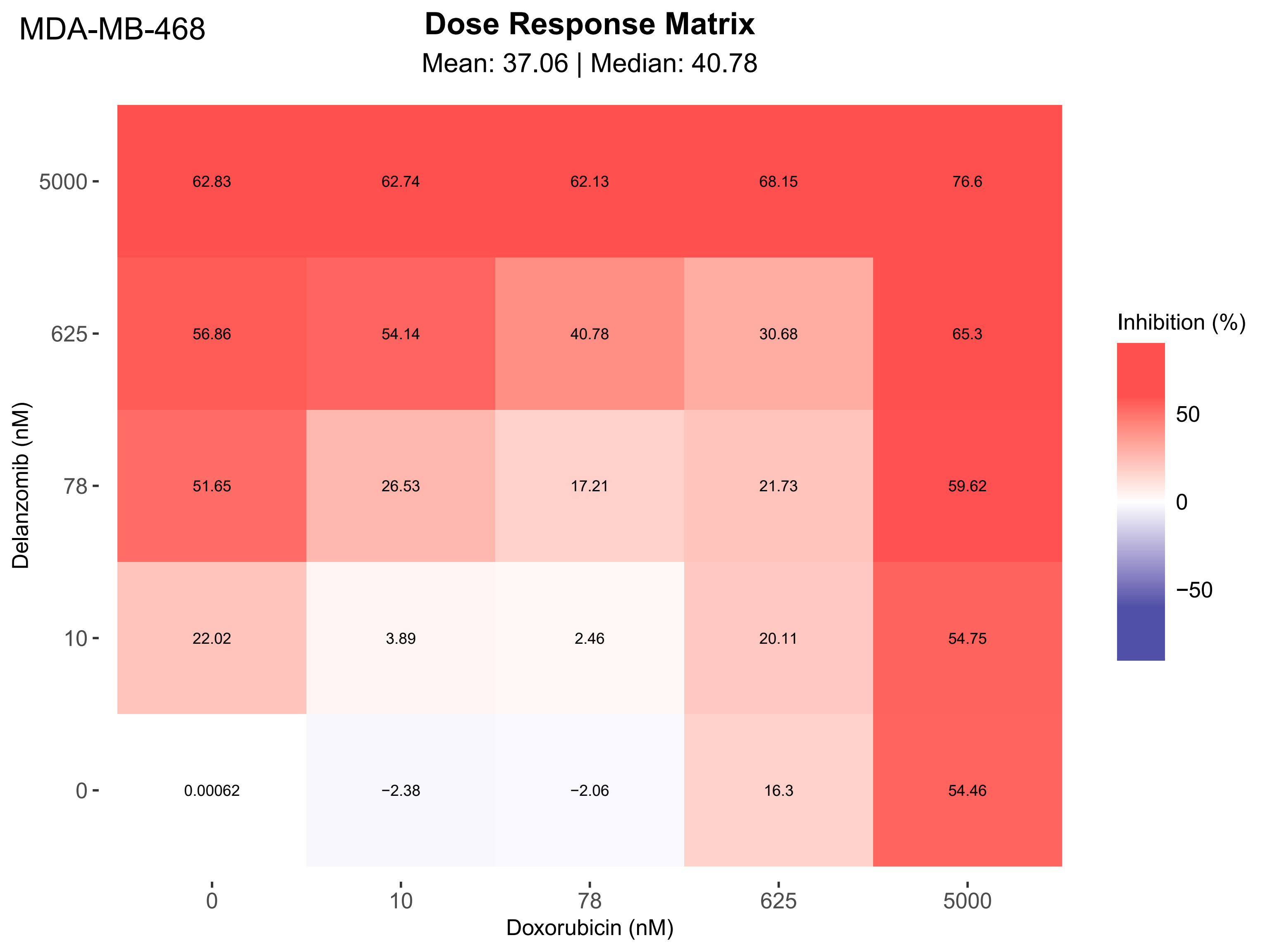
MDA-MB-468
Dose Response Matrix
Mean: 37.06 | Median: 40.78
Inhibition (%)
50
0
−50
Delanzomib (nM)
5000
625
78
10
0
Doxorubicin (nM)
0
10
78
625
5000
62.83
62.74
62.13
68.15
76.6
56.86
54.14
40.78
30.68
65.3
51.65
26.53
17.21
21.73
59.62
22.02
3.89
2.46
20.11
54.75
0.00062
−2.38
−2.06
16.3
54.46

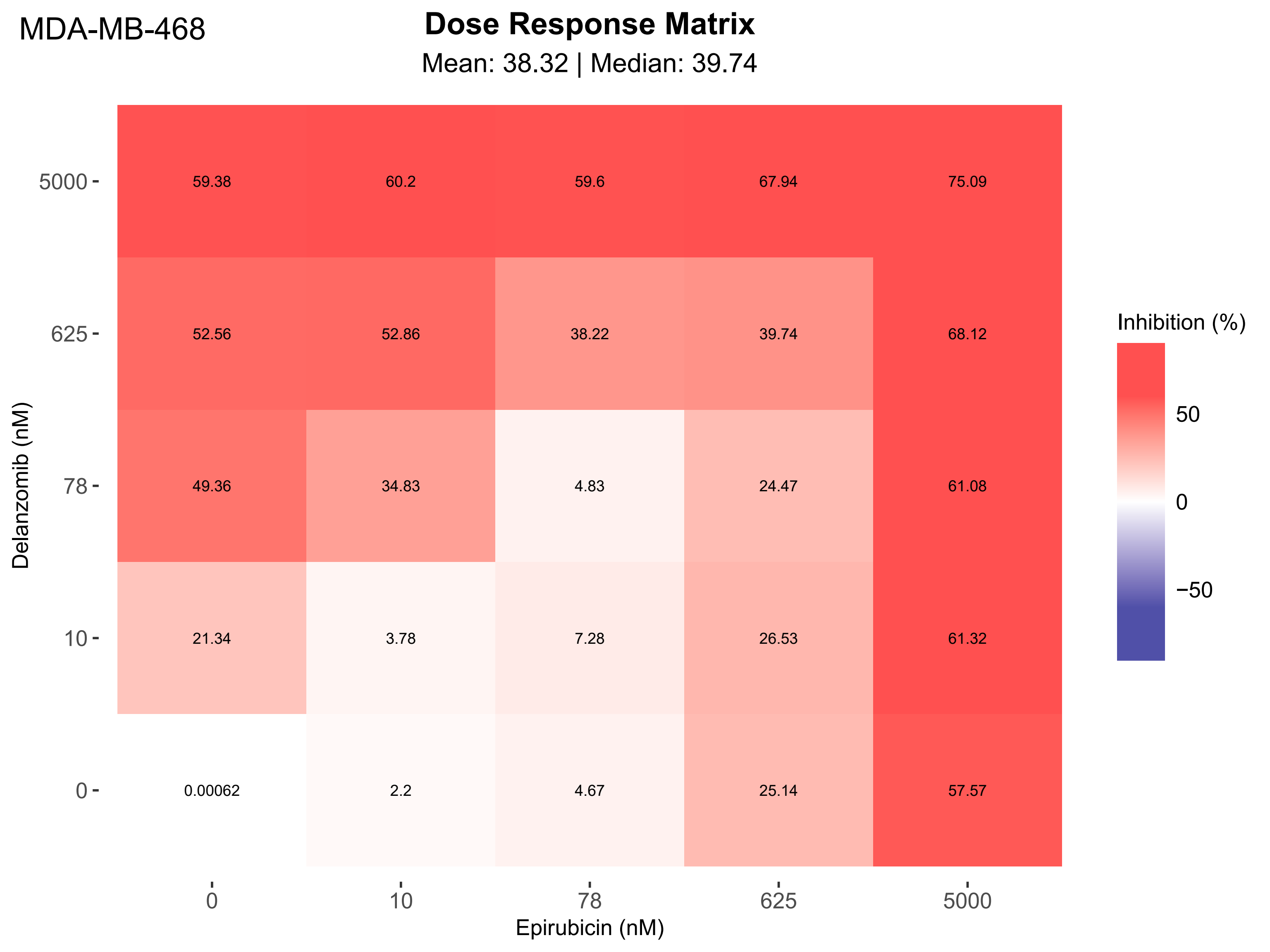

MDA-MB-468
Dose Response Matrix
Mean: 38.32 | Median: 39.74
Delanzomib (nM)
5000
625
78
10
0
59.38
60.2
59.6
67.94
75.09
52.56
52.86
38.22
39.74
68.12
49.36
34.83
4.83
24.47
61.08
21.34
3.78
7.28
26.53
61.32
0.00062
2.2
4.67
25.14
57.57
0
10
78
625
5000
Epirubicin (nM)
Inhibition (%)
50
0
−50

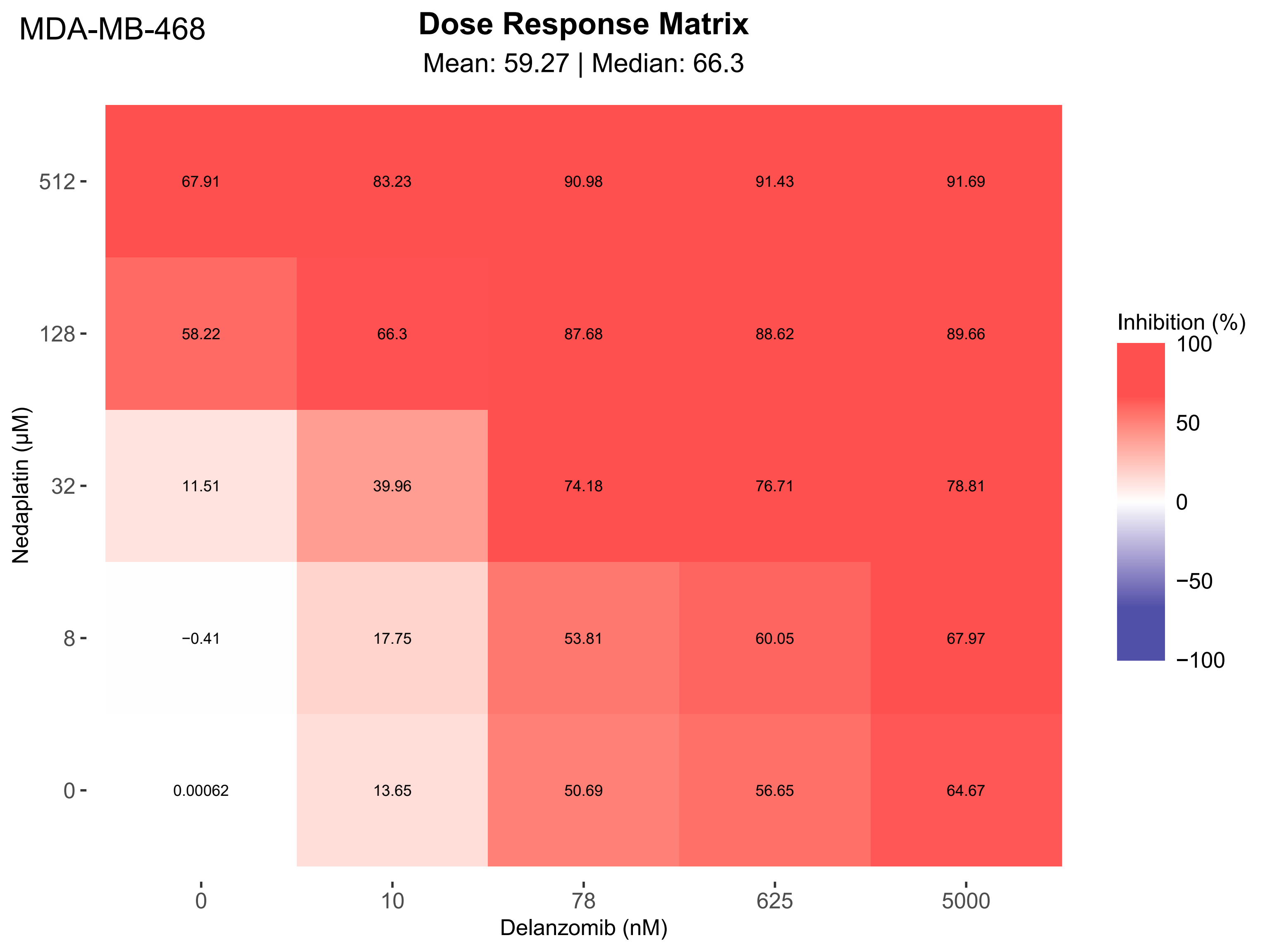
MDA-MB-468
Dose Response Matrix
Mean: 59.27 | Median: 66.3
512
128
32
8
0
67.91
83.23
90.98
91.43
91.69
58.22
66.3
87.68
88.62
89.66
11.51
39.96
74.18
76.71
78.81
−0.41
17.75
53.81
60.05
67.97
0.00062
13.65
50.69
56.65
64.67
0
10
78
625
5000
Delanzomib (nM)
Nedaplatin (μM)
Inhibition (%)
100
50
0
−50
−100

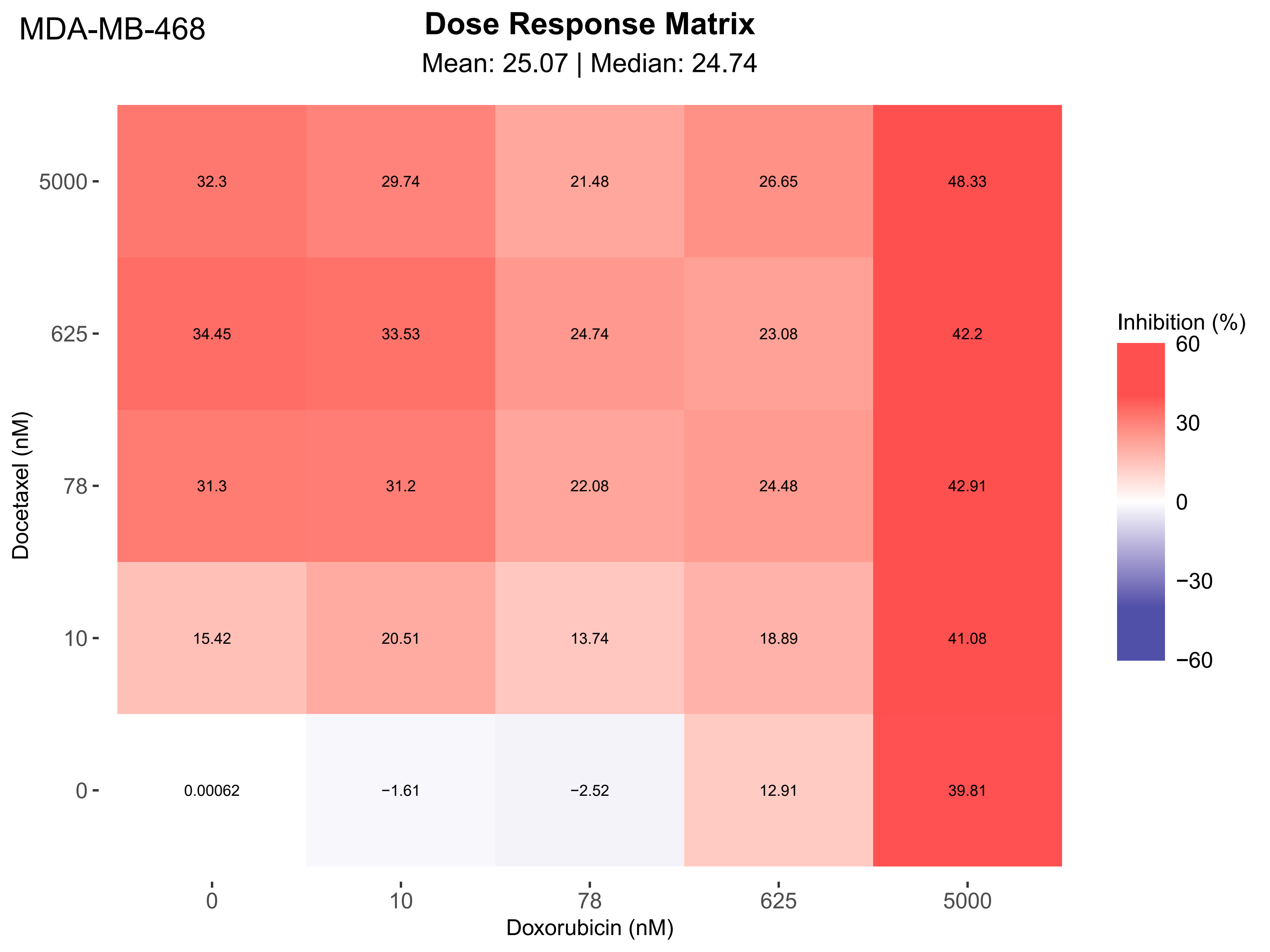

MDA-MB-468
Dose Response Matrix
Mean: 25.07 | Median: 24.74
Docetaxel (nM)
5000
625
78
10
0
32.3
29.74
21.48
26.65
48.33
34.45
33.53
24.74
23.08
42.2
31.3
31.2
22.08
24.48
42.91
15.42
20.51
13.74
18.89
41.08
0.00062
−1.61
−2.52
12.91
39.81
0
10
78
625
5000
Doxorubicin (nM)
Inhibition (%)
60
30
0
−30
−60

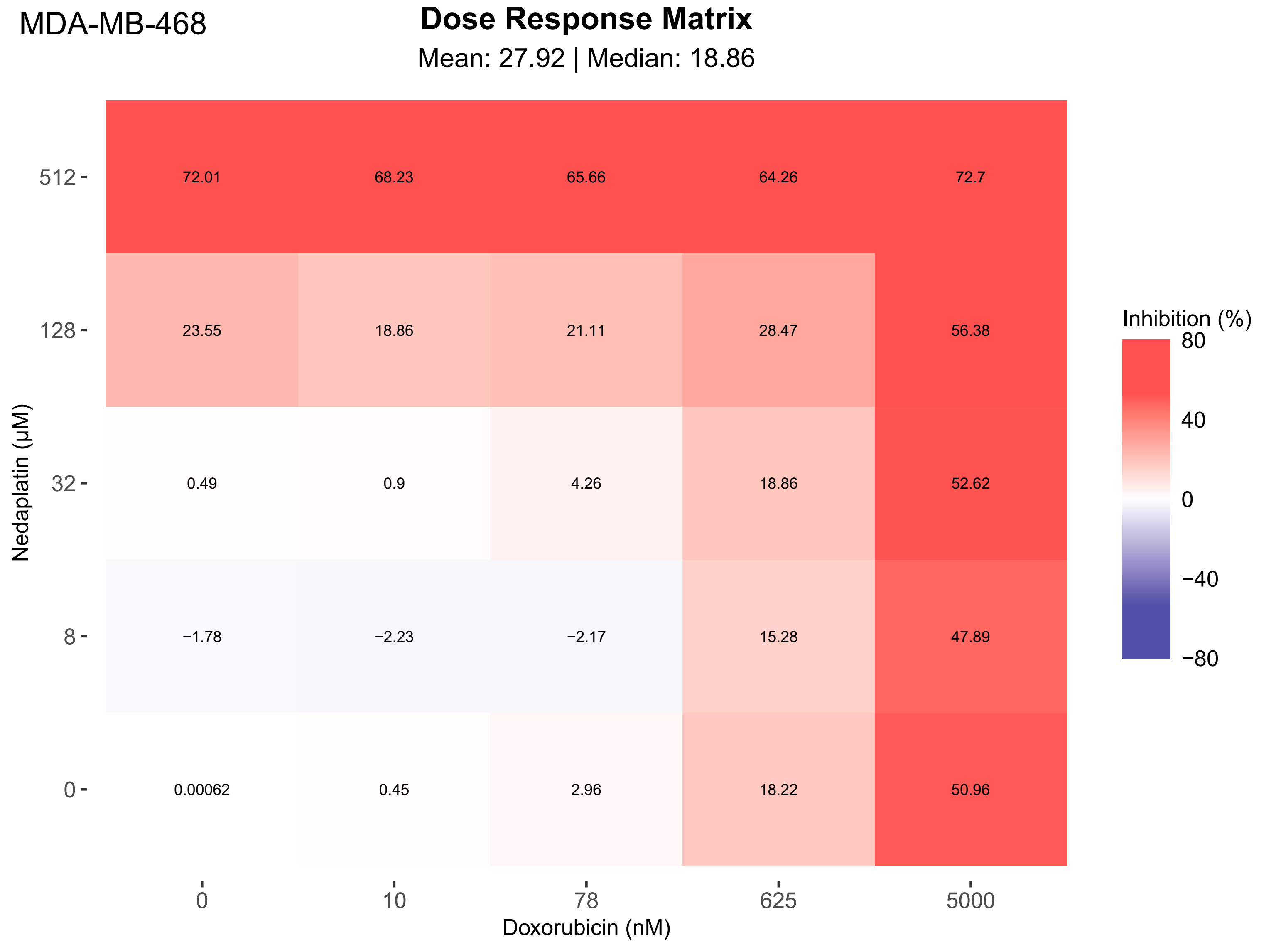

MDA-MB-468
Dose Response Matrix
Mean: 27.92 | Median: 18.86
512
128
32
8
0
Nedaplatin (μM)
72.01
68.23
65.66
64.26
72.7
23.55
18.86
21.11
28.47
56.38
0.49
0.9
4.26
18.86
52.62
−1.78
−2.23
−2.17
15.28
47.89
0.00062
0.45
2.96
18.22
50.96
0
10
78
625
5000
Doxorubicin (nM)
Inhibition (%)
80
40
0
−40
−80

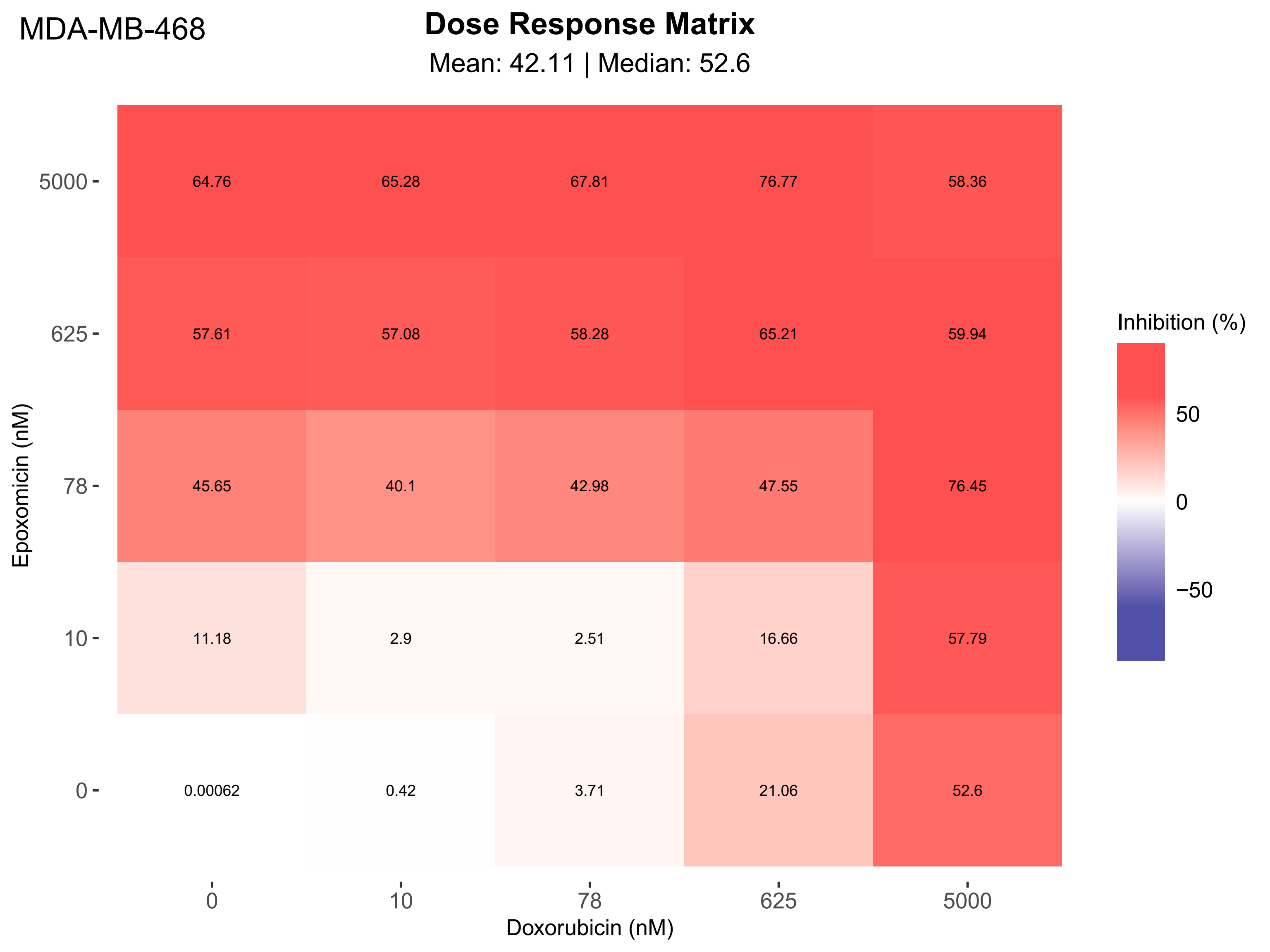
MDA-MB-468
Dose Response Matrix
Mean: 42.11 | Median: 52.6
Inhibition (%)
50
0
−50
Epoxomicin (nM)
5000
625
78
10
0
Doxorubicin (nM)
0
10
78
625
5000
64.76
65.28
67.81
76.77
58.36
57.61
57.08
58.28
65.21
59.94
45.65
40.1
42.98
47.55
76.45
11.18
2.9
2.51
16.66
57.79
0.00062
0.42
3.71
21.06
52.6

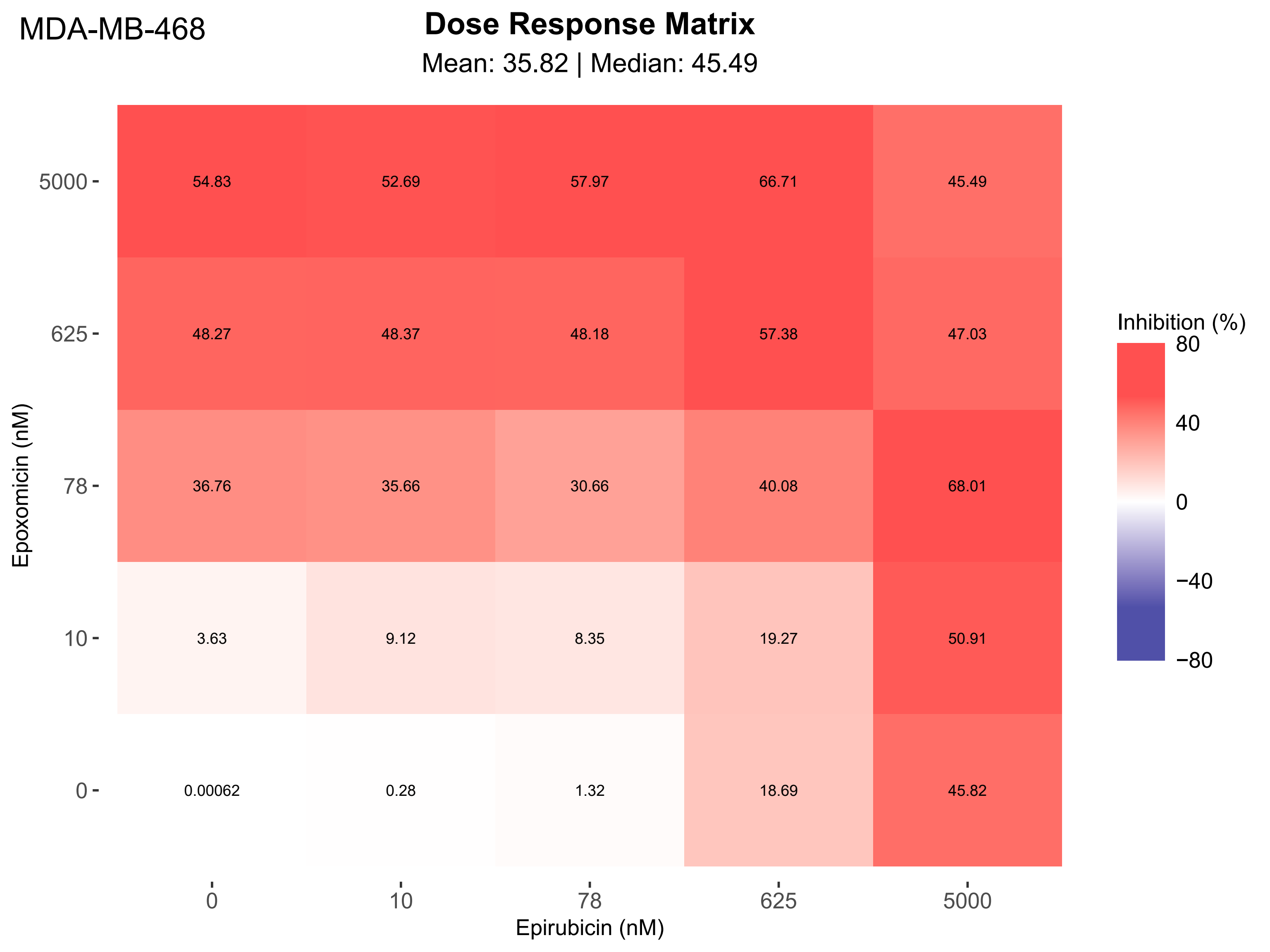
MDA-MB-468
Dose Response Matrix
Mean: 35.82 | Median: 45.49
Epoxomicin (nM)
5000
625
78
10
0
54.83
52.69
57.97
66.71
45.49
48.27
48.37
48.18
57.38
47.03
36.76
35.66
30.66
40.08
68.01
3.63
9.12
8.35
19.27
50.91
0.00062
0.28
1.32
18.69
45.82
0
10
78
625
5000
Epirubicin (nM)
Inhibition (%)
80
40
0
−40
−80

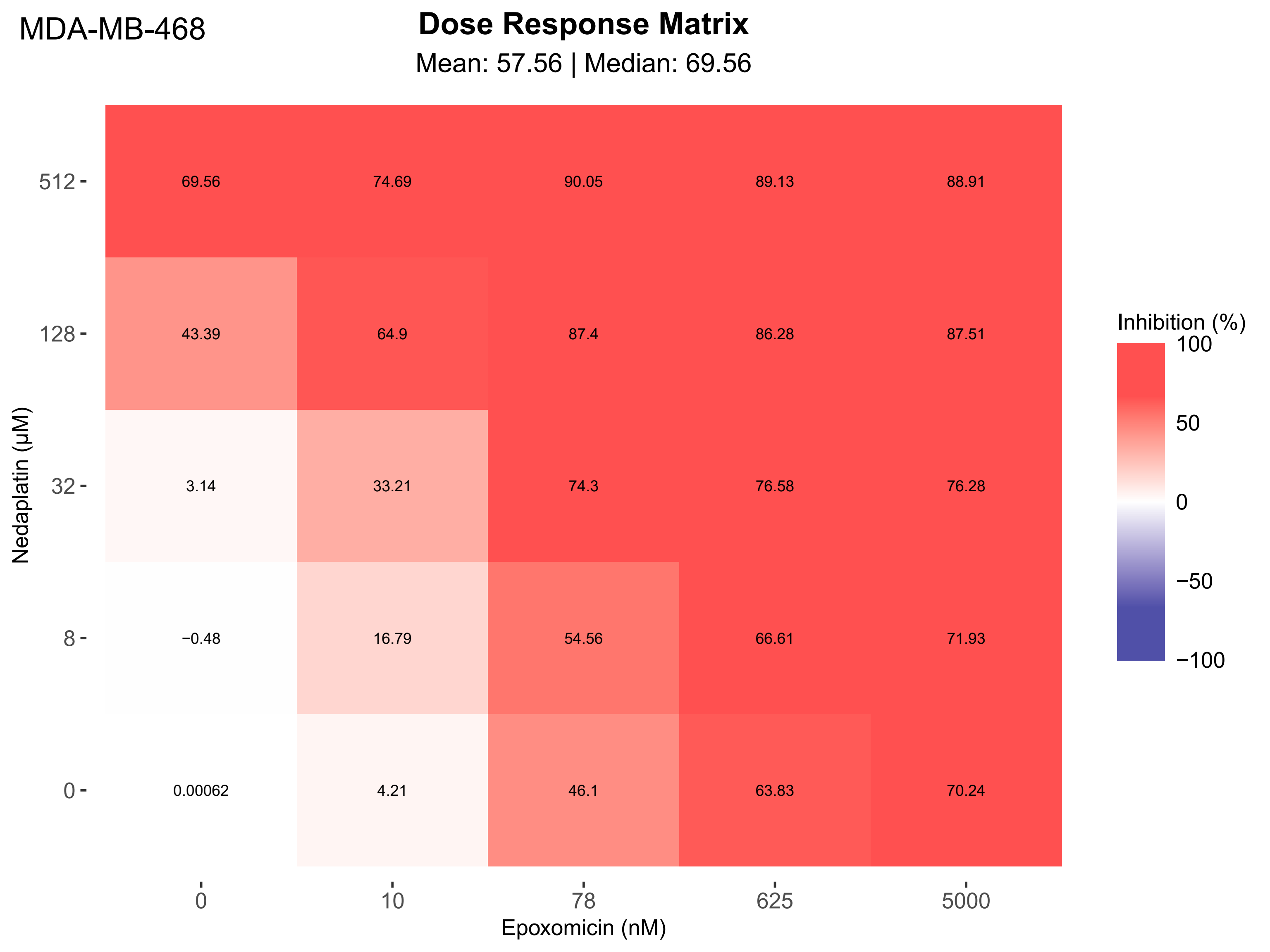
MDA-MB-468
Dose Response Matrix
Mean: 57.56 | Median: 69.56
Nedaplatin (μM)
512
128
32
8
0
69.56
74.69
90.05
89.13
88.91
43.39
64.9
87.4
86.28
87.51
3.14
33.21
74.3
76.58
76.28
−0.48
16.79
54.56
66.61
71.93
0.00062
4.21
46.1
63.83
70.24
0
10
78
625
5000
Epoxomicin (nM)
Inhibition (%)
100
50
0
−50
−100
