# Supplementary material for: Repurposing proteasome inhibitors for improved treatment of triple-negative breast cancer
Source: Cell Death Discov. 2024 Jan 29;10:57. doi: 10.1038/s41420-024-01819-5 (PMC10825133; doi:10.1038/s41420-024-01819-5)
Supplement: Supplementary file 5 — Supplementary Figure 3 [file 41420_2024_1819_MOESM5_ESM.pdf]

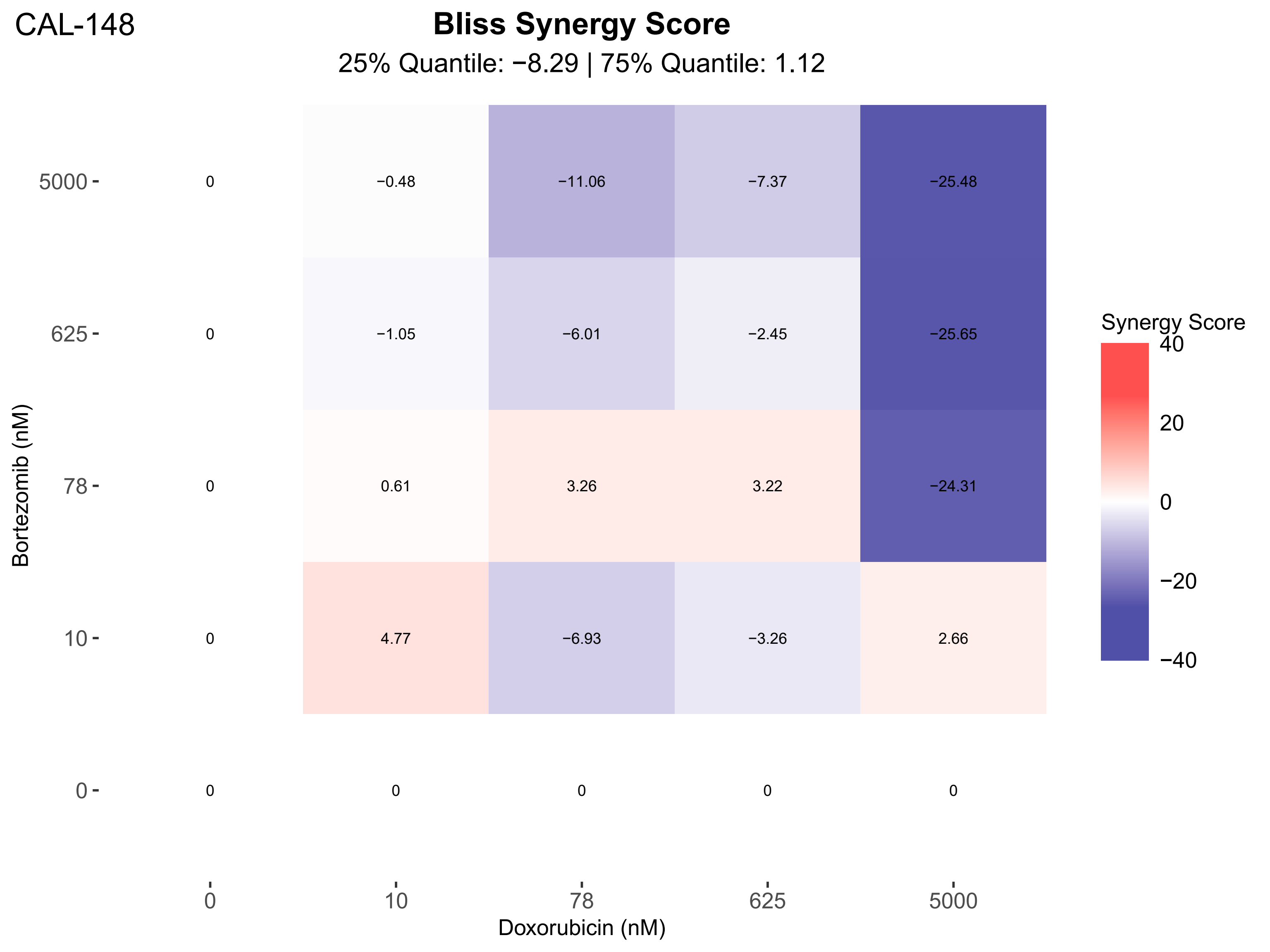
CAL-148
Bliss Synergy Score
25% Quantile: −8.29 | 75% Quantile: 1.12
Bortezomib (nM)
5000
625
78
10
0
Doxorubicin (nM)
0
10
78
625
5000
0
−0.48
−11.06
−7.37
−25.48
0
−1.05
−6.01
−2.45
−25.65
0
0.61
3.26
3.22
−24.31
0
4.77
−6.93
−3.26
2.66
0
0
0
0
0
Synergy Score
40
20
0
−20
−40

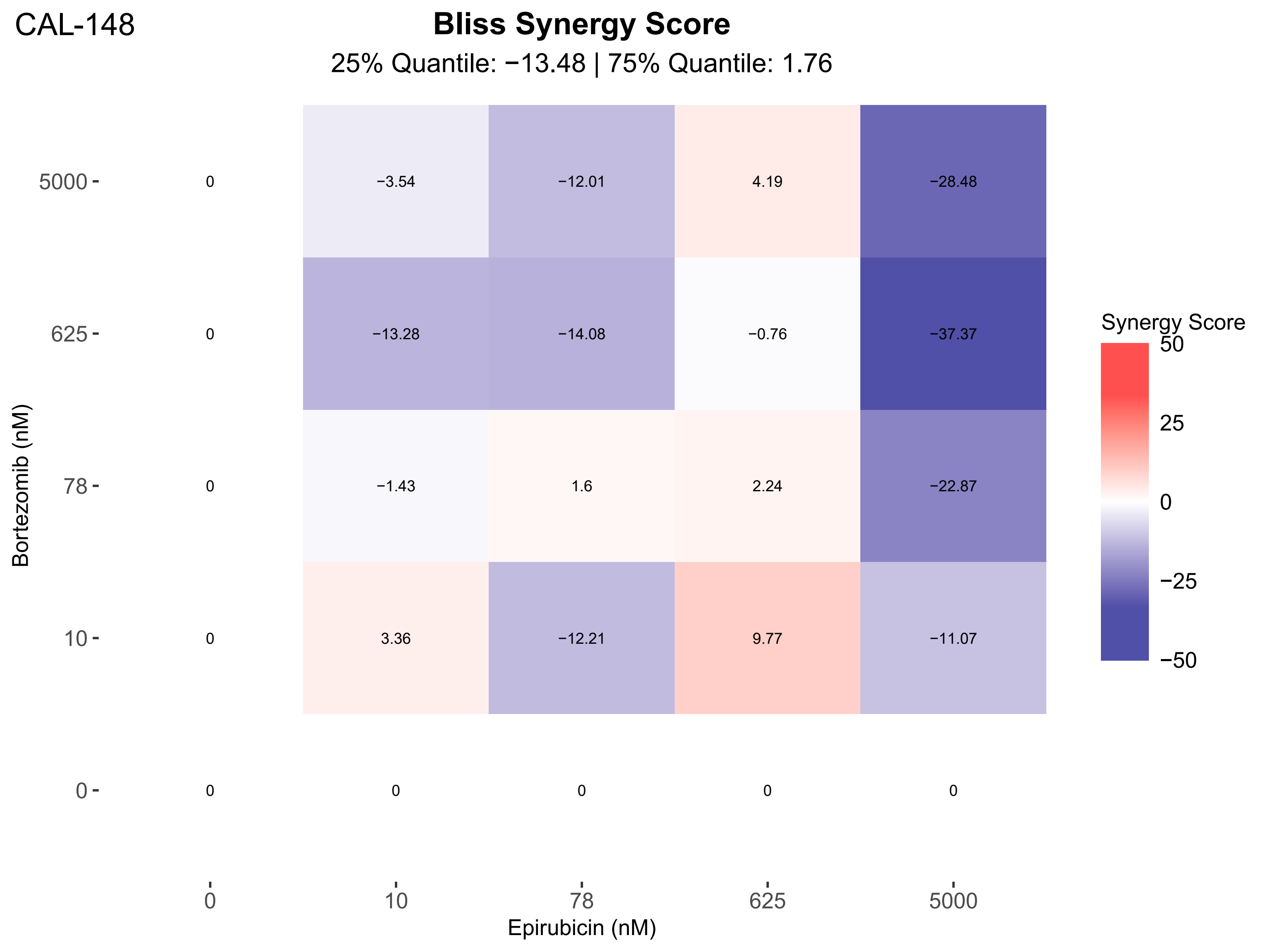
CAL-148
Bliss Synergy Score
25% Quantile: −13.48 | 75% Quantile: 1.76
Bortezomib (nM)
5000
625
78
10
0
Epirubicin (nM)
0
10
78
625
5000
0
−3.54
−12.01
4.19
−28.48
0
−13.28
−14.08
−0.76
−37.37
0
−1.43
1.6
2.24
−22.87
0
3.36
−12.21
9.77
−11.07
0
0
0
0
0
Synergy Score
50
25
0
−25
−50

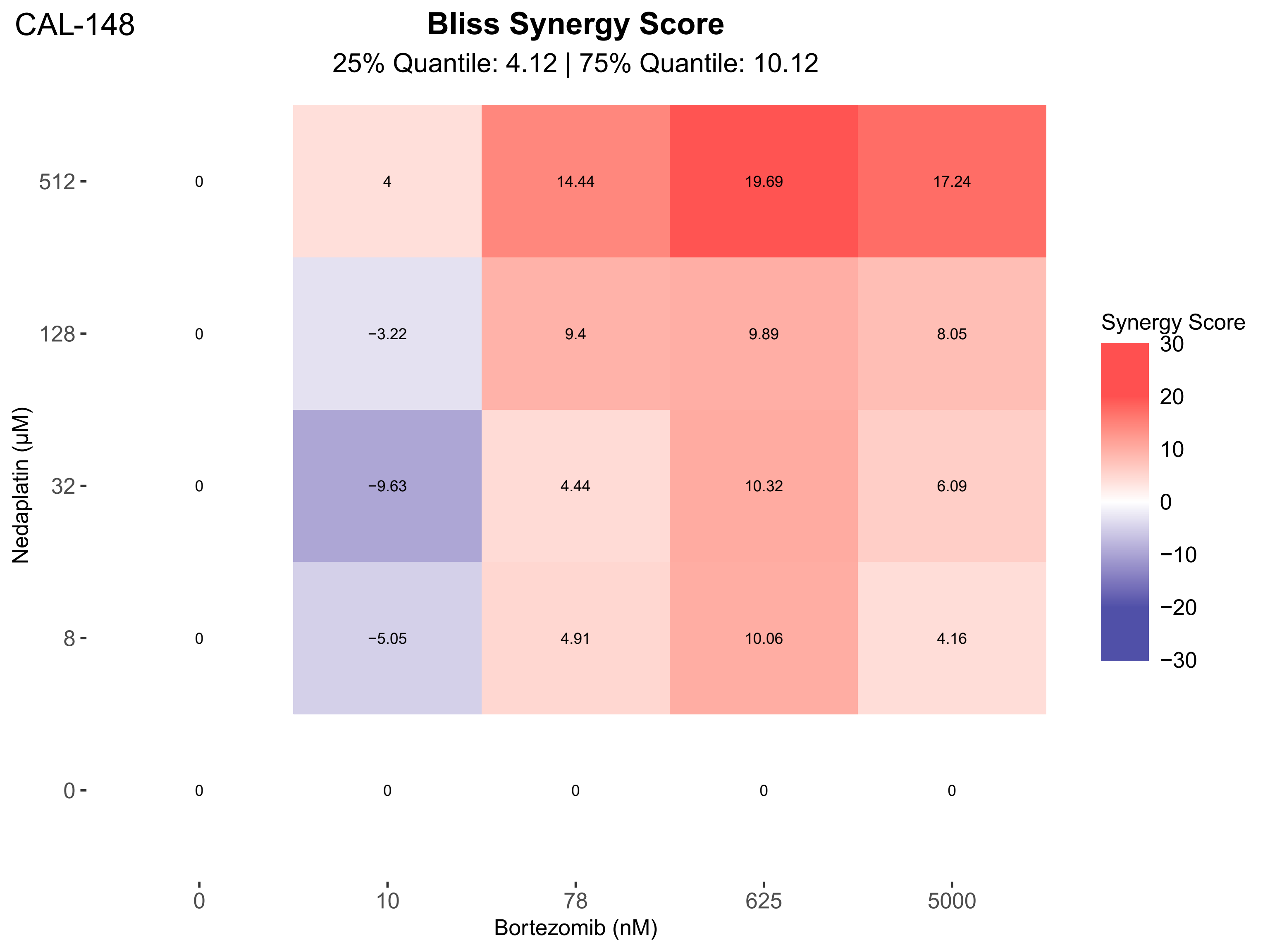
CAL-148
Bliss Synergy Score
25% Quantile: 4.12 | 75% Quantile: 10.12
Nedaplatin (μM)
512
128
32
8
0
0
4
14.44
19.69
17.24
0
−3.22
9.4
9.89
8.05
0
−9.63
4.44
10.32
6.09
0
−5.05
4.91
10.06
4.16
0
0
0
0
0
0
10
78
625
5000
Bortezomib (nM)
Synergy Score
30
20
10
0
−10
−20
−30

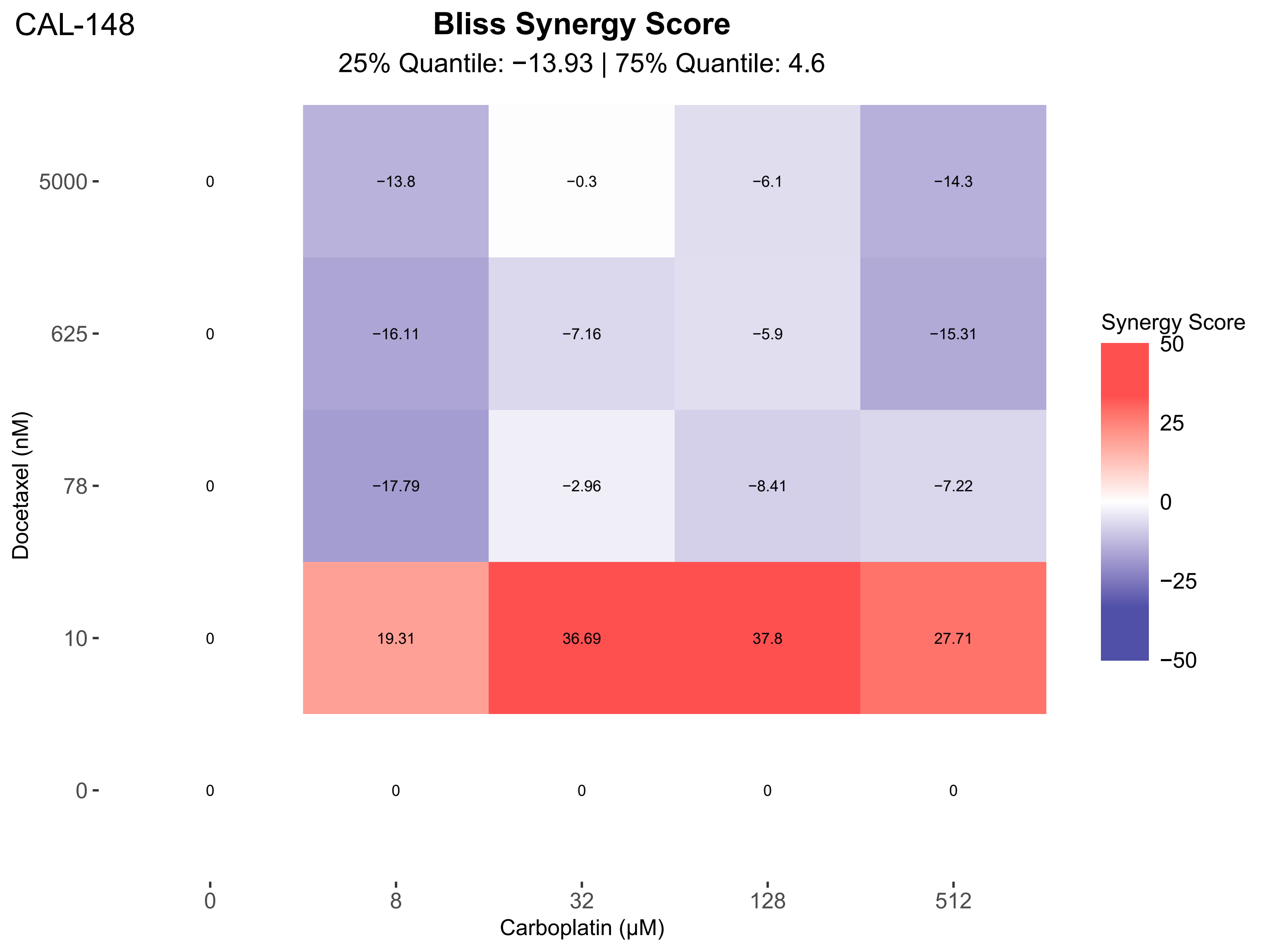

CAL-148
Bliss Synergy Score
25% Quantile: −13.93 | 75% Quantile: 4.6
Docetaxel (nM)
5000
625
78
10
0
0
−13.8
−0.3
−6.1
−14.3
0
−16.11
−7.16
−5.9
−15.31
0
−17.79
−2.96
−8.41
−7.22
0
19.31
36.69
37.8
27.71
0
0
0
0
0
Carboplatin (μM)
0
8
32
128
512
Synergy Score
50
25
0
−25
−50

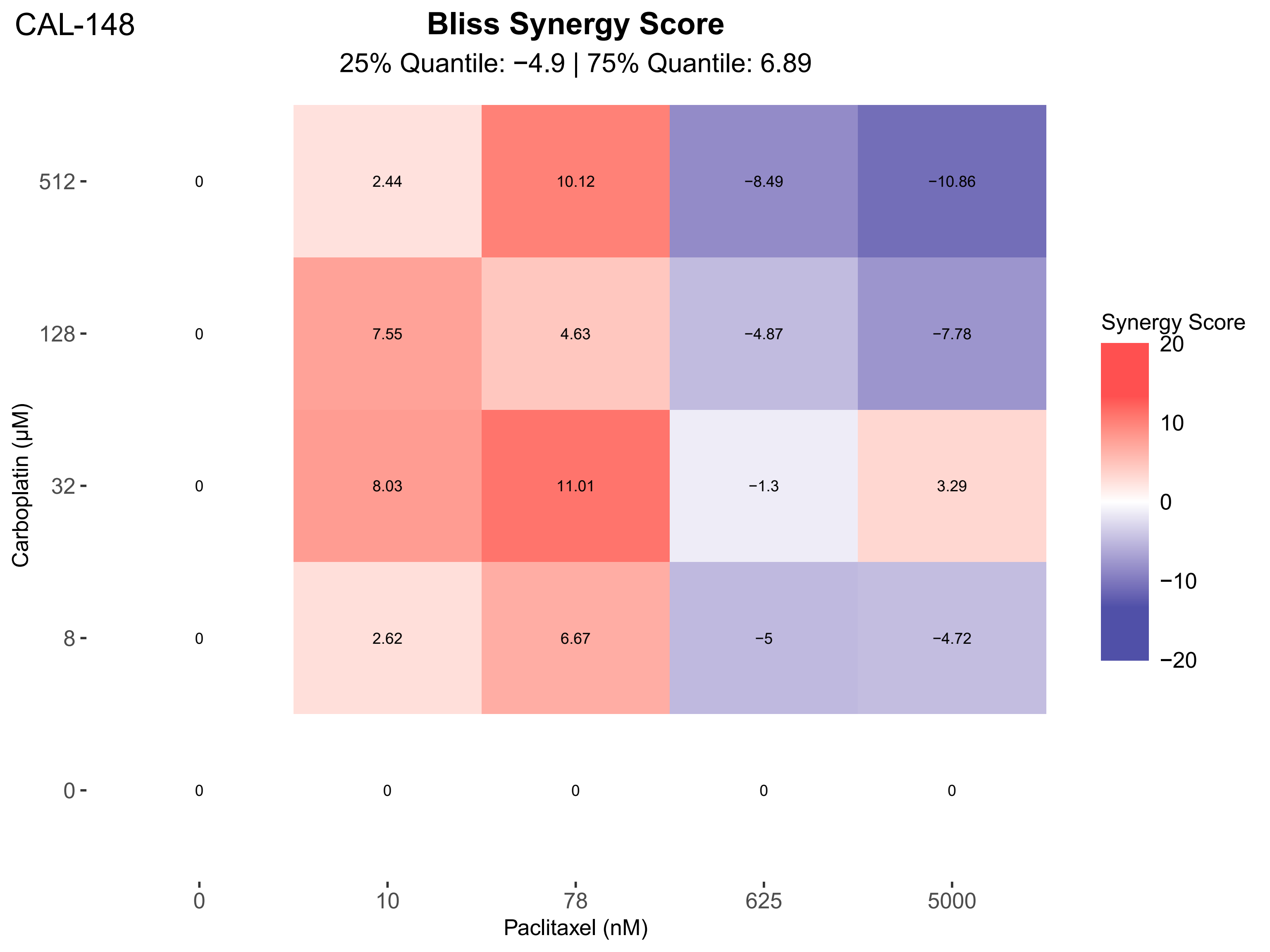
CAL-148
Bliss Synergy Score
25% Quantile: −4.9 | 75% Quantile: 6.89
Carboplatin (μM)
512
128
32
8
0
0
2.44
10.12
−8.49
−10.86
7.55
4.63
−4.87
−7.78
8.03
11.01
−1.3
3.29
2.62
6.67
−5
−4.72
10
78
625
5000
Paclitaxel (nM)
Synergy Score
20
10
−10
−20

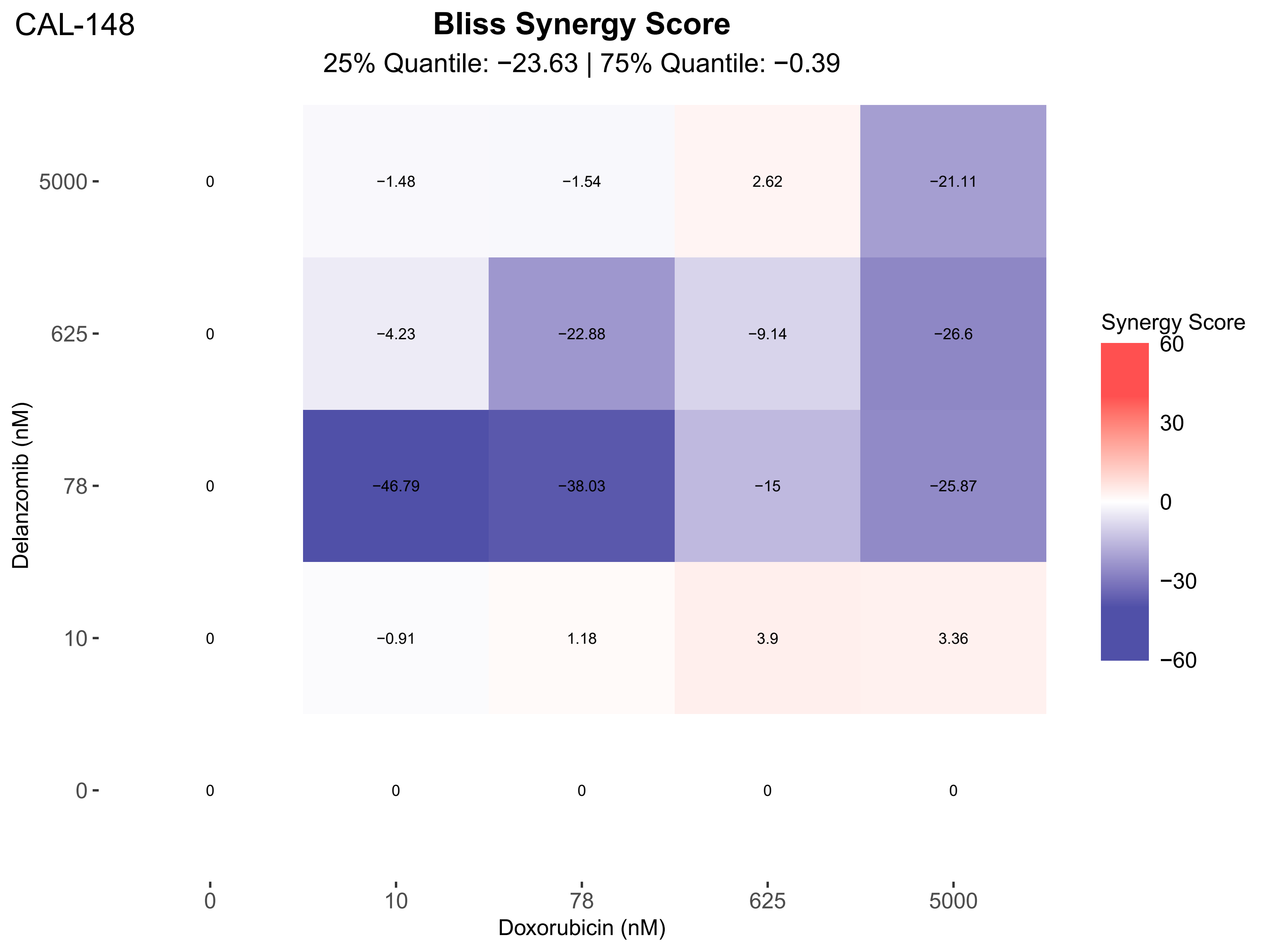

CAL-148
Bliss Synergy Score
25% Quantile: −23.63 | 75% Quantile: −0.39
Delanzomib (nM)
5000
625
78
10
0
Doxorubicin (nM)
0
10
78
625
5000
0 −1.48 −1.54 2.62 −21.11
0 −4.23 −22.88 −9.14 −26.6
0 −46.79 −38.03 −15 −25.87
0 −0.91 1.18 3.9 3.36
0 0 0 0 0
Synergy Score
60
30
0
−30
−60

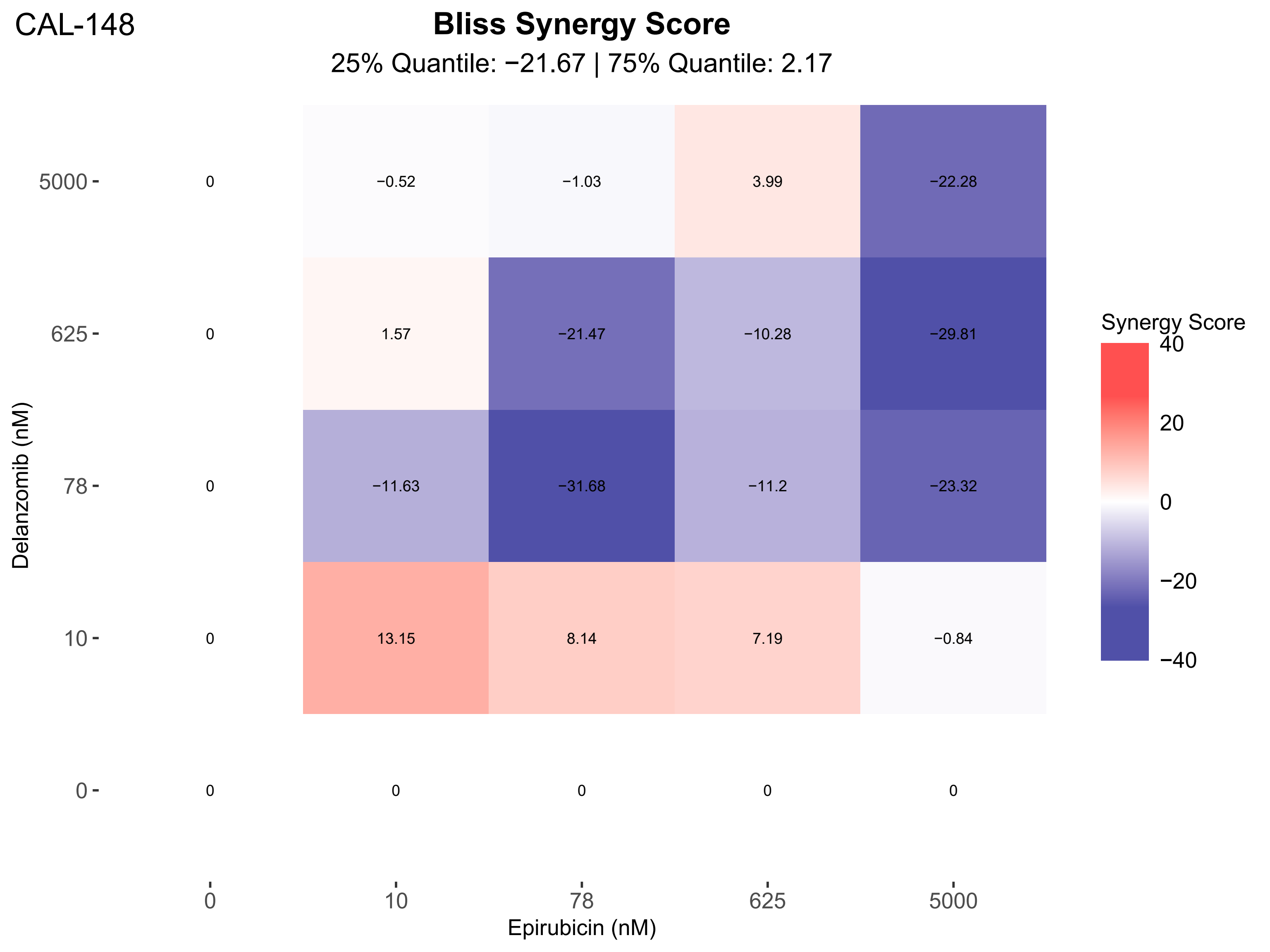

CAL-148
Bliss Synergy Score
25% Quantile: −21.67 | 75% Quantile: 2.17
Delanzomib (nM)
5000
625
78
10
0
0
−0.52
−1.03
3.99
−22.28
0
1.57
−21.47
−10.28
−29.81
0
−11.63
−31.68
−11.2
−23.32
0
13.15
8.14
7.19
−0.84
0
0
0
0
0
0
10
78
625
5000
Epirubicin (nM)
Synergy Score
40
20
0
−20
−40

CAL-148

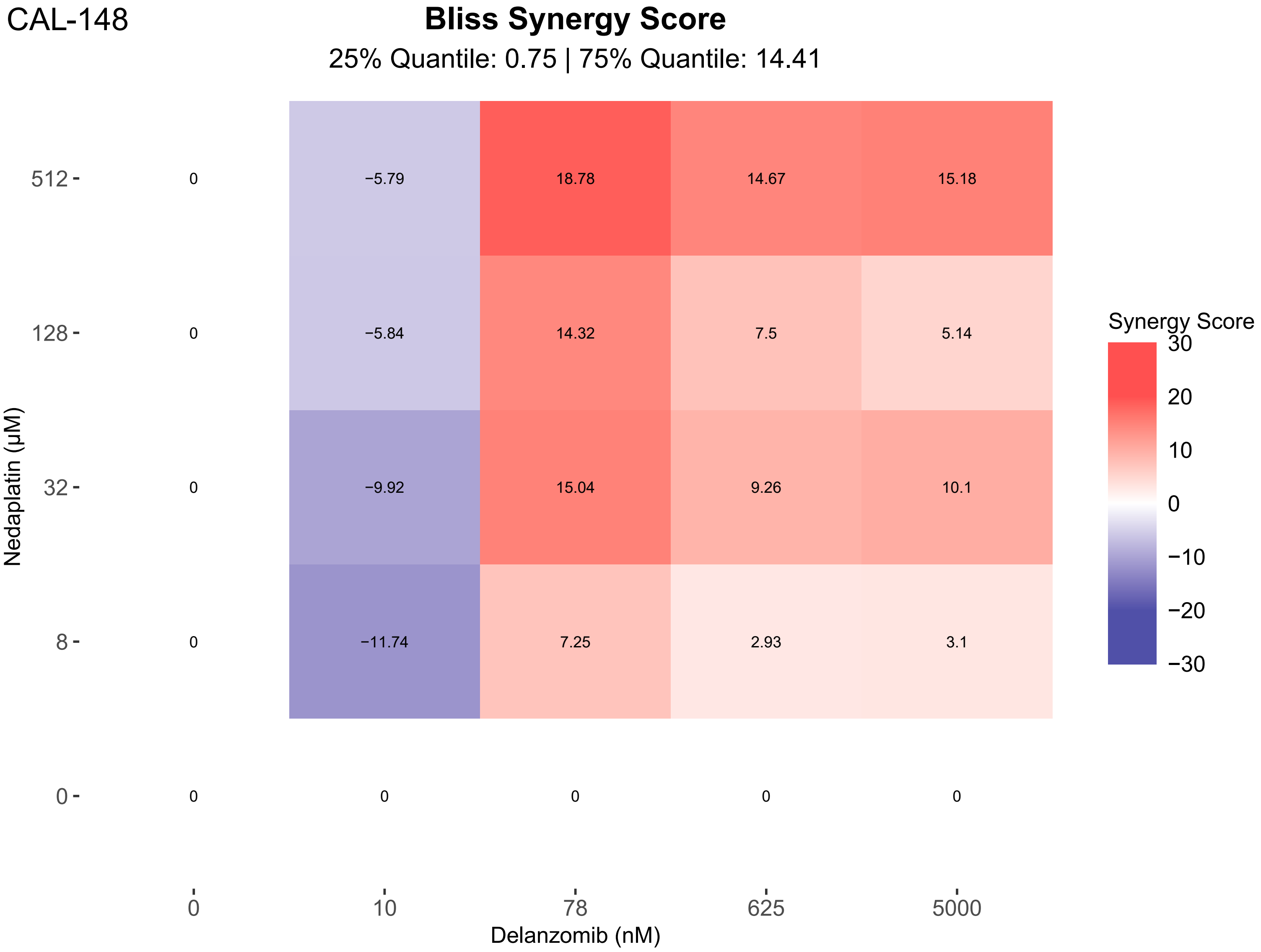

Bliss Synergy Score
25% Quantile: 0.75 | 75% Quantile: 14.41
Nedaplatin (μM)
512
128
32
8
0
Delanzomib (nM)
0
10
78
625
5000
0
−5.79
18.78
14.67
15.18
0
−5.84
14.32
7.5
5.14
0
−9.92
15.04
9.26
10.1
0
−11.74
7.25
2.93
3.1
0
0
0
0
0
Synergy Score
30
20
10
0
−10
−20
−30

CAL-148

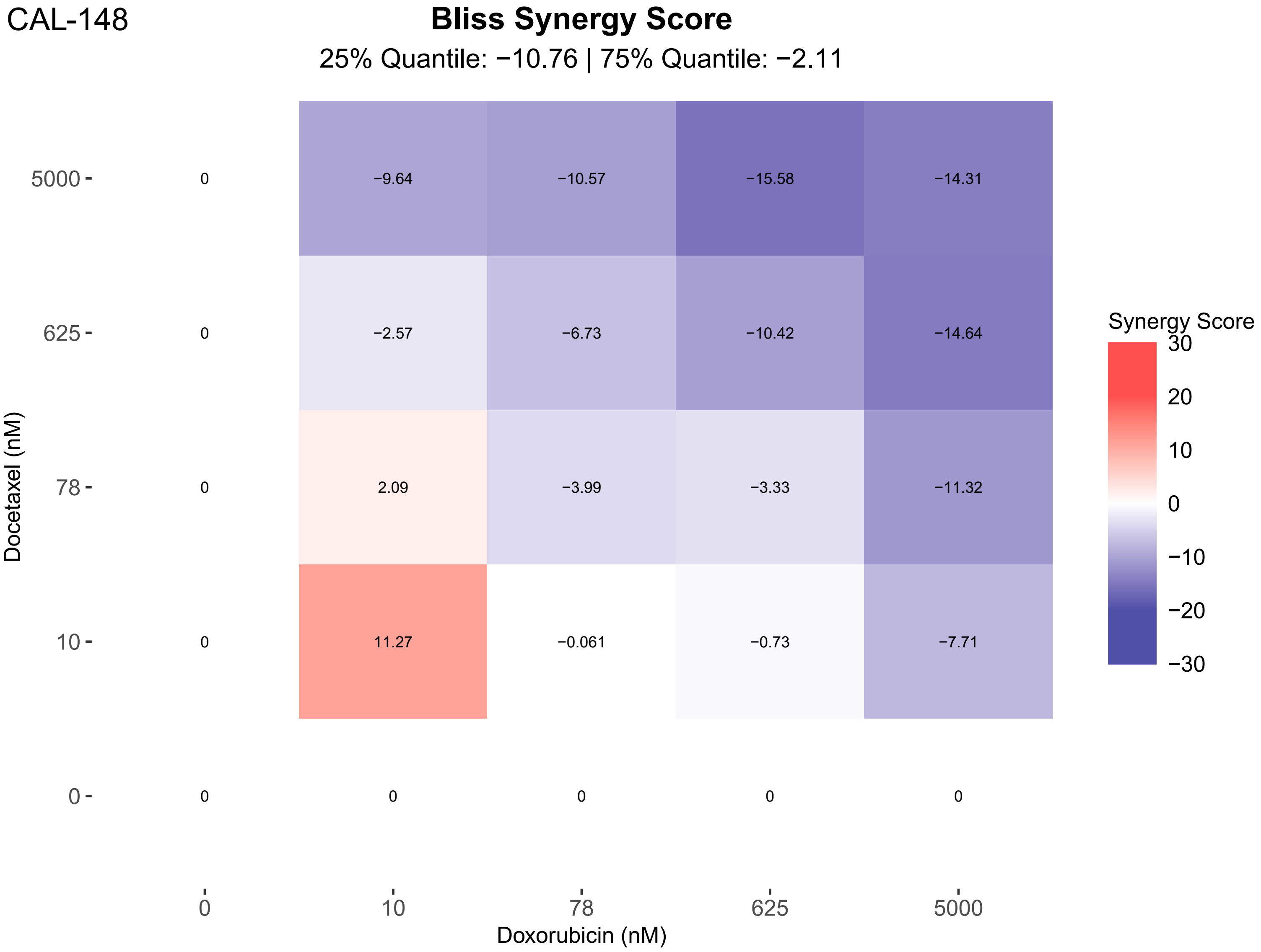

Bliss Synergy Score
25% Quantile: −10.76 | 75% Quantile: −2.11
Docetaxel (nM)
5000
625
78
10
0
Doxorubicin (nM)
0
10
78
625
5000
0
−9.64
−10.57
−15.58
−14.31
0
−2.57
−6.73
−10.42
−14.64
0
2.09
−3.99
−3.33
−11.32
0
11.27
−0.061
−0.73
−7.71
0
0
0
0
0
Synergy Score
30
20
10
0
−10
−20
−30

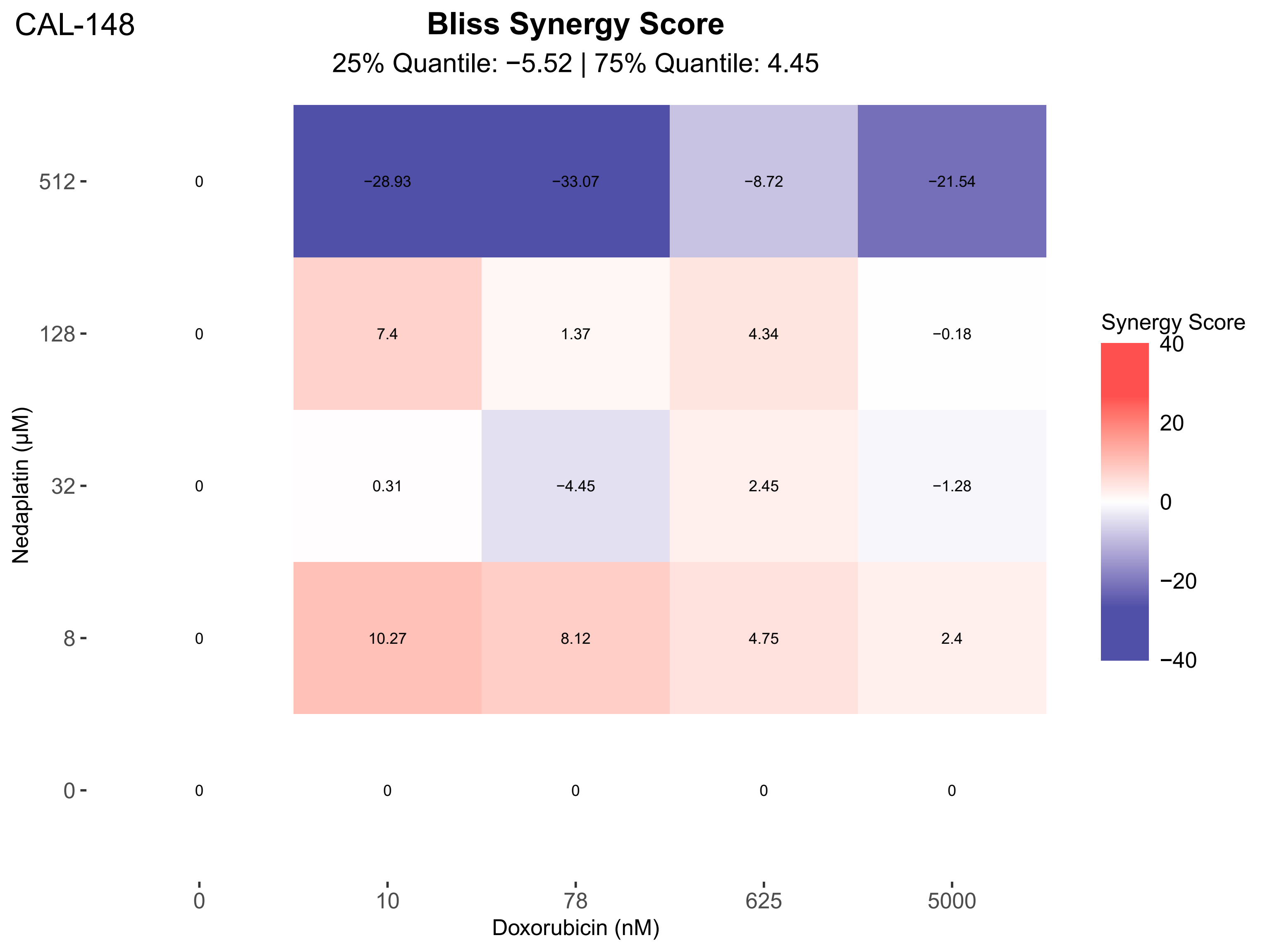

CAL-148
Bliss Synergy Score
25% Quantile: −5.52 | 75% Quantile: 4.45
512
128
32
8
0
Nedaplatin (μM)
0
10
78
625
5000
Doxorubicin (nM)
−28.93
−33.07
−8.72
−21.54
7.4
1.37
4.34
−0.18
0.31
−4.45
2.45
−1.28
10.27
8.12
4.75
2.4
Synergy Score
40
20
0
−20
−40

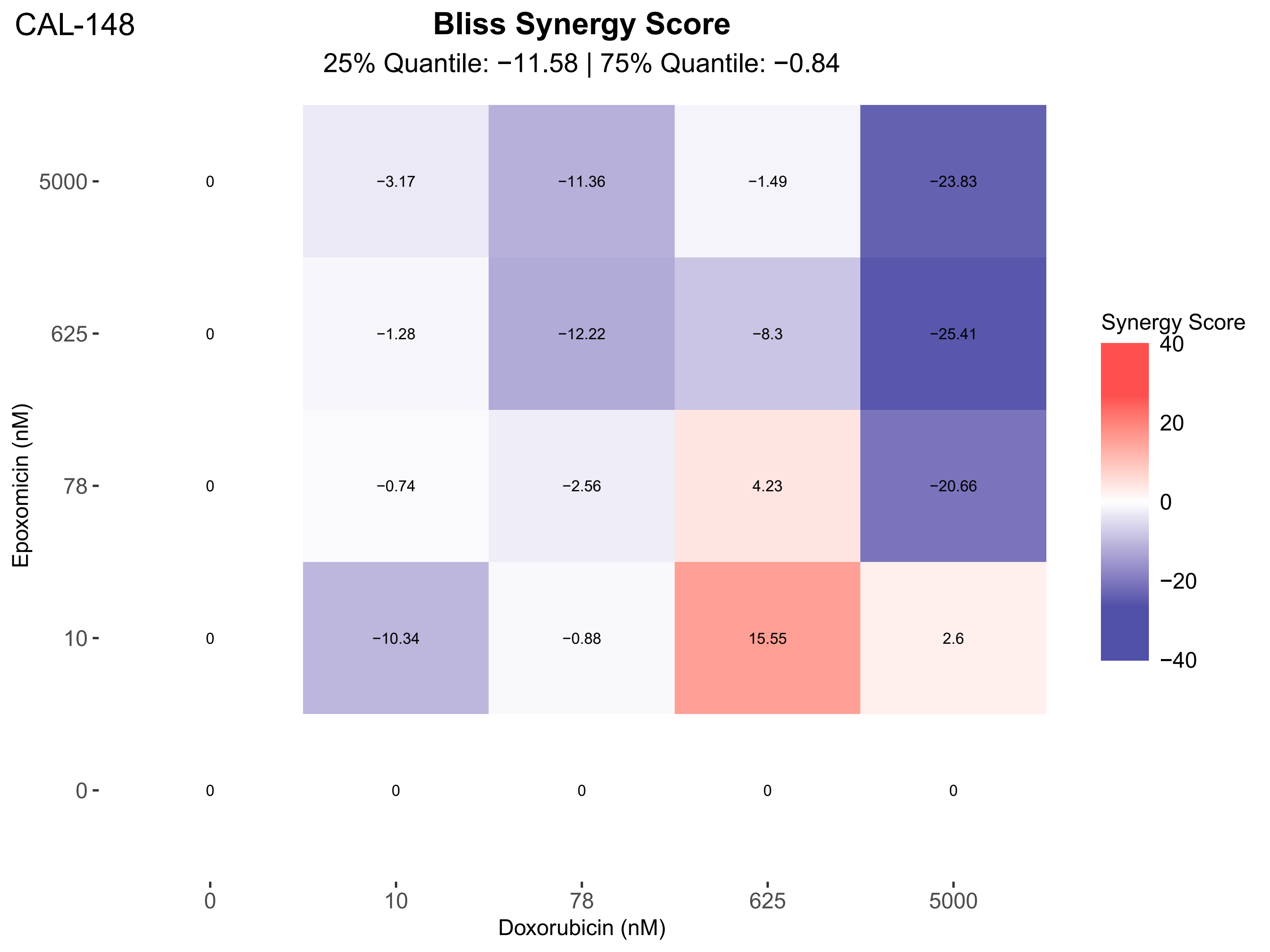

CAL-148
Bliss Synergy Score
25% Quantile: −11.58 | 75% Quantile: −0.84
Epoxomicin (nM)
5000
625
78
10
0
0
−3.17
−11.36
−1.49
−23.83
0
−1.28
−12.22
−8.3
−25.41
0
−0.74
−2.56
4.23
−20.66
0
−10.34
−0.88
15.55
2.6
0
0
0
0
0
0
10
78
625
5000
Doxorubicin (nM)
Synergy Score
40
20
0
−20
−40

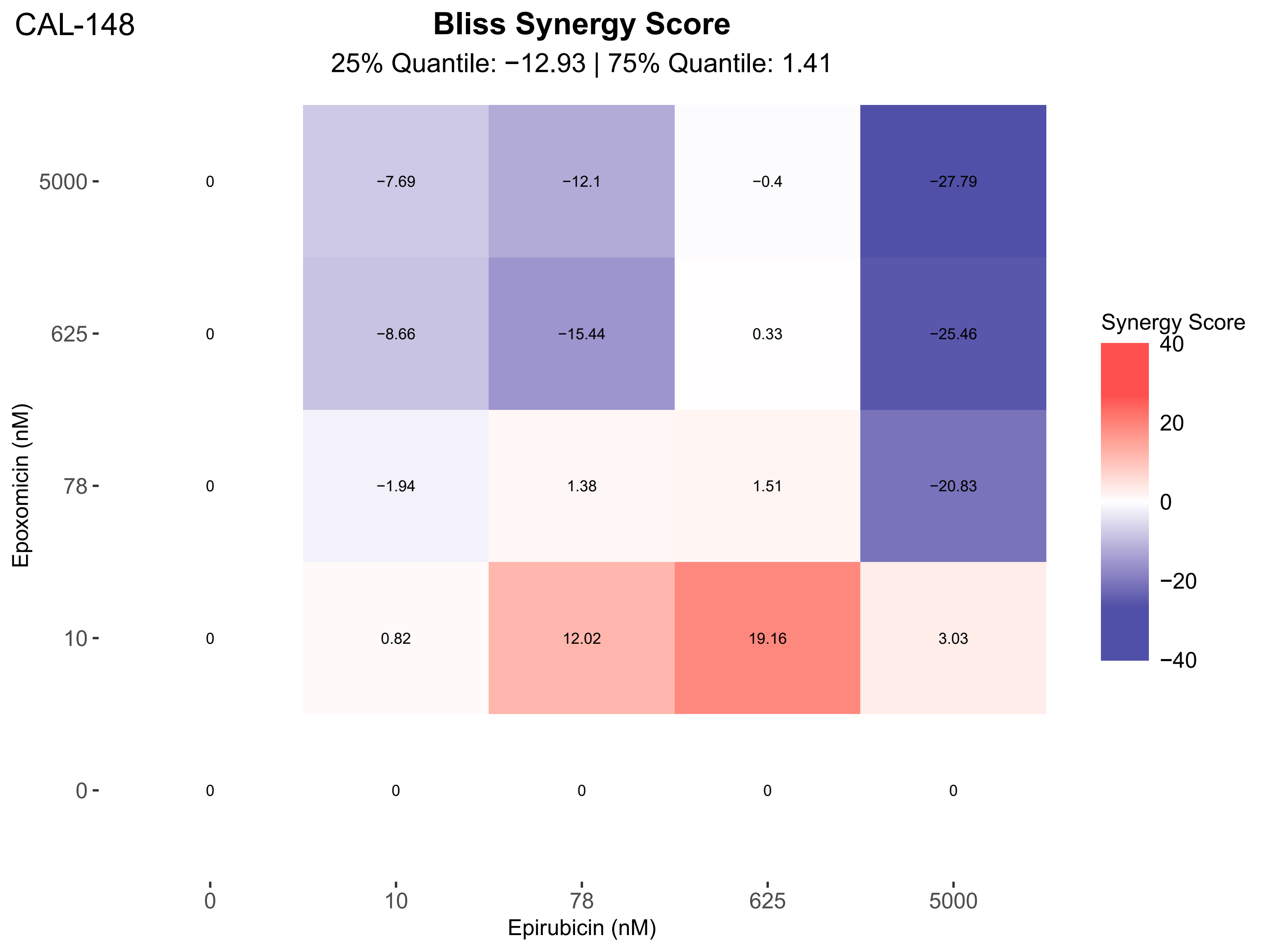
CAL-148
Bliss Synergy Score
25% Quantile: −12.93 | 75% Quantile: 1.41
Epoxomicin (nM)
5000
625
78
10
0
Epirubicin (nM)
0
10
78
625
5000
0 −7.69 −12.1 −0.4 −27.79
0 −8.66 −15.44 0.33 −25.46
0 −1.94 1.38 1.51 −20.83
0 0.82 12.02 19.16 3.03
0 0 0 0 0
Synergy Score
40
20
0
−20
−40

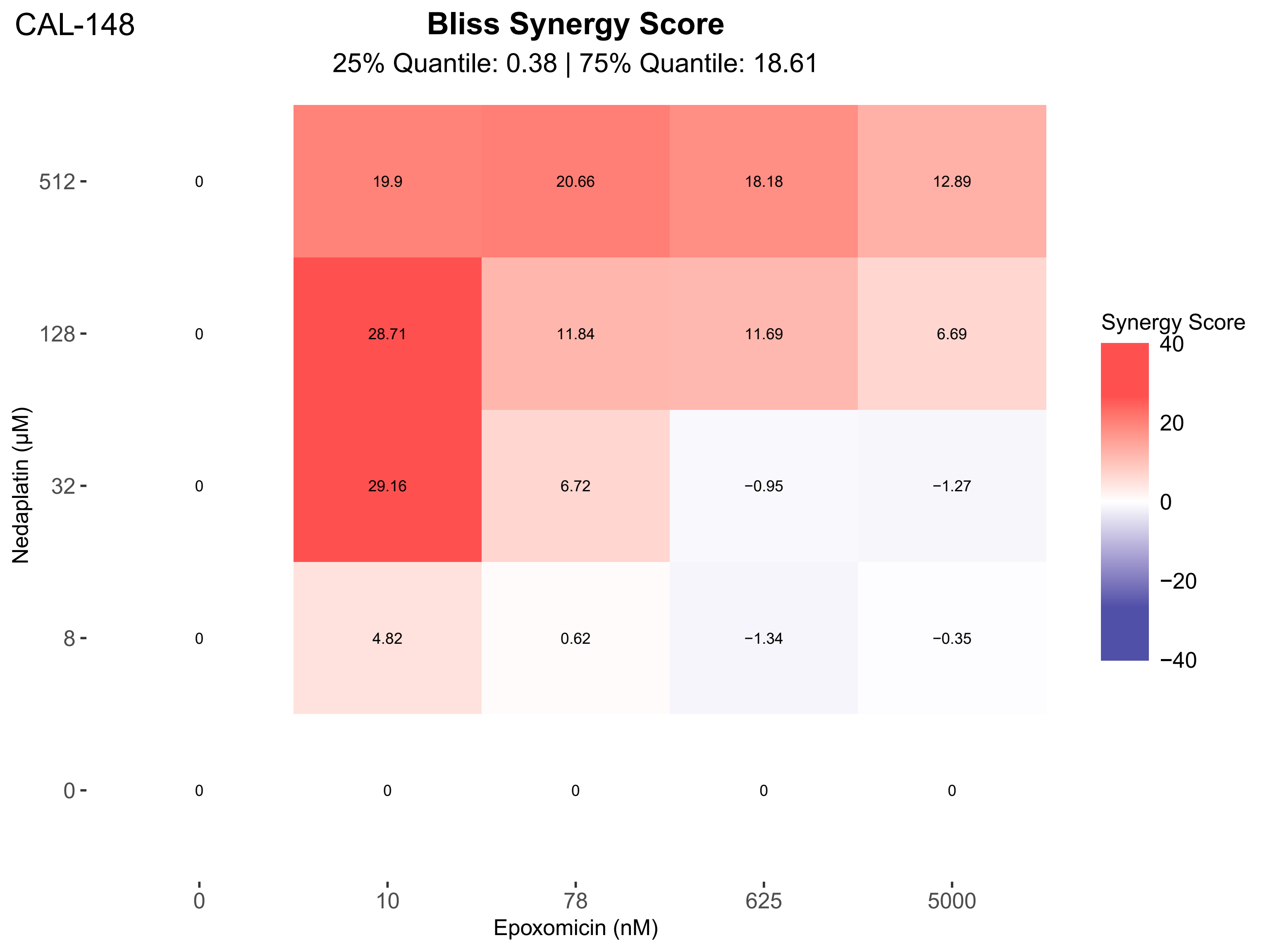

CAL-148
Bliss Synergy Score
25% Quantile: 0.38 | 75% Quantile: 18.61
Nedaplatin (μM)
512
128
32
8
0
Epoxomicin (nM)
0
10
78
625
5000
512: 0, 19.9, 20.66, 18.18, 12.89
128: 0, 28.71, 11.84, 11.69, 6.69
32: 0, 29.16, 6.72, −0.95, −1.27
8: 0, 4.82, 0.62, −1.34, −0.35
0: 0, 0, 0, 0, 0
Synergy Score
40
20
0
−20
−40

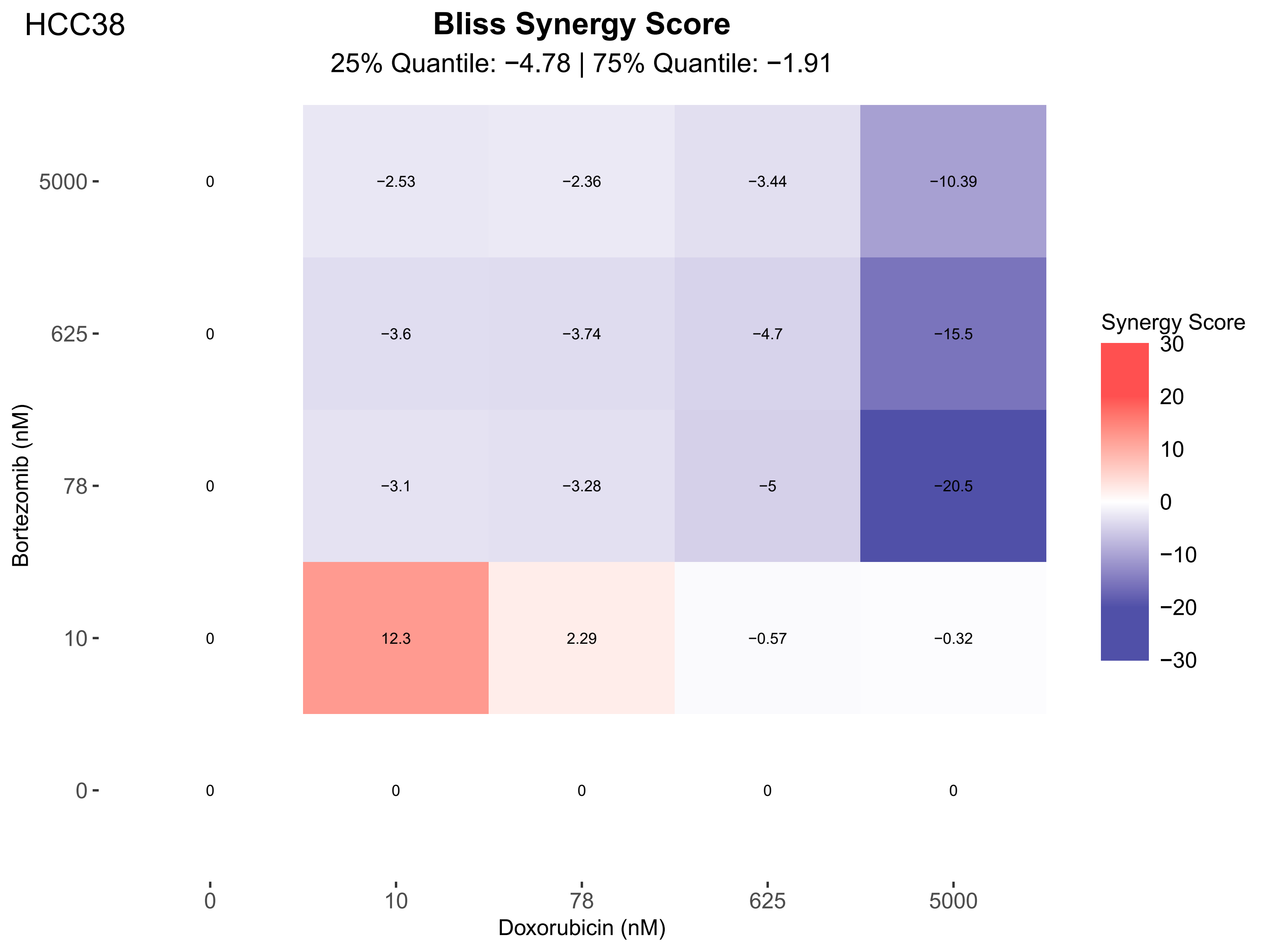
HCC38
Bliss Synergy Score
25% Quantile: −4.78 | 75% Quantile: −1.91
Bortezomib (nM)
5000
625
78
10
0
0
0
0
0
0
−2.53
−2.36
−3.44
−10.39
−3.6
−3.74
−4.7
−15.5
−3.1
−3.28
−5
−20.5
12.3
2.29
−0.57
−0.32
0
0
0
0
Doxorubicin (nM)
0
10
78
625
5000
Synergy Score
30
20
10
0
−10
−20
−30

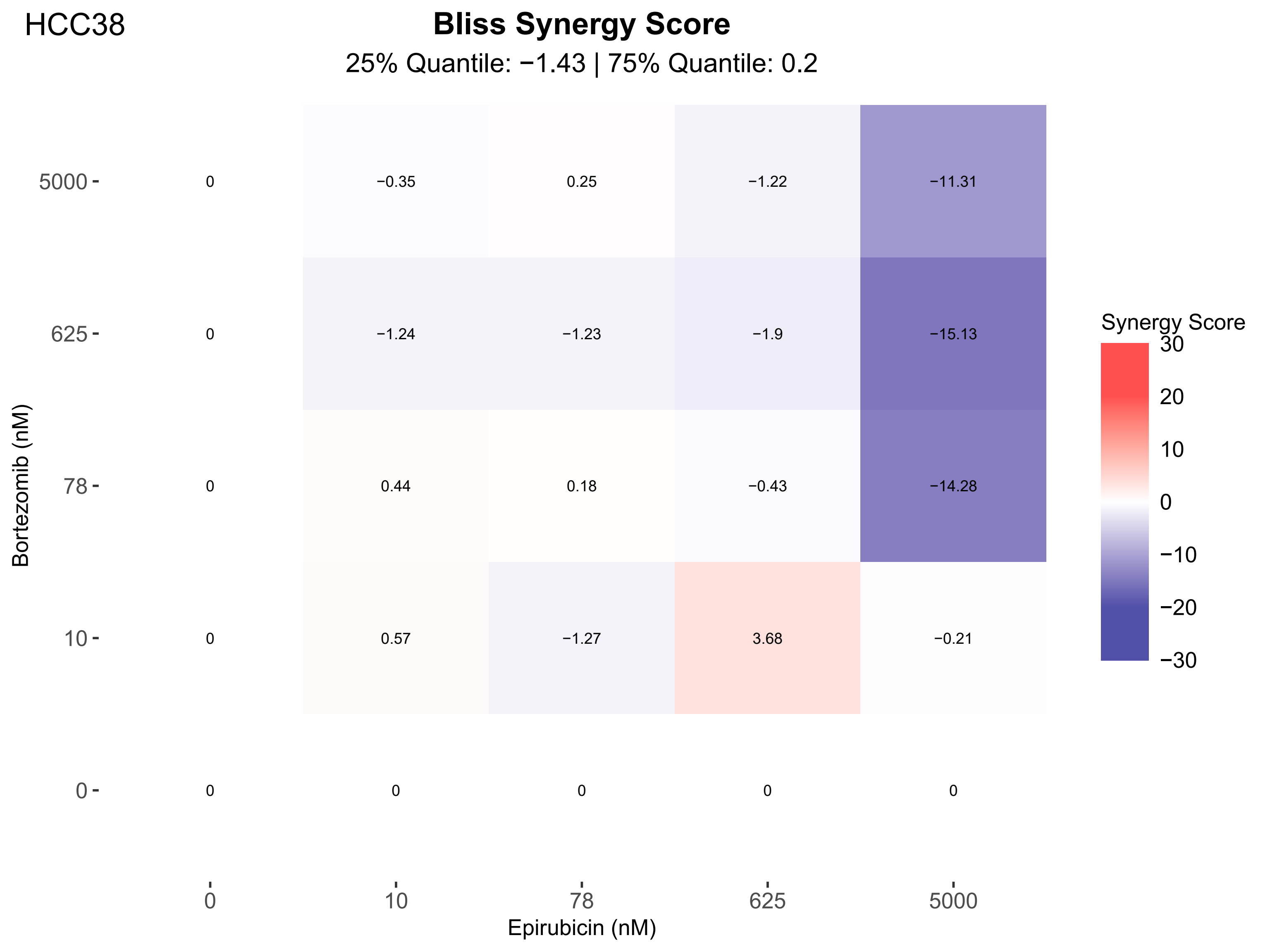
HCC38
Bliss Synergy Score
25% Quantile: −1.43 | 75% Quantile: 0.2
Bortezomib (nM)
5000
625
78
10
0
Epirubicin (nM)
0
10
78
625
5000
0
−0.35
0.25
−1.22
−11.31
0
−1.24
−1.23
−1.9
−15.13
0
0.44
0.18
−0.43
−14.28
0
0.57
−1.27
3.68
−0.21
0
0
0
0
0
Synergy Score
30
20
10
0
−10
−20
−30

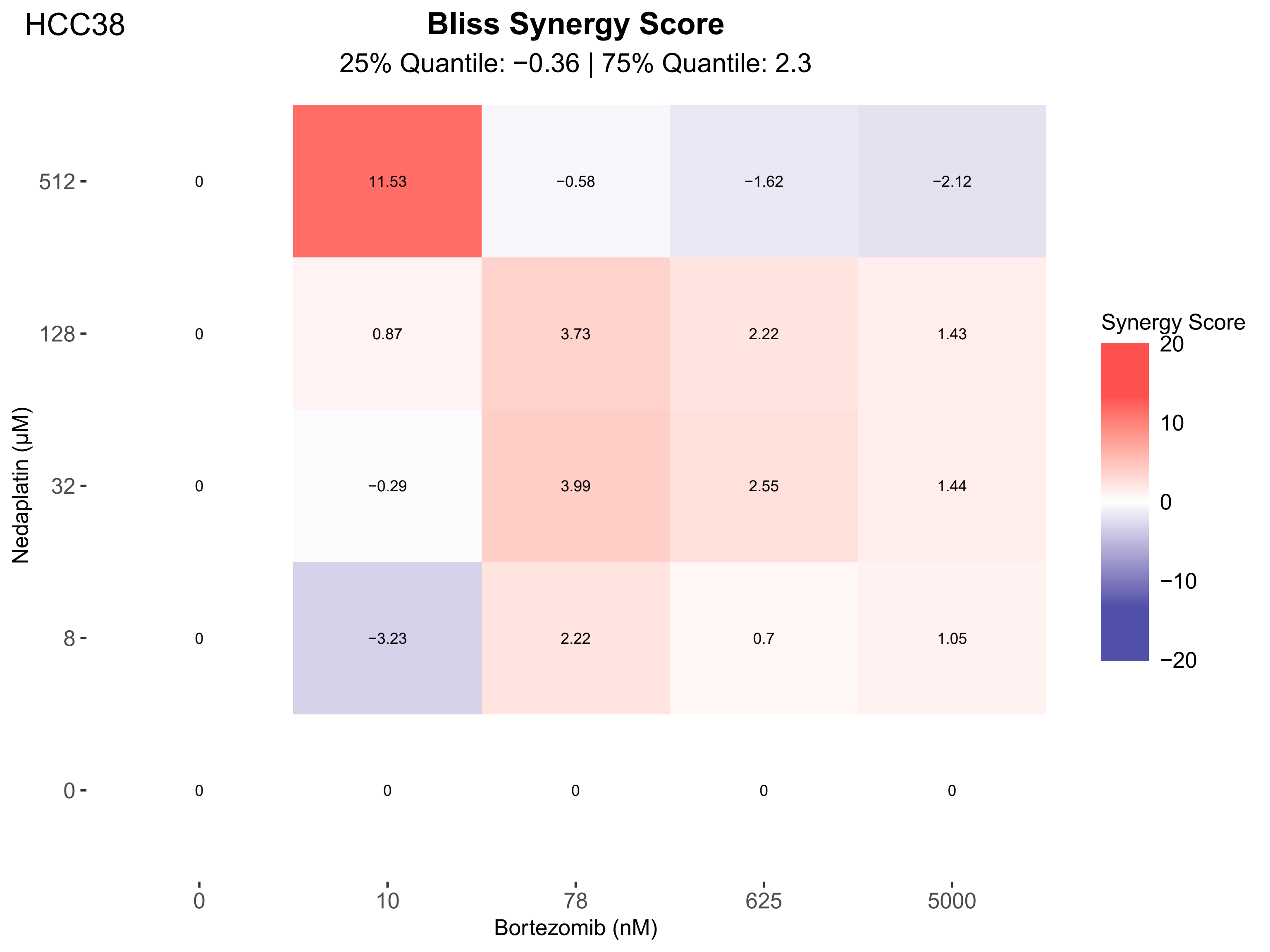
HCC38
Bliss Synergy Score
25% Quantile: −0.36 | 75% Quantile: 2.3
512
128
32
8
0
0
0
0
0
0
11.53
−0.58
−1.62
−2.12
0.87
3.73
2.22
1.43
−0.29
3.99
2.55
1.44
−3.23
2.22
0.7
1.05
0
0
0
0
Nedaplatin (μM)
0
10
78
625
5000
Bortezomib (nM)
Synergy Score
20
10
0
−10
−20

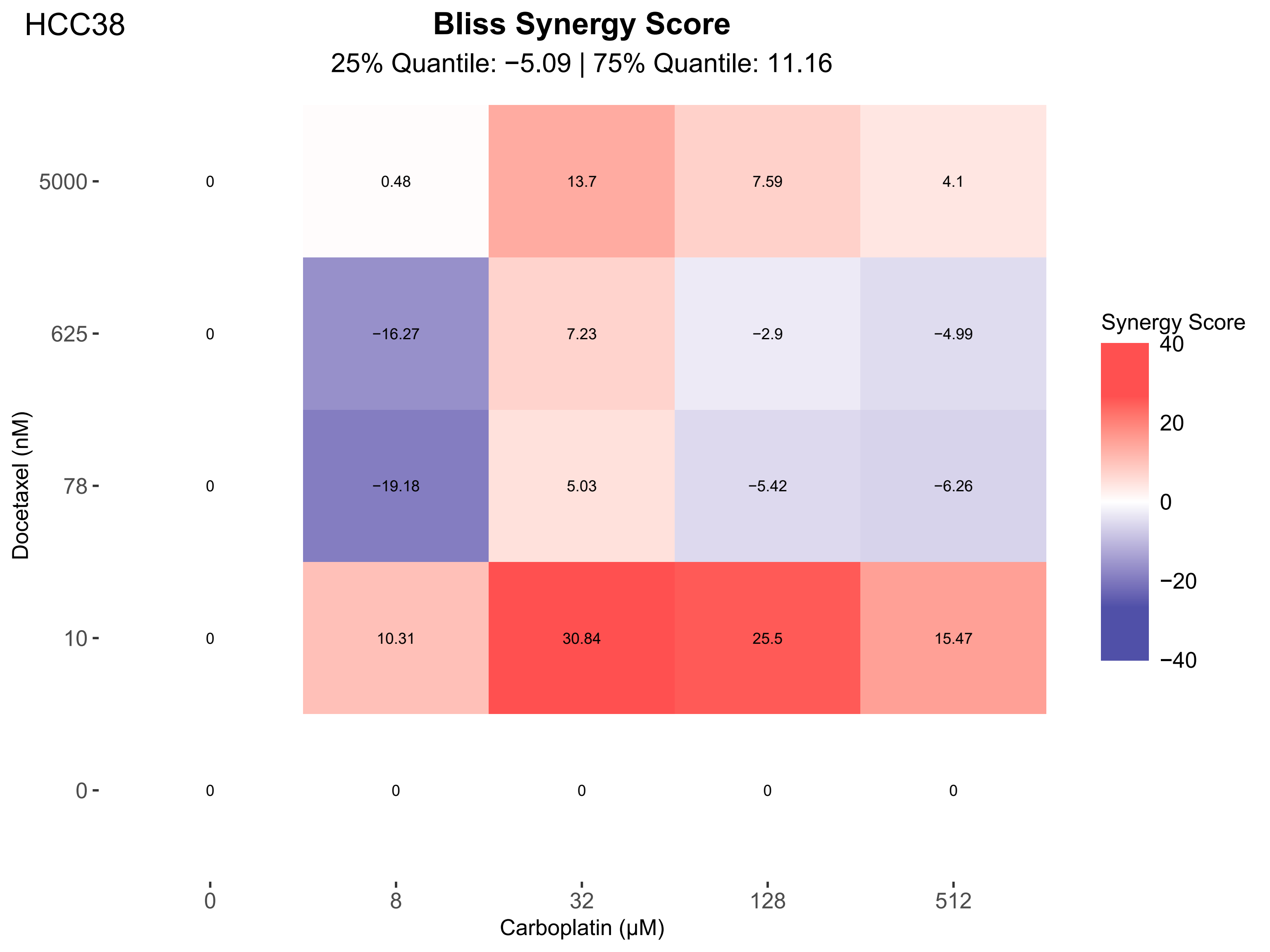
HCC38
Bliss Synergy Score
25% Quantile: −5.09 | 75% Quantile: 11.16
Docetaxel (nM)
5000
625
78
10
0
0
0.48
13.7
7.59
4.1
0
−16.27
7.23
−2.9
−4.99
0
−19.18
5.03
−5.42
−6.26
0
10.31
30.84
25.5
15.47
0
0
0
0
0
0
8
32
128
512
Carboplatin (μM)
Synergy Score
40
20
0
−20
−40

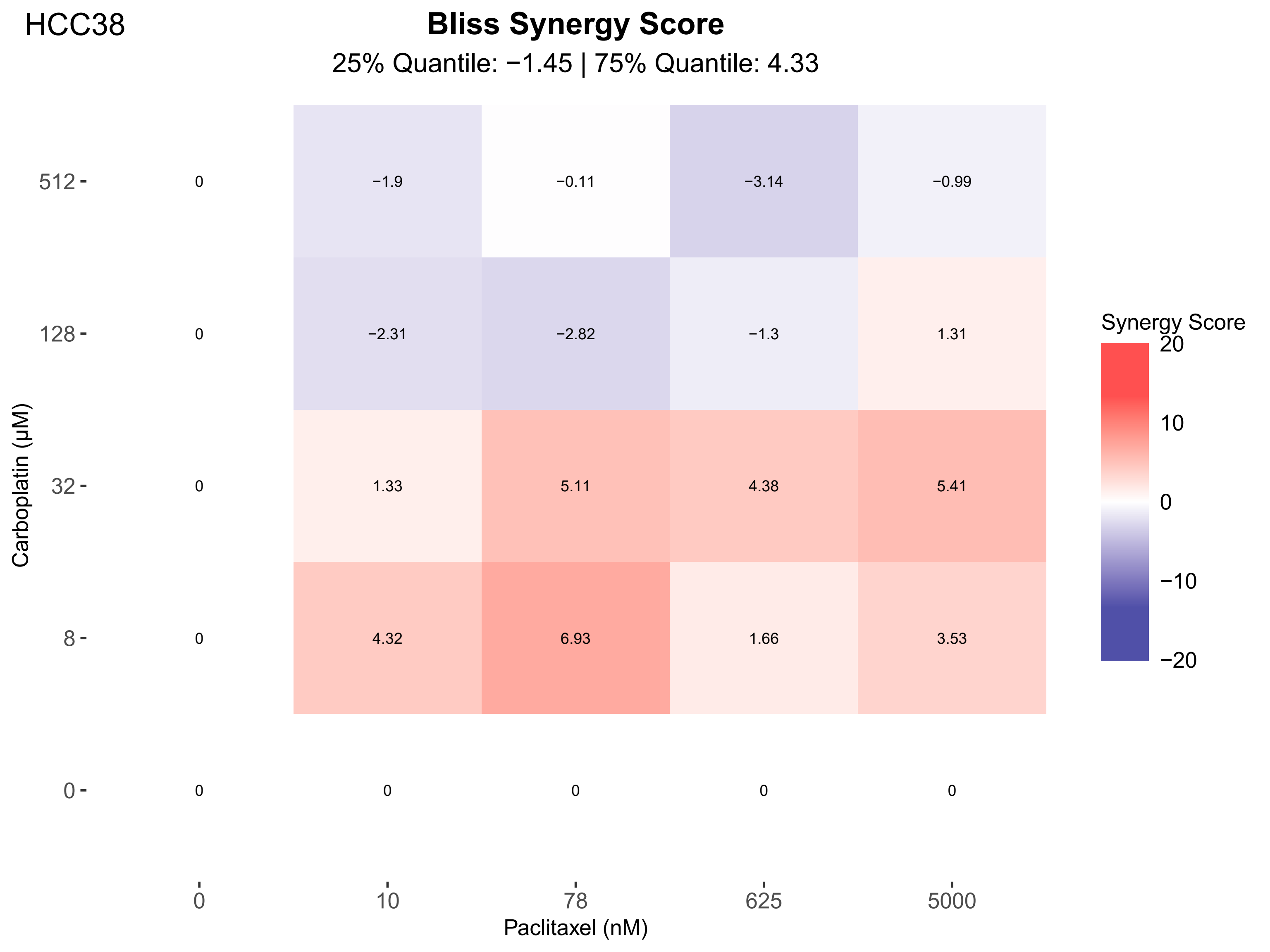
HCC38
Bliss Synergy Score
25% Quantile: −1.45 | 75% Quantile: 4.33
Carboplatin (µM)
512
128
32
8
0
Paclitaxel (nM)
0
10
78
625
5000
−1.9
−0.11
−3.14
−0.99
−2.31
−2.82
−1.3
1.31
1.33
5.11
4.38
5.41
4.32
6.93
1.66
3.53
Synergy Score
20
10
0
−10
−20

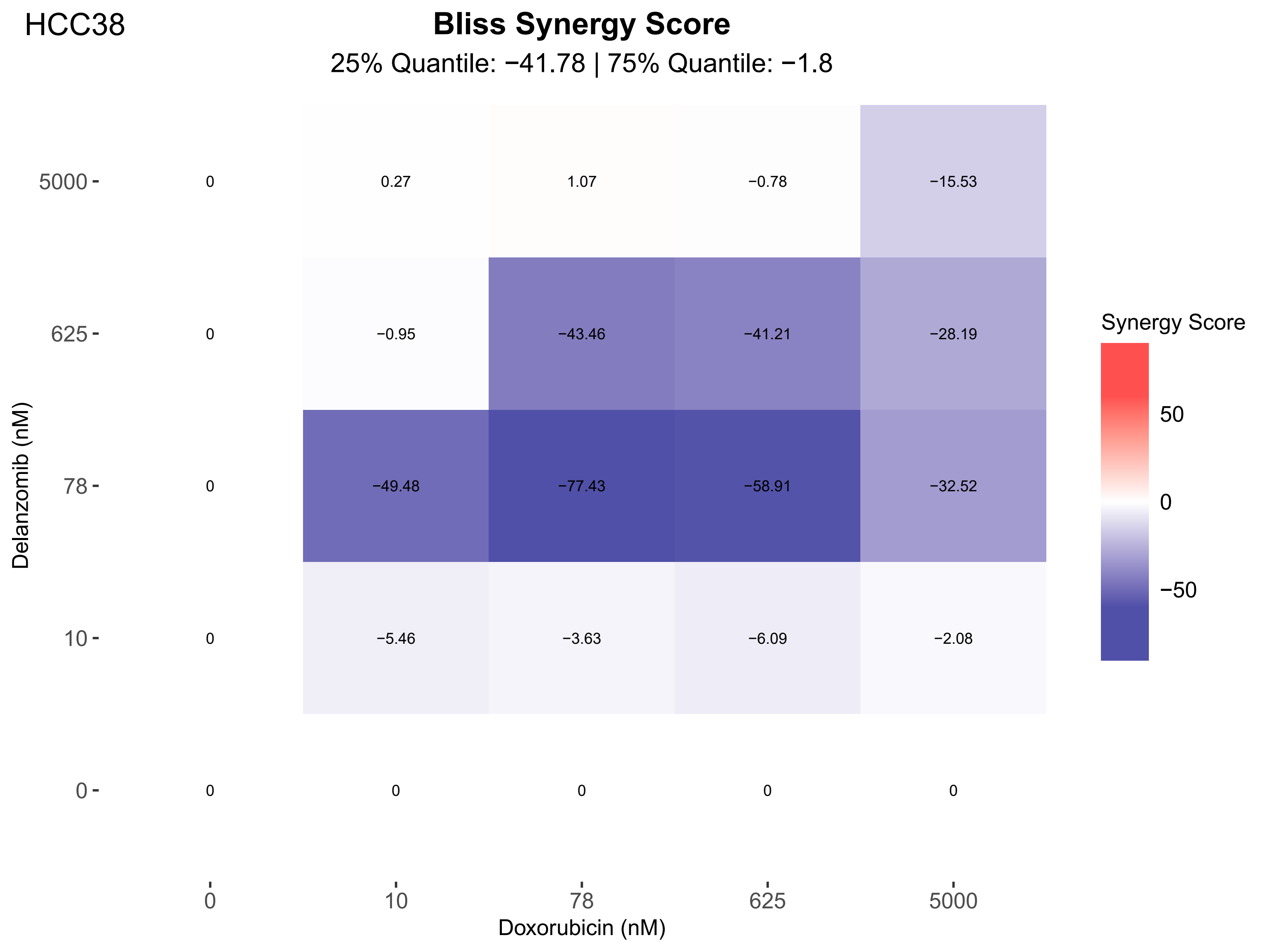

HCC38
Bliss Synergy Score
25% Quantile: −41.78 | 75% Quantile: −1.8
Delanzomib (nM)
5000
625
78
10
0
0
0.27
1.07
−0.78
−15.53
0
−0.95
−43.46
−41.21
−28.19
0
−49.48
−77.43
−58.91
−32.52
0
−5.46
−3.63
−6.09
−2.08
0
0
0
0
0
0
10
78
625
5000
Doxorubicin (nM)
Synergy Score
50
0
−50

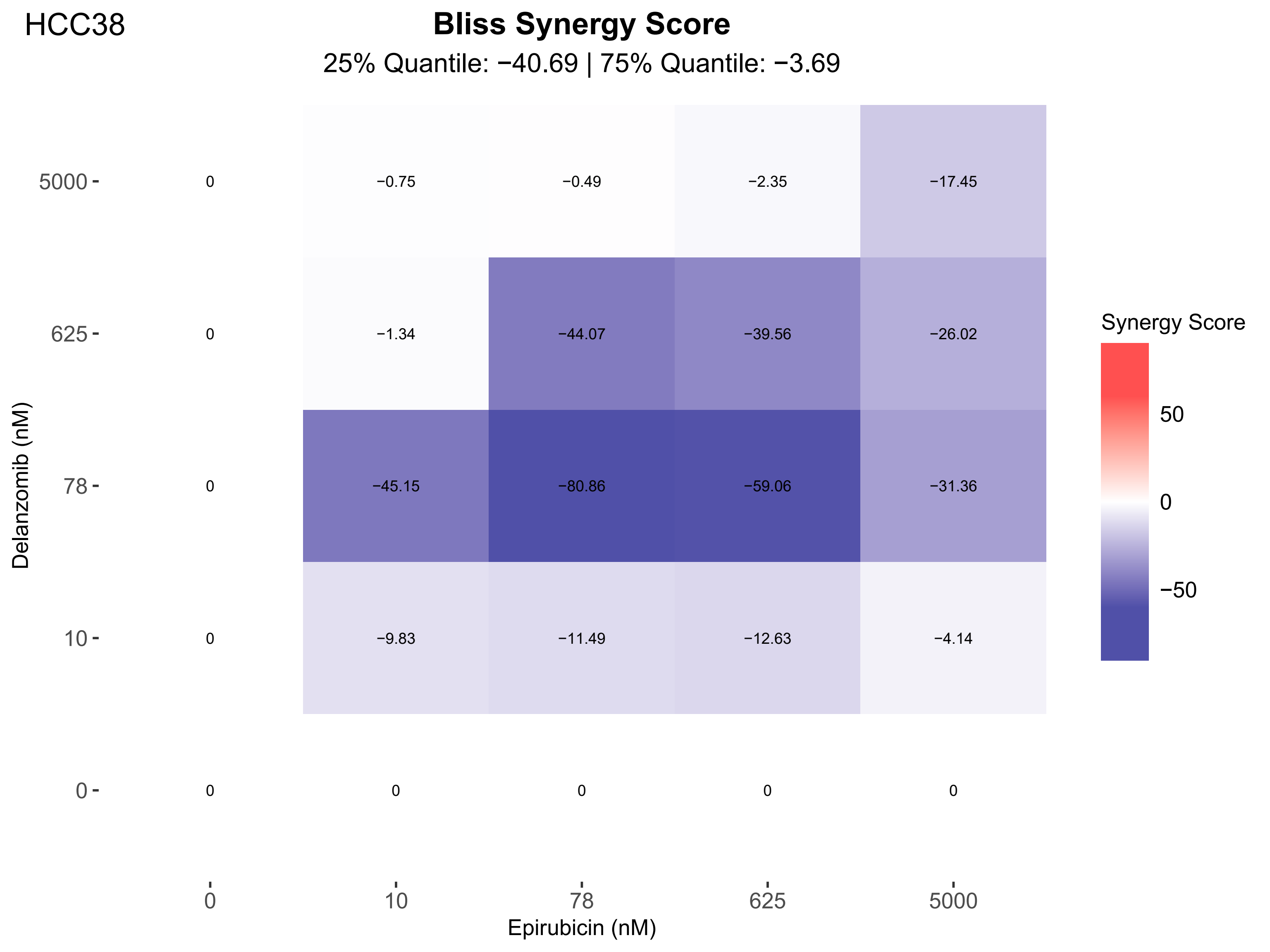

HCC38
Bliss Synergy Score
25% Quantile: −40.69 | 75% Quantile: −3.69
Delanzomib (nM)
Epirubicin (nM)
5000
625
78
10
0
0
10
78
625
5000
0
−0.75
−0.49
−2.35
−17.45
0
−1.34
−44.07
−39.56
−26.02
0
−45.15
−80.86
−59.06
−31.36
0
−9.83
−11.49
−12.63
−4.14
0
0
0
0
0
Synergy Score
50
0
−50

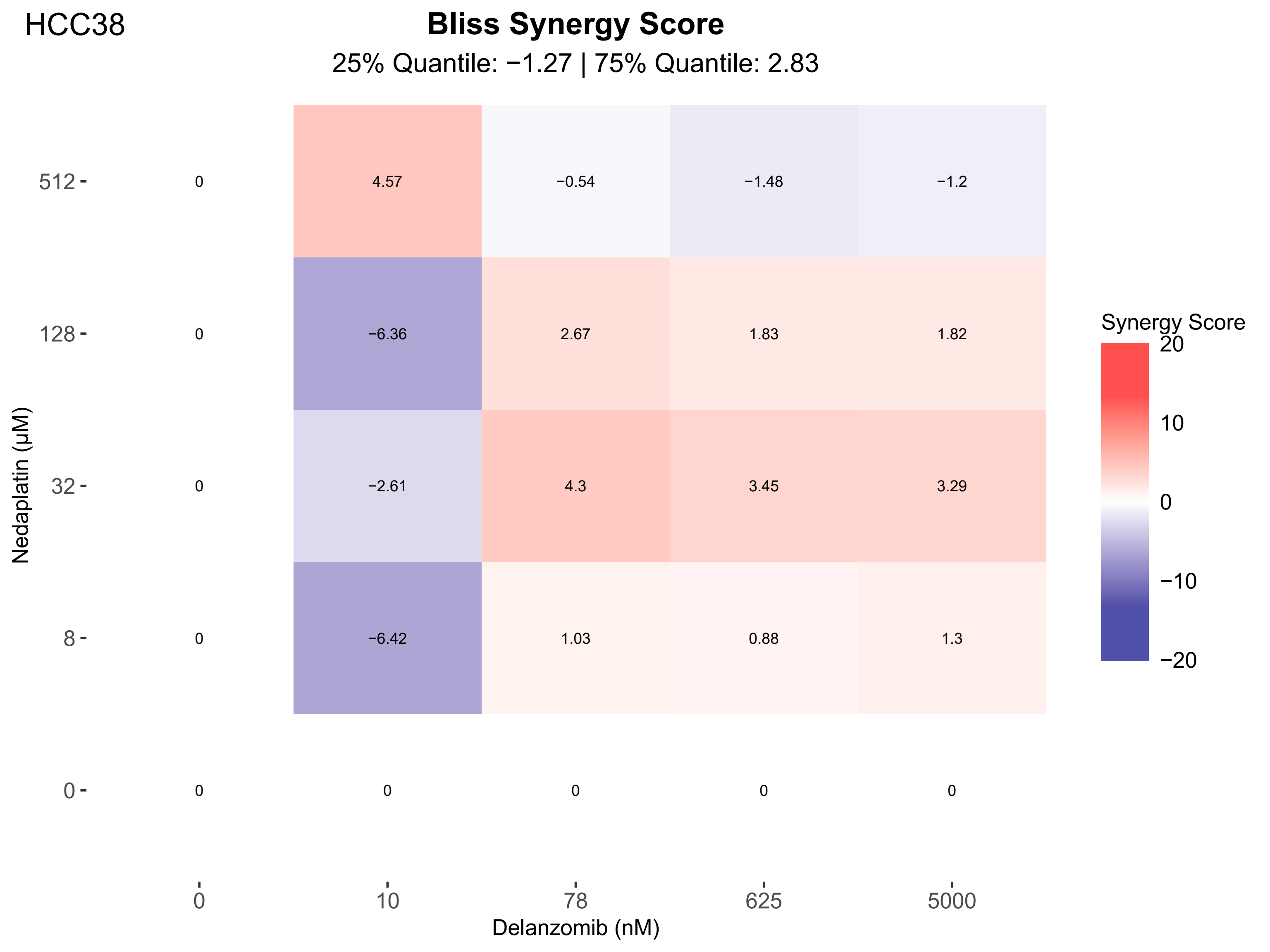

HCC38
Bliss Synergy Score
25% Quantile: −1.27 | 75% Quantile: 2.83
Nedaplatin (μM)
512
128
32
8
0
0
4.57
−0.54
−1.48
−1.2
0
−6.36
2.67
1.83
1.82
0
−2.61
4.3
3.45
3.29
0
−6.42
1.03
0.88
1.3
0
0
0
0
0
0
10
78
625
5000
Delanzomib (nM)
Synergy Score
20
10
0
−10
−20

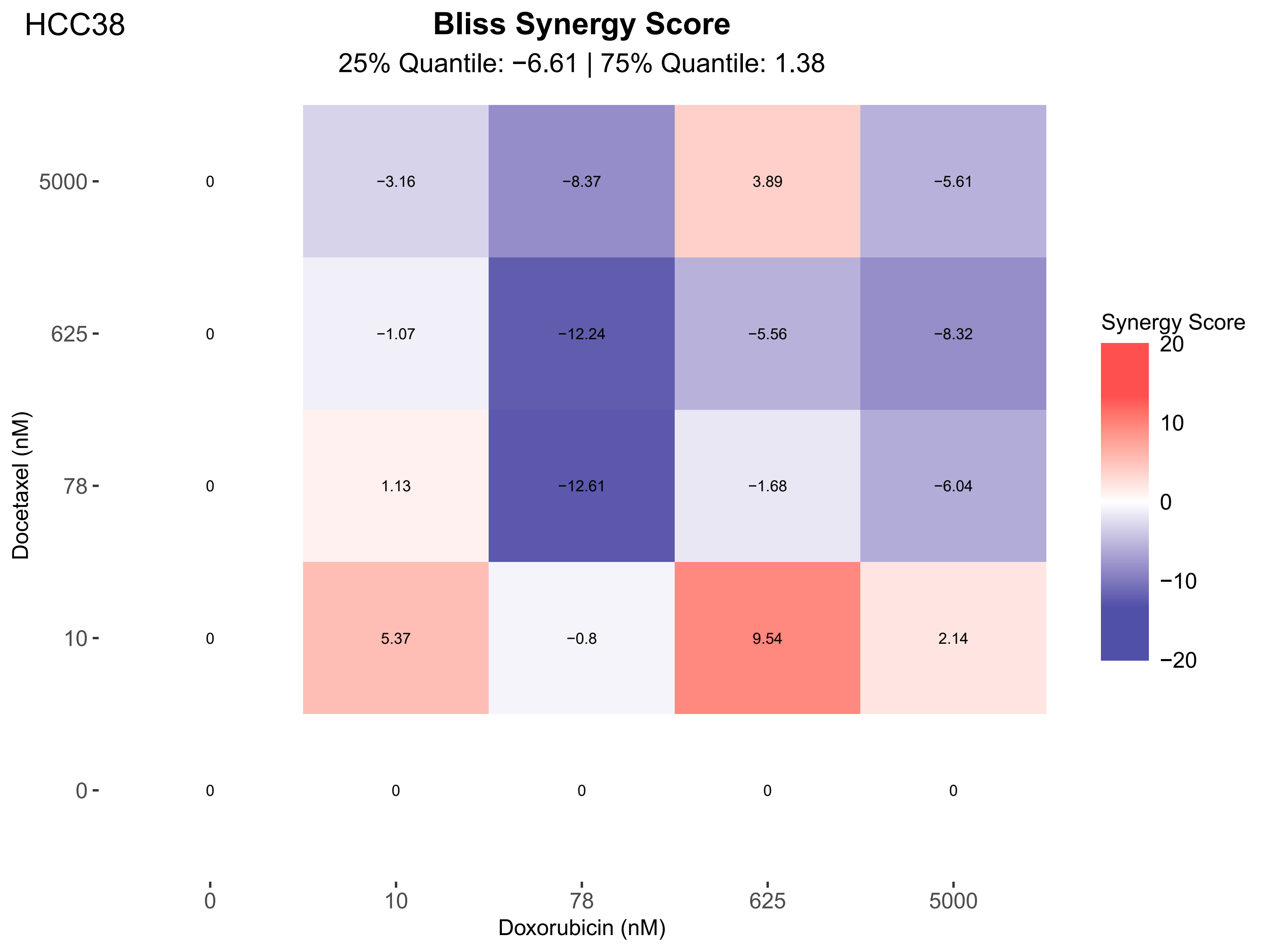
HCC38
Bliss Synergy Score
25% Quantile: −6.61 | 75% Quantile: 1.38
Docetaxel (nM)
5000
625
78
10
0
Doxorubicin (nM)
0
10
78
625
5000
0
−3.16
−8.37
3.89
−5.61
0
−1.07
−12.24
−5.56
−8.32
0
1.13
−12.61
−1.68
−6.04
0
5.37
−0.8
9.54
2.14
0
0
0
0
0
Synergy Score
20
10
0
−10
−20

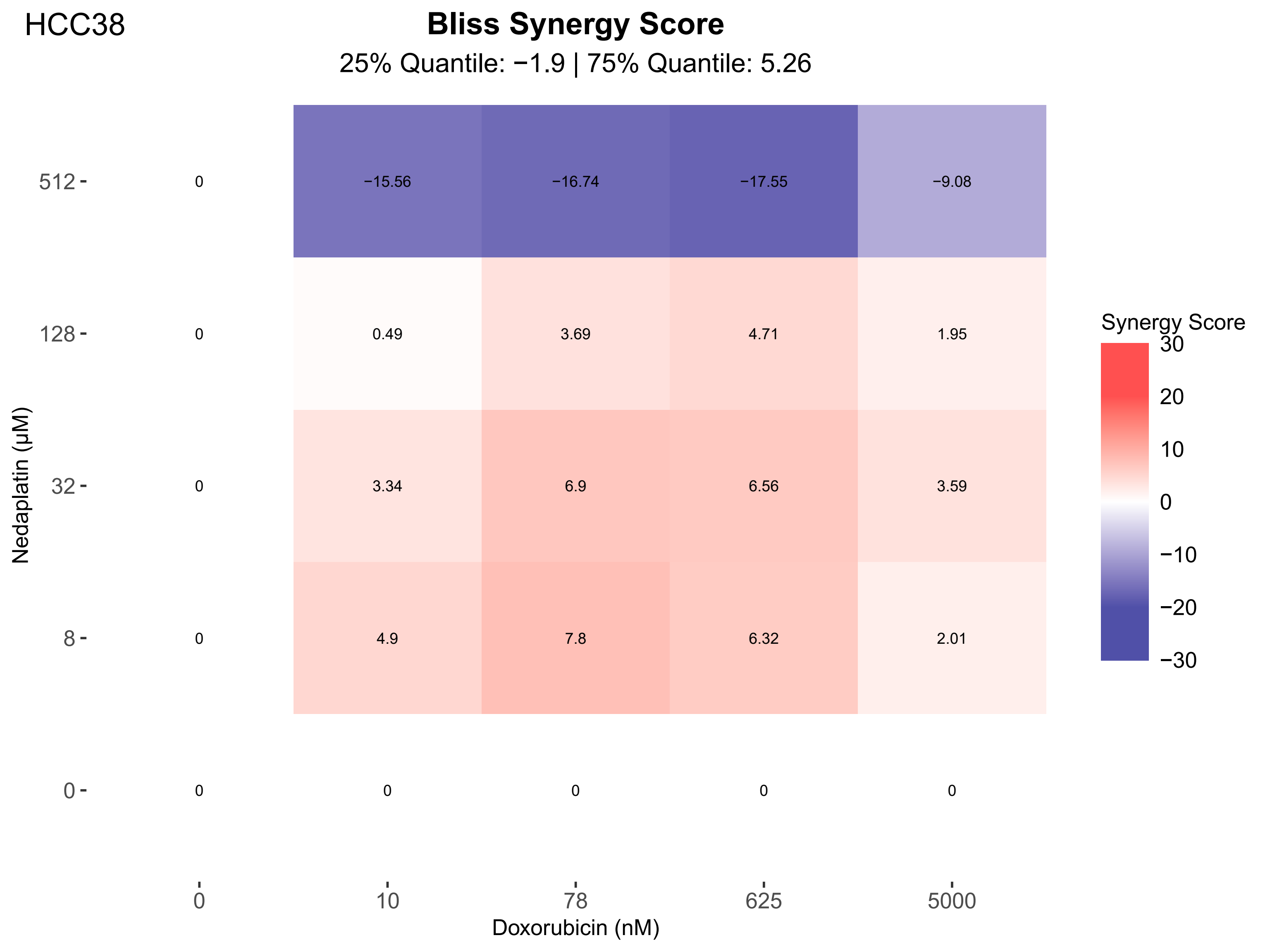
HCC38
Bliss Synergy Score
25% Quantile: −1.9 | 75% Quantile: 5.26
Nedaplatin (μM)
512
128
32
8
0
Doxorubicin (nM)
0
10
78
625
5000
−15.56
−16.74
−17.55
−9.08
0.49
3.69
4.71
1.95
3.34
6.9
6.56
3.59
4.9
7.8
6.32
2.01
Synergy Score
30
20
10
0
−10
−20
−30

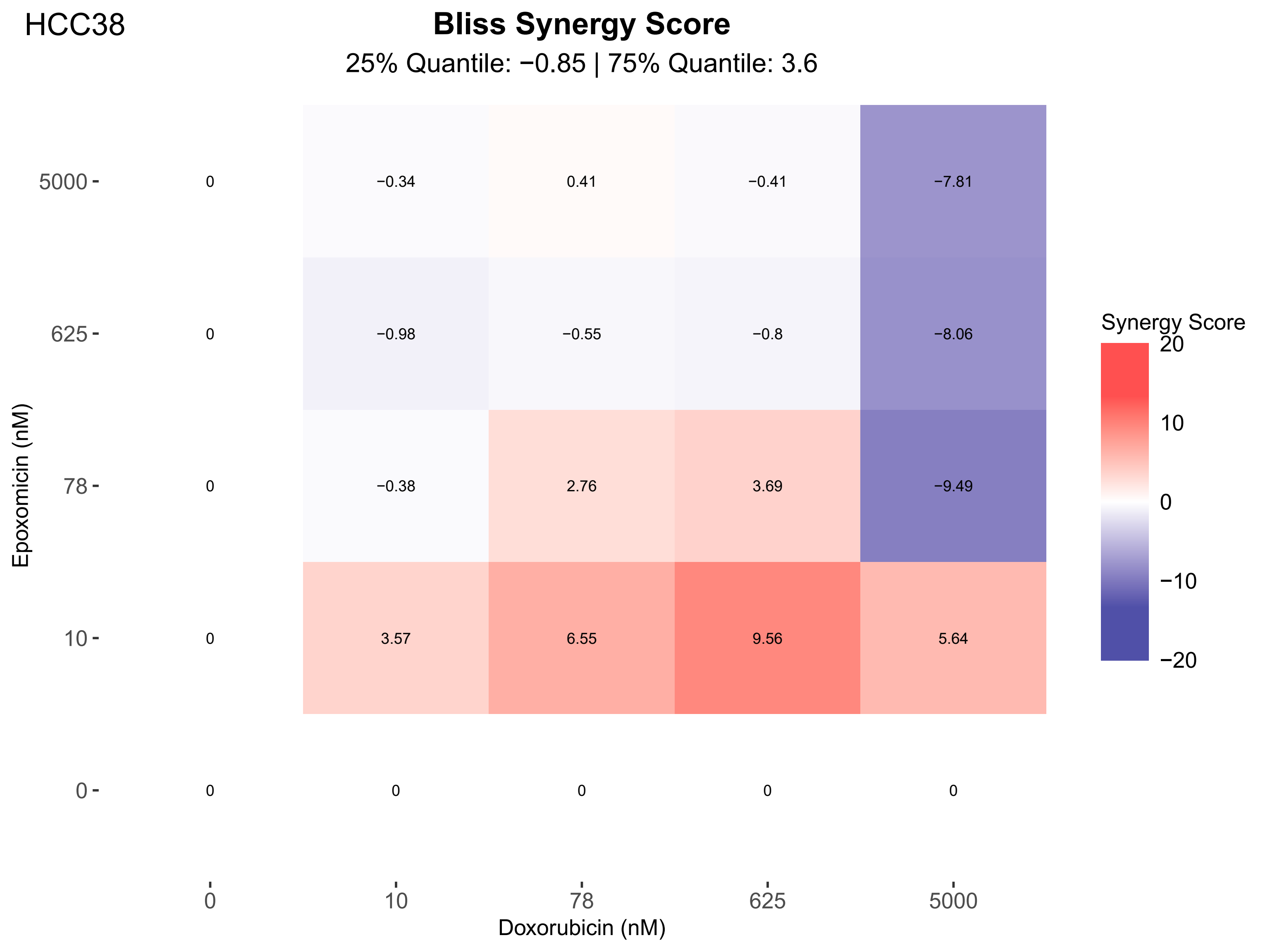
HCC38
Bliss Synergy Score
25% Quantile: −0.85 | 75% Quantile: 3.6
Epoxomicin (nM)
5000
625
78
10
0
Doxorubicin (nM)
0
10
78
625
5000
0
−0.34
0.41
−0.41
−7.81
0
−0.98
−0.55
−0.8
−8.06
0
−0.38
2.76
3.69
−9.49
0
3.57
6.55
9.56
5.64
0
0
0
0
0
Synergy Score
20
10
0
−10
−20

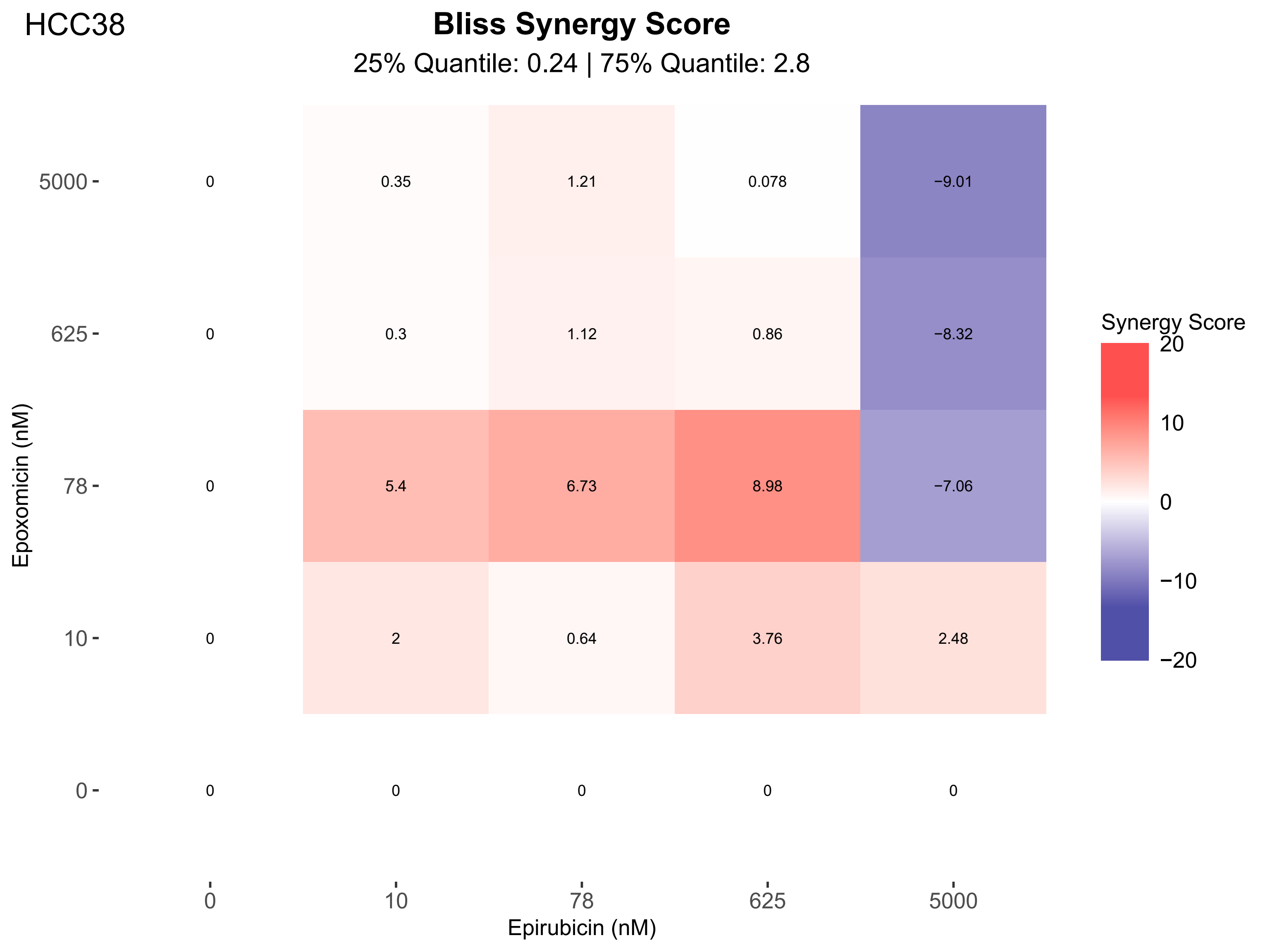
HCC38
Bliss Synergy Score
25% Quantile: 0.24 | 75% Quantile: 2.8
Epoxomicin (nM)
5000
625
78
10
0
0 0.35 1.21 0.078 −9.01
0 0.3 1.12 0.86 −8.32
0 5.4 6.73 8.98 −7.06
0 2 0.64 3.76 2.48
0 0 0 0 0
0
10
78
625
5000
Epirubicin (nM)
Synergy Score
20
10
0
−10
−20

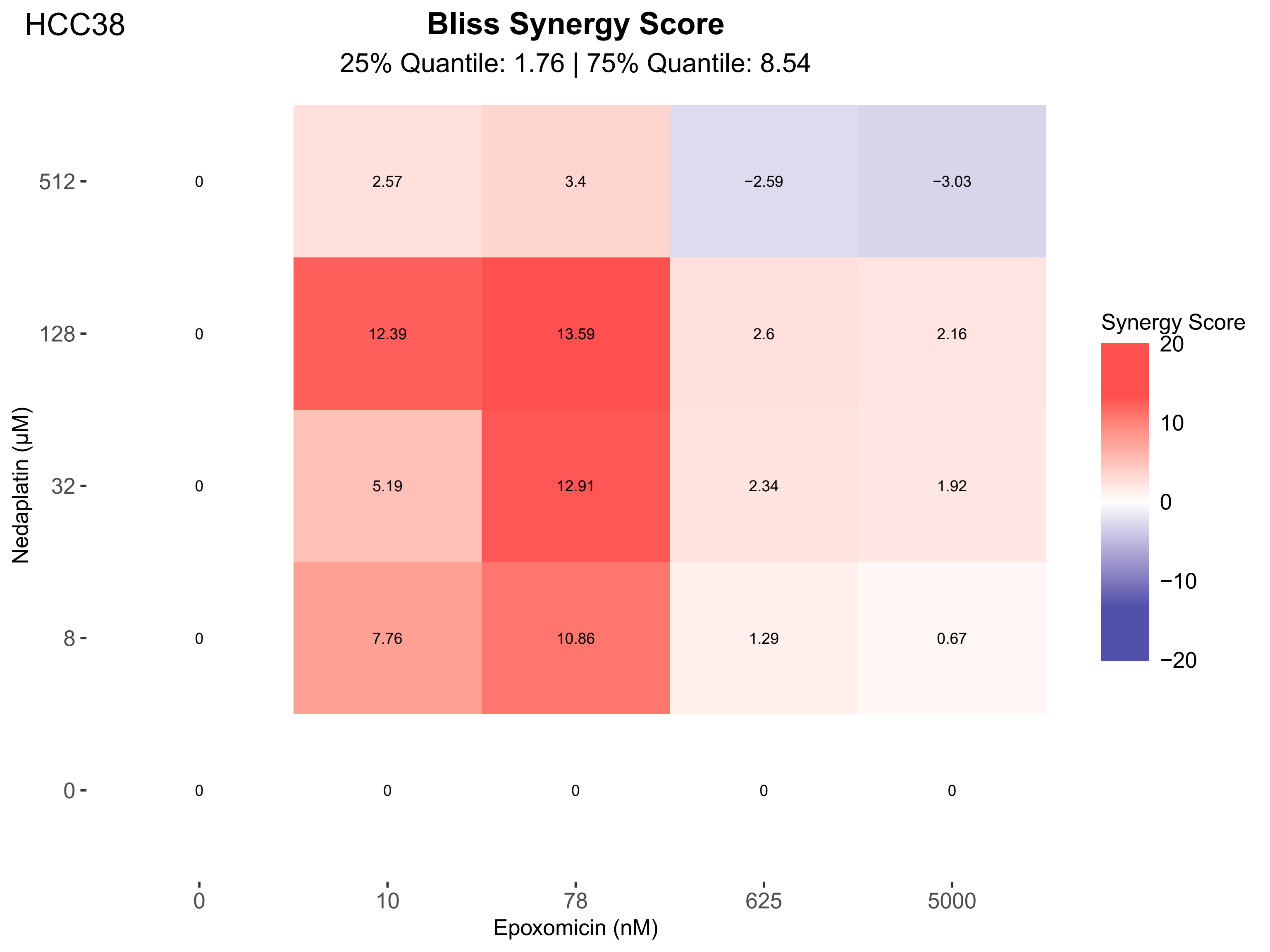
HCC38
Bliss Synergy Score
25% Quantile: 1.76 | 75% Quantile: 8.54
Nedaplatin (μM)
512
128
32
8
0
0
2.57
3.4
−2.59
−3.03
0
12.39
13.59
2.6
2.16
0
5.19
12.91
2.34
1.92
0
7.76
10.86
1.29
0.67
0
0
0
0
0
0
10
78
625
5000
Epoxomicin (nM)
Synergy Score
20
10
0
−10
−20

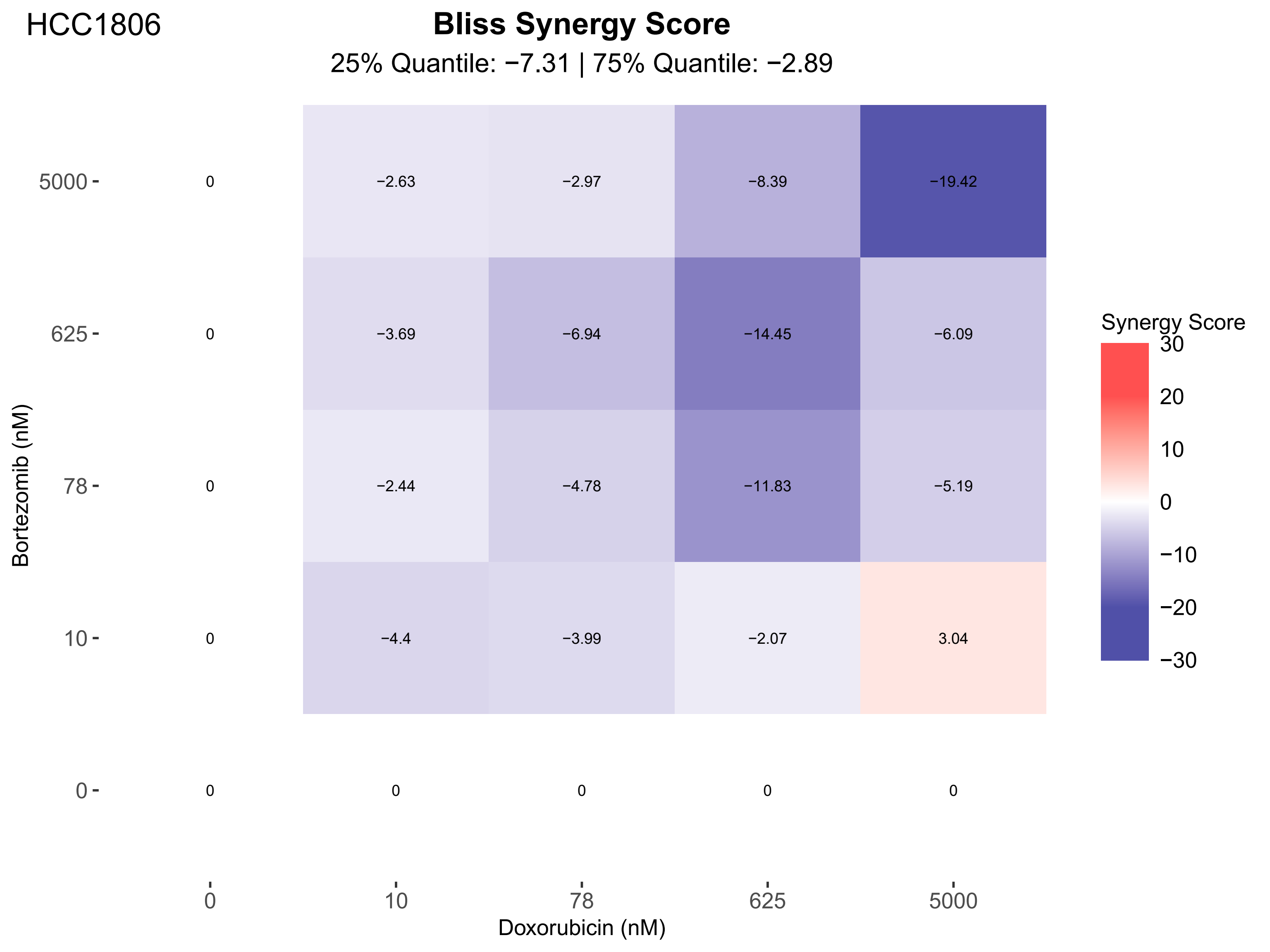
HCC1806
Bliss Synergy Score
25% Quantile: −7.31 | 75% Quantile: −2.89
Bortezomib (nM)
5000
625
78
10
0
0
−2.63
−2.97
−8.39
−19.42
0
−3.69
−6.94
−14.45
−6.09
0
−2.44
−4.78
−11.83
−5.19
0
−4.4
−3.99
−2.07
3.04
0
0
0
0
0
0
10
78
625
5000
Doxorubicin (nM)
Synergy Score
30
20
10
0
−10
−20
−30

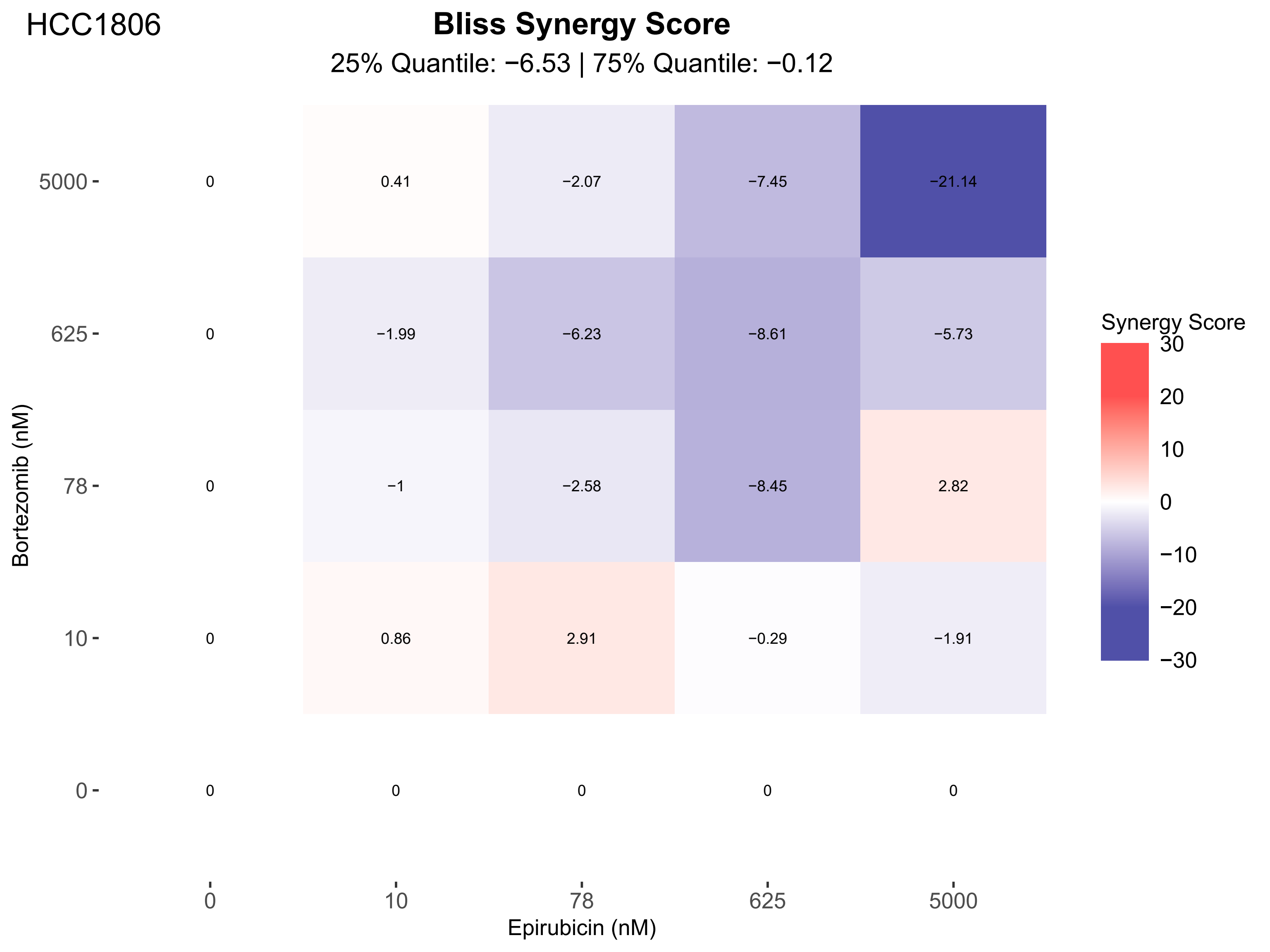
HCC1806
Bliss Synergy Score
25% Quantile: −6.53 | 75% Quantile: −0.12
Bortezomib (nM)
5000
625
78
10
0
0
0.41
−2.07
−7.45
−21.14
0
−1.99
−6.23
−8.61
−5.73
0
−1
−2.58
−8.45
2.82
0
0.86
2.91
−0.29
−1.91
0
0
0
0
0
0
10
78
625
5000
Epirubicin (nM)
Synergy Score
30
20
10
0
−10
−20
−30

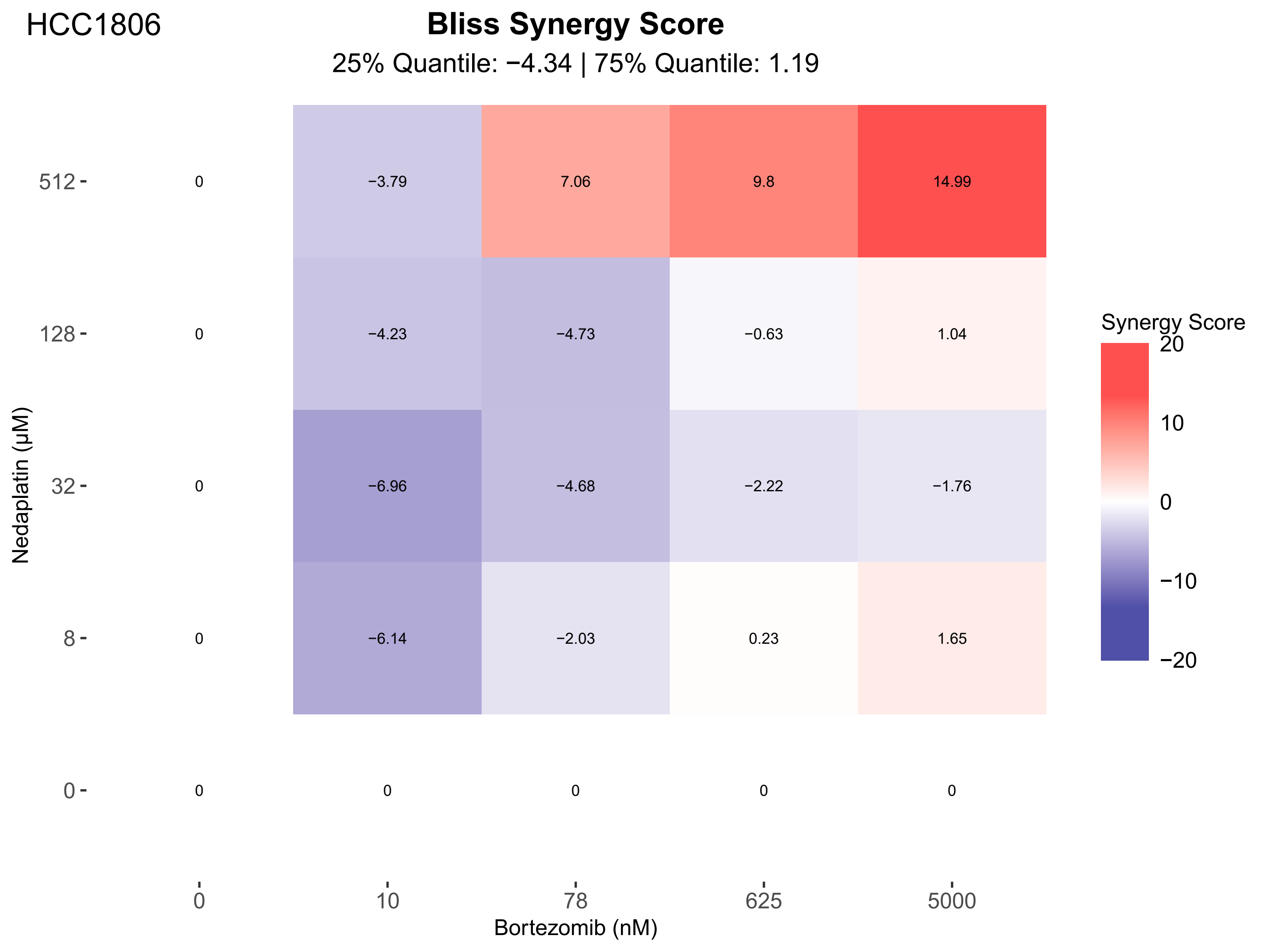
HCC1806
Bliss Synergy Score
25% Quantile: −4.34 | 75% Quantile: 1.19
Nedaplatin (μM)
512
128
32
8
0
0
−3.79
7.06
9.8
14.99
0
−4.23
−4.73
−0.63
1.04
0
−6.96
−4.68
−2.22
−1.76
0
−6.14
−2.03
0.23
1.65
0
0
0
0
0
0
10
78
625
5000
Bortezomib (nM)
Synergy Score
20
10
0
−10
−20

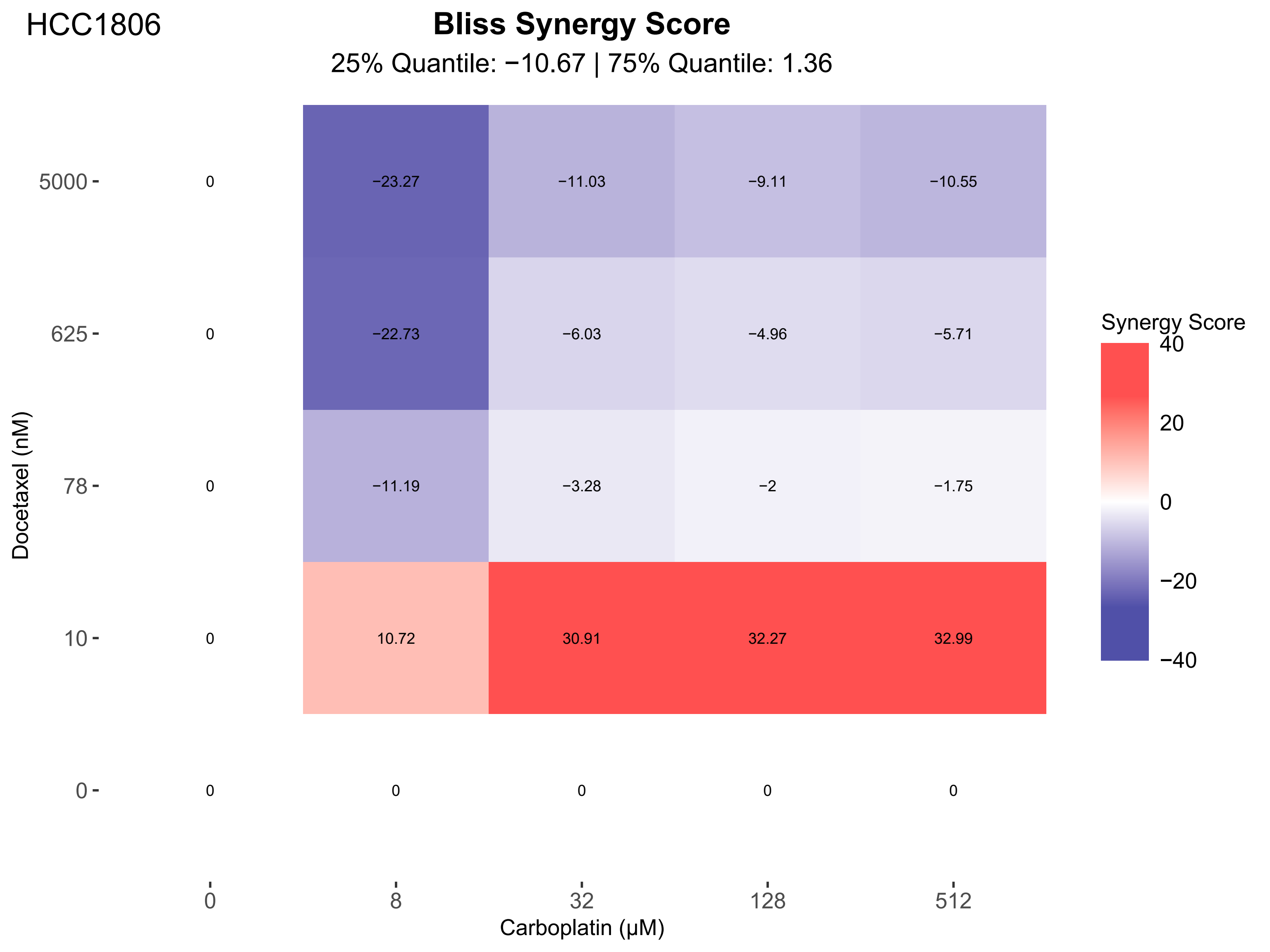
HCC1806
Bliss Synergy Score
25% Quantile: −10.67 | 75% Quantile: 1.36
Docetaxel (nM)
5000
625
78
10
0
Carboplatin (μM)
0
8
32
128
512
0
−23.27
−11.03
−9.11
−10.55
0
−22.73
−6.03
−4.96
−5.71
0
−11.19
−3.28
−2
−1.75
0
10.72
30.91
32.27
32.99
0
0
0
0
0
Synergy Score
40
20
0
−20
−40

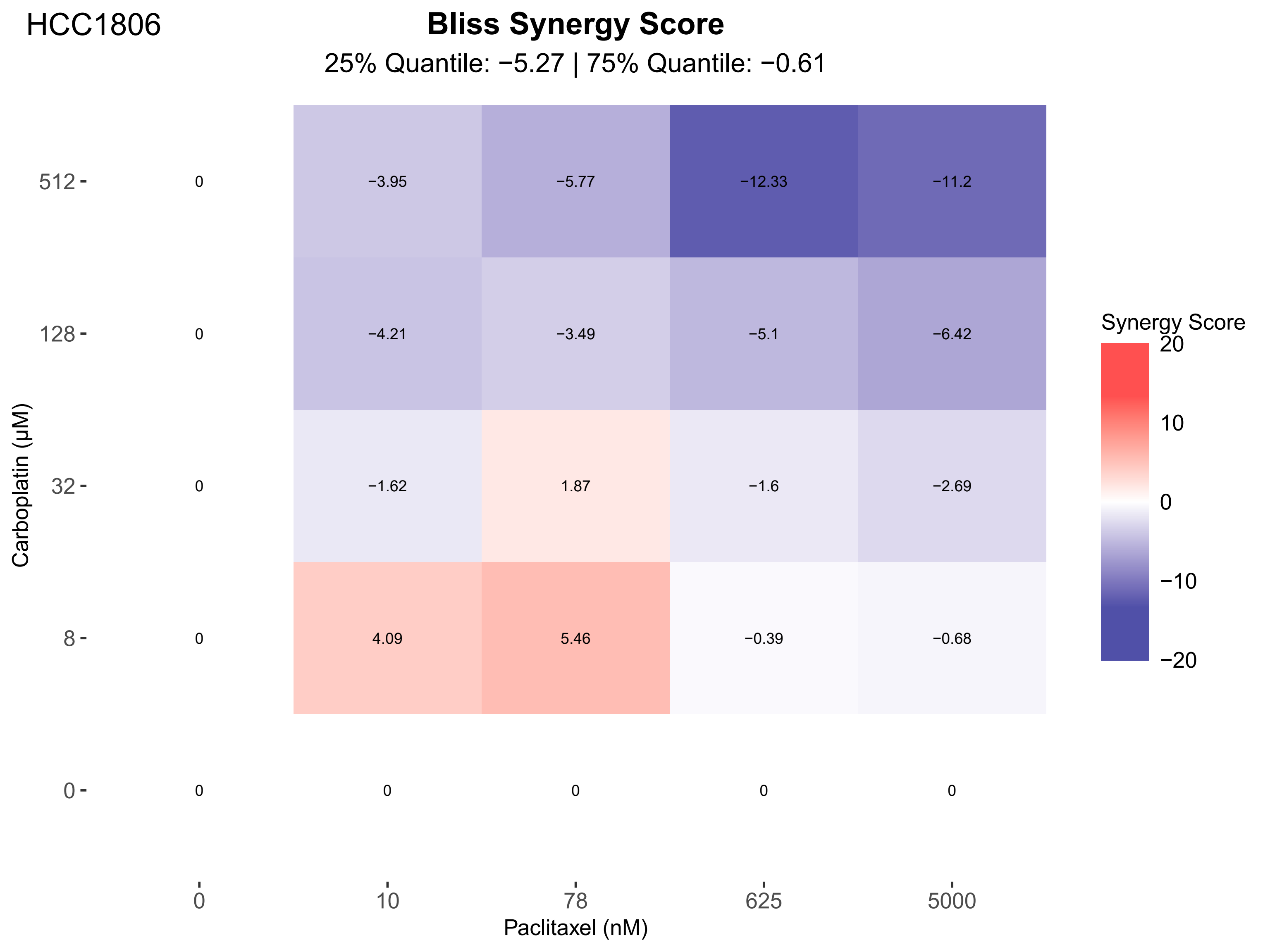

HCC1806
Bliss Synergy Score
25% Quantile: −5.27 | 75% Quantile: −0.61
512
128
32
8
0
0
0
0
0
0
−3.95
−5.77
−12.33
−11.2
−4.21
−3.49
−5.1
−6.42
−1.62
1.87
−1.6
−2.69
4.09
5.46
−0.39
−0.68
0
0
0
0
Carboplatin (µM)
0
10
78
625
5000
Paclitaxel (nM)
Synergy Score
20
10
0
−10
−20

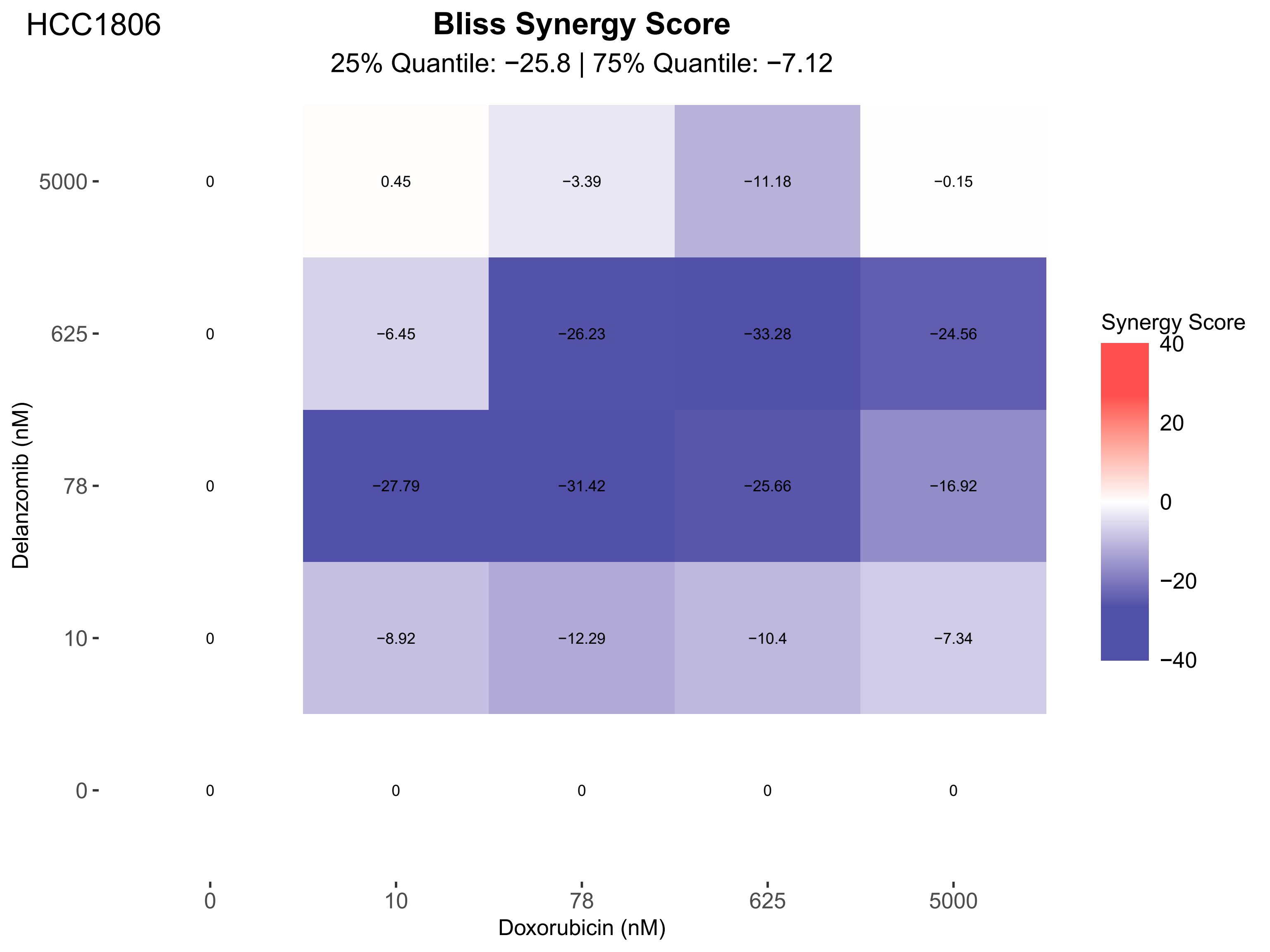
HCC1806
Bliss Synergy Score
25% Quantile: −25.8 | 75% Quantile: −7.12
Delanzomib (nM)
5000
625
78
10
0
Doxorubicin (nM)
0
10
78
625
5000
0
0.45
−3.39
−11.18
−0.15
0
−6.45
−26.23
−33.28
−24.56
0
−27.79
−31.42
−25.66
−16.92
0
−8.92
−12.29
−10.4
−7.34
0
0
0
0
0
Synergy Score
40
20
0
−20
−40

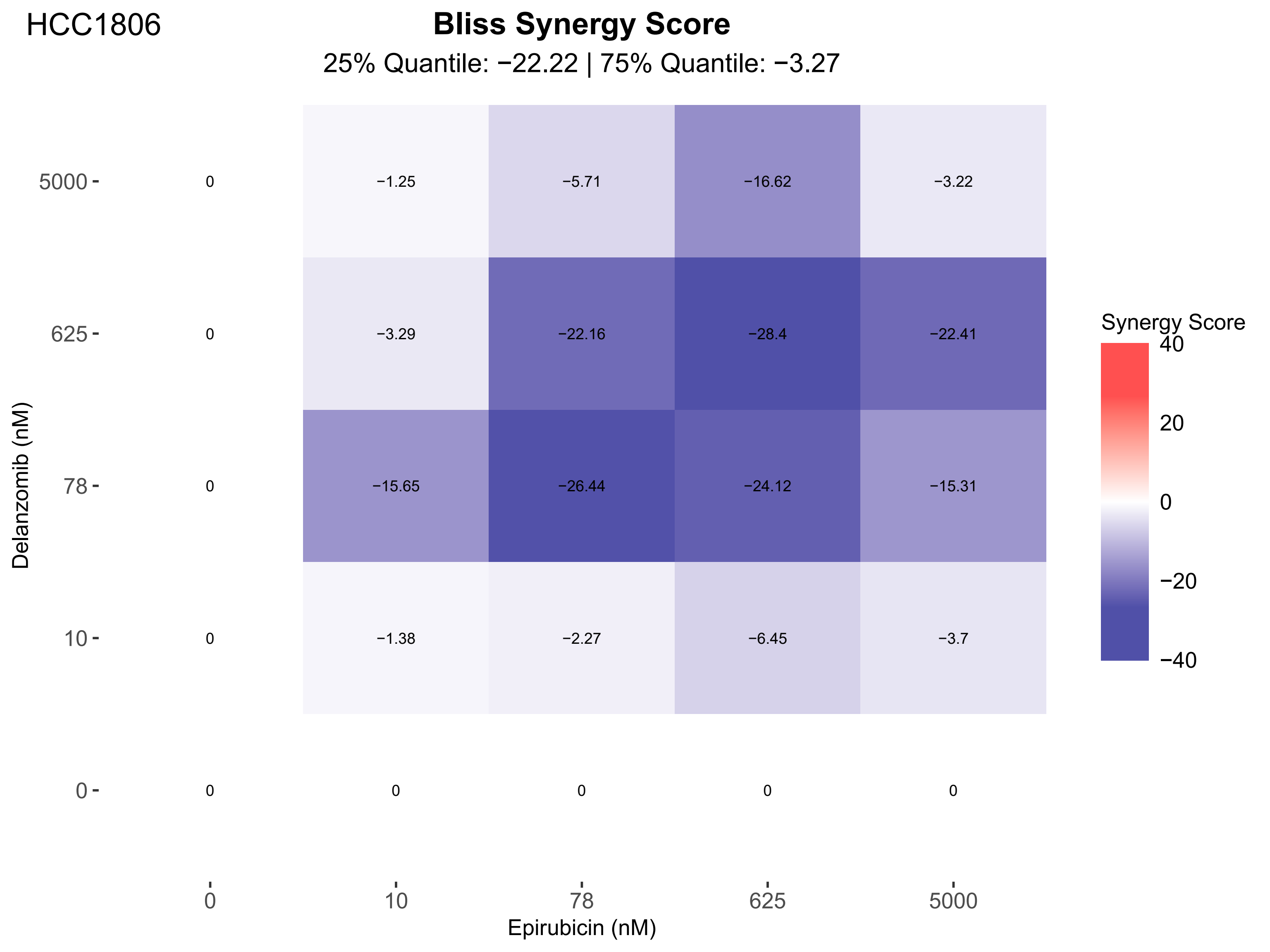
HCC1806
Bliss Synergy Score
25% Quantile: −22.22 | 75% Quantile: −3.27
Delanzomib (nM)
5000
625
78
10
0
0
−1.25
−5.71
−16.62
−3.22
0
−3.29
−22.16
−28.4
−22.41
0
−15.65
−26.44
−24.12
−15.31
0
−1.38
−2.27
−6.45
−3.7
0
0
0
0
0
0
10
78
625
5000
Epirubicin (nM)
Synergy Score
40
20
0
−20
−40

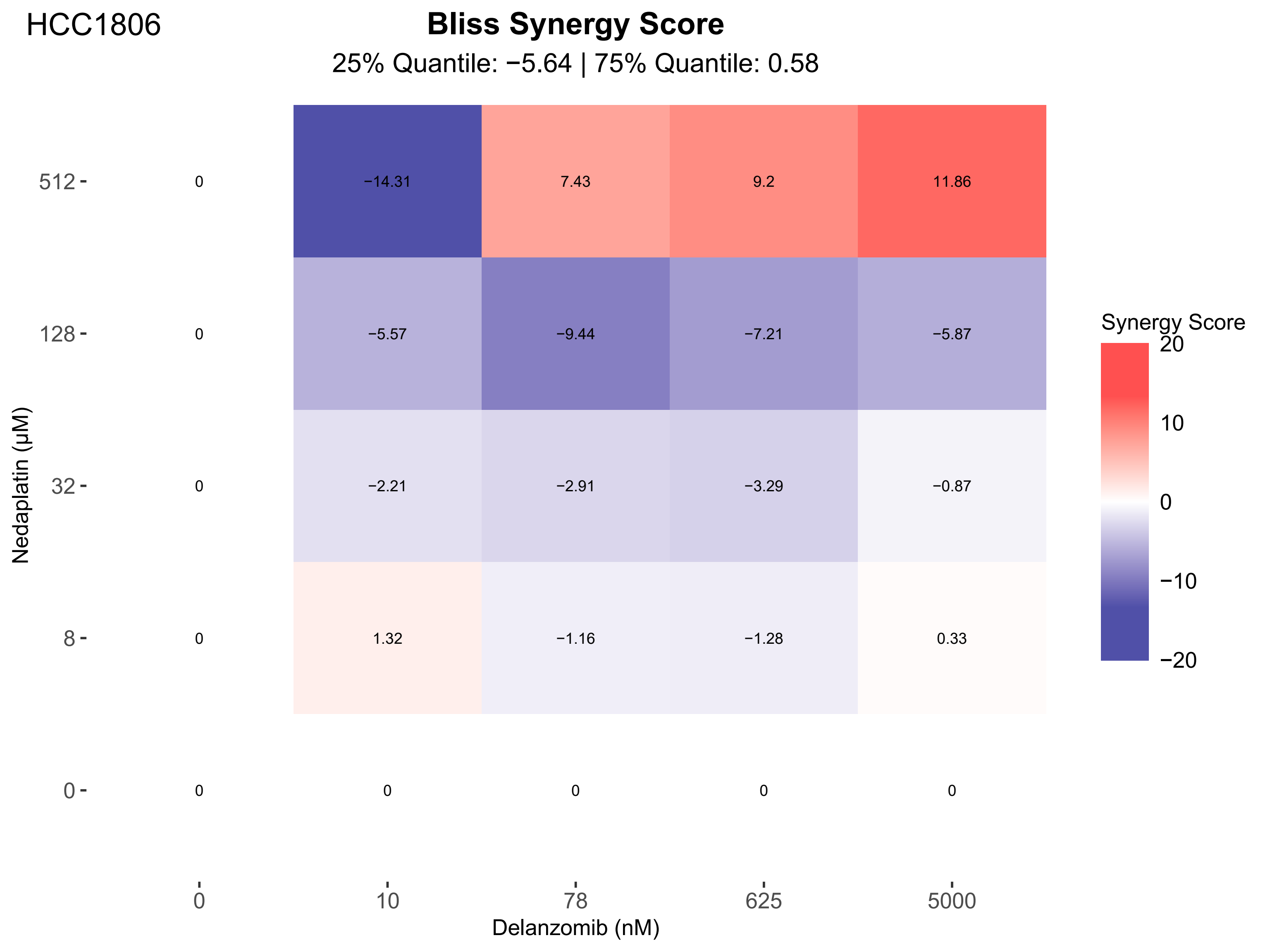
HCC1806
Bliss Synergy Score
25% Quantile: −5.64 | 75% Quantile: 0.58
Nedaplatin (μM)
512
128
32
8
0
Delanzomib (nM)
0
10
78
625
5000
−14.31
7.43
9.2
11.86
−5.57
−9.44
−7.21
−5.87
−2.21
−2.91
−3.29
−0.87
1.32
−1.16
−1.28
0.33
Synergy Score
20
10
0
−10
−20

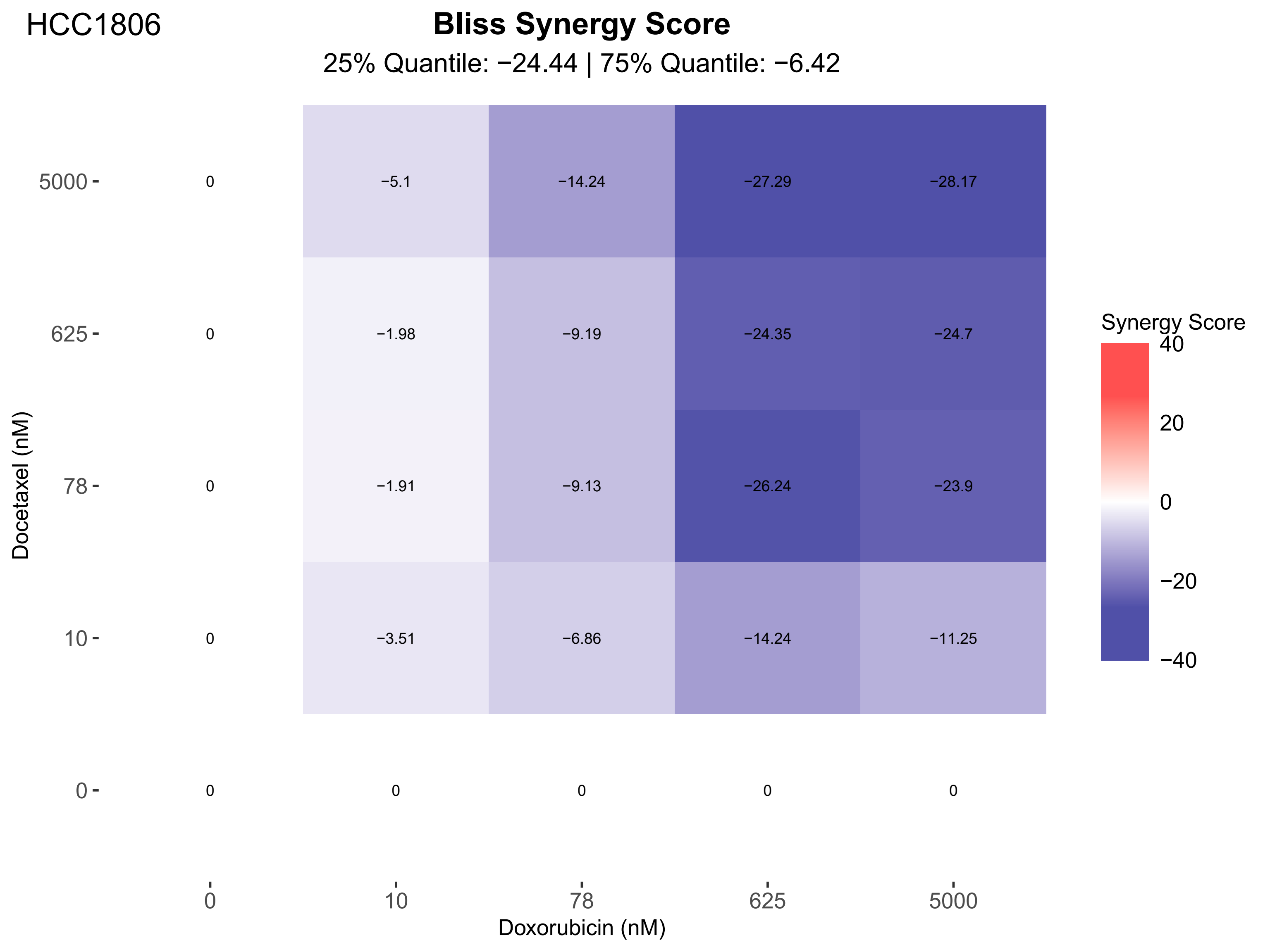
HCC1806
Bliss Synergy Score
25% Quantile: −24.44 | 75% Quantile: −6.42
Docetaxel (nM)
5000
625
78
10
0
Doxorubicin (nM)
0
10
78
625
5000
0
−5.1
−14.24
−27.29
−28.17
0
−1.98
−9.19
−24.35
−24.7
0
−1.91
−9.13
−26.24
−23.9
0
−3.51
−6.86
−14.24
−11.25
0
0
0
0
0
Synergy Score
40
20
0
−20
−40

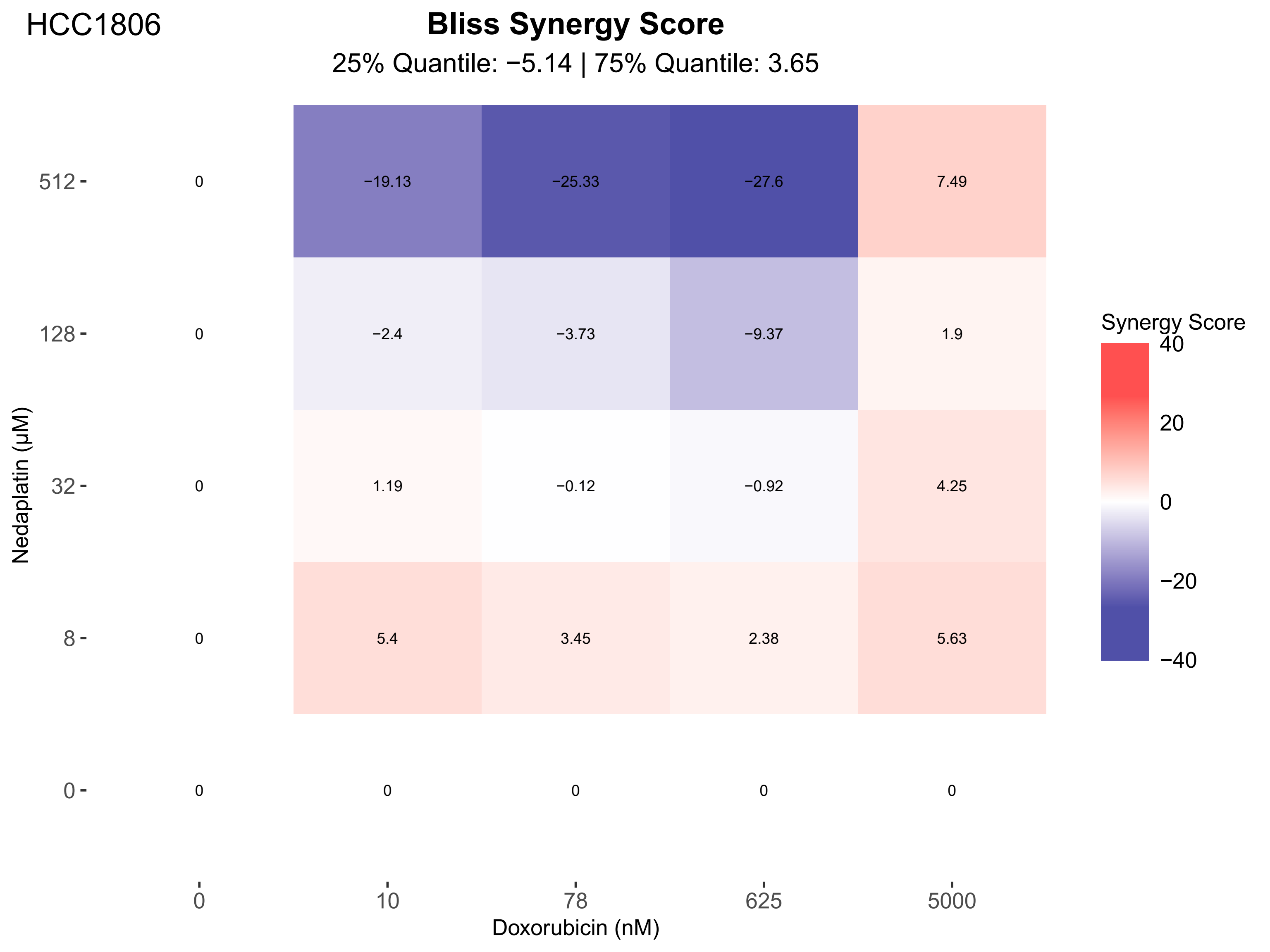

HCC1806
Bliss Synergy Score
25% Quantile: −5.14 | 75% Quantile: 3.65
Nedaplatin (μM)
512
128
32
8
0
Doxorubicin (nM)
0
10
78
625
5000
0
−19.13
−25.33
−27.6
7.49
−2.4
−3.73
−9.37
1.9
1.19
−0.12
−0.92
4.25
5.4
3.45
2.38
5.63
Synergy Score
40
20
−20
−40

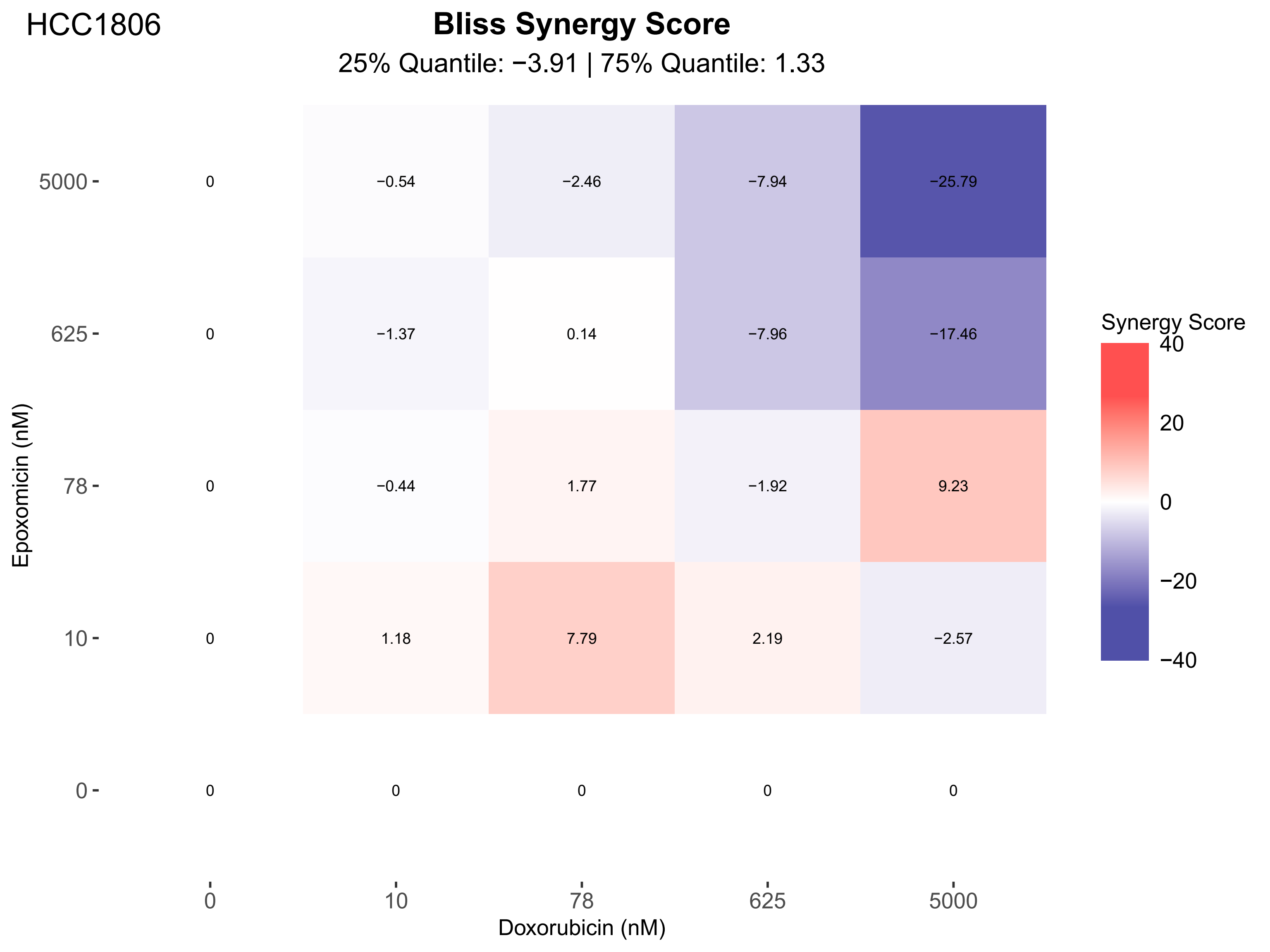
HCC1806
Bliss Synergy Score
25% Quantile: −3.91 | 75% Quantile: 1.33
Epoxomicin (nM)
5000
625
78
10
0
0
−0.54
−2.46
−7.94
−25.79
0
−1.37
0.14
−7.96
−17.46
0
−0.44
1.77
−1.92
9.23
0
1.18
7.79
2.19
−2.57
0
0
0
0
0
0
10
78
625
5000
Doxorubicin (nM)
Synergy Score
40
20
0
−20
−40

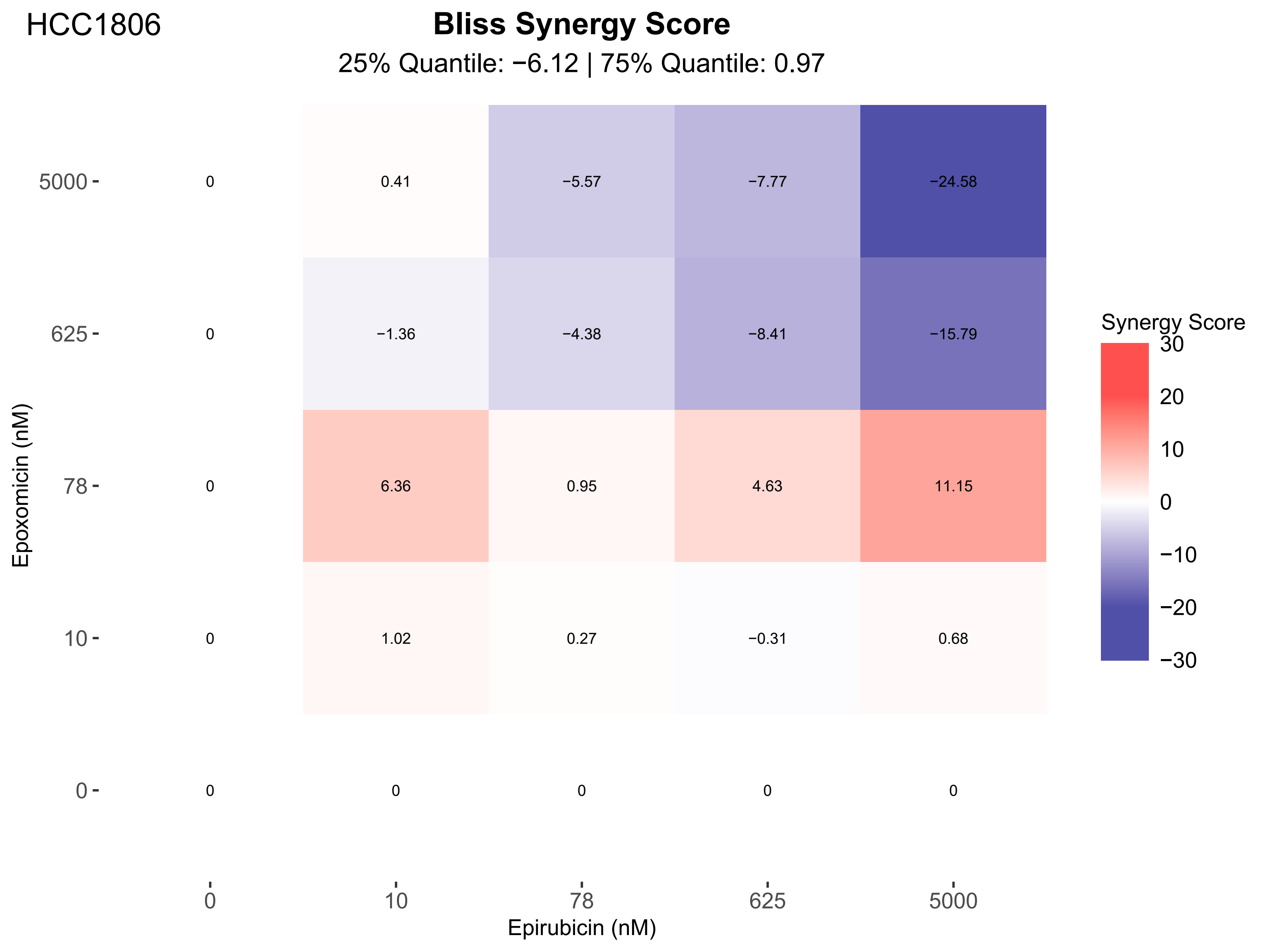

HCC1806
Bliss Synergy Score
25% Quantile: −6.12 | 75% Quantile: 0.97
Epoxomicin (nM)
5000
625
78
10
0
0
0.41
−5.57
−7.77
−24.58
0
−1.36
−4.38
−8.41
−15.79
0
6.36
0.95
4.63
11.15
0
1.02
0.27
−0.31
0.68
0
0
0
0
0
0
10
78
625
5000
Epirubicin (nM)
Synergy Score
30
20
10
0
−10
−20
−30

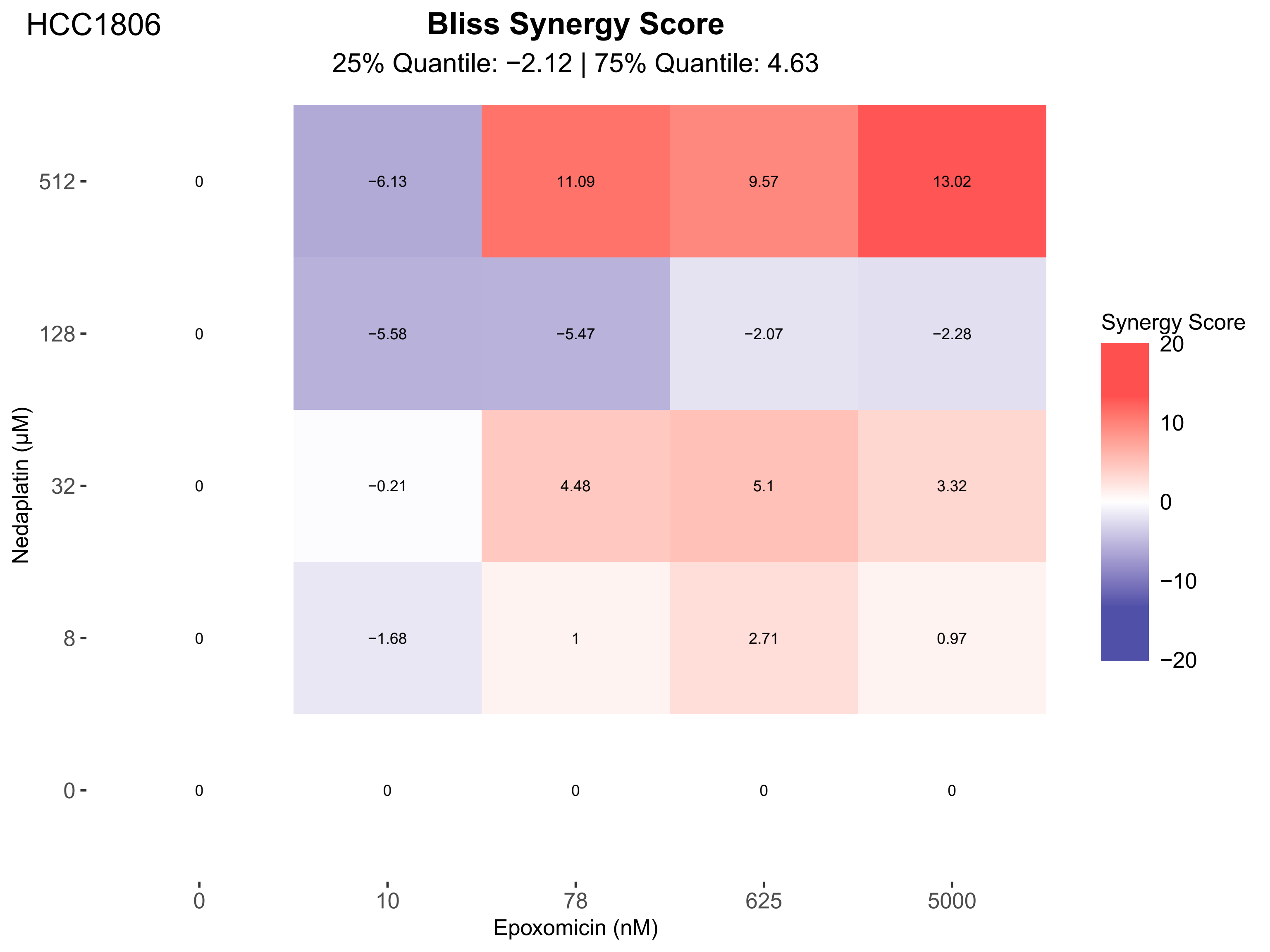
HCC1806
Bliss Synergy Score
25% Quantile: −2.12 | 75% Quantile: 4.63
Nedaplatin (μM)
512
128
32
8
0
Epoxomicin (nM)
0
10
78
625
5000
−6.13
11.09
9.57
13.02
−5.58
−5.47
−2.07
−2.28
−0.21
4.48
5.1
3.32
−1.68
1
2.71
0.97
Synergy Score
20
10
0
−10
−20

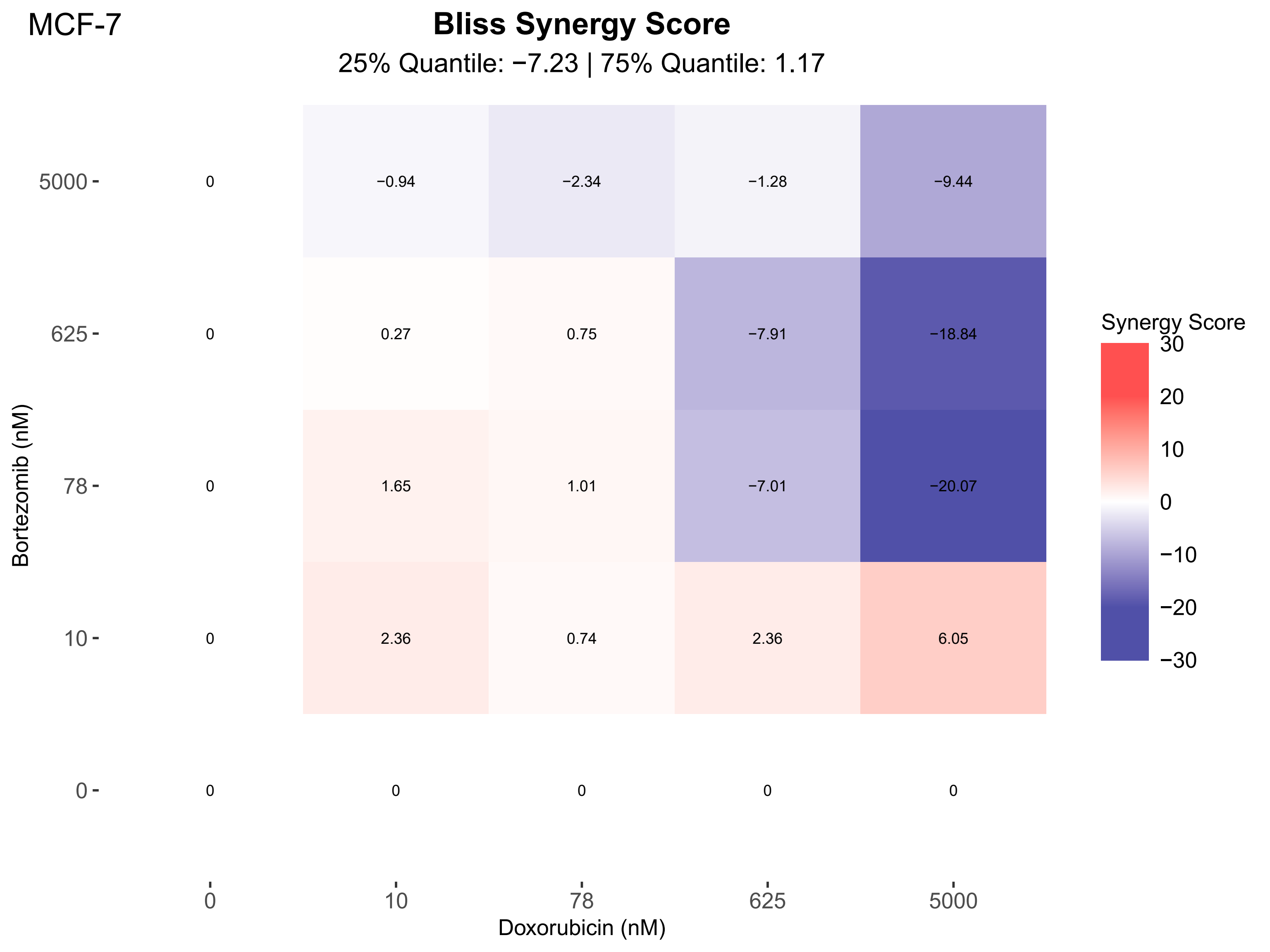
MCF-7
Bliss Synergy Score
25% Quantile: −7.23 | 75% Quantile: 1.17
Bortezomib (nM)
5000
625
78
10
0
Doxorubicin (nM)
0
10
78
625
5000
−0.94
−2.34
−1.28
−9.44
0.27
0.75
−7.91
−18.84
1.65
1.01
−7.01
−20.07
2.36
0.74
2.36
6.05
Synergy Score
30
20
10
0
−10
−20
−30

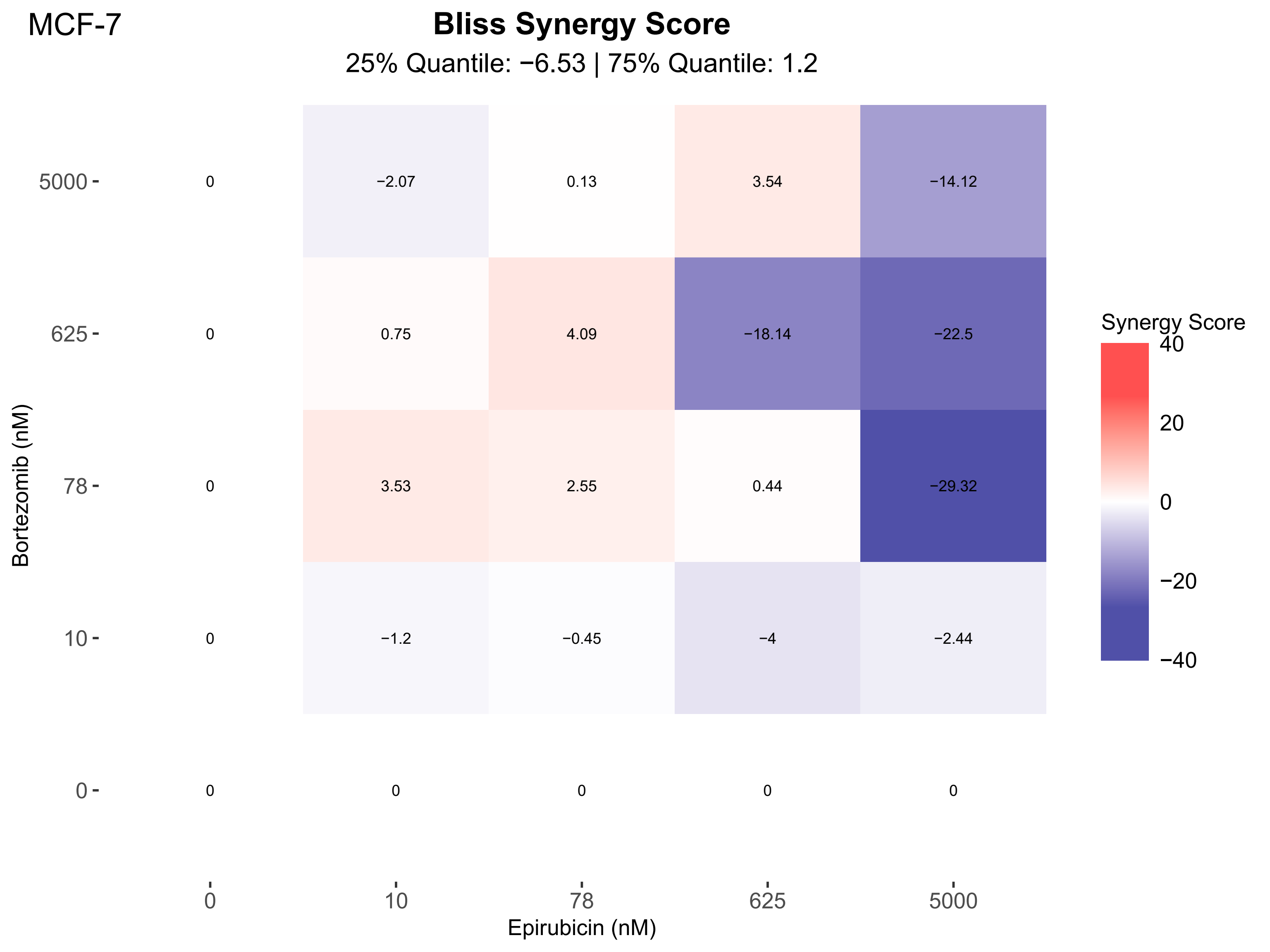
MCF-7
Bliss Synergy Score
25% Quantile: −6.53 | 75% Quantile: 1.2
Bortezomib (nM)
5000
625
78
10
0
Epirubicin (nM)
0
10
78
625
5000
0
−2.07
0.13
3.54
−14.12
0
0.75
4.09
−18.14
−22.5
0
3.53
2.55
0.44
−29.32
0
−1.2
−0.45
−4
−2.44
0
0
0
0
0
Synergy Score
40
20
0
−20
−40

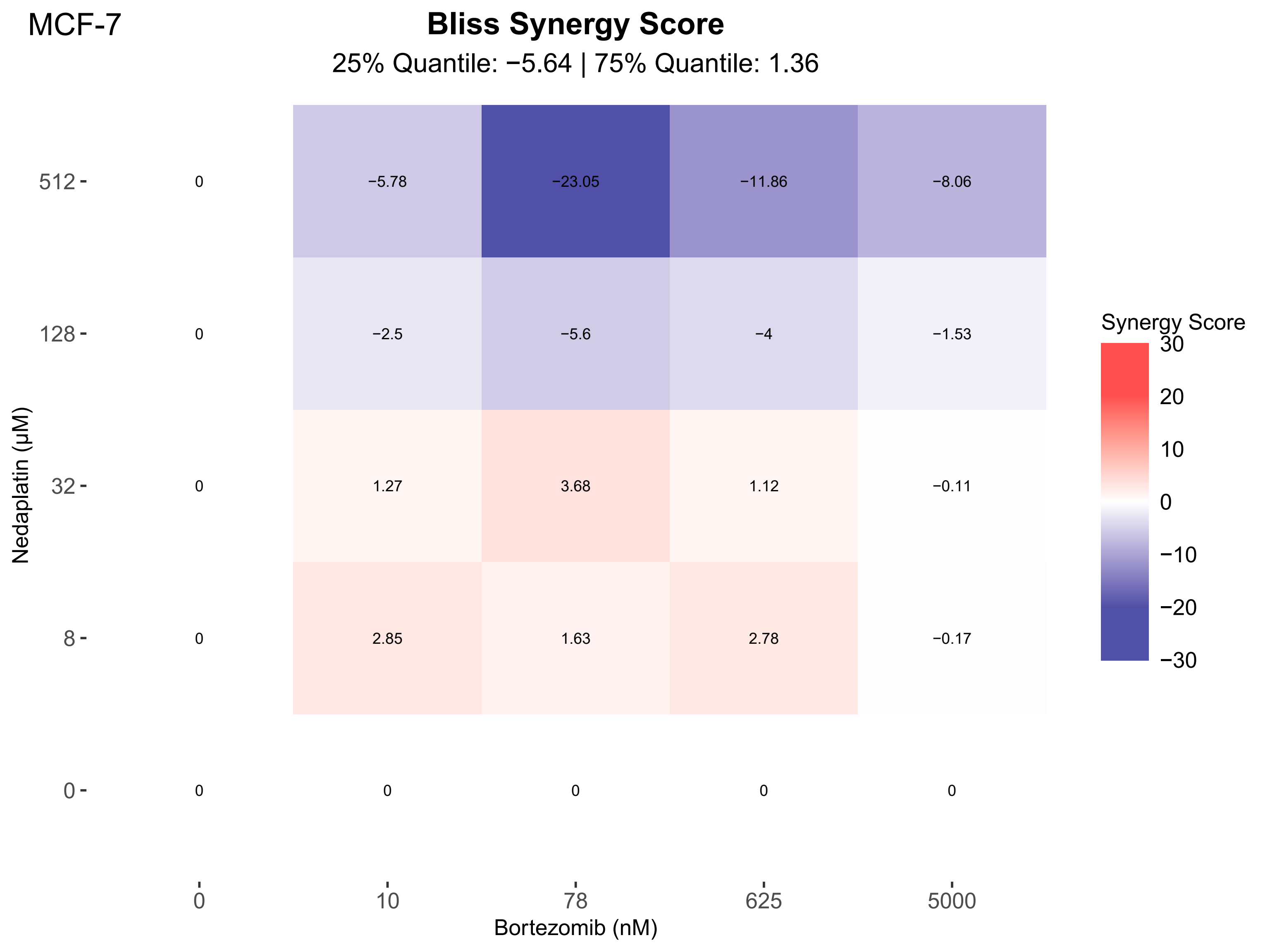
MCF-7
Bliss Synergy Score
25% Quantile: −5.64 | 75% Quantile: 1.36
Nedaplatin (μM)
512
128
32
8
0
Bortezomib (nM)
0
10
78
625
5000
0
−5.78
−23.05
−11.86
−8.06
0
−2.5
−5.6
−4
−1.53
0
1.27
3.68
1.12
−0.11
0
2.85
1.63
2.78
−0.17
0
0
0
0
0
Synergy Score
30
20
10
0
−10
−20
−30

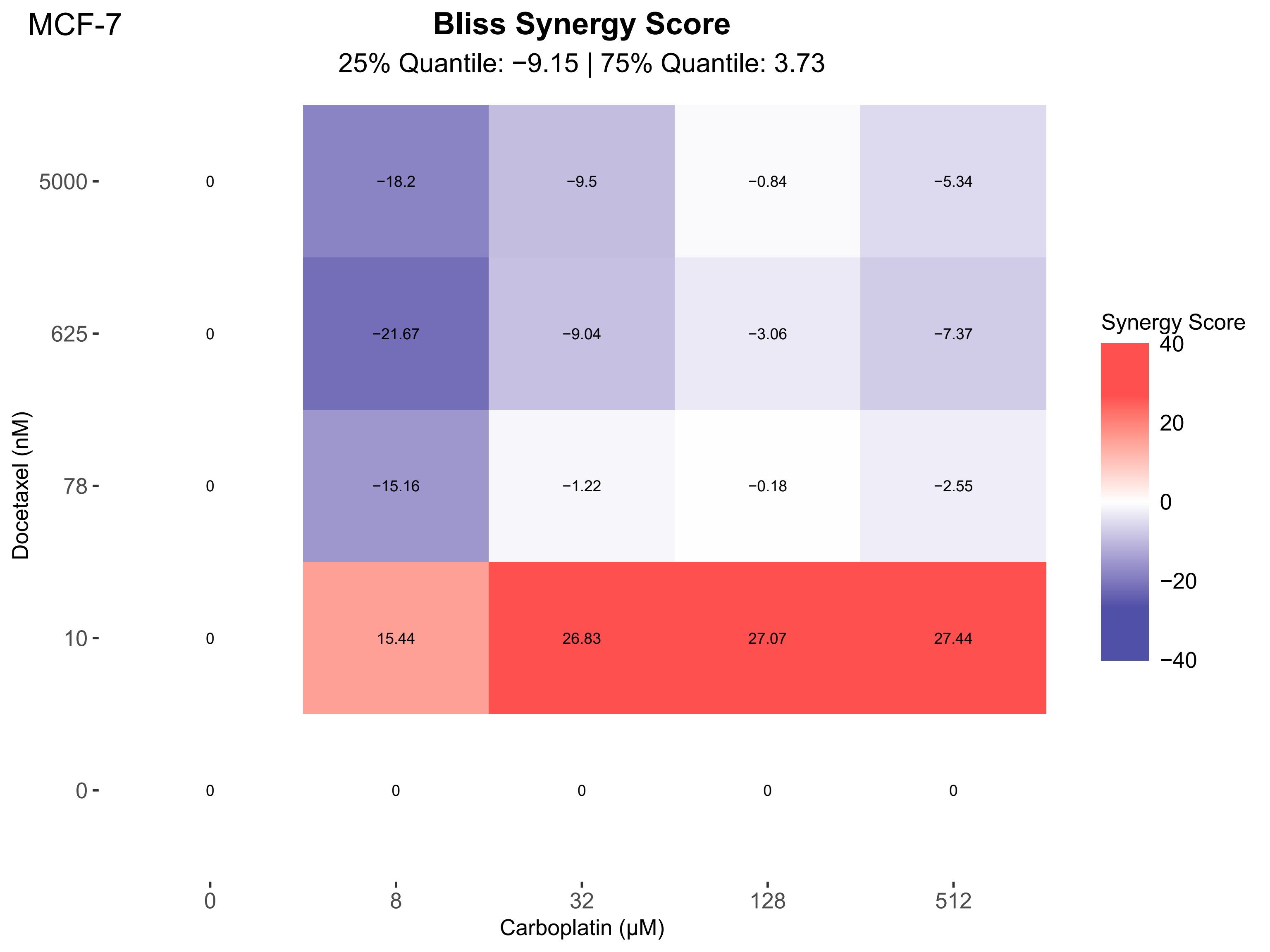
MCF-7
Bliss Synergy Score
25% Quantile: −9.15 | 75% Quantile: 3.73
Docetaxel (nM)
5000
625
78
10
0
Carboplatin (μM)
0
8
32
128
512
0
−18.2
−9.5
−0.84
−5.34
0
−21.67
−9.04
−3.06
−7.37
0
−15.16
−1.22
−0.18
−2.55
0
15.44
26.83
27.07
27.44
0
0
0
0
0
Synergy Score
40
20
0
−20
−40

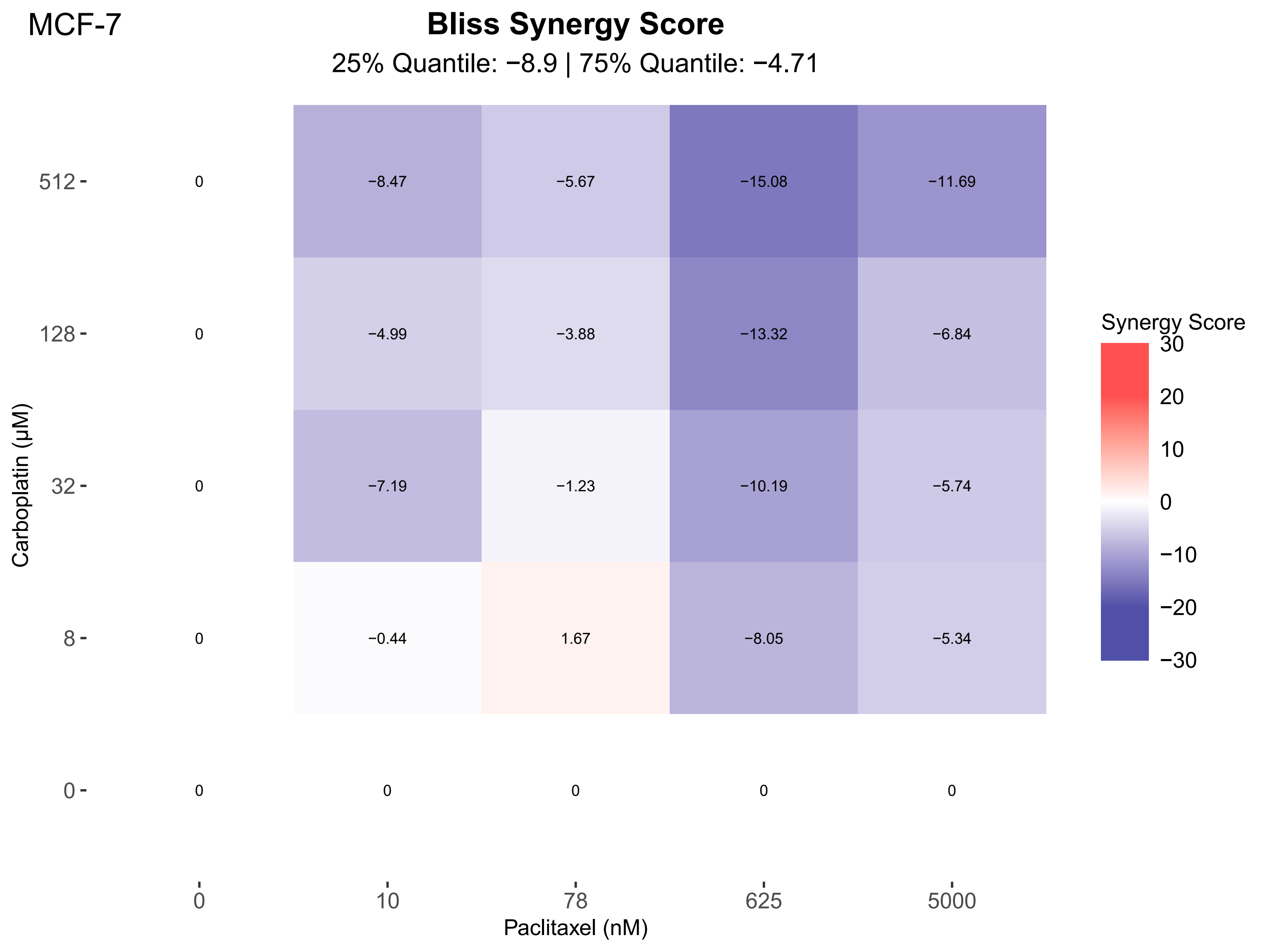
MCF-7
Bliss Synergy Score
25% Quantile: −8.9 | 75% Quantile: −4.71
Carboplatin (μM)
512
128
32
8
0
Paclitaxel (nM)
0
10
78
625
5000
0 −8.47 −5.67 −15.08 −11.69
0 −4.99 −3.88 −13.32 −6.84
0 −7.19 −1.23 −10.19 −5.74
0 −0.44 1.67 −8.05 −5.34
0 0 0 0 0
Synergy Score
30
20
10
0
−10
−20
−30

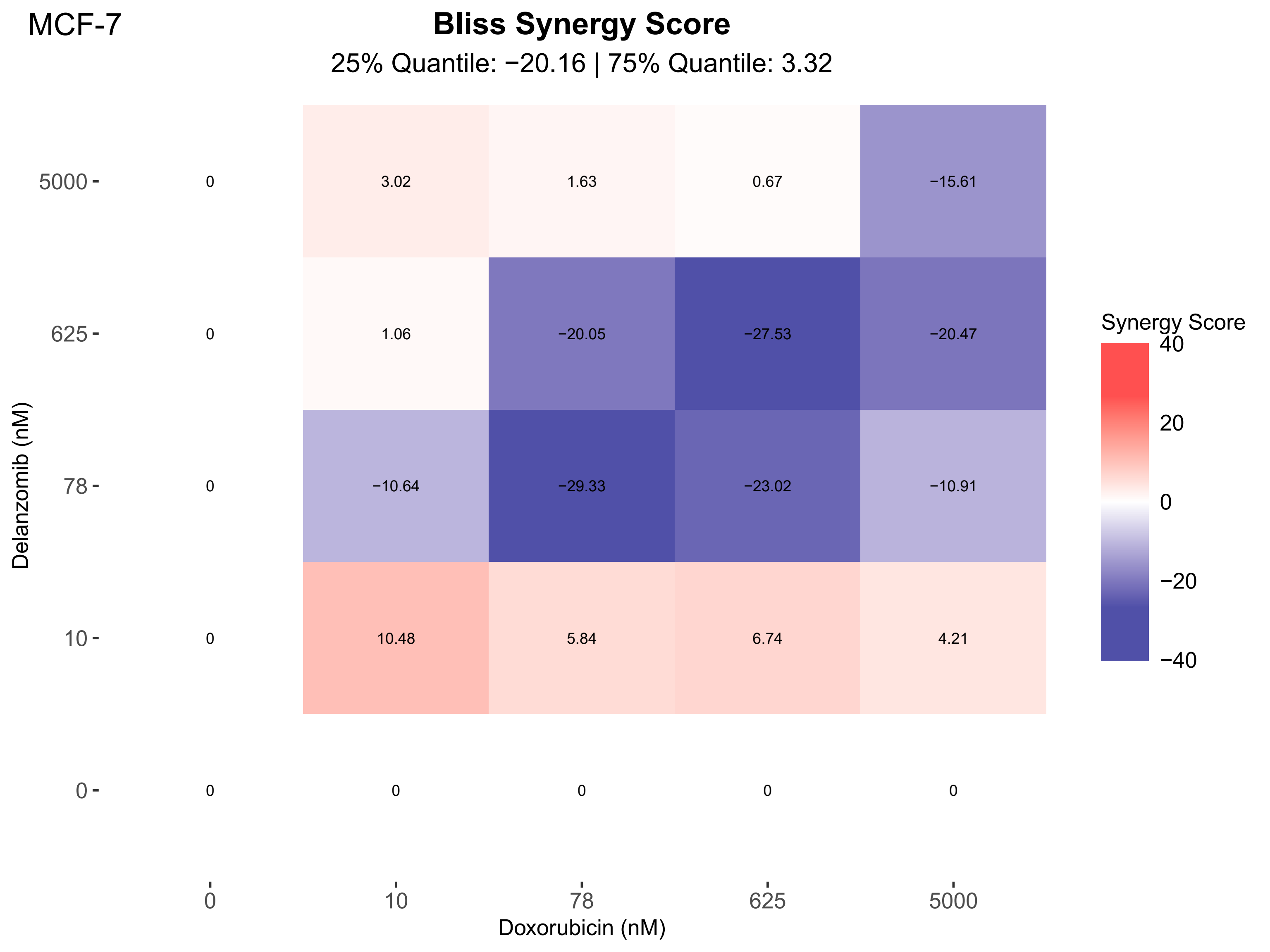
MCF-7
Bliss Synergy Score
25% Quantile: −20.16 | 75% Quantile: 3.32
Delanzomib (nM)
5000
625
78
10
0
Doxorubicin (nM)
0
10
78
625
5000
0
3.02
1.63
0.67
−15.61
0
1.06
−20.05
−27.53
−20.47
0
−10.64
−29.33
−23.02
−10.91
0
10.48
5.84
6.74
4.21
0
0
0
0
0
Synergy Score
40
20
0
−20
−40

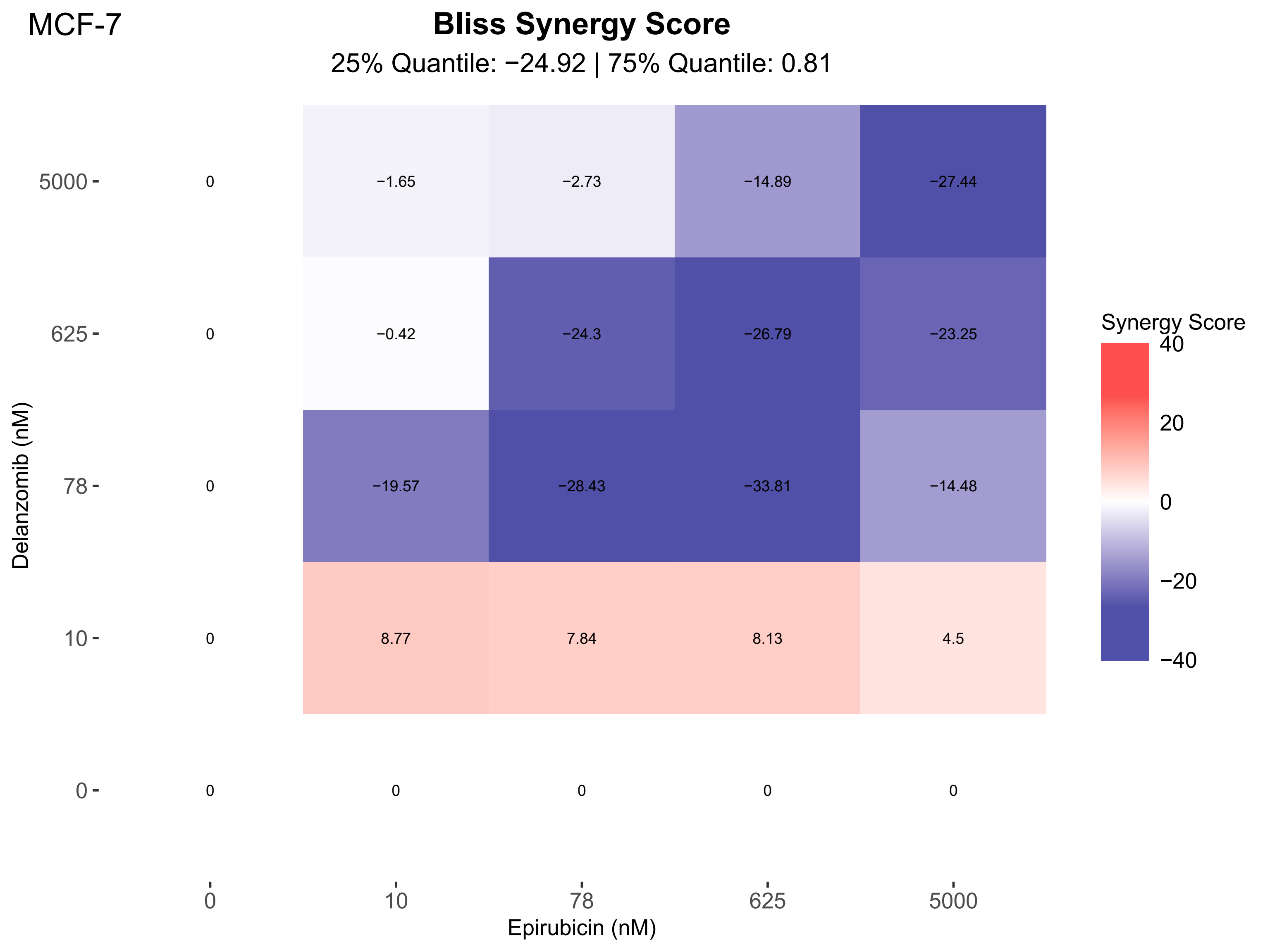
MCF-7
Bliss Synergy Score
25% Quantile: −24.92 | 75% Quantile: 0.81
Delanzomib (nM)
5000
625
78
10
0
Epirubicin (nM)
0
10
78
625
5000
0
−1.65
−2.73
−14.89
−27.44
0
−0.42
−24.3
−26.79
−23.25
0
−19.57
−28.43
−33.81
−14.48
0
8.77
7.84
8.13
4.5
0
0
0
0
0
Synergy Score
40
20
0
−20
−40

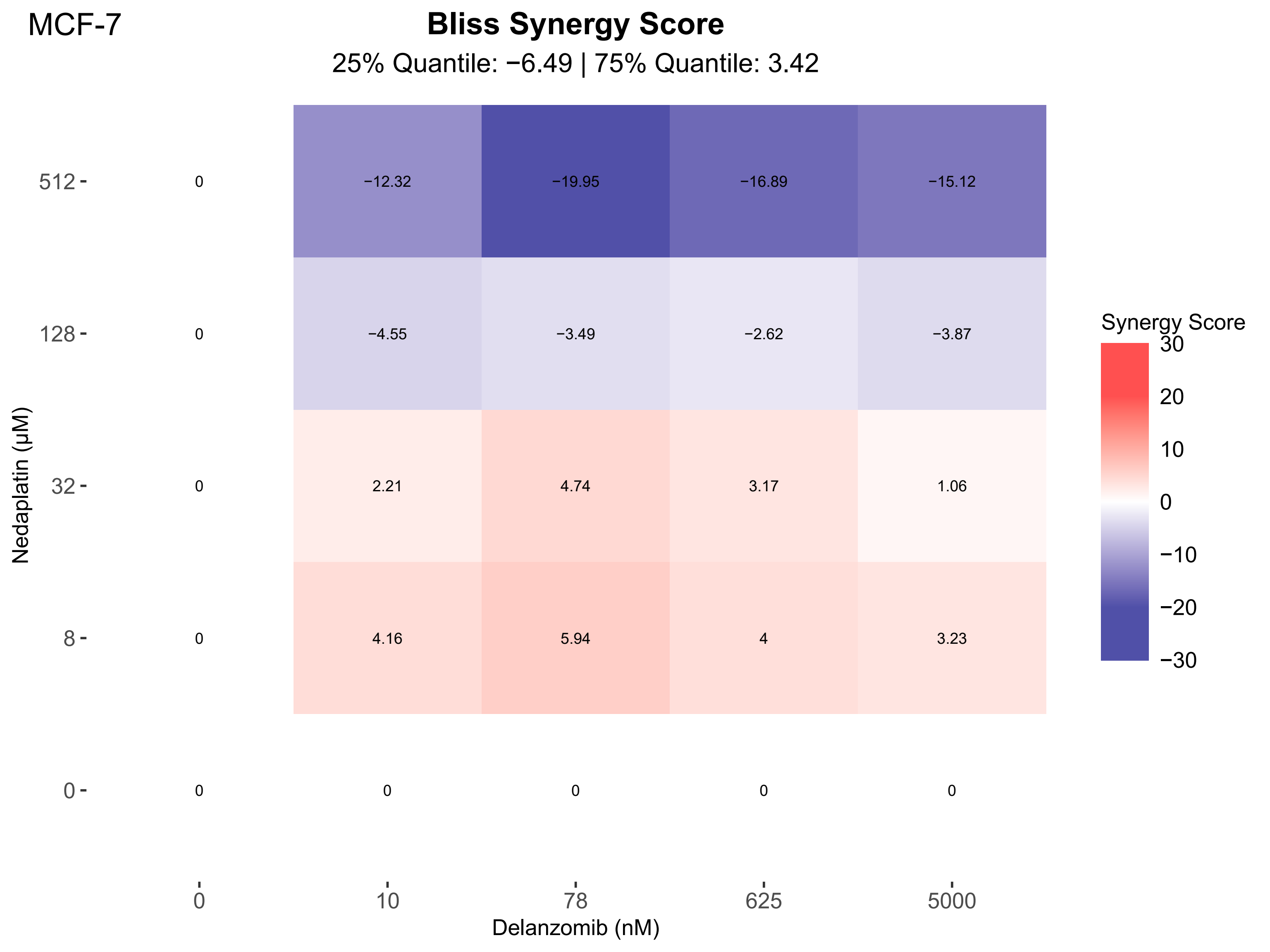
MCF-7
Bliss Synergy Score
25% Quantile: −6.49 | 75% Quantile: 3.42
Nedaplatin (μM)
512
128
32
8
0
Delanzomib (nM)
0
10
78
625
5000
−12.32
−19.95
−16.89
−15.12
−4.55
−3.49
−2.62
−3.87
2.21
4.74
3.17
1.06
4.16
5.94
4
3.23
Synergy Score
30
20
10
0
−10
−20
−30

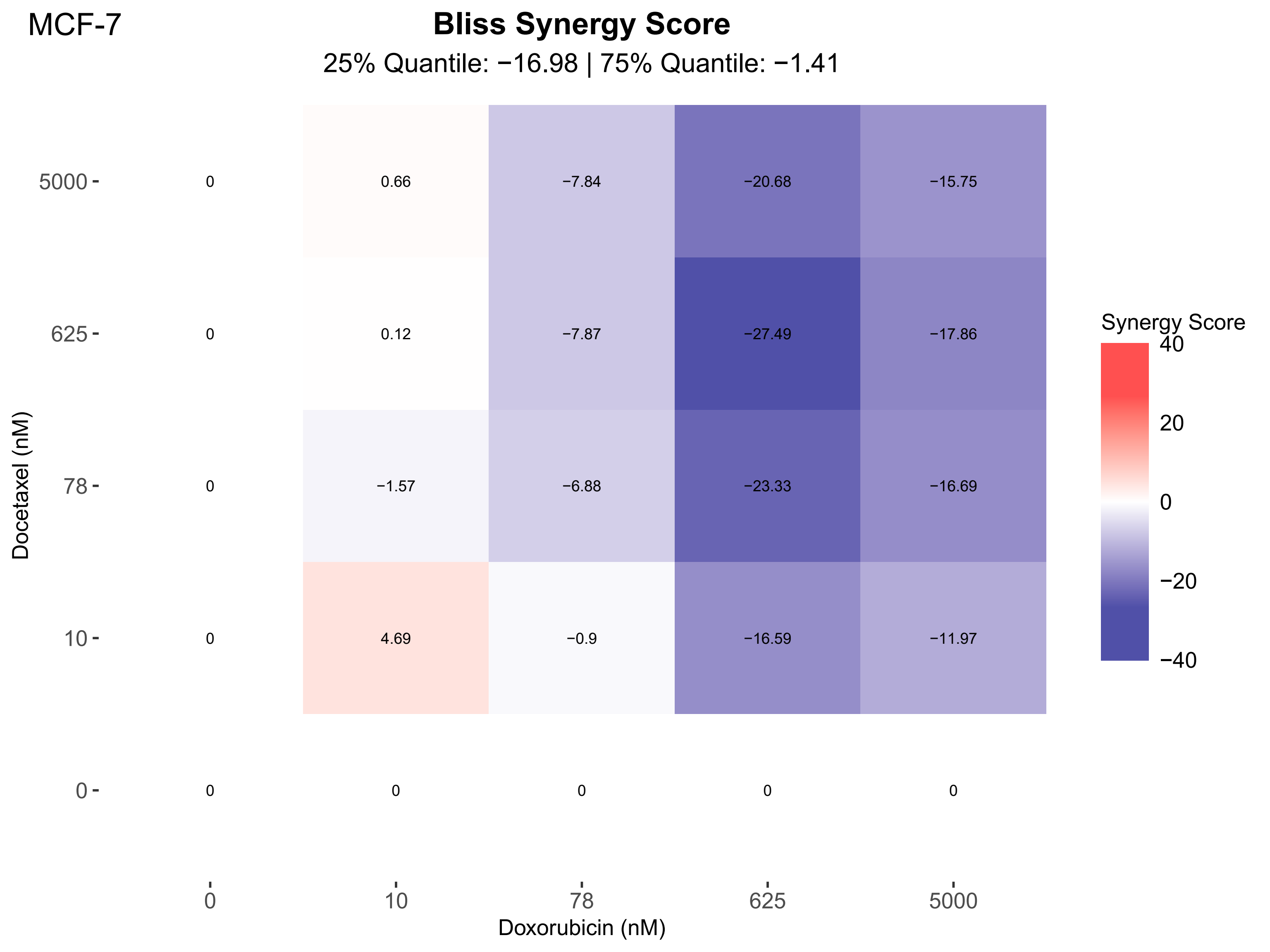
MCF-7
Bliss Synergy Score
25% Quantile: −16.98 | 75% Quantile: −1.41
Docetaxel (nM)
5000
625
78
10
0
Doxorubicin (nM)
0
10
78
625
5000
0
0.66
−7.84
−20.68
−15.75
0
0.12
−7.87
−27.49
−17.86
0
−1.57
−6.88
−23.33
−16.69
0
4.69
−0.9
−16.59
−11.97
0
0
0
0
0
Synergy Score
40
20
0
−20
−40

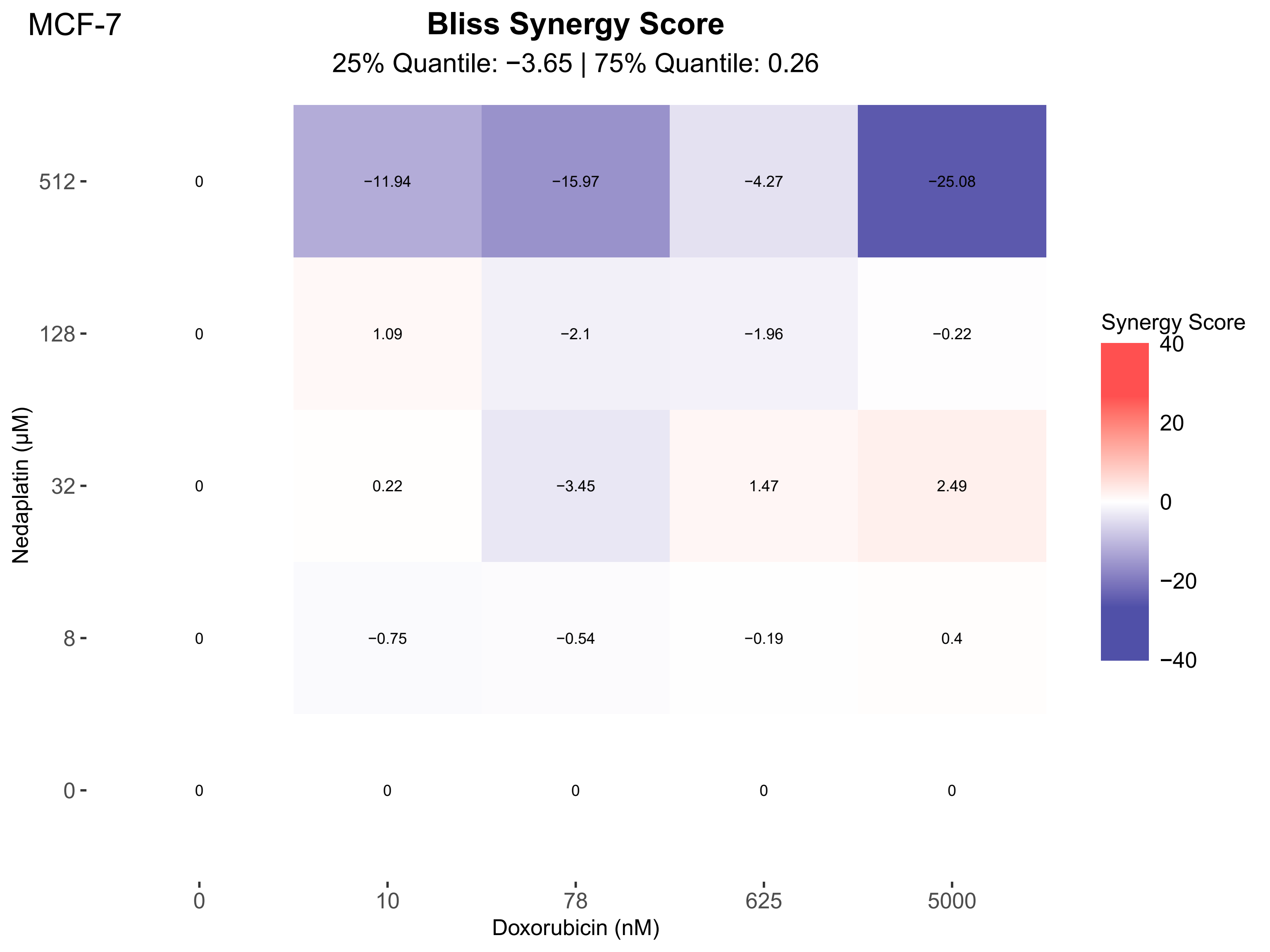
MCF-7
Bliss Synergy Score
25% Quantile: −3.65 | 75% Quantile: 0.26
Nedaplatin (μM)
512
128
32
8
0
Doxorubicin (nM)
0
10
78
625
5000
0
−11.94
−15.97
−4.27
−25.08
0
1.09
−2.1
−1.96
−0.22
0
0.22
−3.45
1.47
2.49
0
−0.75
−0.54
−0.19
0.4
0
0
0
0
0
Synergy Score
40
20
0
−20
−40

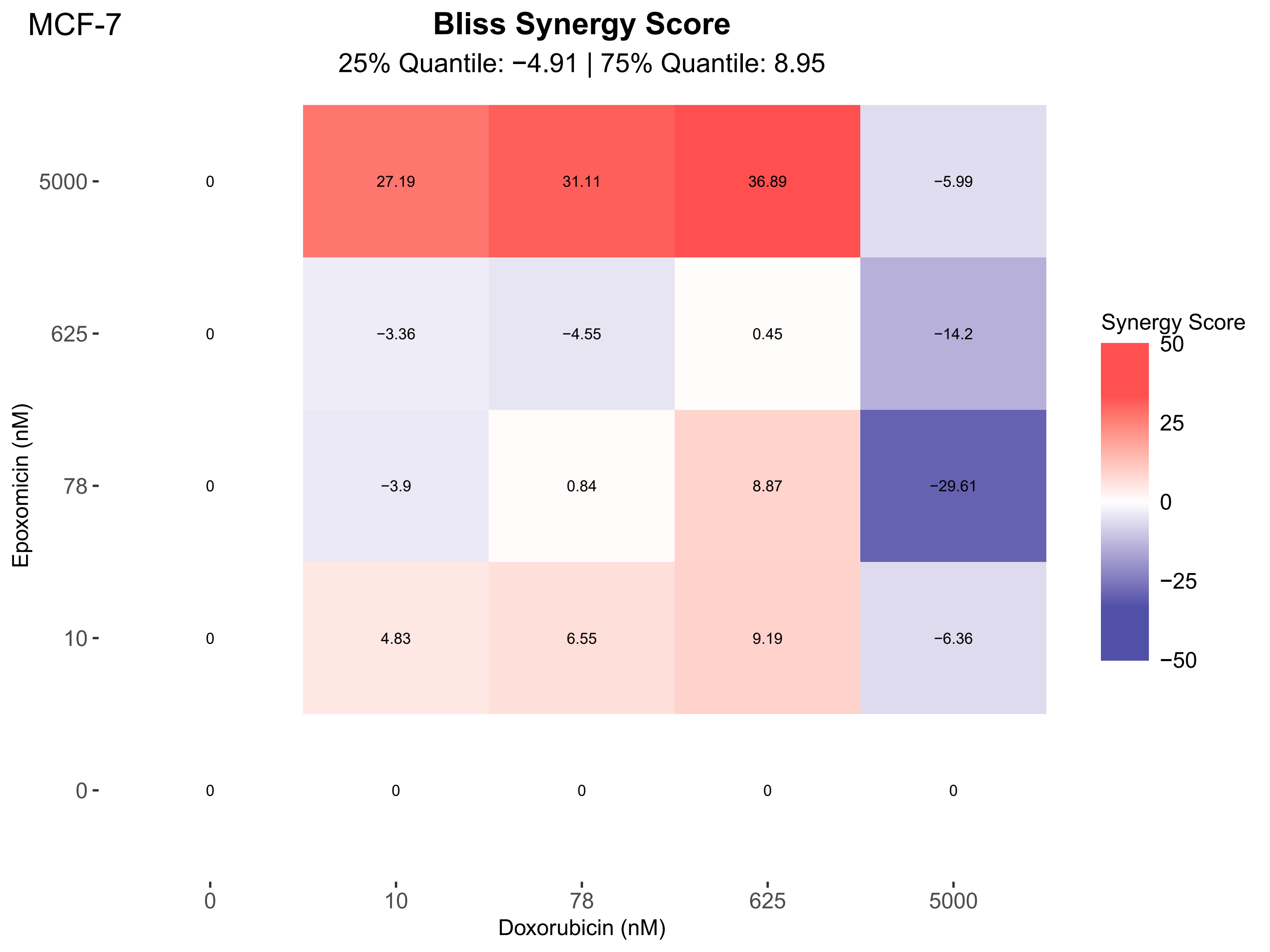

MCF-7
Bliss Synergy Score
25% Quantile: −4.91 | 75% Quantile: 8.95
Synergy Score
50
25
0
−25
−50
Epoxomicin (nM)
5000
625
78
10
0
Doxorubicin (nM)
0
10
78
625
5000
27.19
31.11
36.89
−5.99
−3.36
−4.55
0.45
−14.2
−3.9
0.84
8.87
−29.61
4.83
6.55
9.19
−6.36

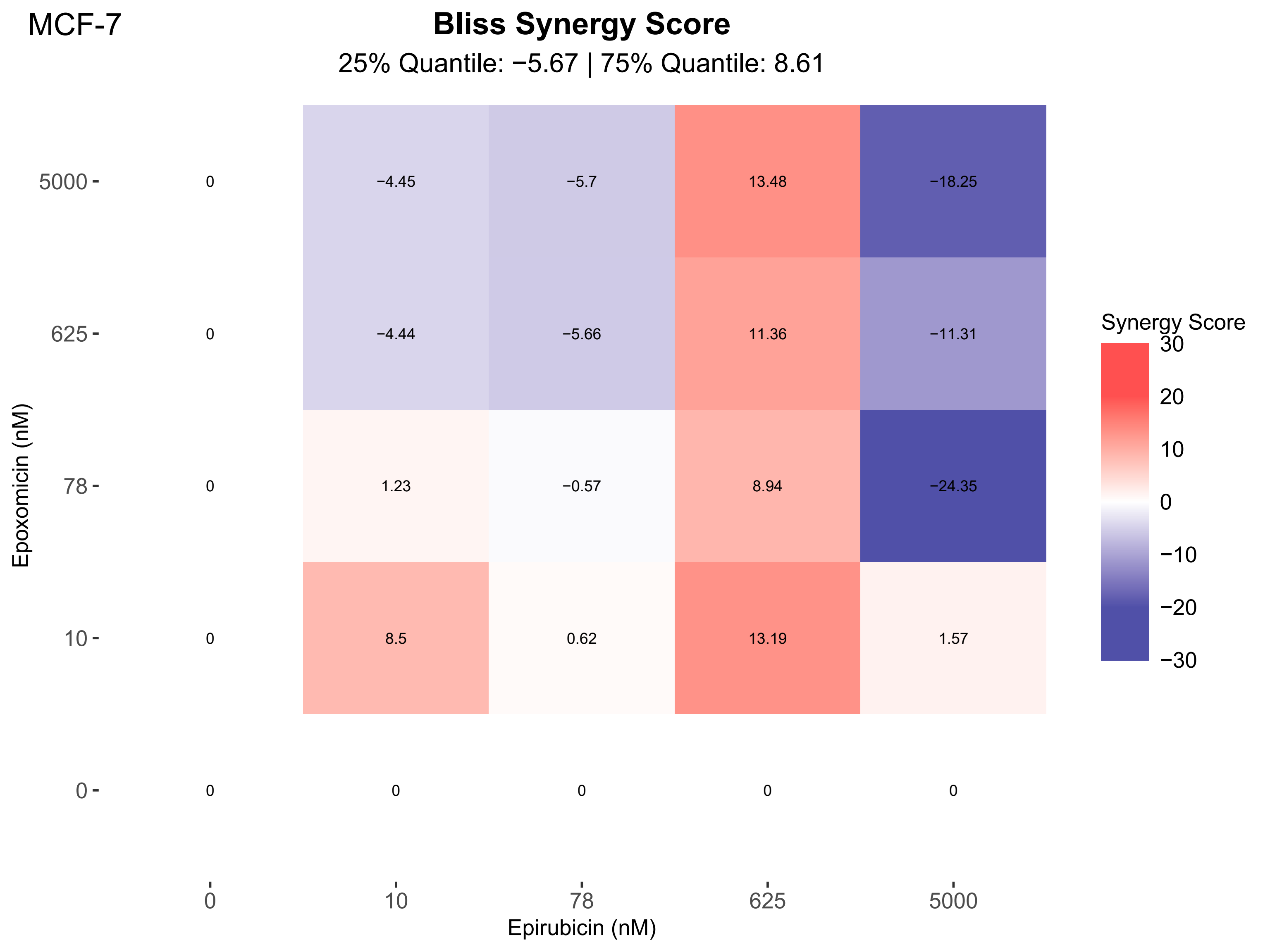
MCF-7
Bliss Synergy Score
25% Quantile: −5.67 | 75% Quantile: 8.61
Synergy Score
30
20
10
0
−10
−20
−30
Epoxomicin (nM)
5000
625
78
10
0
Epirubicin (nM)
0
10
78
625
5000
0
−4.45
−5.7
13.48
−18.25
0
−4.44
−5.66
11.36
−11.31
0
1.23
−0.57
8.94
−24.35
0
8.5
0.62
13.19
1.57
0
0
0
0
0

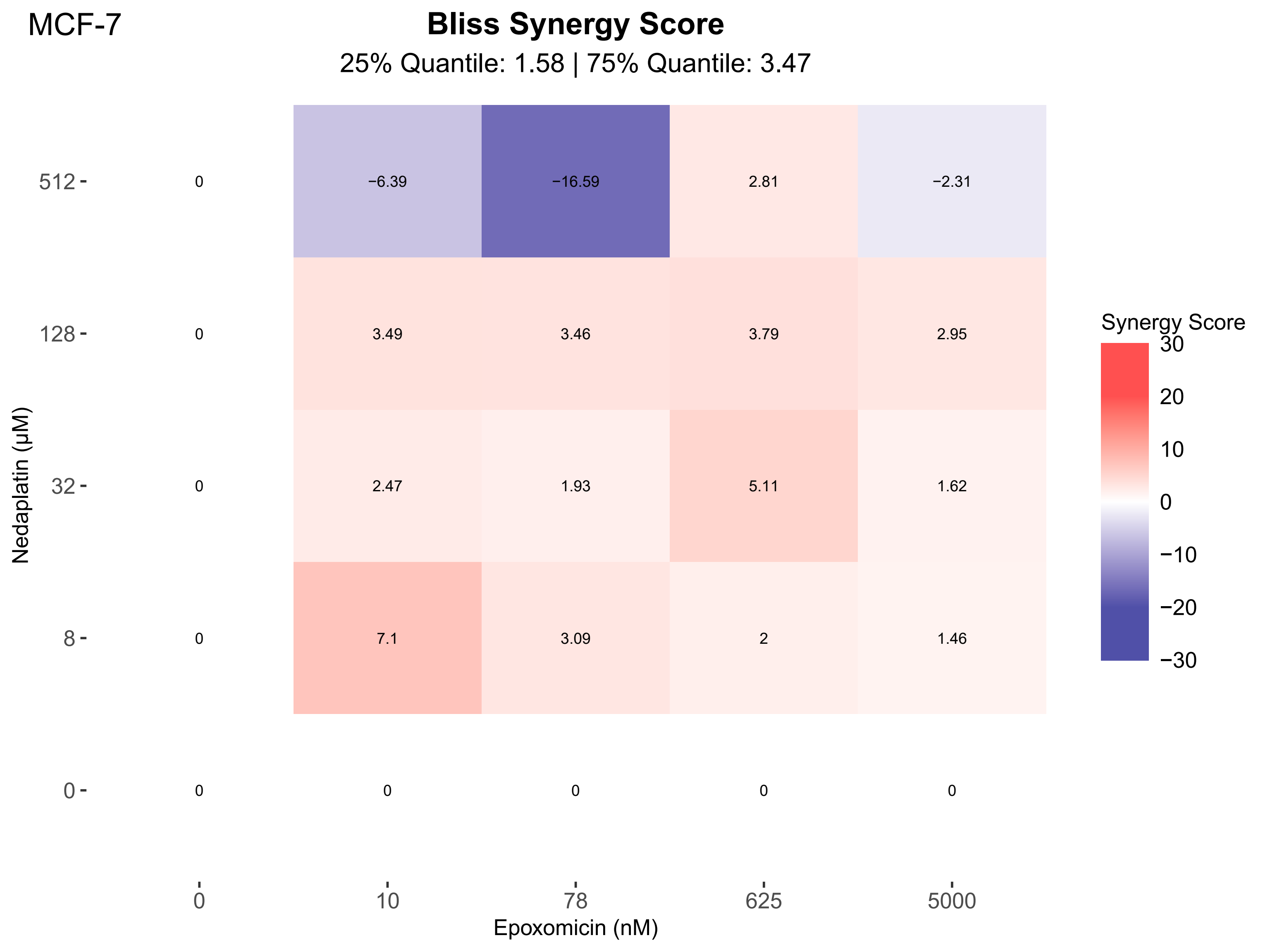
MCF-7
Bliss Synergy Score
25% Quantile: 1.58 | 75% Quantile: 3.47
Nedaplatin (μM)
512
128
32
8
0
Epoxomicin (nM)
0
10
78
625
5000
0 −6.39 −16.59 2.81 −2.31
0 3.49 3.46 3.79 2.95
0 2.47 1.93 5.11 1.62
0 7.1 3.09 2 1.46
0 0 0 0 0
Synergy Score
30
20
10
0
−10
−20
−30

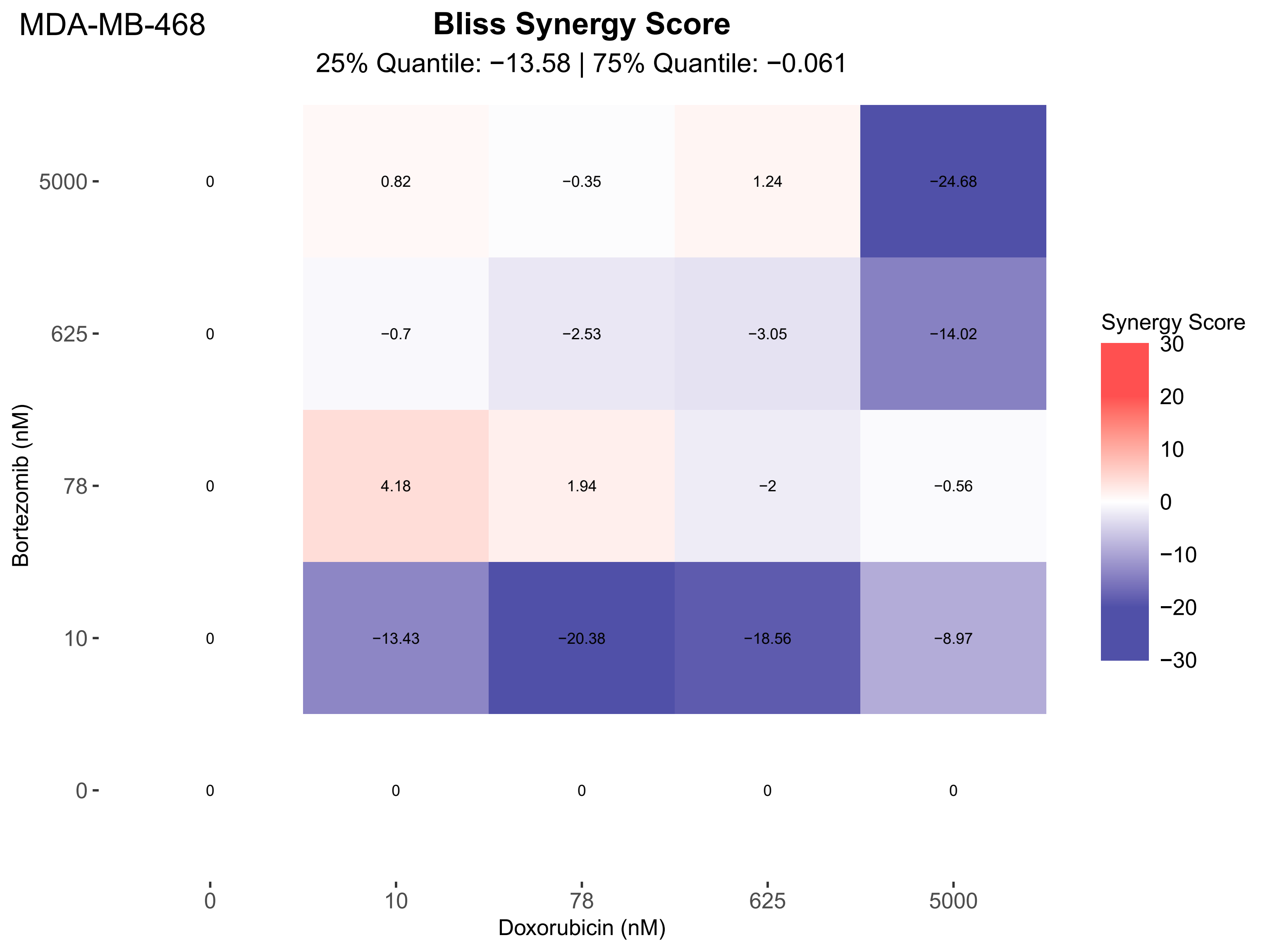
MDA-MB-468
Bliss Synergy Score
25% Quantile: −13.58 | 75% Quantile: −0.061
Bortezomib (nM)
5000
625
78
10
0
Doxorubicin (nM)
0
10
78
625
5000
0
0.82
−0.35
1.24
−24.68
0
−0.7
−2.53
−3.05
−14.02
0
4.18
1.94
−2
−0.56
0
−13.43
−20.38
−18.56
−8.97
0
0
0
0
0
Synergy Score
30
20
10
0
−10
−20
−30

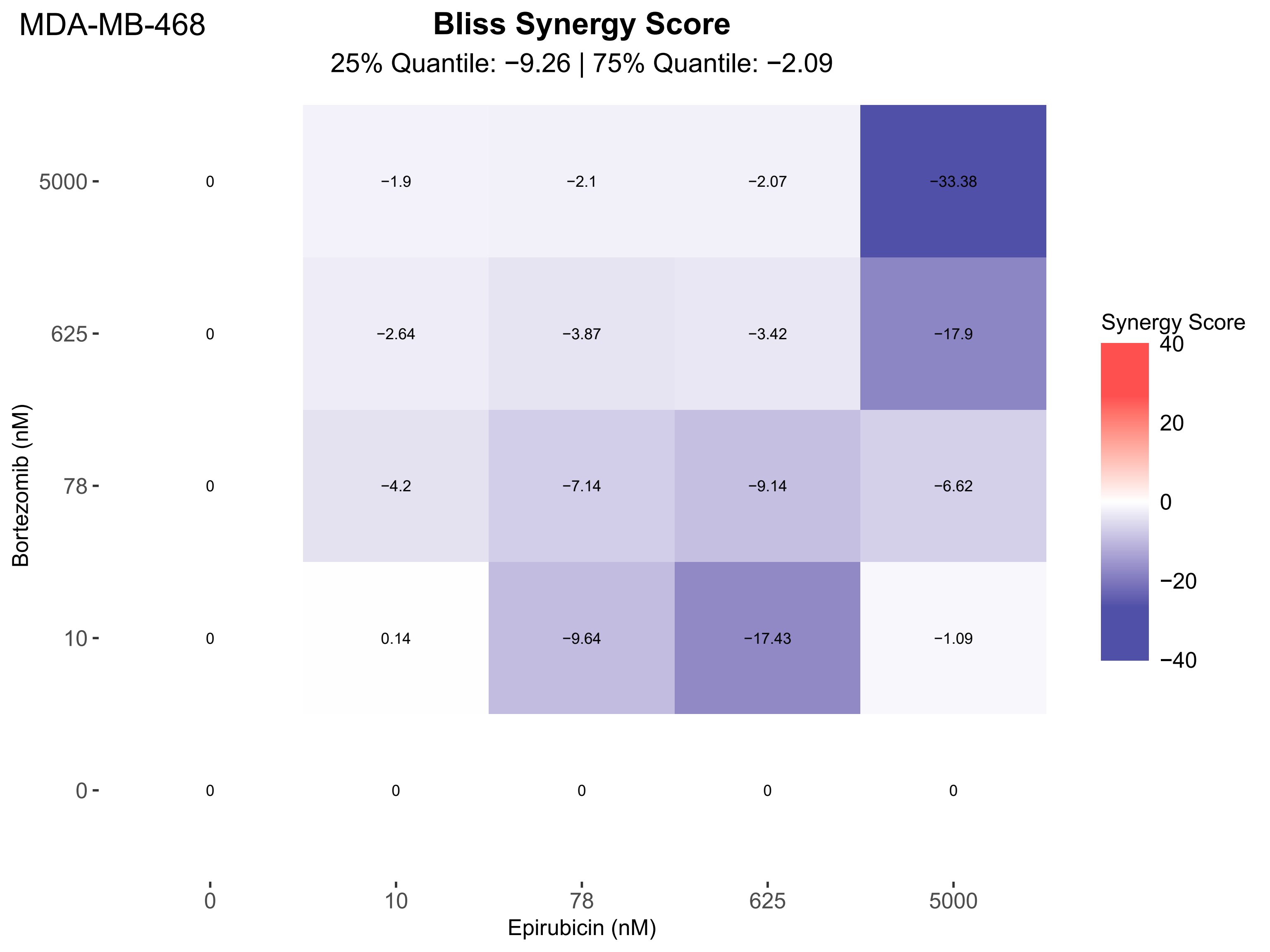

MDA-MB-468
Bliss Synergy Score
25% Quantile: −9.26 | 75% Quantile: −2.09
Bortezomib (nM)
5000
625
78
10
0
0
−1.9
−2.1
−2.07
−33.38
0
−2.64
−3.87
−3.42
−17.9
0
−4.2
−7.14
−9.14
−6.62
0
0.14
−9.64
−17.43
−1.09
0
0
0
0
0
0
10
78
625
5000
Epirubicin (nM)
Synergy Score
40
20
0
−20
−40

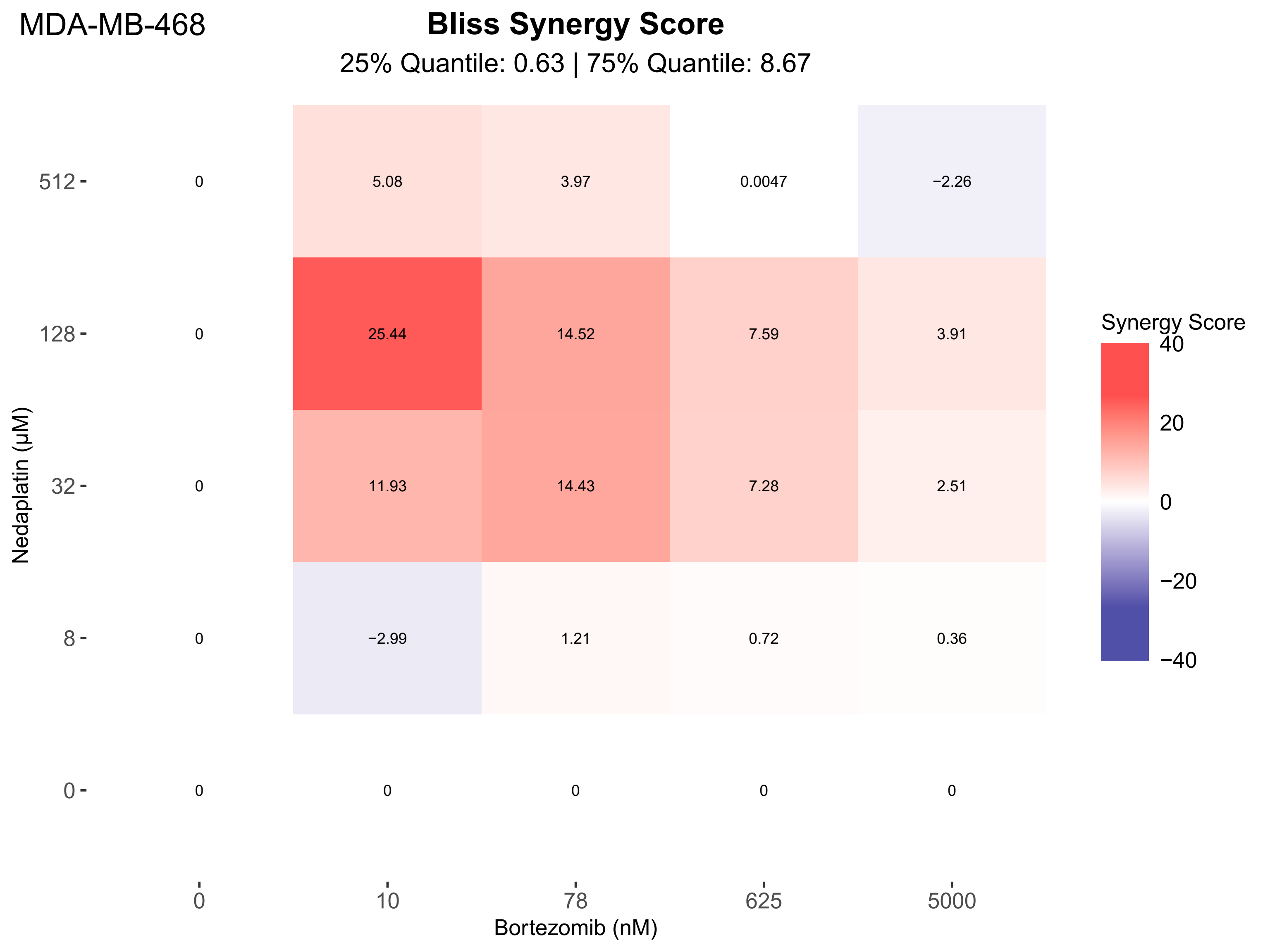
MDA-MB-468
Bliss Synergy Score
25% Quantile: 0.63 | 75% Quantile: 8.67
Nedaplatin (μM)
512
128
32
8
0
0
5.08
3.97
0.0047
−2.26
0
25.44
14.52
7.59
3.91
0
11.93
14.43
7.28
2.51
0
−2.99
1.21
0.72
0.36
0
0
0
0
0
0
10
78
625
5000
Bortezomib (nM)
Synergy Score
40
20
0
−20
−40

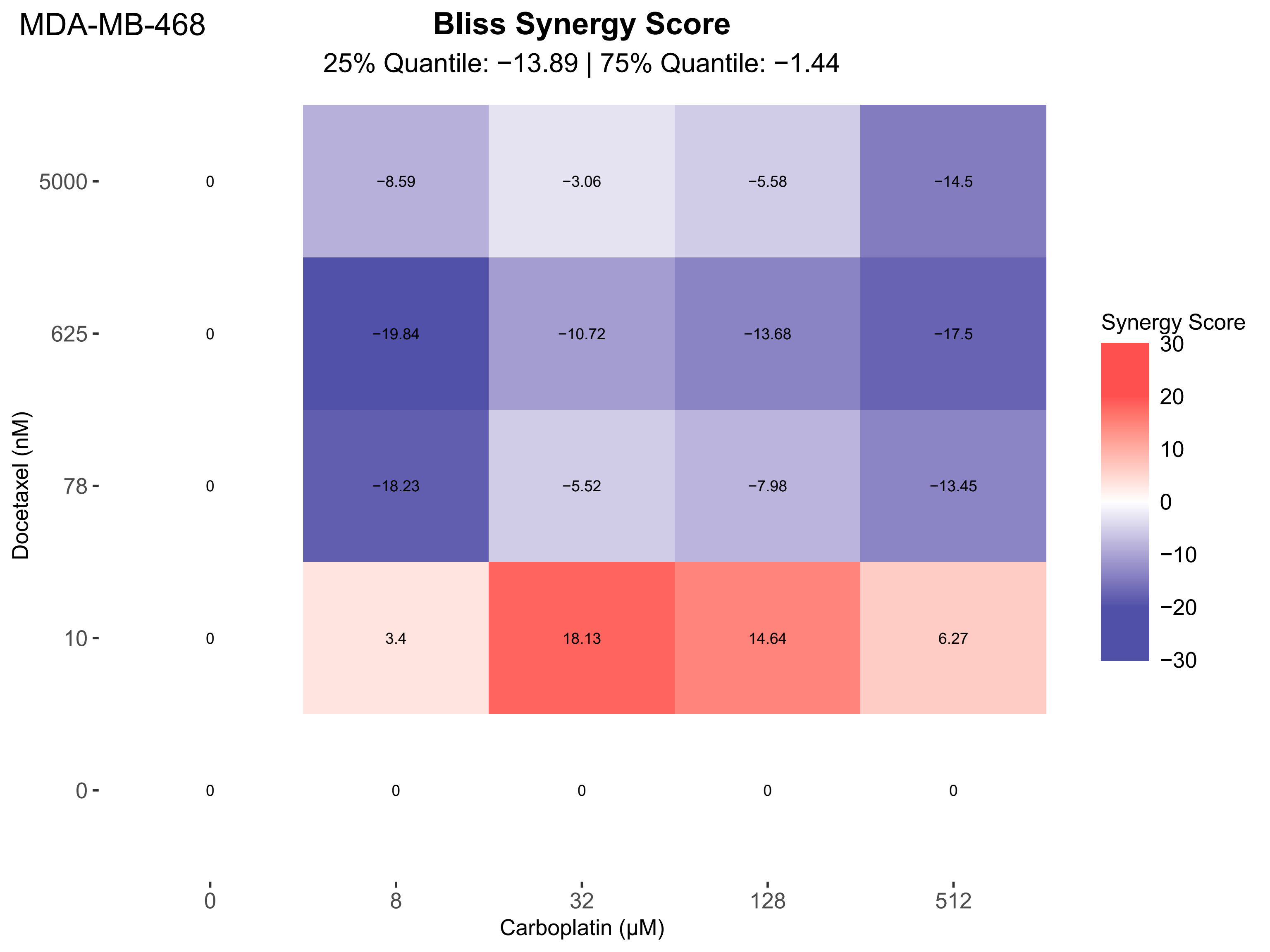
MDA-MB-468
Bliss Synergy Score
25% Quantile: −13.89 | 75% Quantile: −1.44
Docetaxel (nM)
5000
625
78
10
0
0
−8.59
−3.06
−5.58
−14.5
0
−19.84
−10.72
−13.68
−17.5
0
−18.23
−5.52
−7.98
−13.45
0
3.4
18.13
14.64
6.27
0
0
0
0
0
0
8
32
128
512
Carboplatin (μM)
Synergy Score
30
20
10
0
−10
−20
−30

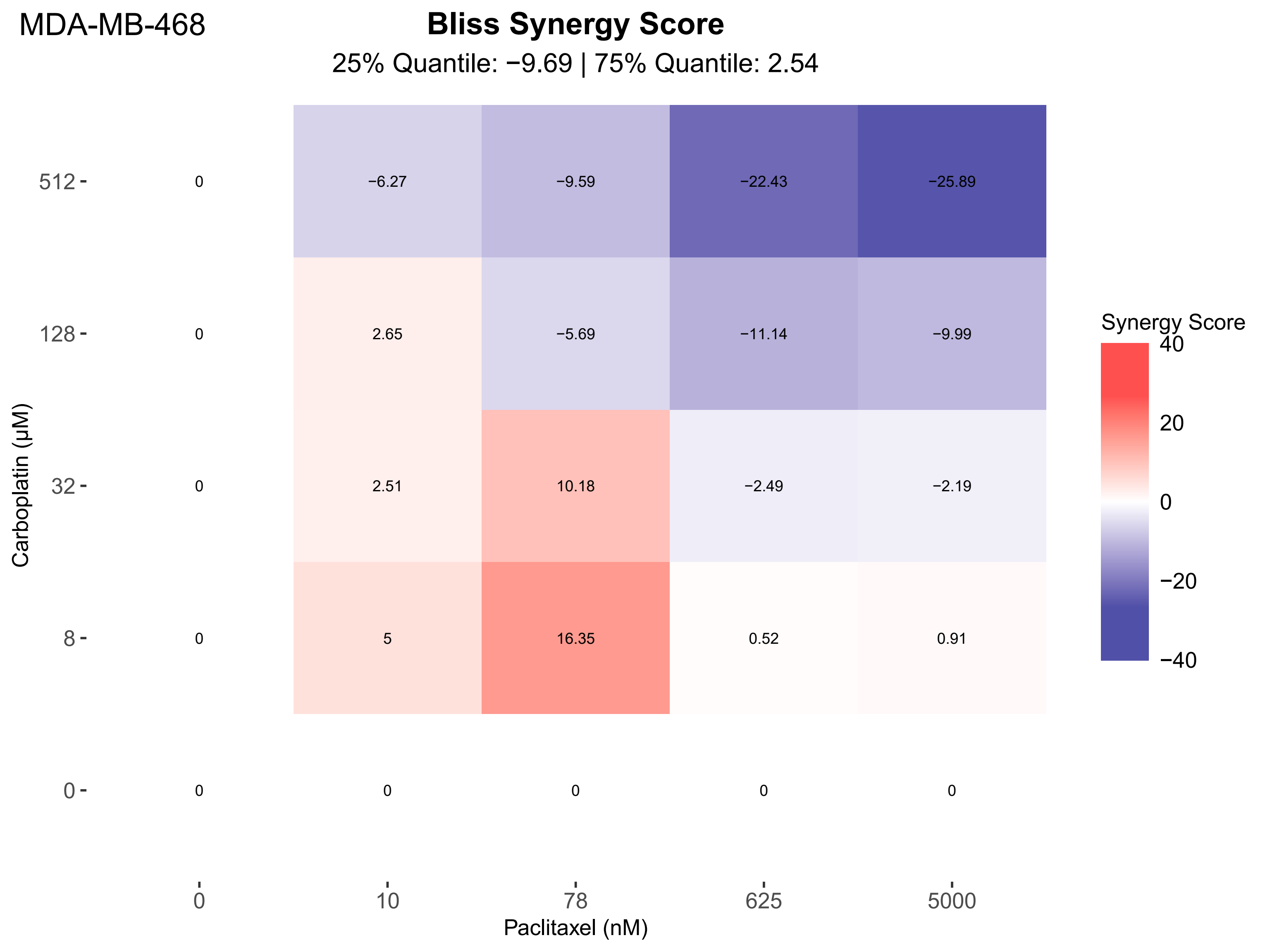
MDA-MB-468
Bliss Synergy Score
25% Quantile: −9.69 | 75% Quantile: 2.54
Carboplatin (μM)
512
128
32
8
0
Paclitaxel (nM)
0
10
78
625
5000
0
−6.27
−9.59
−22.43
−25.89
2.65
−5.69
−11.14
−9.99
2.51
10.18
−2.49
−2.19
5
16.35
0.52
0.91
Synergy Score
40
20
−20
−40

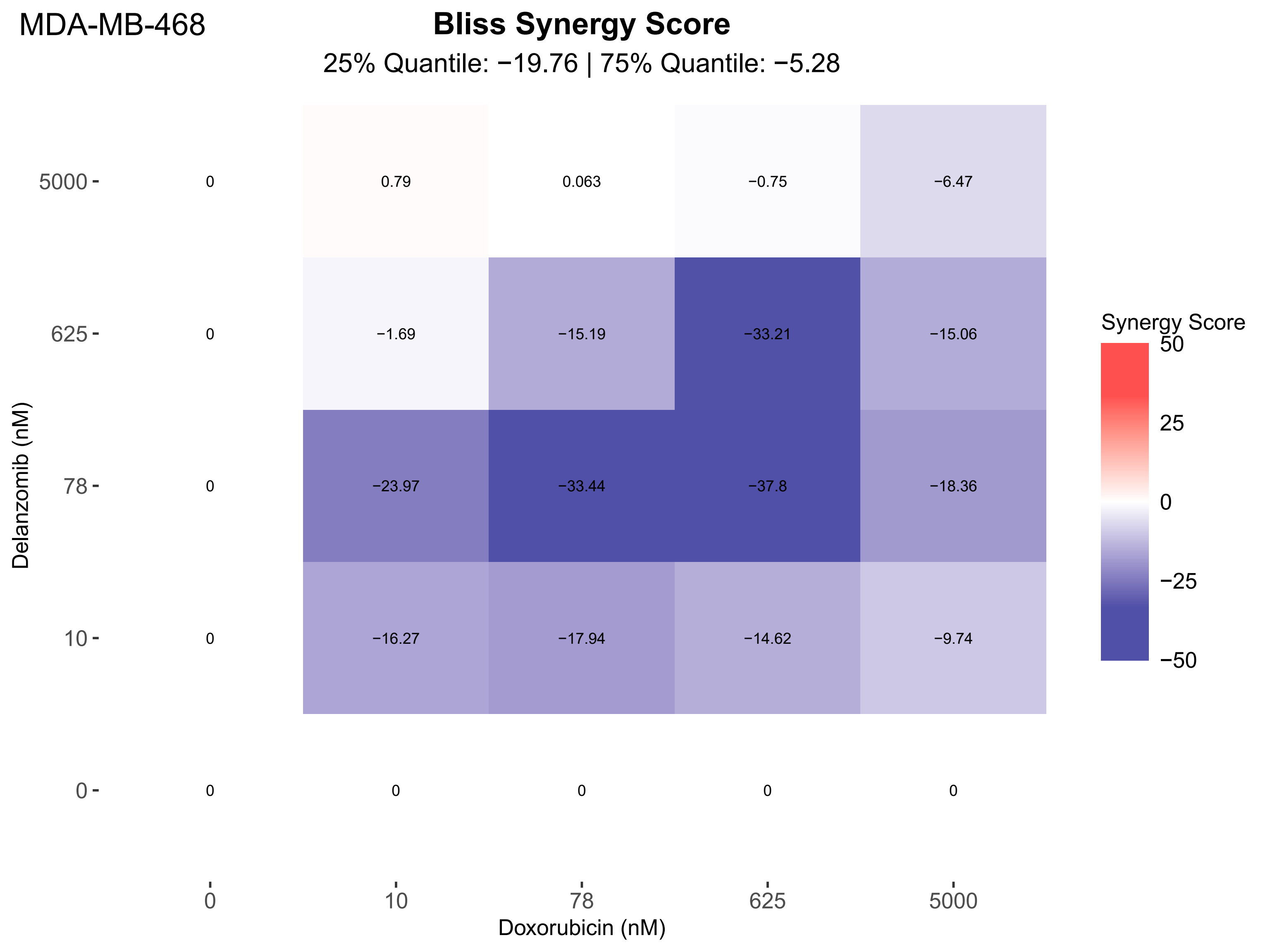
MDA-MB-468
Bliss Synergy Score
25% Quantile: −19.76 | 75% Quantile: −5.28
Delanzomib (nM)
5000
625
78
10
0
Doxorubicin (nM)
0
10
78
625
5000
0
0.79
0.063
−0.75
−6.47
0
−1.69
−15.19
−33.21
−15.06
0
−23.97
−33.44
−37.8
−18.36
0
−16.27
−17.94
−14.62
−9.74
0
0
0
0
0
Synergy Score
50
25
0
−25
−50

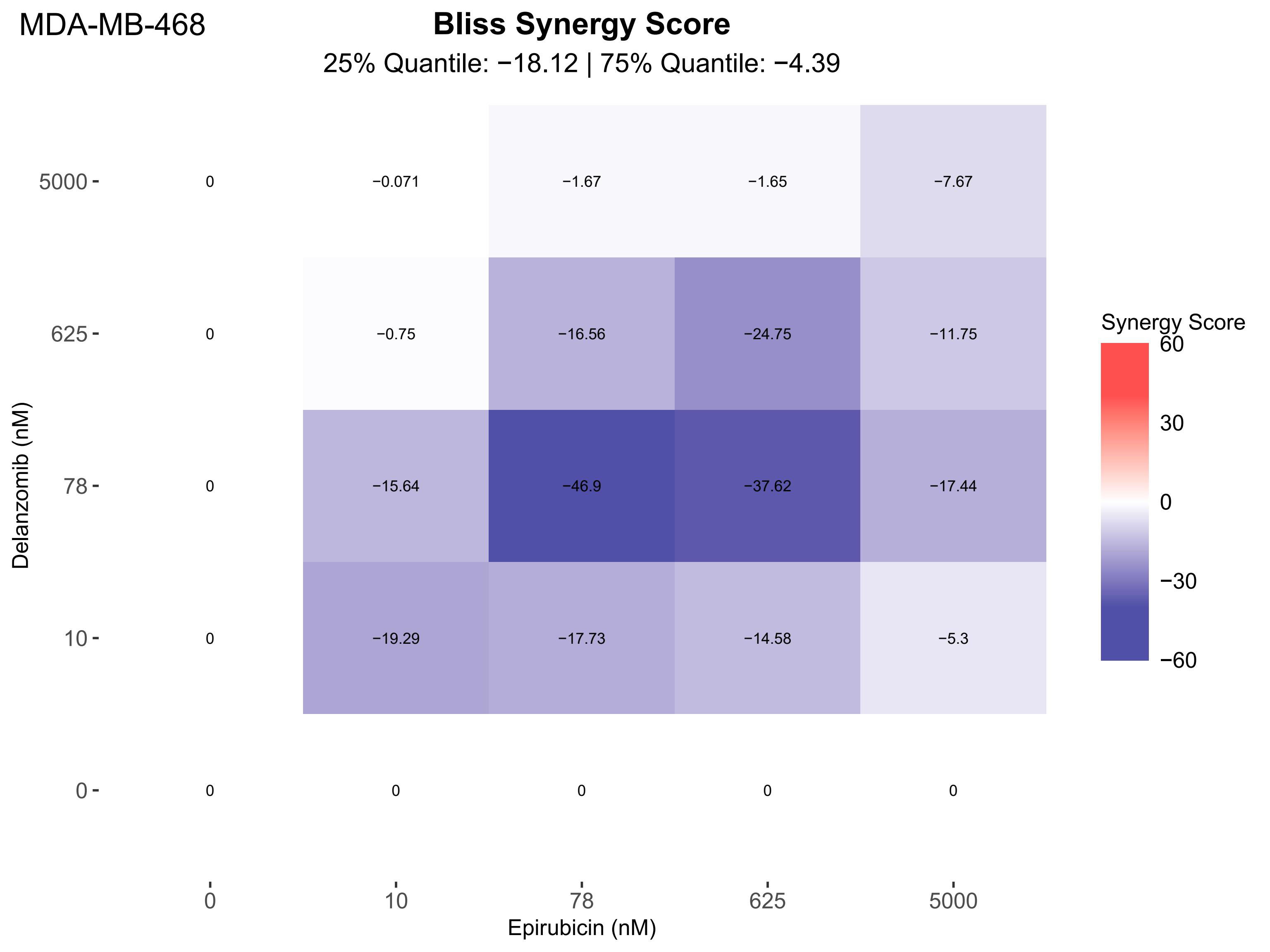
MDA-MB-468
Bliss Synergy Score
25% Quantile: −18.12 | 75% Quantile: −4.39
Delanzomib (nM)
5000
625
78
10
0
Epirubicin (nM)
0
10
78
625
5000
0
−0.071
−1.67
−1.65
−7.67
0
−0.75
−16.56
−24.75
−11.75
0
−15.64
−46.9
−37.62
−17.44
0
−19.29
−17.73
−14.58
−5.3
0
0
0
0
0
Synergy Score
60
30
0
−30
−60

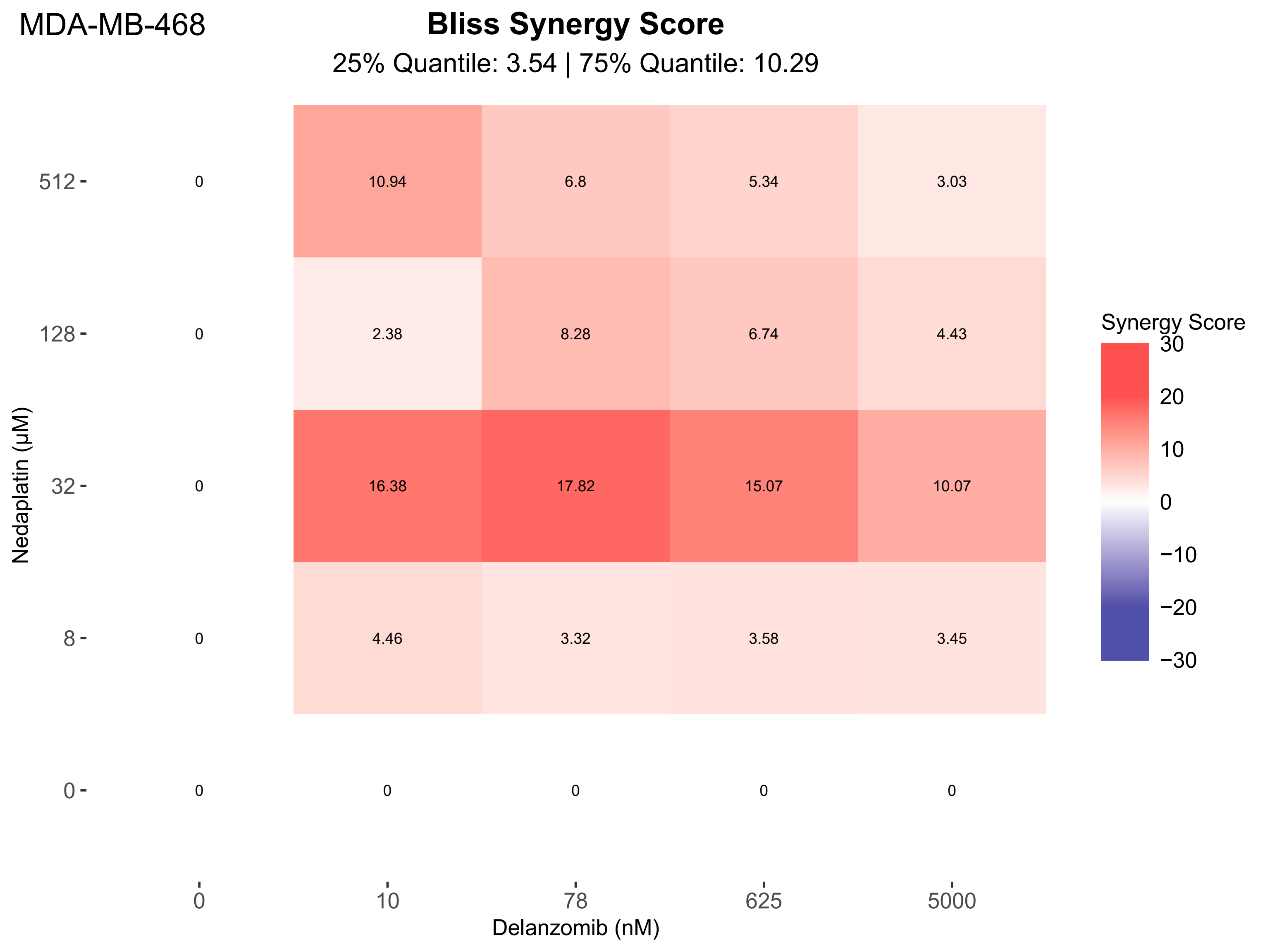

MDA-MB-468
Bliss Synergy Score
25% Quantile: 3.54 | 75% Quantile: 10.29
Nedaplatin (μM)
512
128
32
8
0
Delanzomib (nM)
0
10
78
625
5000
10.94
6.8
5.34
3.03
2.38
8.28
6.74
4.43
16.38
17.82
15.07
10.07
4.46
3.32
3.58
3.45
Synergy Score
30
20
10
0
−10
−20
−30

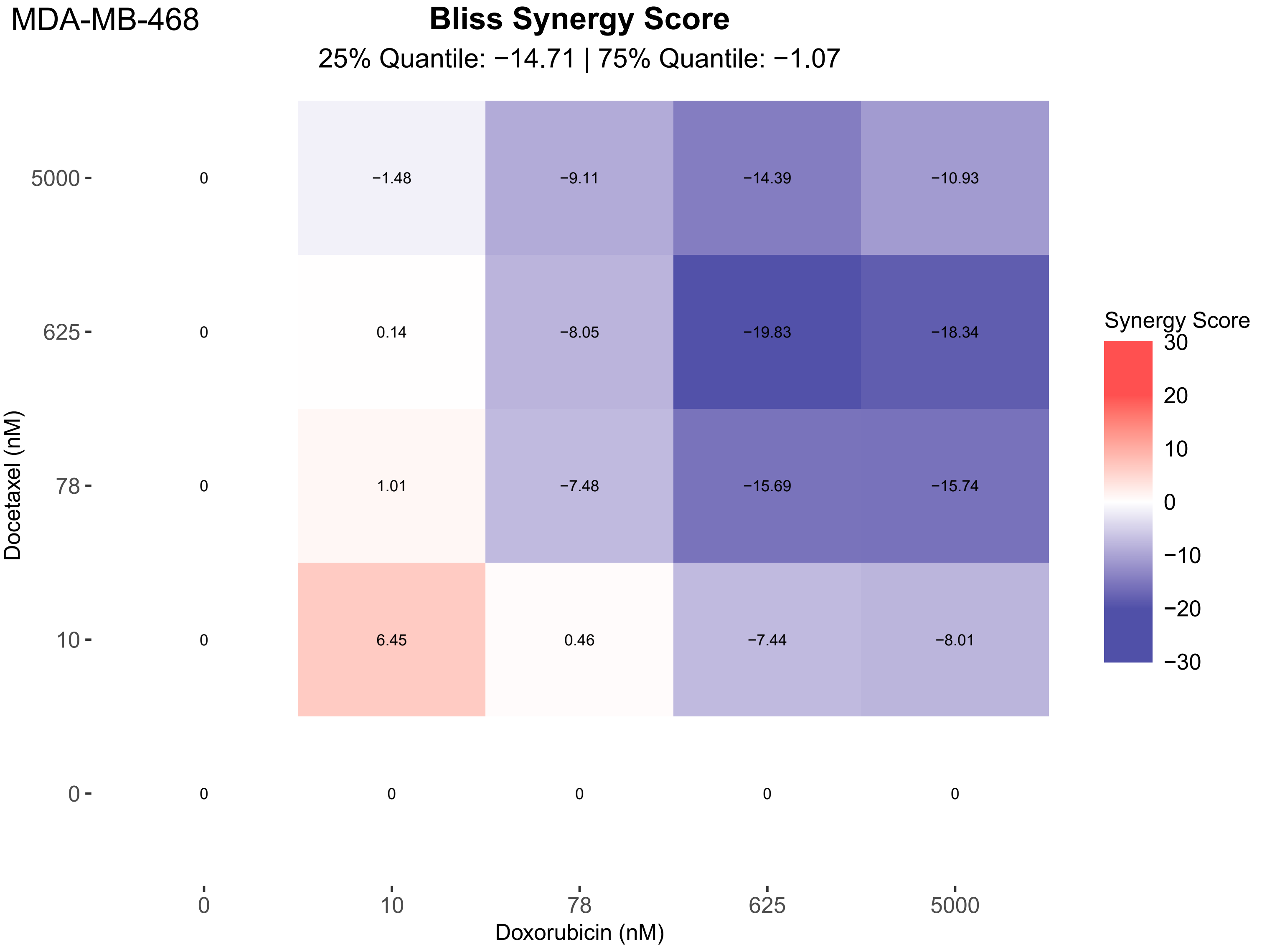
MDA-MB-468
Bliss Synergy Score
25% Quantile: −14.71 | 75% Quantile: −1.07
Docetaxel (nM)
5000
625
78
10
0
Doxorubicin (nM)
0
10
78
625
5000
0
−1.48
−9.11
−14.39
−10.93
0
0.14
−8.05
−19.83
−18.34
0
1.01
−7.48
−15.69
−15.74
0
6.45
0.46
−7.44
−8.01
0
0
0
0
0
Synergy Score
30
20
10
0
−10
−20
−30

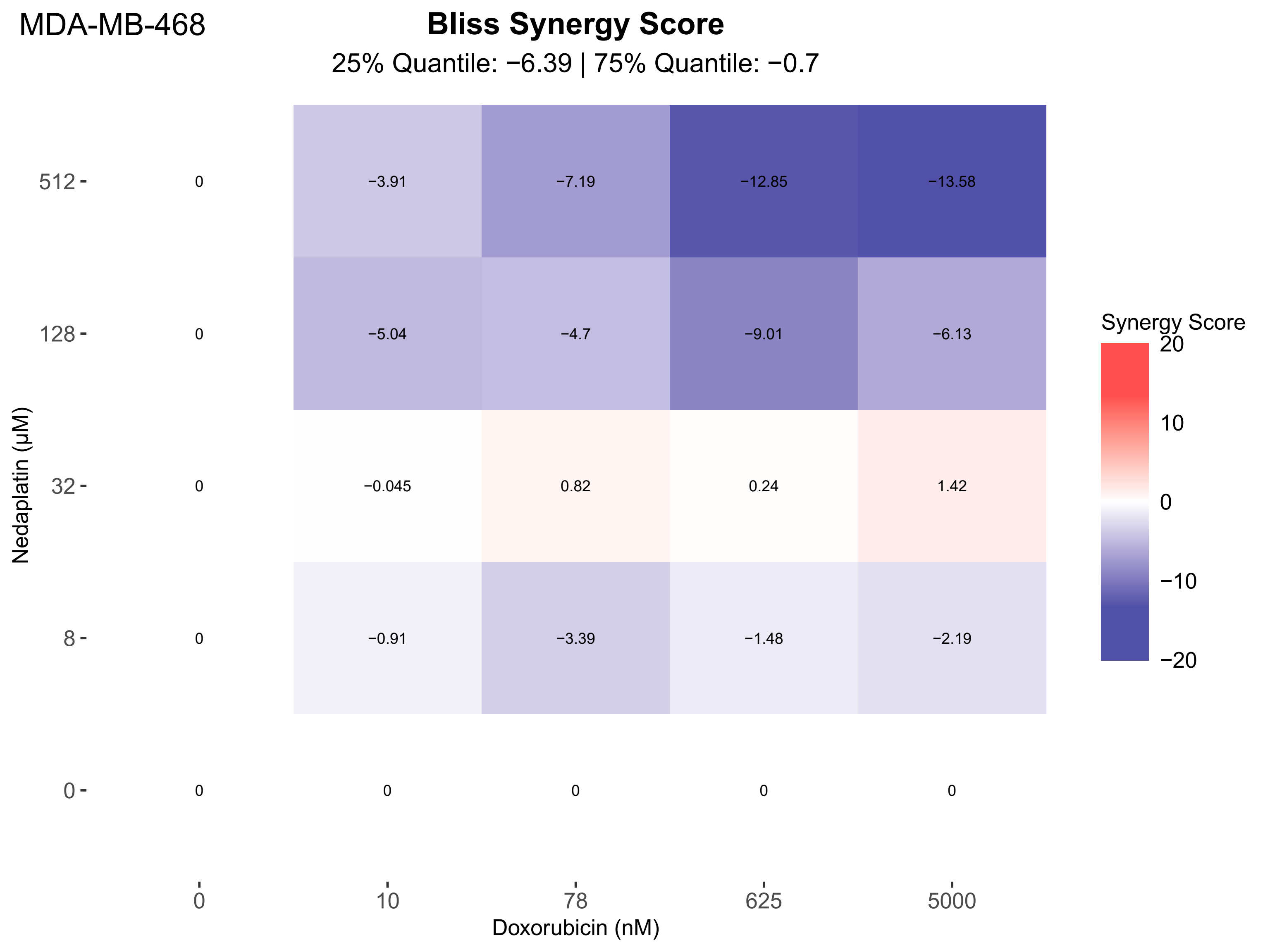
MDA-MB-468
Bliss Synergy Score
25% Quantile: −6.39 | 75% Quantile: −0.7
Nedaplatin (μM)
512
128
32
8
0
Doxorubicin (nM)
0
10
78
625
5000
0
−3.91
−7.19
−12.85
−13.58
0
−5.04
−4.7
−9.01
−6.13
0
−0.045
0.82
0.24
1.42
0
−0.91
−3.39
−1.48
−2.19
0
0
0
0
0
Synergy Score
20
10
0
−10
−20

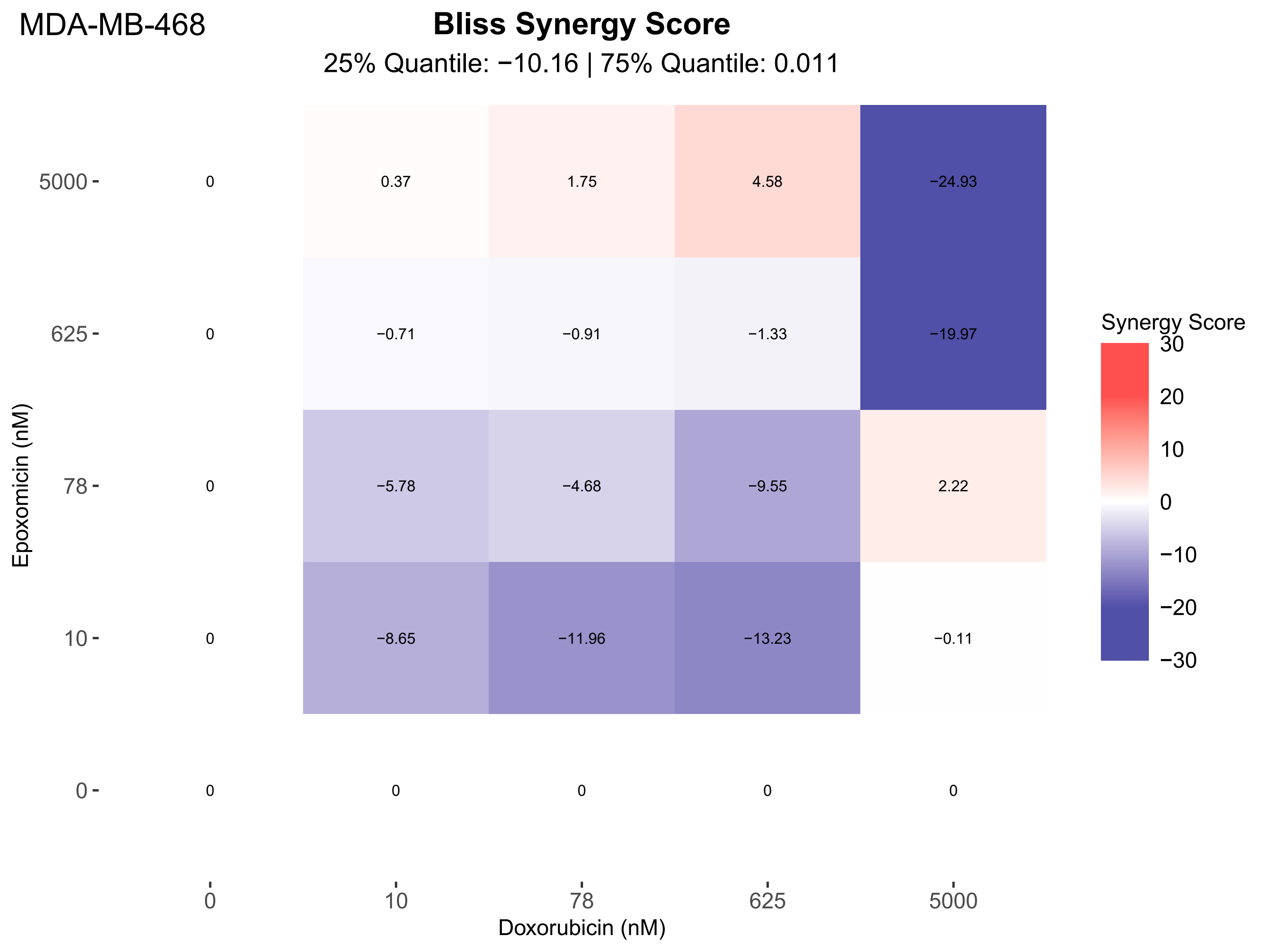
MDA-MB-468
Bliss Synergy Score
25% Quantile: −10.16 | 75% Quantile: 0.011
Epoxomicin (nM)
5000
625
78
10
0
Doxorubicin (nM)
0
10
78
625
5000
0 0.37 1.75 4.58 −24.93
0 −0.71 −0.91 −1.33 −19.97
0 −5.78 −4.68 −9.55 2.22
0 −8.65 −11.96 −13.23 −0.11
0 0 0 0 0
Synergy Score
30
20
10
0
−10
−20
−30

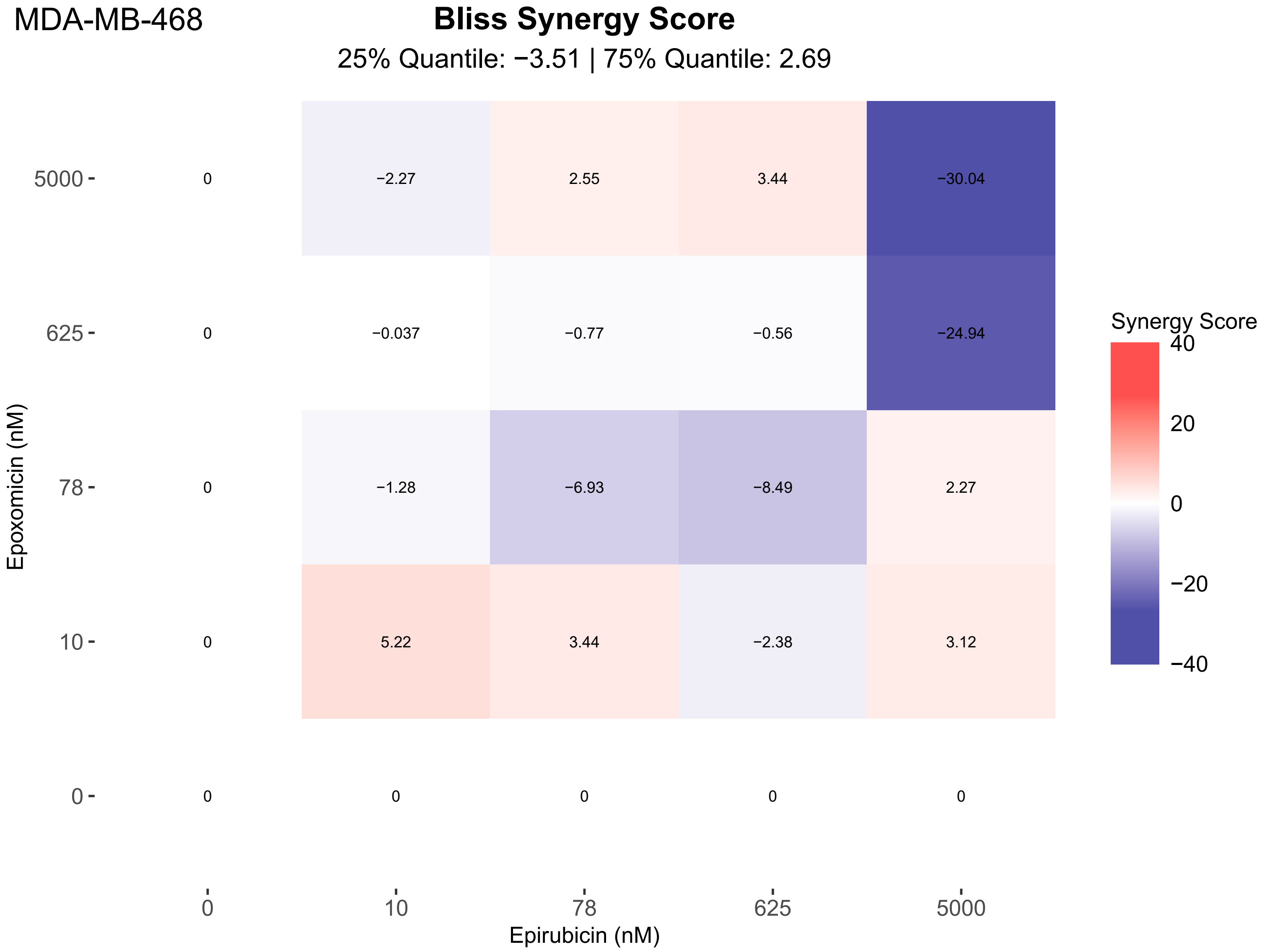

MDA-MB-468
Bliss Synergy Score
25% Quantile: −3.51 | 75% Quantile: 2.69
Epoxomicin (nM)
5000
625
78
10
0
0
−2.27
2.55
3.44
−30.04
0
−0.037
−0.77
−0.56
−24.94
0
−1.28
−6.93
−8.49
2.27
0
5.22
3.44
−2.38
3.12
0
0
0
0
0
0
10
78
625
5000
Epirubicin (nM)
Synergy Score
40
20
0
−20
−40

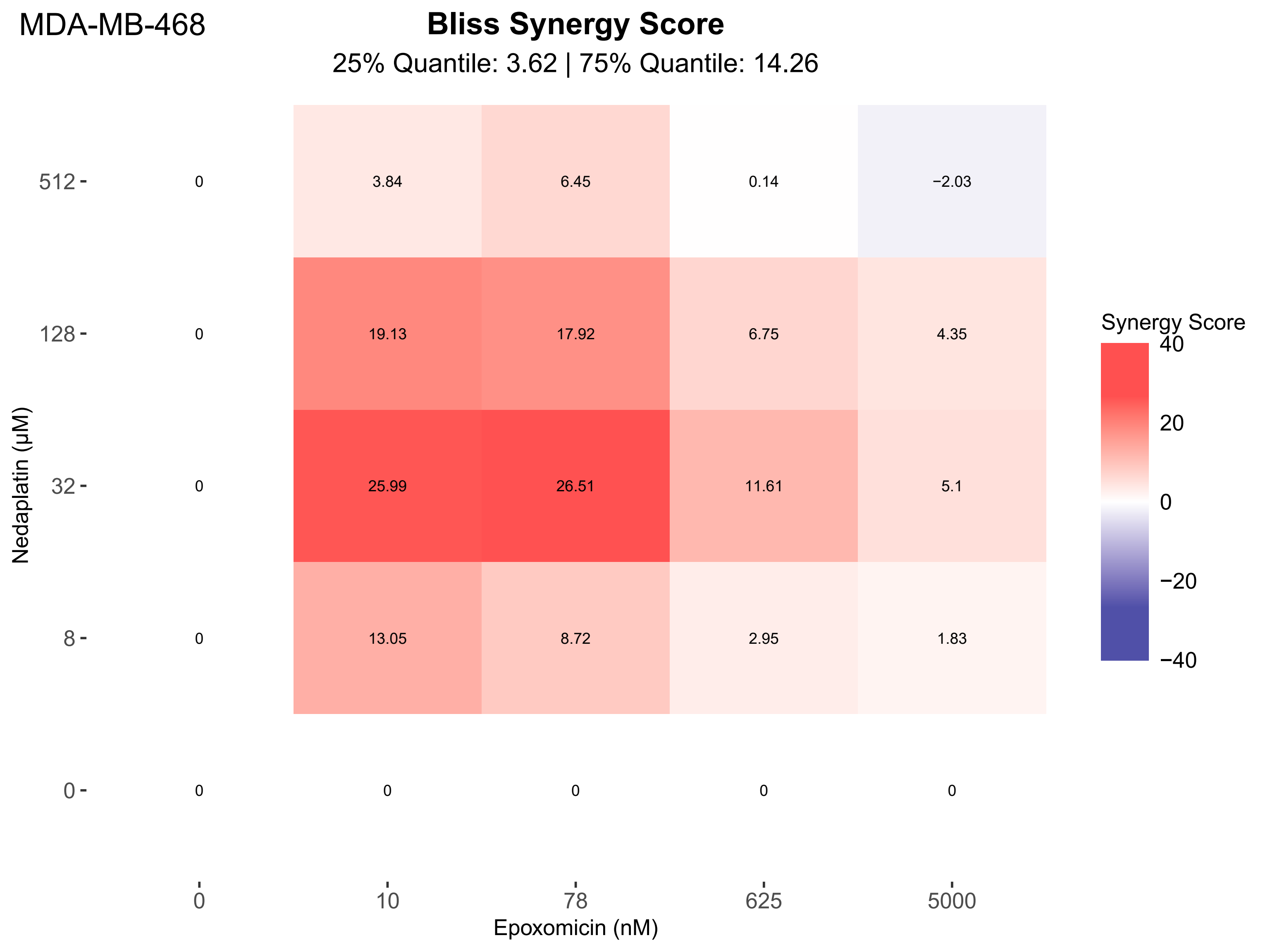
MDA-MB-468
Bliss Synergy Score
25% Quantile: 3.62 | 75% Quantile: 14.26
Nedaplatin (μM)
512
128
32
8
0
Epoxomicin (nM)
0
10
78
625
5000
3.84
6.45
0.14
−2.03
19.13
17.92
6.75
4.35
25.99
26.51
11.61
5.1
13.05
8.72
2.95
1.83
Synergy Score
40
20
0
−20
−40
